# Factors Influencing Continuous Breath Signal in Intubated and Mechanically-Ventilated Intensive Care Unit Patients Measured by an Electronic Nose

**DOI:** 10.3390/s16081337

**Published:** 2016-08-22

**Authors:** Jan Hendrik Leopold, Ameen Abu-Hanna, Camilla Colombo, Peter J. Sterk, Marcus J. Schultz, Lieuwe D. J. Bos

**Affiliations:** 1 Department of Intensive Care, Academic Medical Center, University of Amsterdam, Amsterdam 1100DD, The Netherlands; marcus.j.schultz@gmail.com (M.J.S.); l.d.bos@amc.uva.nl (L.D.J.B.); 2 Department of Medical Informatics, Academic Medical Center, University of Amsterdam, Amsterdam 1100DD, The Netherlands; a.abu-hanna@amc.uva.nl (A.A.-H.); colombo.cam@gmail.com (C.C.); 3 Department of Respiratory Medicine, Academic Medical Center, University of Amsterdam, Amsterdam 1100DD, The Netherlands; p.j.sterk@amc.uva.nl

**Keywords:** electronic nose, exhaled breath, ICU, critical care, mechanically-ventilated, continuous, pre-processing, noise

## Abstract

Introduction: Continuous breath analysis by electronic nose (eNose) technology in the intensive care unit (ICU) may be useful in monitoring (patho) physiological changes. However, the application of breath monitoring in a non-controlled clinical setting introduces noise into the data. We hypothesized that the sensor signal is influenced by: (1) humidity in the side-stream; (2) patient-ventilator disconnections and the nebulization of medication; and (3) changes in ventilator settings and the amount of exhaled CO_2_. We aimed to explore whether the aforementioned factors introduce noise into the signal, and discuss several approaches to reduce this noise. Methods: Study in mechanically-ventilated ICU patients. Exhaled breath was monitored using a continuous eNose with metal oxide sensors. Linear (mixed) models were used to study hypothesized associations. Results: In total, 1251 h of eNose data were collected. First, the initial 15 min of the signal was discarded. There was a negative association between humidity and Sensor 1 (Fixed-effect β: −0.05 ± 0.002) and a positive association with Sensors 2–4 (Fixed-effect β: 0.12 ± 0.001); the signal was corrected for this noise. Outliers were most likely due to noise and therefore removed. Sensor values were positively associated with end-tidal CO_2_, tidal volume and the pressure variables. The signal was corrected for changes in these ventilator variables after which the associations disappeared. Conclusion: Variations in humidity, ventilator disconnections, nebulization of medication and changes of ventilator settings indeed influenced exhaled breath signals measured in ventilated patients by continuous eNose analysis. We discussed several approaches to reduce the effects of these noise inducing variables.

## 1. Introduction

### 1.1. Introduction into Breath Analysis

Analysis of exhaled breath has received increasing attention over the last years as a potential diagnostic tool for a variety of diseases. Several technologies are available for breath analysis [1]. Gas chromatography and mass-spectrometry (GC-MS) is an often used technique to obtain a detailed snapshot of the volatile organic compounds (VOCs) in breath. Therefore, GC-MS is well-suited for biomarker discovery and discrimination between disease-states at a single time-point. However, for clinical practice, monitoring of dynamic processes is frequently more desired than a singular measurement representing a stable state. Monitoring of several specific VOCs has been successfully attempted with proton-transfer reaction time-of-flight mass-spectrometry (PTR-TOF-MS) [2]. The PTR-TOF-MS device however is large, with limited possibility for miniaturization, expensive and data analysis is elaborate. Another possibility for continuous analysis of VOCs is by cheaper and more portable electronic nose (eNose) technology [3,4,5,6,7,8,9,10,11,12]. Many eNose studies have shown its potential both in the field of medicine [13,14,15,16] and in other fields [17,18,19]. eNoses contain an array of cross-reactive sensors. The physical properties (such as electrical conductivity) of these sensors change upon exposure to certain VOCs. The sensors are typically very sensitive for a wide range of VOCs but show remarkable cross-reactivity. Therefore, the analysis of eNose data relies on pattern recognition.

### 1.2. Ventilated ICU Patients

Intensive care unit (ICU) patients are per definition severely ill and their physiology is easily disturbed, which makes their clinical condition highly unstable. Therefore, there is a need for continuous monitoring, as is standard of care for several physiological parameters such as pulse oxymetry and end-tidal CO_2_ monitoring in exhaled air [20,21]. The latter is an example that illustrates the advantage of breath analysis over other bio-monitoring techniques, such as frequent blood draws and expensive blood gas analyses. Therefore, breath analysis may be desirable as bio-monitoring technique in the ICU and, because of constant access to breath in intubated and mechanically-ventilated patients, continuous breath analysis is highly feasible. Breath can be collected and analyzed fully non-invasively in these patients [22], but so far continuous measurements have not been investigated. Therefore, several challenges have to be faced before safe and meaningful continuous analysis of the exhaled breath is possible in intubated and ventilated ICU patients. Continuous exhaled breath analysis should be safe and should never interfere with any of the clinical activities, such as ventilatory support and monitoring. However, the application in a non-controlled, non-laboratory setting is likely to introduce noise into the data. The aim of this study was to evaluate noise inducing variables in exhaled breath signals as obtained through an eNose sensor in a non-controlled setting and to discuss several approaches to reduce noise. We hypothesized that the sensor signal was influenced by: (1) the humidity in the side-stream, as the sensor are cross-reactive with water; (2) patient-ventilator disconnections and the nebulization of medication; and (3) changes in ventilator settings and/or exhaled CO_2_.

## 2. Methods

### 2.1. Study Design and Population

Intubated and mechanically-ventilated ICU patients, expected to remain mechanically-ventilated for at least 48 h, were eligible for this study. Additionally, patients had to be older than 18 years. Patients were excluded when they were not expected to survive for a considerable amount of time. The Institutional Review Board of the Academic Medical Center, Amsterdam, The Netherlands concluded that the legislation on human participation in research was not applicable because of the non-invasive, non-interventional nature of the study.

### 2.2. Standard of Care

Standard of ventilatory support for intubated and mechanically-ventilated patients on the ICU of the Academic Medical Center in Amsterdam included, but was not limited to: Pressure Support or Pressure Controlled mode of ventilation, tidal volume of 6–8 mg/kg predicted body weight, a level of Positive End Expiratory Pressure (PEEP) ≥5 cm H_2_O, nebulization of acetylcysteine and salbutamol, and a pulmonary toilet including suction of secretions every 6 h. In addition, a heat-moist exchanger was placed in the circuit and active humidification was not used routinely. Mechanical ventilators from several vendors were used. Arterial blood gas measurements were taken on indication, with a minimum of 3 measurements per day. These factors, and other aspects of clinical care were not influenced by study procedures.

### 2.3. Study Procedures and Data Collection

**Figure 1:**
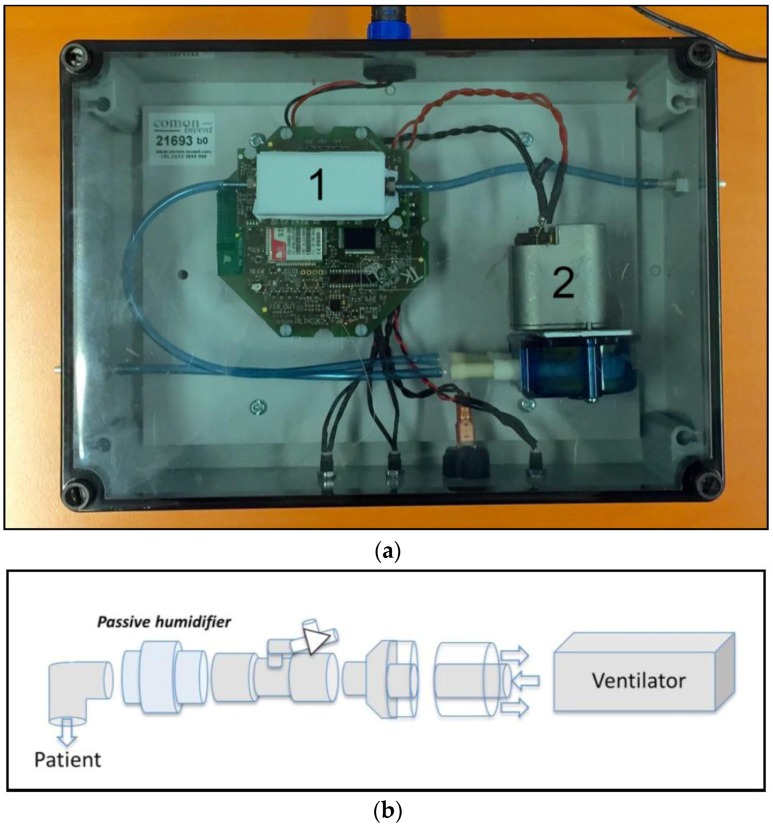
eNose and Side stream of the that is created to connect the eNose. In the upper panel (**a**), the eNose that is used in our study is pictured: (1) is the sensor block containing the metal oxide sensors; and (2) is the pump that is used to pump exhaled breath over the metal oxide sensors. The lower panel (**b**) illustrates the side stream of that is created to connect the eNose. A T-piece is connected distal of the heat moist exchanger in the ventilation circuit.

Exhaled breath was monitored in patients of the mixed medical/surgical ICU of the Academic Medical Center in Amsterdam, the Netherlands using a continuous eNose adapted for clinical use in the intensive care unit (Comon Invent, Delft, The Netherlands). The sensor array contained 4 different metal oxide sensors (Figaro, Japan), which were chosen for their stability, clinical potential, performance and because they were widely used [14,23,24]. Tin dioxide was used as sensing material and the metal oxide sensors could operate between −40 and +70 °C. The device was similar to that described by De Vries et al. [14], with the following adaptions: (1) it consists of a single sensor array; (2) it has a roller-pump to continuously supply exhaled breath at a flow of 35 mL/min; (3) it has a plastic outer body to allow for thorough cleaning; and (4) it has an offline modus that disabled mobile connectivity to prevent interference with the mechanical ventilator and other devices [25]. The eNose was connected to the expiratory tube of the ventilator using a T-piece to create a side-stream. This is illustrated in Figure 1. The flow of 35 mL/min to the eNose was not enough to trigger a ventilator alarm in our experiment. Data of the metal oxide sensors and a humidity sensor were stored every minute. Ventilator data were automatically stored in the Patient Data Management System (PDMS).

### 2.4. Data Analysis

Data analysis was performed using R version 3.2.4 (R Foundation, Vienna, Austria).

#### 2.4.1. Noise Inducing Variables

**Figure 2:**
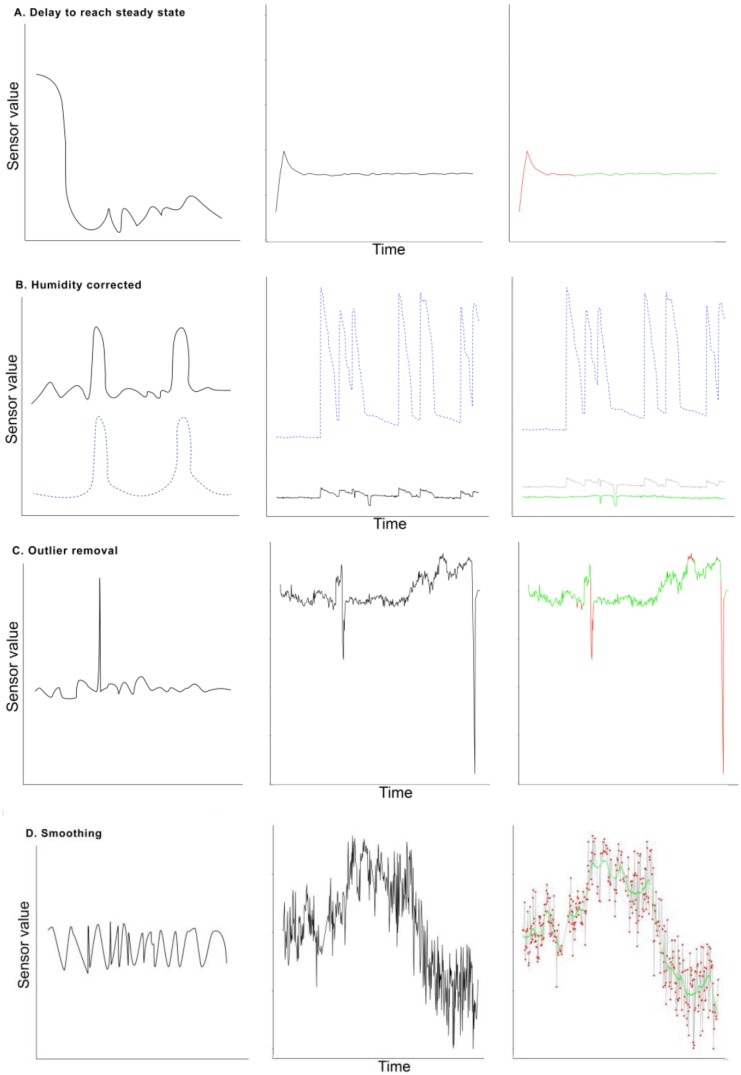

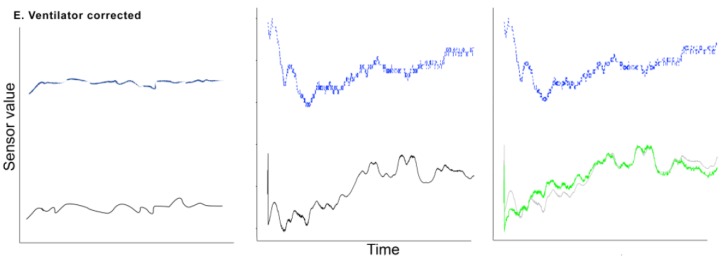
Noise inducing variables in continuous breath signals of ventilated intensive care unit patients (**A**–**E**). From left to right, Panel 1: schematic representation of type of noise; Panel 2: example of the noise in the data, where blue dashed lines indicate humidity in (**B**) and an example of a ventilator signal (end tidal CO_2_) in (**E**); and Panel 3: example after correction for the noise type, where green line indicates sensor signal after noise reduction step, red line indicates discarded signal values. In (**D**), the grey line represents the initial values of one of the eNose sensors, the green line corresponds to the LOESS smoother and the red dots are values that are above the set threshold and are therefore considered outliers.

Noise inducing variables in the data were identified by plotting data for visual inspection by the investigators. Changes occurring concurrently in both sensor signals and variables were noted. The major noise inducing variables are plotted in Figure 2. These include time delay to reach steady state, changes in humidity in the side-stream connector, disconnections, nebulization of medication and changing ventilator settings. Each of these causes of noise will be discussed in detail below.

#### 2.4.2. Time Delay to Reach Steady State

The sensors of the eNose have to adapt to the changing substances in the air when connecting an eNose to a subject. The left panel in Figure 2A shows this schematically. Therefore, the first measurements after connecting the eNose cannot be relied on and must be deleted from the data. Different time periods were investigated and the period that resulted in stabilization in all patients was chosen.

#### 2.4.3. Changes in Humidity

The humidity in side-stream connector leading to the eNose may cause a change in the sensor response, unrelated to the actual VOC profile (Figure 2B). The association between humidity and sensor response was investigated by Pearson’s correlation coefficient. A linear regression model was fitted with the sensor response as dependent variable and the humidity as independent variable. Standardized residuals of these regression models were used to replace sensor variables as these values are corrected for the variance imposed by changes in humidity.

#### 2.4.4. Outlier Removal and Smoothing

After correcting for humidity, outliers, particularly those due to intermittent disconnections and nebulization of medication, were removed. As short periods of extreme values are most likely to be erroneous, we chose to discard the top and bottom 2.5% of the measurements. This removes the most prominent peaks and dips. Then, a LOESS smoother with default settings was calculated, after which the relative error between the signal and the smoother was computed. When this relative error was above a set threshold at a point in the data, this data point was replaced by the value of the LOESS smoother. Finally, to illuminate the worst jitter, the signal was smoothed using a LOESS smoother, with a span of 30 observations.

#### 2.4.5. Changes in Ventilation Settings

Changes in ventilation settings could have a big influence on the eNose signal. It can be imagined that a change in tidal volume, PEEP or minute volume may influence the abundance of measured VOCs. Ventilation settings stored in the PDMS were used to correct for this phenomenon. A similar strategy as used for correcting for changes in humidity was followed. However, instead of a linear regression model, a mixed model with the patient number as random effect was used. We investigated the available relevant variables: changes in minute volume, changes in end tidal CO_2_ (ETCO_2_), changes in tidal volume, inspiratory pressure, peak pressure and PEEP. Backwards variable selection was used to eliminate non-significant effects. Finally, a LOESS smoother with a span of 15 observations was used once more to correct for jitter introduced after ventilator variable correction.

## 3. Results of Noise Reduction

Between October 2012 and July 2015, 1251 h of eNose data were collected in 23 different patients. Patient characteristics are shown in Table 1.

### 3.1. Steady State

The first fifteen minutes of sensor signal was discarded to allow the sensors to adapt to the new circumstances. A shorter period did not result in a steady state in all patients. The middle and right panels in Figure 2A illustrate this.

### 3.2. Changes in Humidity

**Figure 3:**
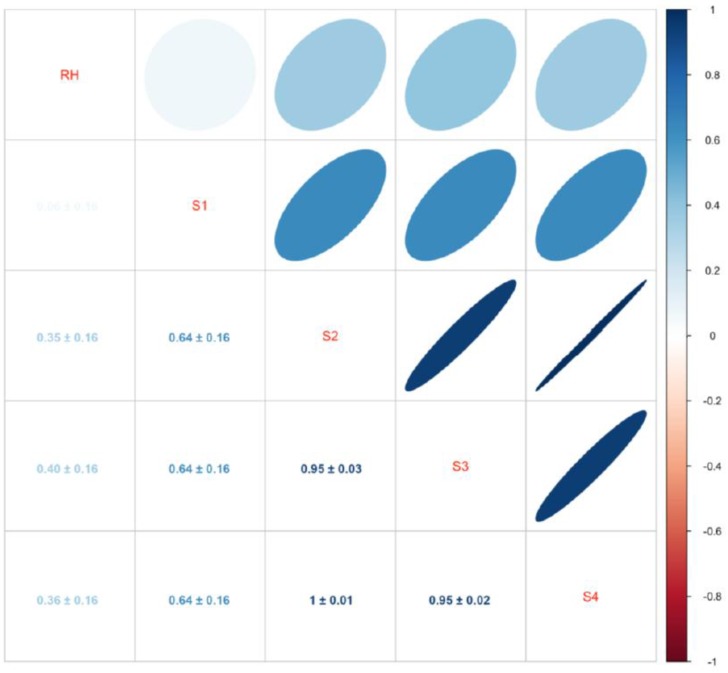
Correlation between relative humidity (RH) and Sensors 1–4. Values indicate mean fixed effect regression coefficients ± their 95% confidence intervals. Blue indicates positive correlation while red indicates negative correlation. Darker more elliptical shapes indicate greater correlation.

**Figure 4:**
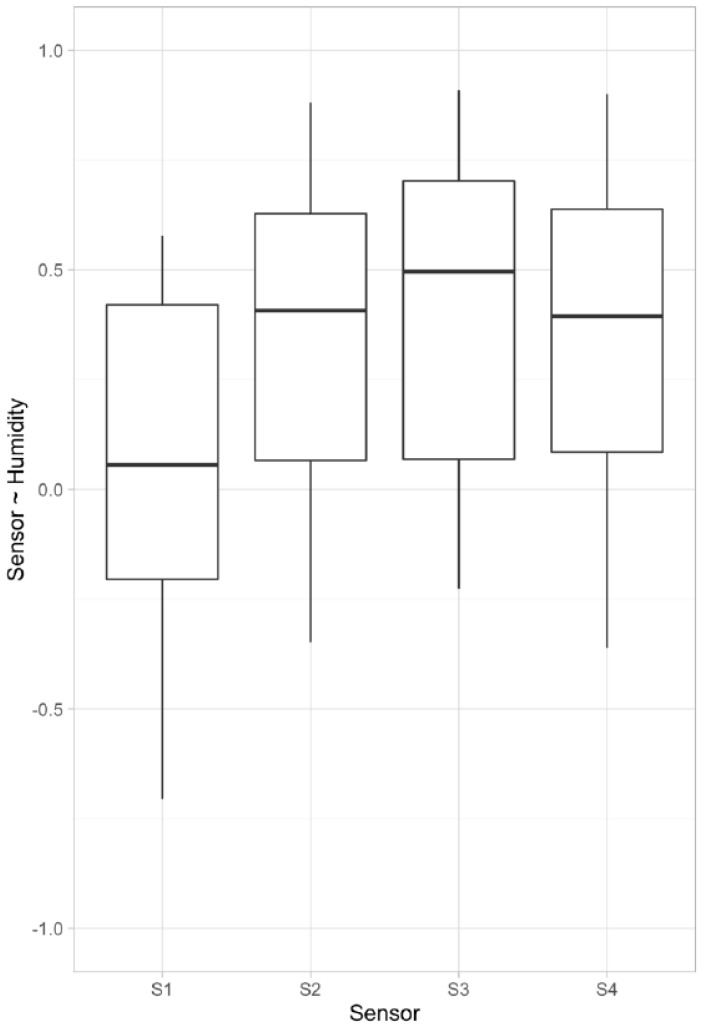
Mean correlation coefficient R between sensors and relative humidity. Upper and lower “hinges” correspond to first and third quartile, respectively, and whiskers indicate 95% confidence interval.

Figure 3 illustrates the mean correlation between sensors and relative humidity (RH) of all included patients. Influence of changes in humidity differed among patients, but it indicates that humidity indeed introduced noise into the signal. In Figure 2B, influence of humidity, and signal after correcting for it are found. Correlation matrixes and plots for each individual patient can be found in Supplementary Material 1. The fixed effect of the mixed effects model for each sensor can be found in Table 2; there was a negative association between humidity and Sensor 1 (−0.05 ± 0.002) and a positive association with Sensors 2–4 (0.12 ± 0.002). The correlation coefficient *r* between humidity and eNose sensors for each patient can be found in Figure 4.

### 3.3. Outlier Removal and Smoothing

The middle and right panel of Figure 2C illustrate one of the sensor signals before and after outlier removal. As the example in the right panel demonstrates, large influential peaks and dips caused by outliers were removed from the signal. By removing theses peaks, a less aggressive LOESS smoother was necessary to remove the remaining outliers and jitter from signal. This process is illustrated in the right panel of Figure 2D. Plots for all other patients are found in Supplementary Materials.

### 3.4. Changes in Ventilation Settings

**Figure 5:**
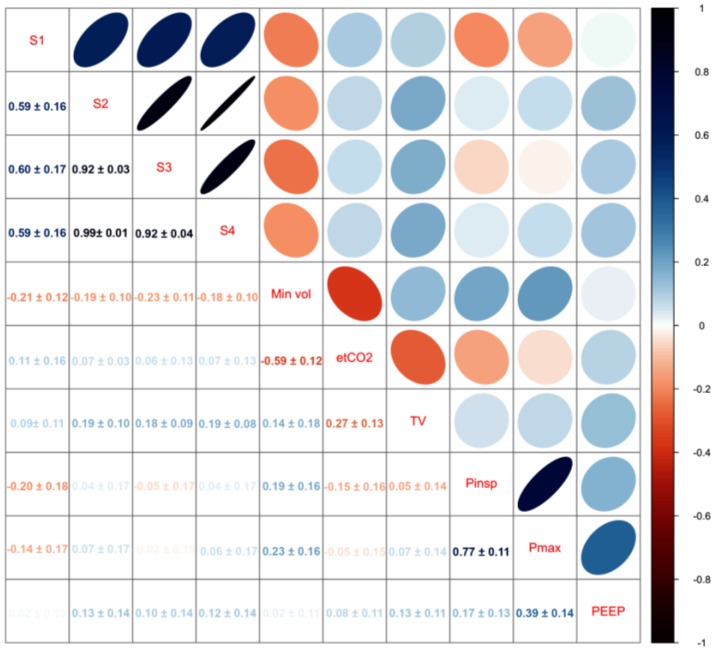
Correlation between ventilator variables and Sensors 1–4. Values indicate mean fixed effect regression coefficients ± their 95% confidence intervals. Blue indicates positive correlation while red indicates negative correlation. Darker more elliptical shapes indicate greater correlation.

**Figure 6:**
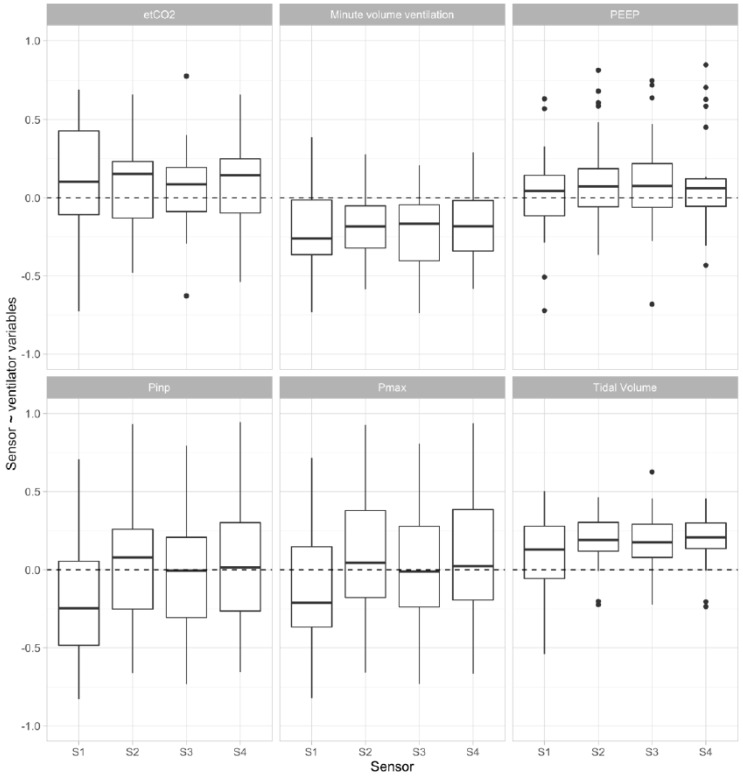
Mean correlation coefficient R between sensors and ventilator variables. Upper and lower “hinges” correspond to first and third quartile, respectively, and whiskers indicate 95% confidence interval.

**Figure 7:**
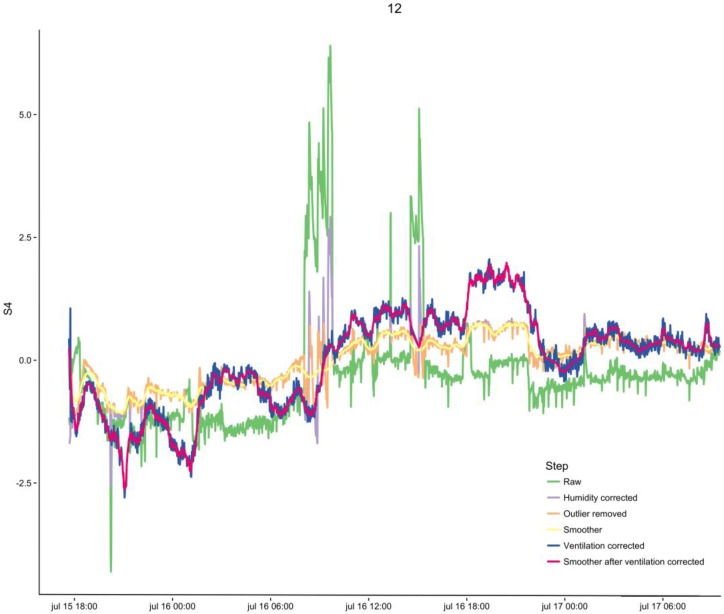
eNose signal for one sensor in one patient after each pre-processing step.

A matrix with mean correlation values between ventilator readings and sensors of all patients in our study is shown in Figure 5. The fixed effect of the mixed effects model for each sensor can be found in Table 3. Since ventilation settings varied greatly between patients, there was not one setting that seemed highly correlated with sensor signals in every patient (Figure 5 and Figure 6). Nonetheless, when plotting raw sensor values and ventilator settings, change in ventilator settings seems to influence sensor values (Figure 2E). In addition, changes in settings were more likely to occur when patients were monitored for a longer period of time. After backwards variable selection, none of the variables was eliminated and sensors were corrected for all pre-defined variables. Table 4 shows the fixed effects of the mixed effects models after correcting for ventilator variables. Sensor values were positively associated with end-tidal CO_2_, tidal volume and the variables. Figure 7 illustrates the signal in one patient after each pre-processing step.

## 4. Discussion

The presented results suggest that humidity, ventilator disconnections, nebulization of medication and ventilator settings indeed influenced exhaled breaths signal measured in ventilated patients by continues eNose analysis. We described several approaches to reduce these types of noise. This implies that direct translation of breath analysis technology developed for singular measurements is impossible; the signal should be corrected in a multi-step fashion to remove noise that correlates with variations in patient care that are not directly linked to the (patho) physiological process of interest.

This influence on the signal is explained by the following considerations. First, the commonly used metal oxide sensors that we also used in this study are known to be influenced by humidity [23,28]. Therefore, the eNose is connected behind the heat-moist exchanger of the ventilation circuit to minimize the influence of moist. The influence of moisture was further diminished by correcting the signal for the remaining fluctuations in humidity. Second, disconnections of the eNose or ventilation circuit had a major effect on the signal because of a sudden inlet of ambient air. This leads to lower concentration of exhaled compounds, but an increase in concentration of, for example, ethanol. This has a large impact on sensor values. Third, frequent nebulization of medication such as acetylcysteine and salbutamol can influence sensor signals. When nebulized medication is not completely absorbed by the lungs and is consequently partially exhaled, it could possibly bind the eNose sensors and inflict a change in sensor signal. Finally, changes in ventilator settings were associated with sensor readings. Increased inspiratory and end-expiratory pressure, for example, may cause parts of the lung that were previously collapsed to open, thereby influencing the exhaled VOC mixture [29]. Increased minute volume, while everything else is constant, also decreases the concentration of systemically produced VOCs, in accordance with end-tidal CO_2_ [30].

Several strengths of this study should also be noted. First, correcting for confounding factors was possible because we recorded data systematically in a database at one-minute intervals. Therefore, distilling the actual underlying eNose signal was possible. Second, the long observation periods per patient ensured that we measured a large number of possible sources of noise. Third, the sensor array that has been used was shown to discriminate between asthmatics, COPD patients, lung cancer patients and healthy controls [14]. Therefore, it could be argued that it is a valid tool to use in our investigation. There are also several weaknesses. Since metal oxide sensors that are used in eNoses are very cross-reactive, the analysis of this type of data relies on pattern recognition. Therefore, changes in individual VOC concentrations cannot be identified. Although this is not a limitation of the eNose as a measurement instrument, it does hamper us in identifying or quantifying individual VOCs in this study. While this is the first study to use cross-reactive sensors for continuous breath analysis in intubated and ventilated ICU-patients, PTR-MS has been studied in this setting [2]. Contrary to eNoses, PTR-MS allows for breath-by-breath measurements of the concentration of specific VOCs. However, the large size and high costs of PTR-MS machines currently limit the application as a bedside test. Breath-by-breath responsiveness was not obtained with the used sensor array. Therefore, it only facilitates monitoring of changes over hours, not minute by minute. However, biological phenomena like changing glucose levels do not require this high frequency of measurements. Finally, we cannot be certain that some of the signal of interest is influenced by our methods for noise reduction.

The described steps to remove noise inducing variables from eNose signals is a first step towards continuous breath monitoring in a clinical setting. In addition, continuous monitoring of biological markers allows for trend analysis, which is impossible with infrequent blood draws. Next to monitoring systemic markers, investigating molecular processes in the lung can possibly be simplified by monitoring exhaled breath. Currently, this is only possible by performing a bronchoalveolar lavage, which is considered to be very invasive. Diagnostic accuracy, however, should be studied and will be reported separately. Our findings help other researchers in their analysis, and interpretation of their results is beneficial to developers of eNose technology.

To conclude, changing humidity influenced eNose sensors and sensor signals were corrected. After outlier removal and smoothing, the signal was corrected for changes in ventilator settings. Pre-processing is the first step toward using continuous monitoring of exhaled breath.

## Figures and Tables

**Table 1:** Patient characteristics.

Parameter	Value	
Age in years, median (IQR ^a^)	67	62–75
Male gender, number (%)	9	39
Admission type ^b^, number (%)		
Acute medical	21	91
Planned surgery	2	9
APACHE II score ^c^, median (IQR)	22	19–28
SAPS II score ^d^, median (IQR)	52	42–65
ICU length of stay ^e^, days, median (IQR)	12	9–15
ICU mortality ^f^, number (%)	10	43
Measurement duration in hours, median (IQR)	51	41–65

^a^ IQR, interquartile range; ^b^ Admission type, the proportion of included patients admitted to the intensive care unit for either medical of surgical reasons; ^c^ APACHE II score, Acute Physiology and Chronic Health Evaluation II Score. This is a severity of illness score based on 12 routinely collected physiologic measurements in the ICU [26]; ^d^ SAPS II Score, Simplified Acute Physiology Score II. This is a severity of illness score based on 17 variables that is used to estimate of the risk of death without having to specify a primary diagnosis [27]; ^e^ ICU length of stay, the number of days a patient was admitted to the intensive care unit during the studied episode; ^f^ ICU mortality, proportion of patients dying on the intensive care unit during the studied episode.

**Table 2:** Fixed effects regression coefficients of relative humidity vs. sensors. Values indicate mean fixed effect regression coefficients ± their 95% confidence intervals.

Sensor	Relative Humidity
S1	−0.056 ± 0.002
S2	0.116 ± 0.001
S3	0.119 ± 0.001
S4	0.118 ± 0.001

**Table 3:** Fixed effects regression coefficients of ventilation parameters vs. sensors. Values indicate mean fixed effect regression coefficients ± their 95% confidence intervals.

Sensor	Minute Volume	etCO_2_	Tidal Volume	Inspiratory Pressure	Peak Pressure	PEEP
S1	−0.292 ± 0.043	0.028 ± 0.009	28.147 ± 1.362	0.164 ± 0.047	−0.093 ± 0.054	0.066 ± 0.019
S2	−0.177 ± 0.054	0.146 ± 0.012	39.485 ± 1.743	0.091 ± 0.060	0.218 ± 0.065	0.428 ± 0.024
S3	−0.500 ± 0.089	0.251 ± 0.020	60.292 ± 2.862	−0.728 ± 0.098	−0.628 ± 0.106	0.112 ± 0.041
S4	−0.219 ± 0.057	0.144 ± 0.013	41.749 ± 1.846	0.041 ± 0.064	0.103 ± 0.079	0.396 ± 0.026

**Table 4:** Fixed effects regression coefficients of ventilation parameters vs. sensors after correction for ventilator settings and ETCO_2_ Values indicate mean fixed effect regression coefficients ± their 95% confidence intervals.

Sensor	Minute Volume	etCO_2_	Tidal Volume	Inspiratory Pressure	Peak Pressure	PEEP
S1	0.000 ± 0.015	0.000 ± 0.004	0.014 ± 0.508	−0.001 ± 0.018	0.000 ± 0.018	0.000 ± 0.007
S2	−0.001 ± 0.015	0.000 ± 0.004	0.023 ± 0.508	−0.001 ± 0.018	0.000 ± 0.018	0.000 ± 0.007
S3	−0.001 ± 0.015	0.000 ± 0.004	0.014 ± 0.508	−0.001 ± 0.018	0.000 ± 0.018	0.000 ± 0.007
S4	−0.001 ± 0.015	0.000 ± 0.004	0.023 ± 0.508	−0.001 ± 0.018	0.000 ± 0.018	0.000 ± 0.007

## Supplementary Materials

The following are available online at http://www.mdpi.com/1424-8220/16/8/1337/s1, Figures for each individual patient.
