# Supplementary material for: Factors Influencing Continuous Breath Signal in Intubated and Mechanically-Ventilated Intensive Care Unit Patients Measured by an Electronic Nose
Source: Sensors (Basel). 2016 Aug 22;16(8):1337. doi: 10.3390/s16081337 (PMC5017501; doi:10.3390/s16081337)
Supplement: Supplementary file 1 [file sensors-16-01337-s001.pdf]

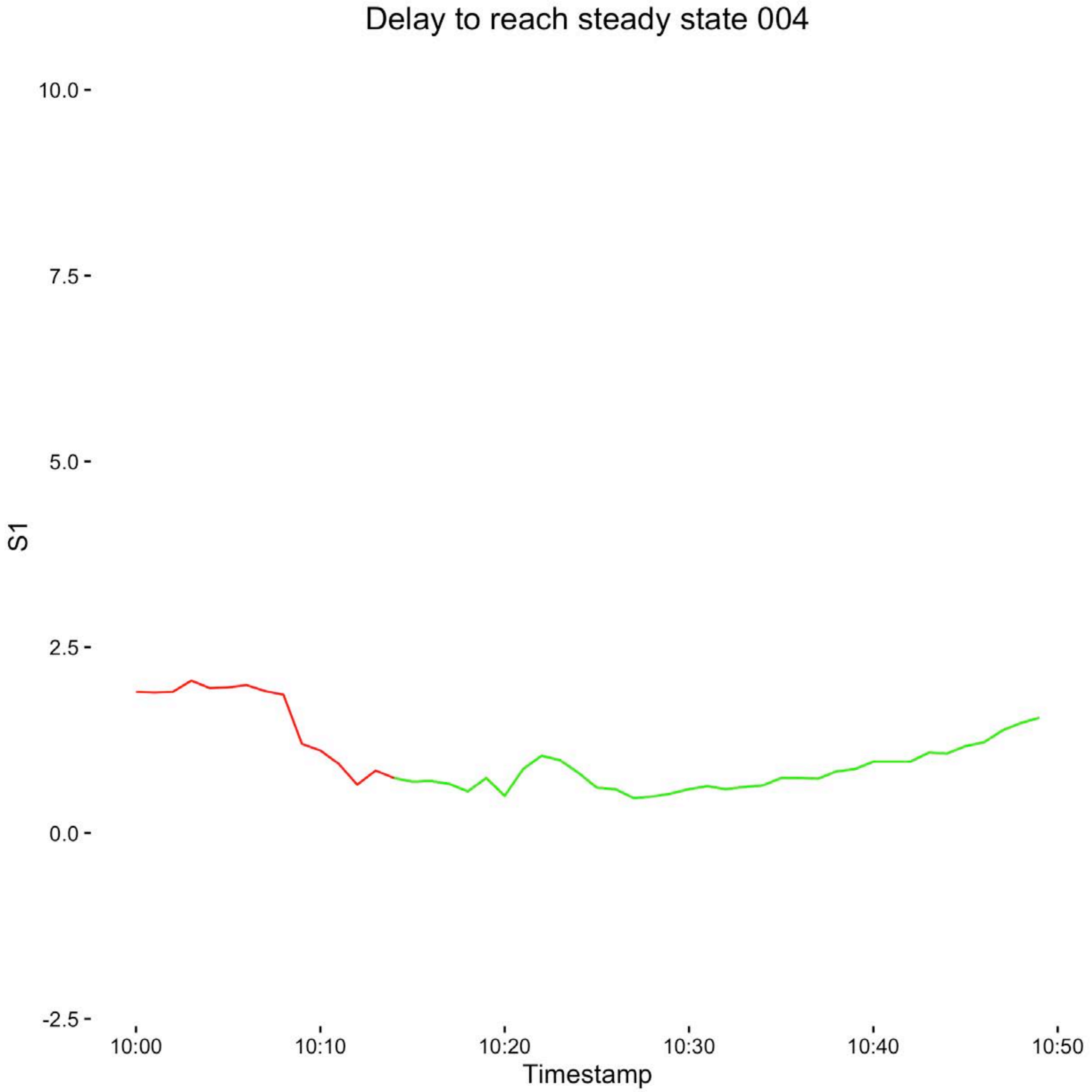
Delay to reach steady state 004
10.0
7.5
5.0
2.5
0.0
-2.5
S1
10:00
10:10
10:20
10:30
10:40
10:50
Timestamp

Delay to reach steady state 005

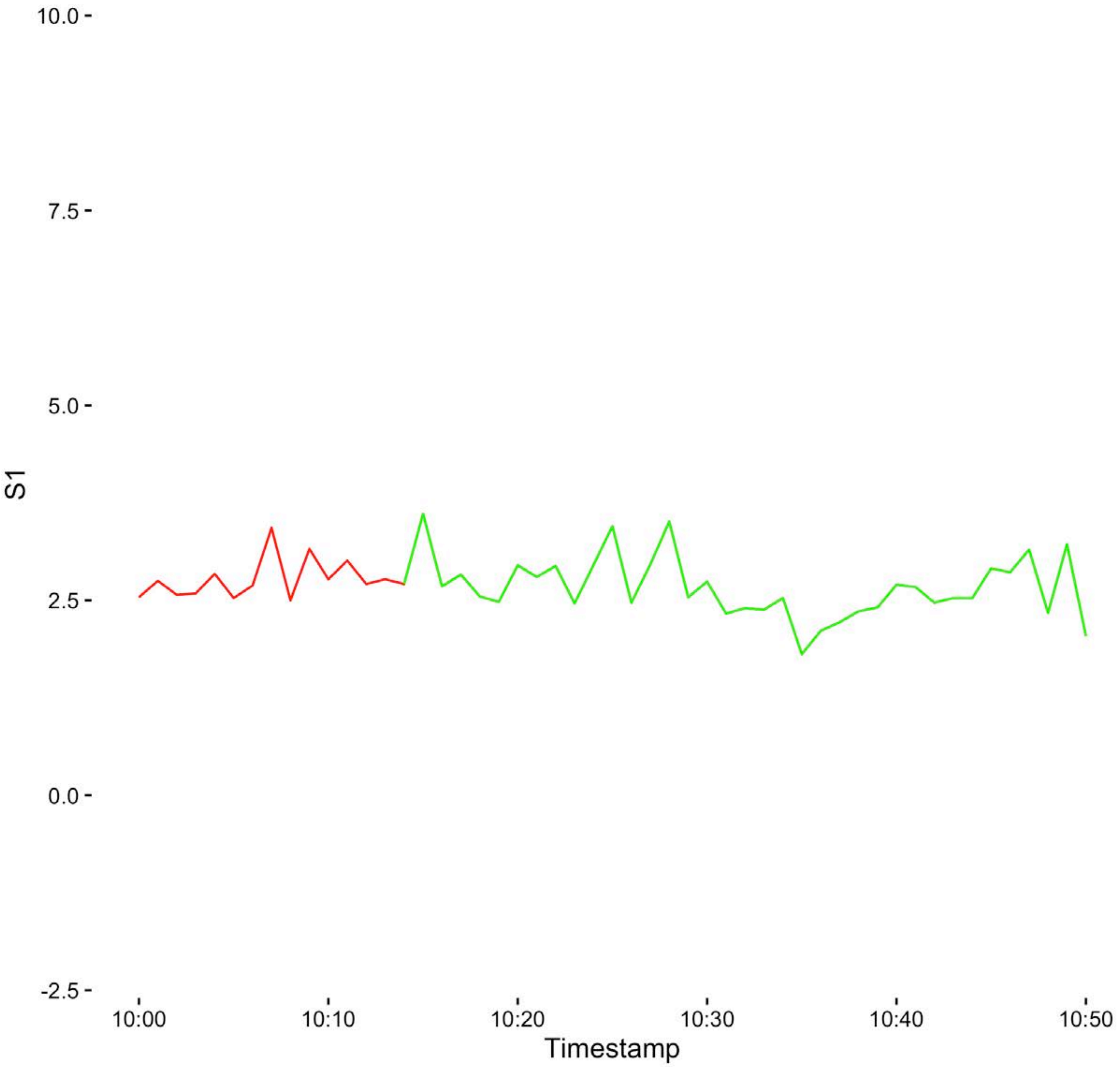

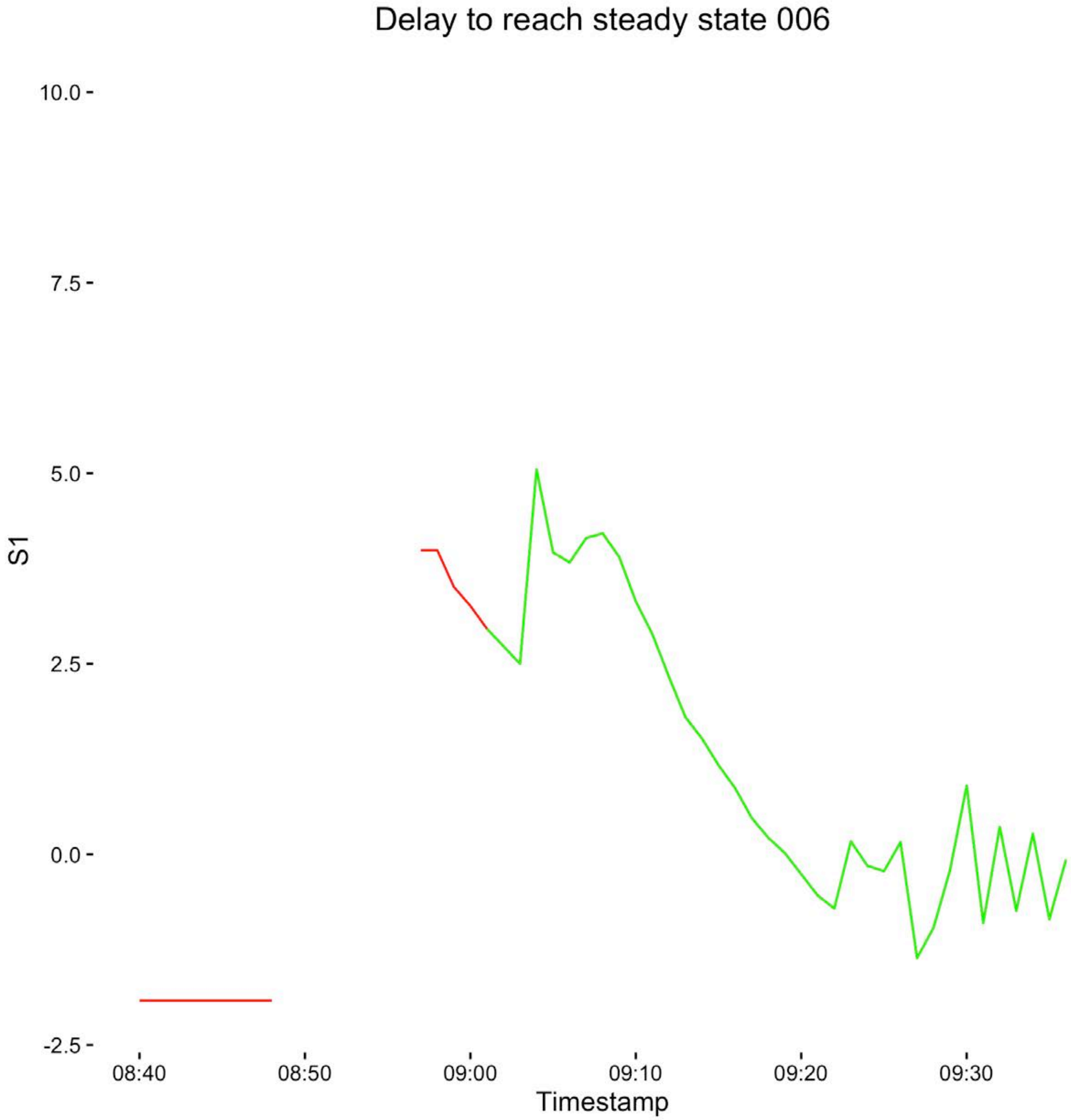

Delay to reach steady state 006
10.0
7.5
5.0
2.5
0.0
-2.5
S1
08:40
08:50
09:00
09:10
09:20
09:30
Timestamp

Delay to reach steady state 007

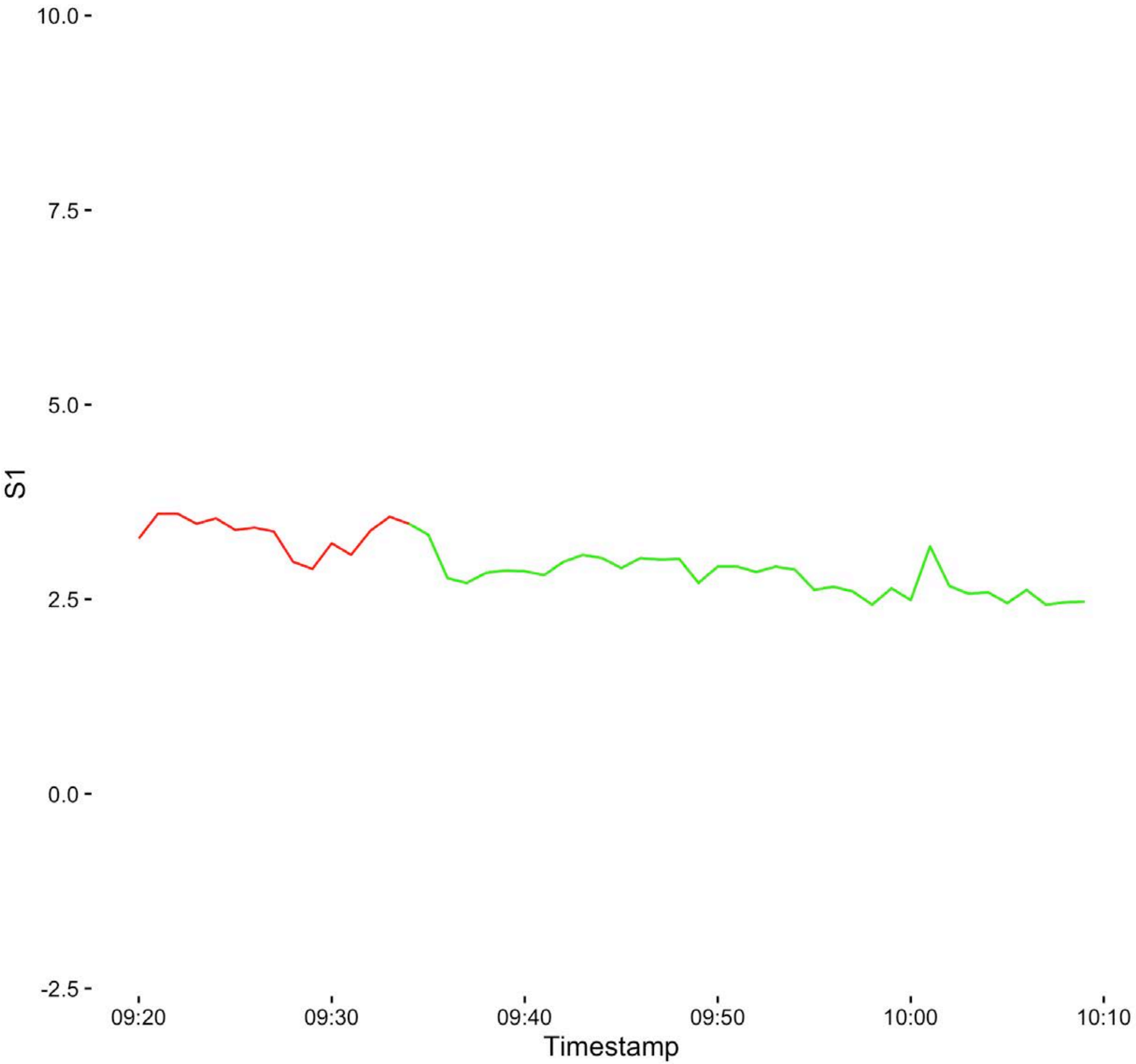

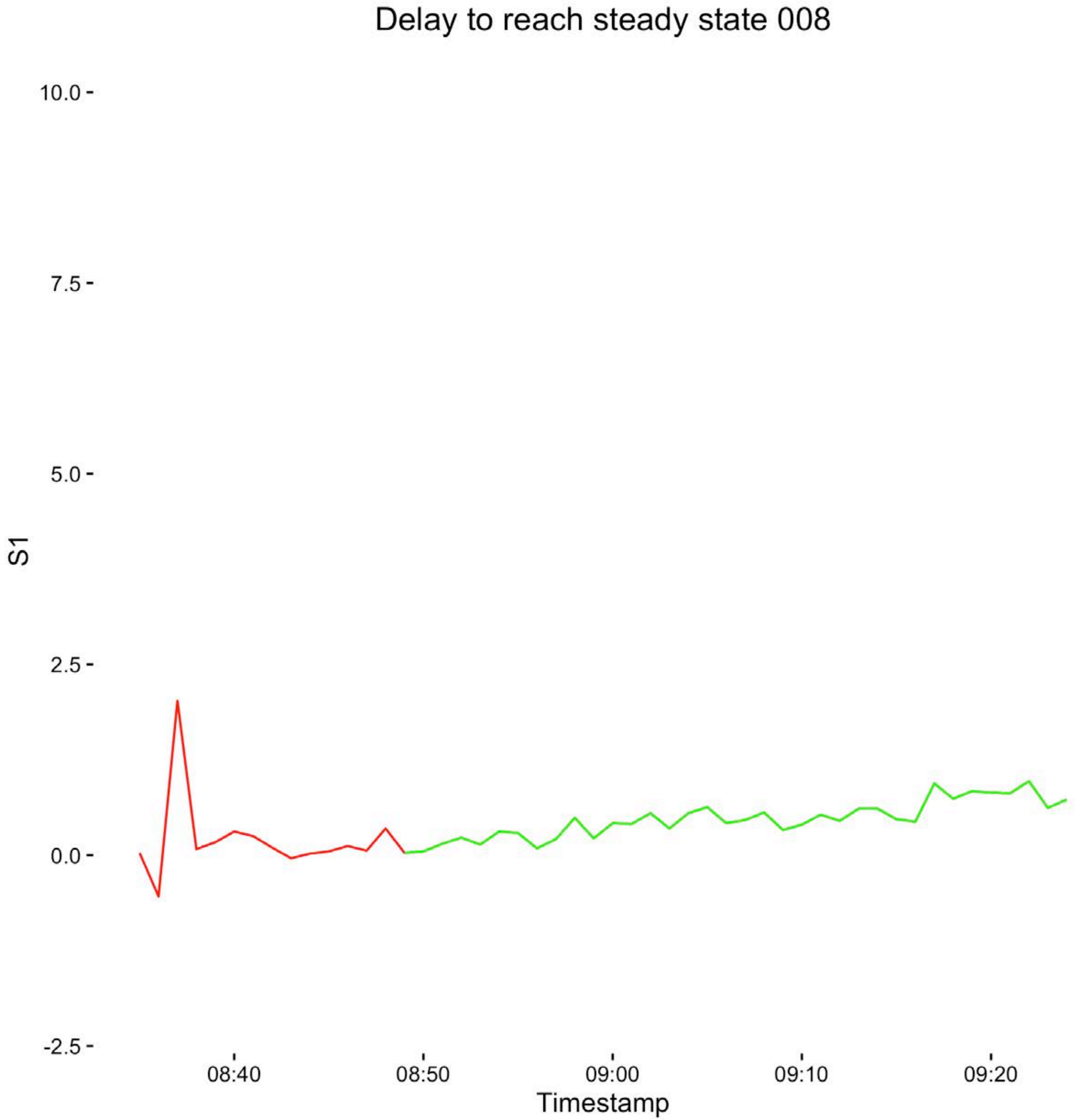

Delay to reach steady state 008
10.0
7.5
5.0
2.5
0.0
-2.5
S1
08:40
08:50
09:00
09:10
09:20
Timestamp

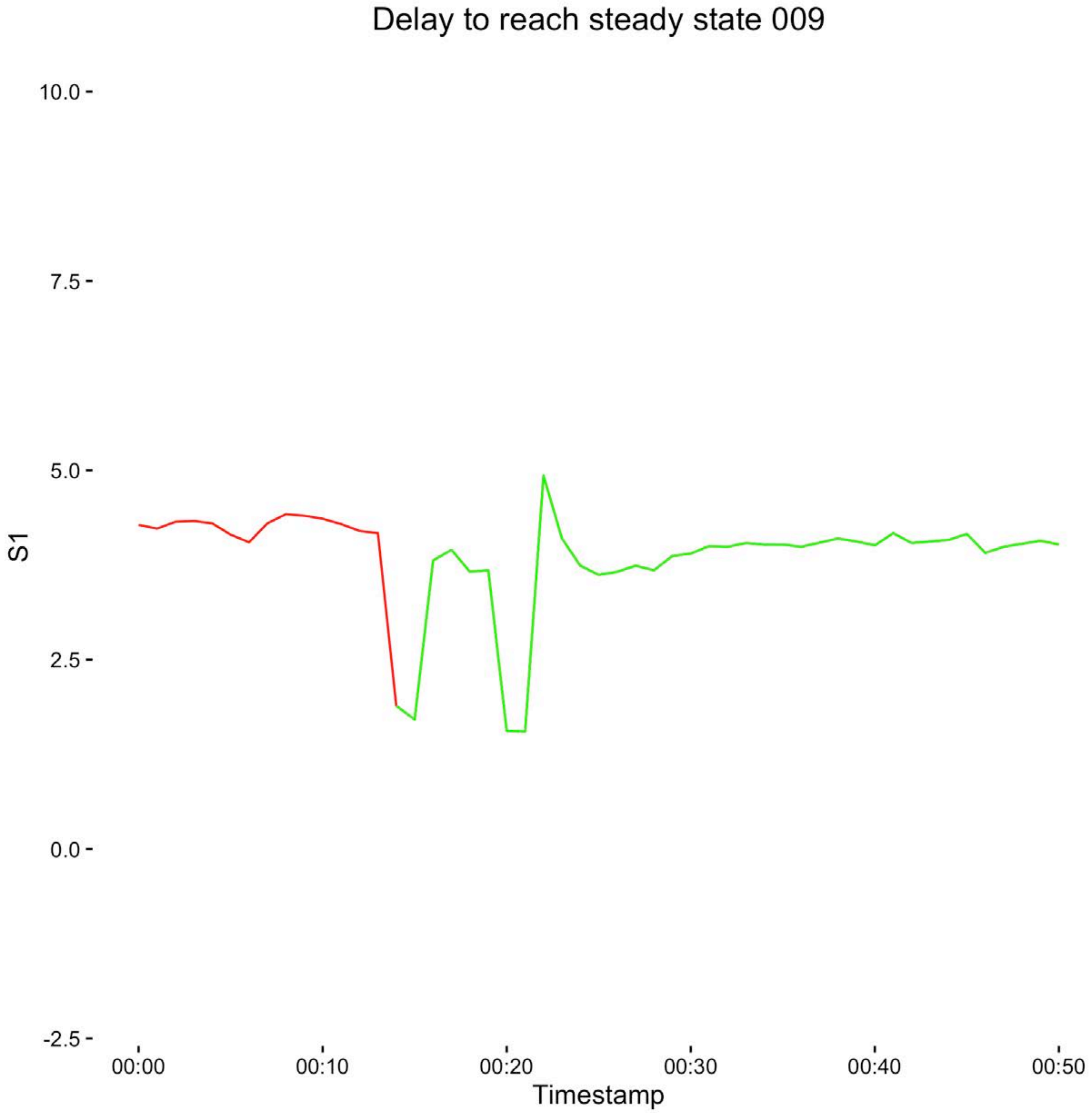

Delay to reach steady state 009
10.0
7.5
5.0
2.5
0.0
-2.5
S1
00:00
00:10
00:20
00:30
00:40
00:50
Timestamp

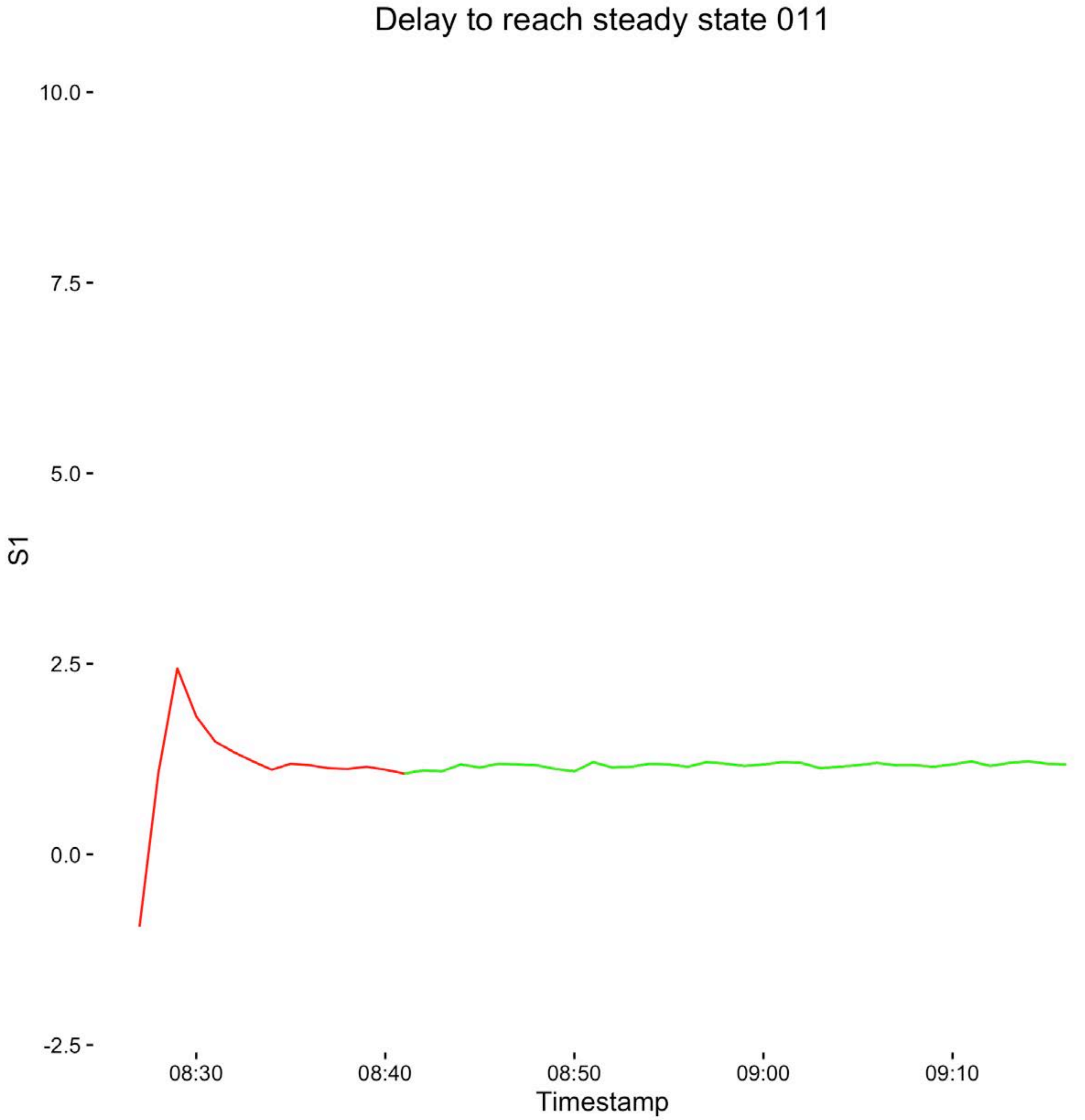
Delay to reach steady state 011
10.0
7.5
5.0
2.5
0.0
-2.5
S1
08:30
08:40
08:50
09:00
09:10
Timestamp

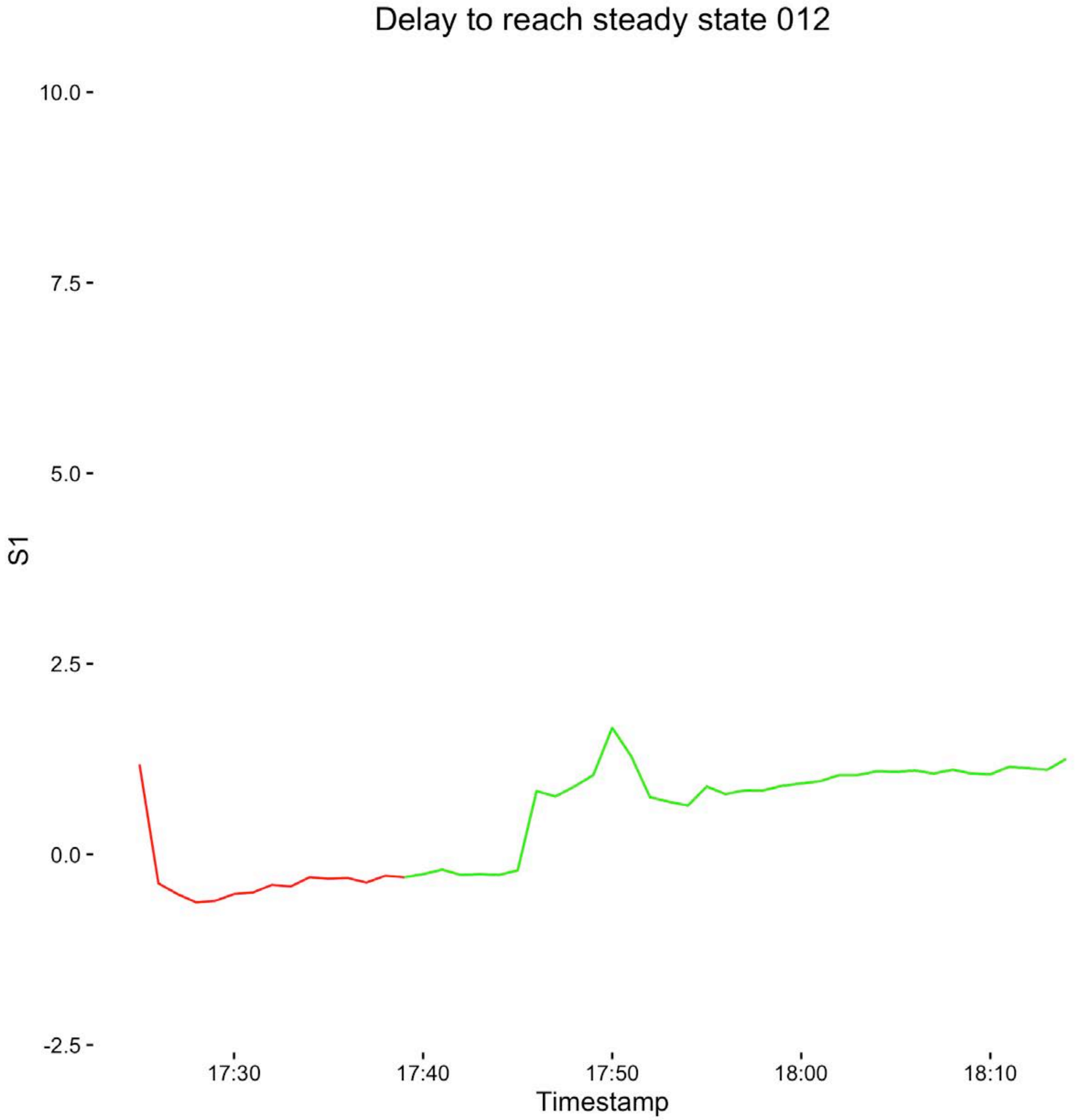

Delay to reach steady state 012
10.0
7.5
5.0
2.5
0.0
-2.5
S1
17:30
17:40
17:50
18:00
18:10
Timestamp

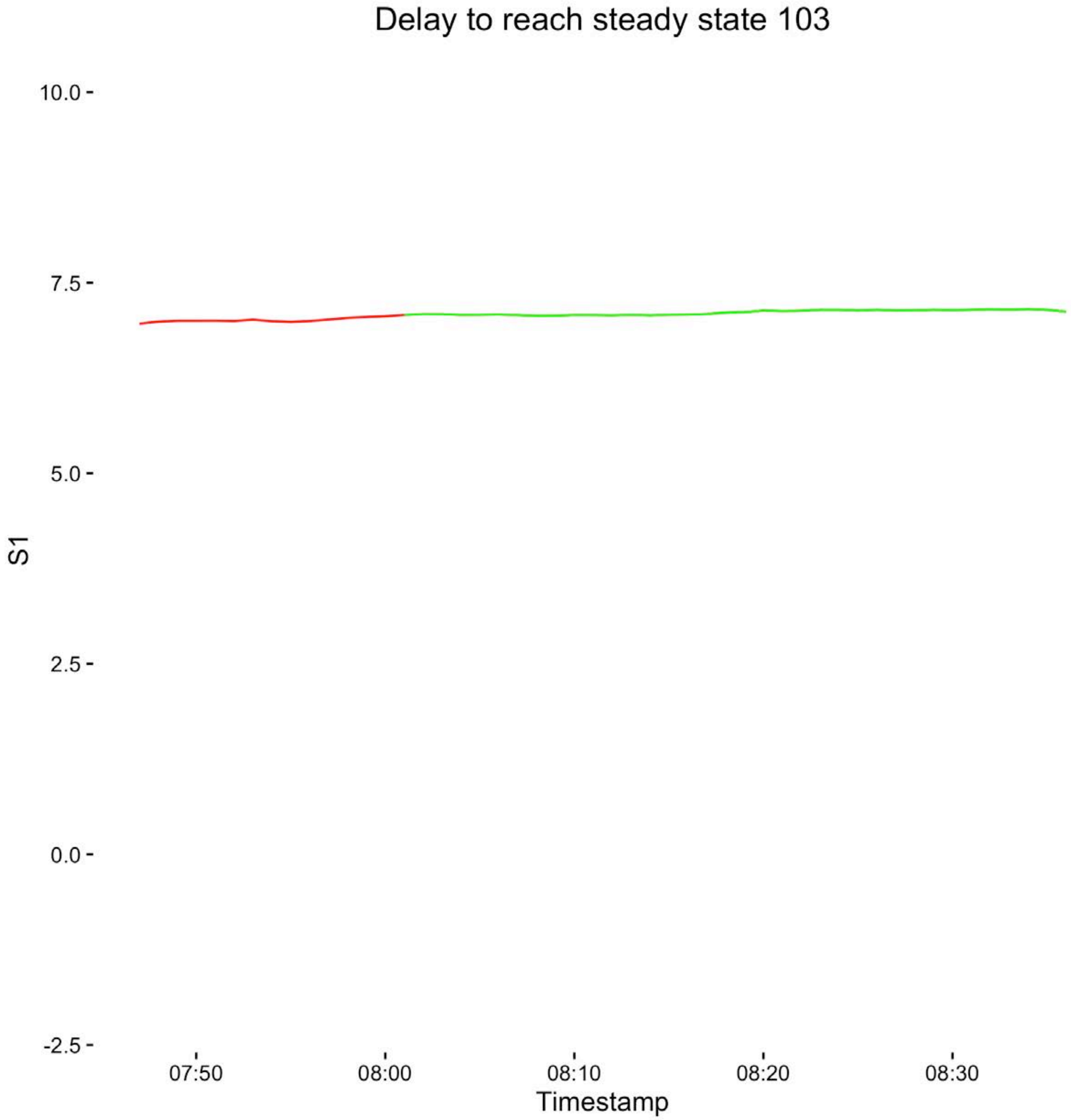

Delay to reach steady state 103
10.0
7.5
5.0
2.5
0.0
-2.5
S1
07:50
08:00
08:10
08:20
08:30
Timestamp

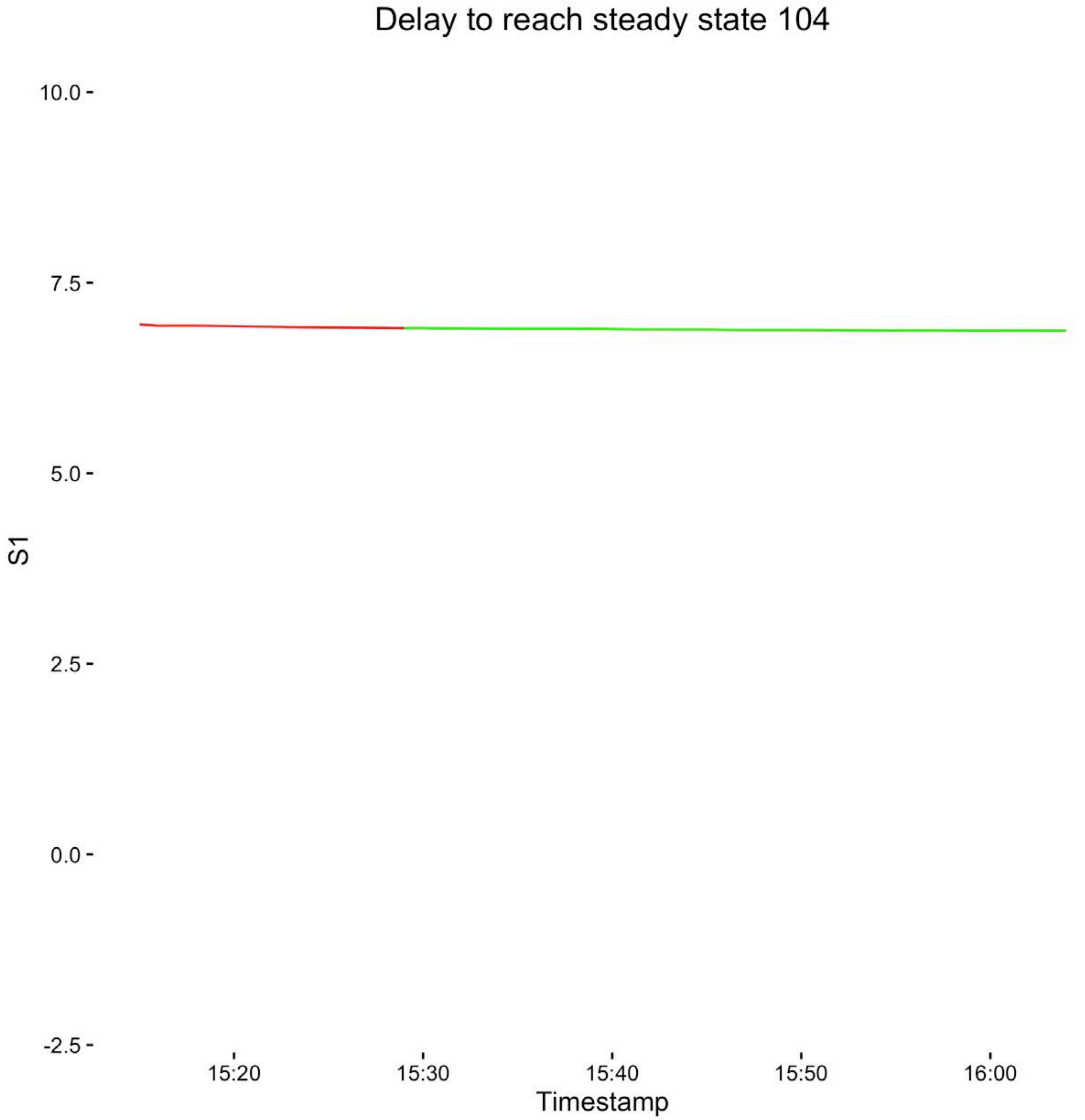

Delay to reach steady state 104
10.0
7.5
5.0
2.5
0.0
-2.5
S1
15:20
15:30
15:40
15:50
16:00
Timestamp

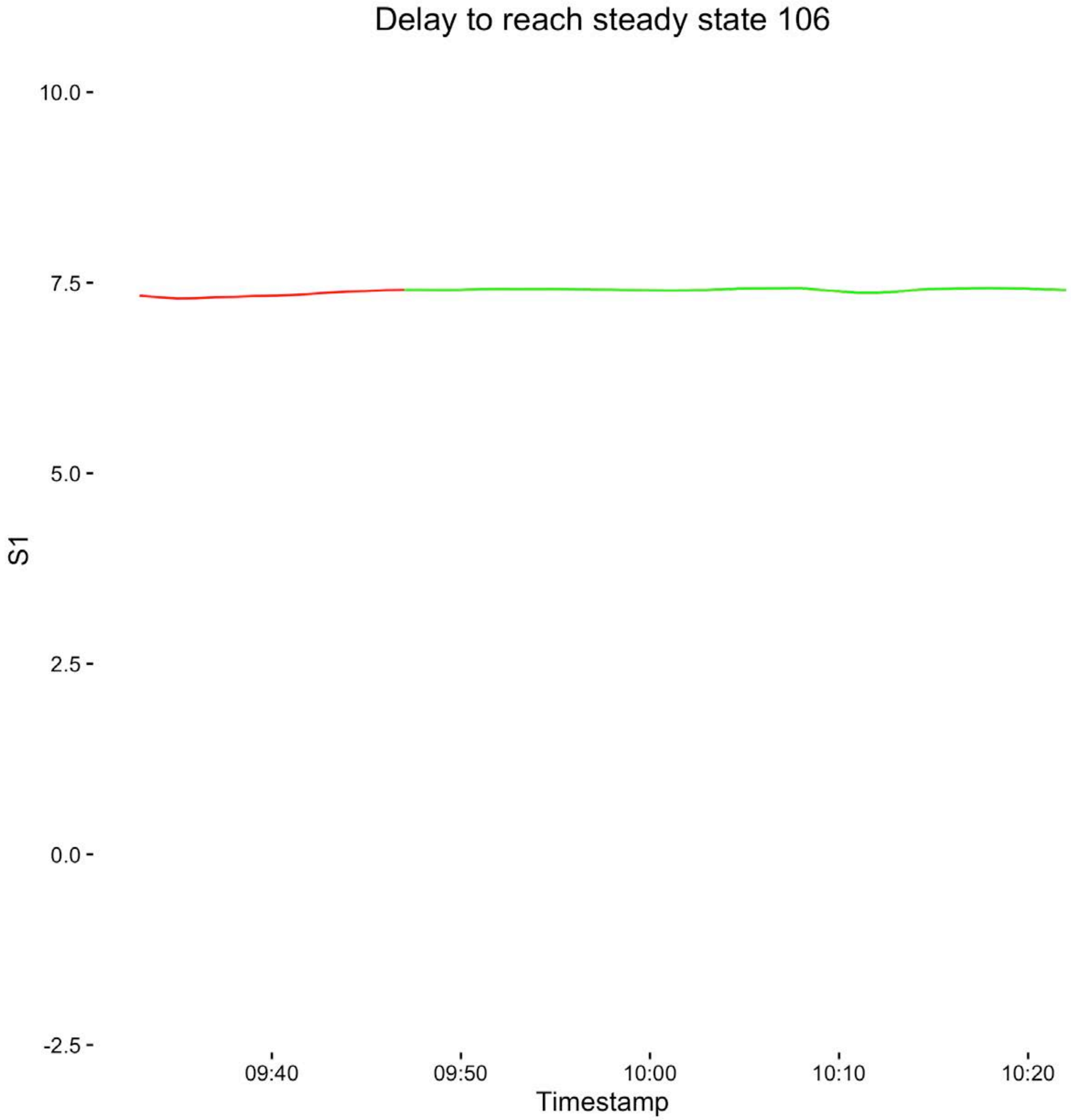

Delay to reach steady state 106
10.0
7.5
5.0
2.5
0.0
-2.5
S1
09:40
09:50
10:00
10:10
10:20
Timestamp

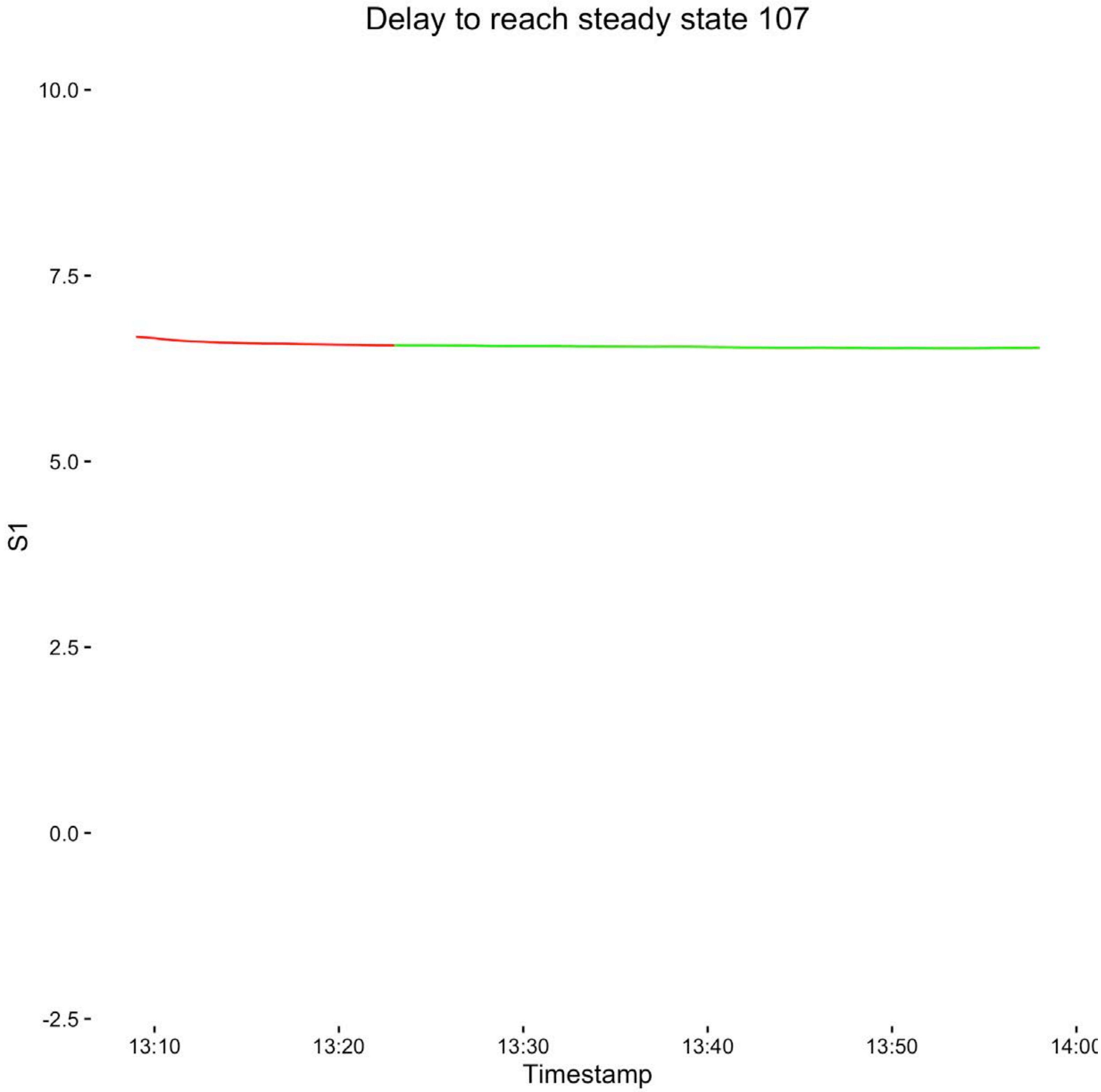
Delay to reach steady state 107
10.0
7.5
5.0
2.5
0.0
-2.5
S1
13:10
13:20
13:30
13:40
13:50
14:0
Timestamp

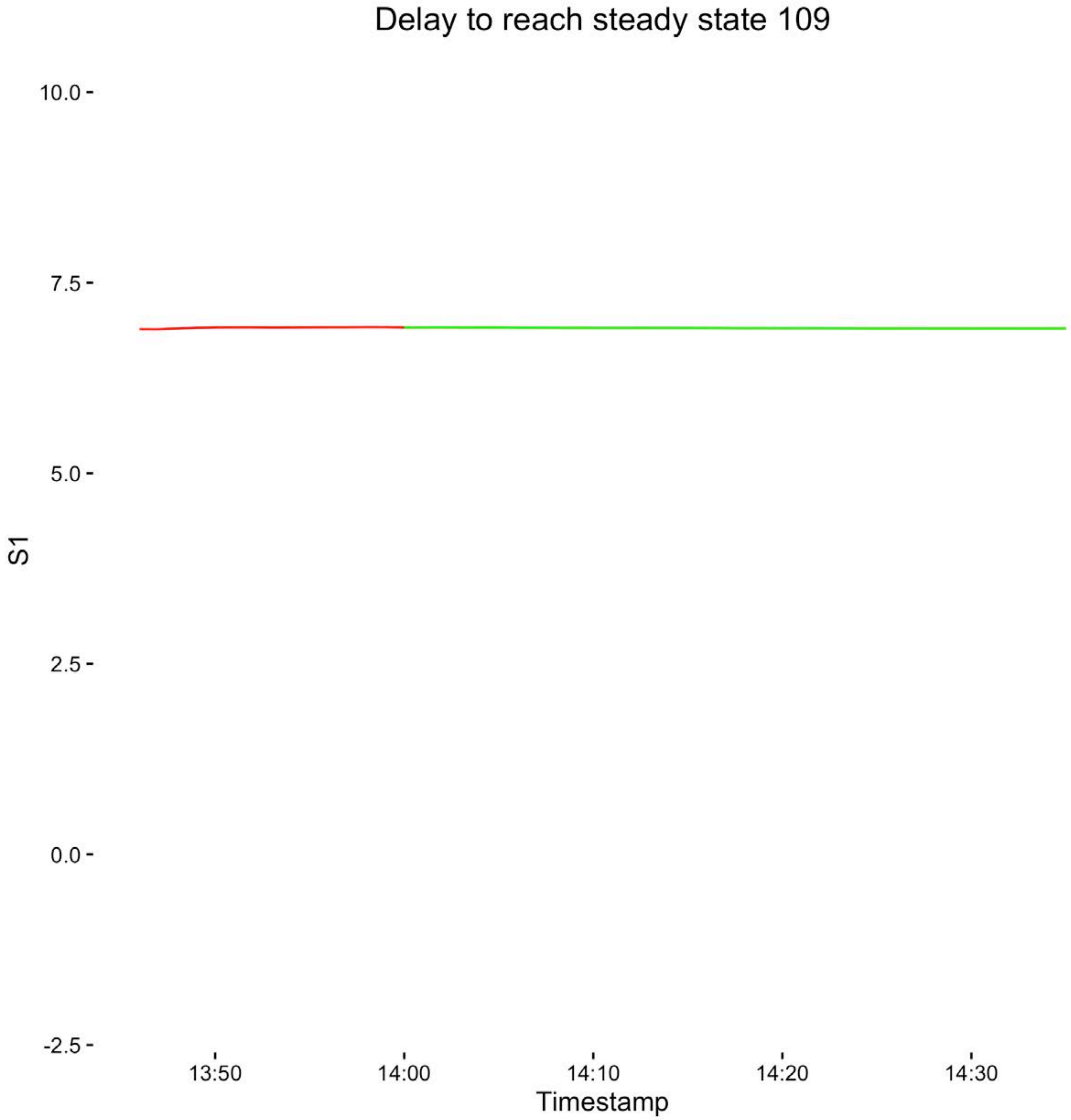
Delay to reach steady state 109
10.0
7.5
5.0
2.5
0.0
-2.5
S1
13:50
14:00
14:10
14:20
14:30
Timestamp

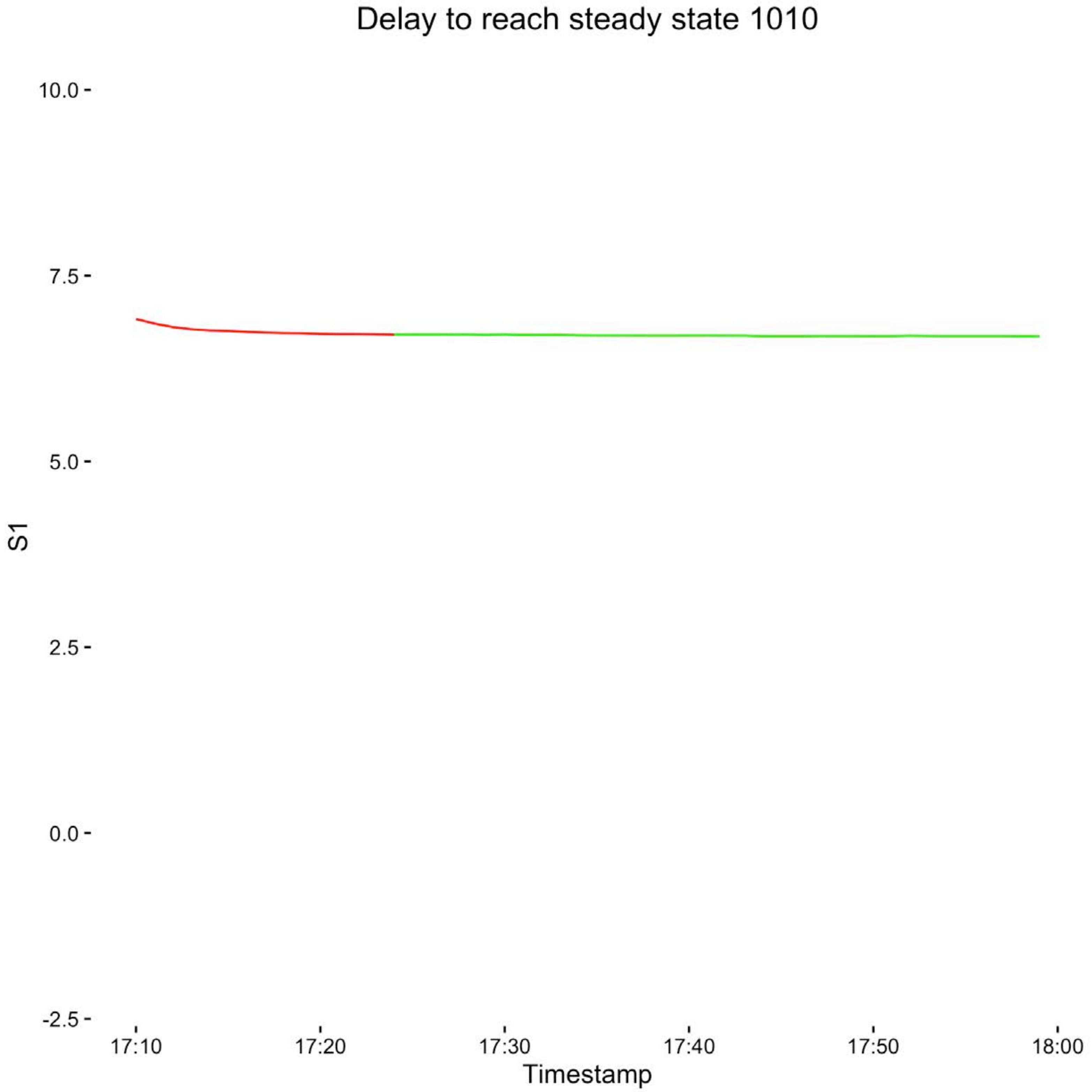
Delay to reach steady state 1010
10.0
7.5
5.0
2.5
0.0
-2.5
S1
17:10
17:20
17:30
17:40
17:50
18:00
Timestamp

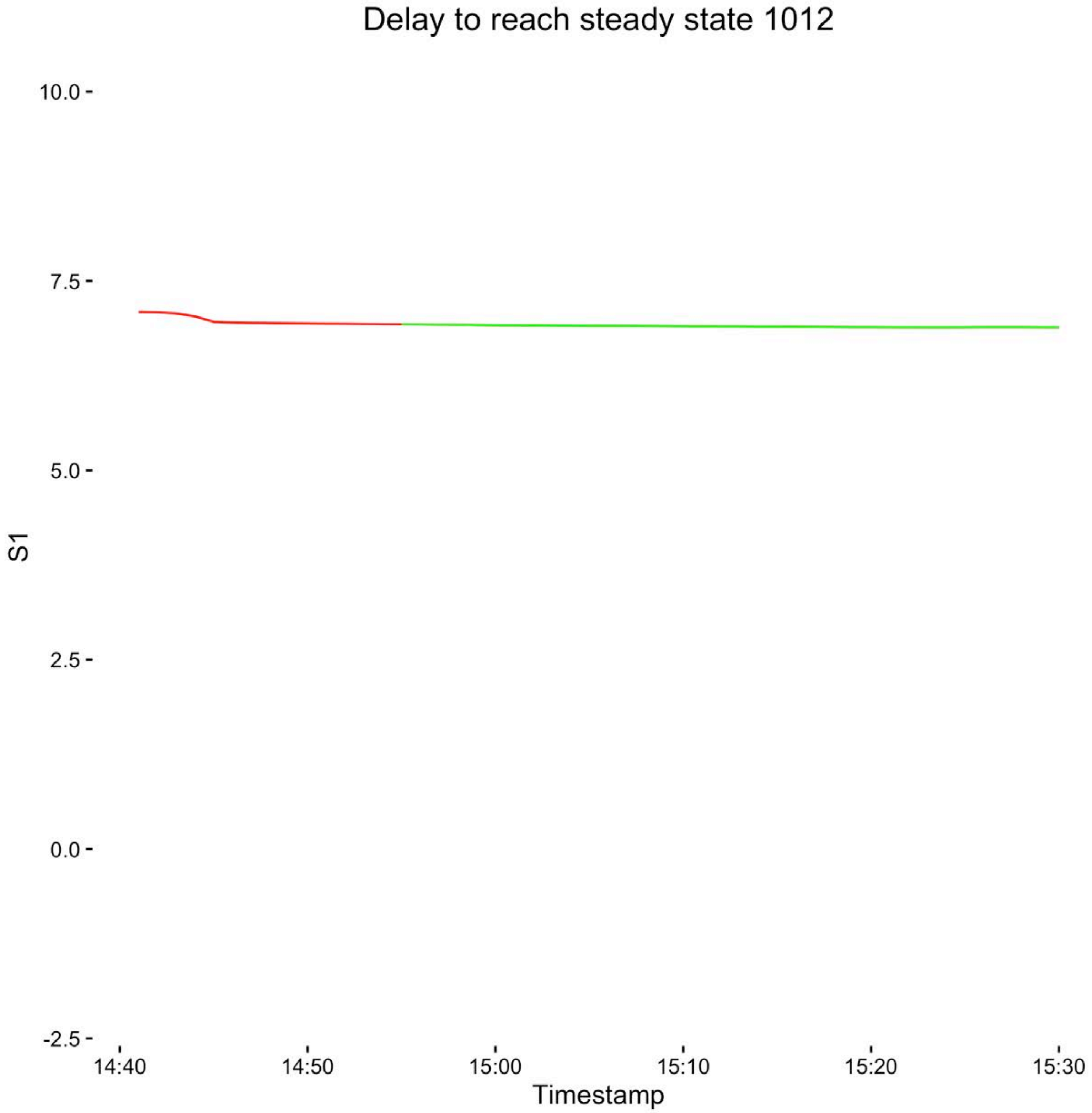

Delay to reach steady state 1012
10.0
7.5
5.0
2.5
0.0
-2.5
S1
14:40
14:50
15:00
15:10
15:20
15:30
Timestamp

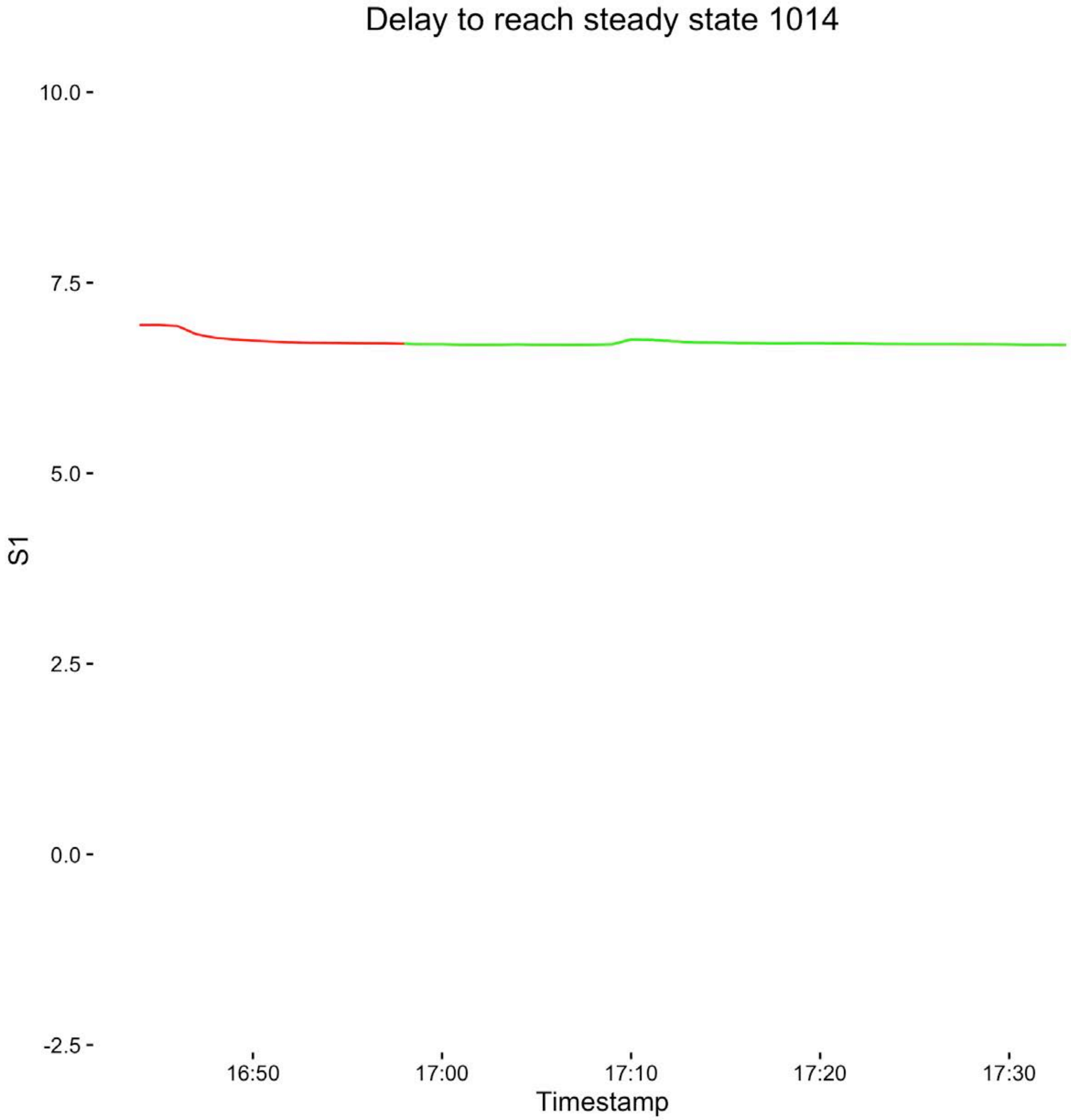
Delay to reach steady state 1014
10.0
7.5
5.0
2.5
0.0
-2.5
S1
16:50
17:00
17:10
17:20
17:30
Timestamp

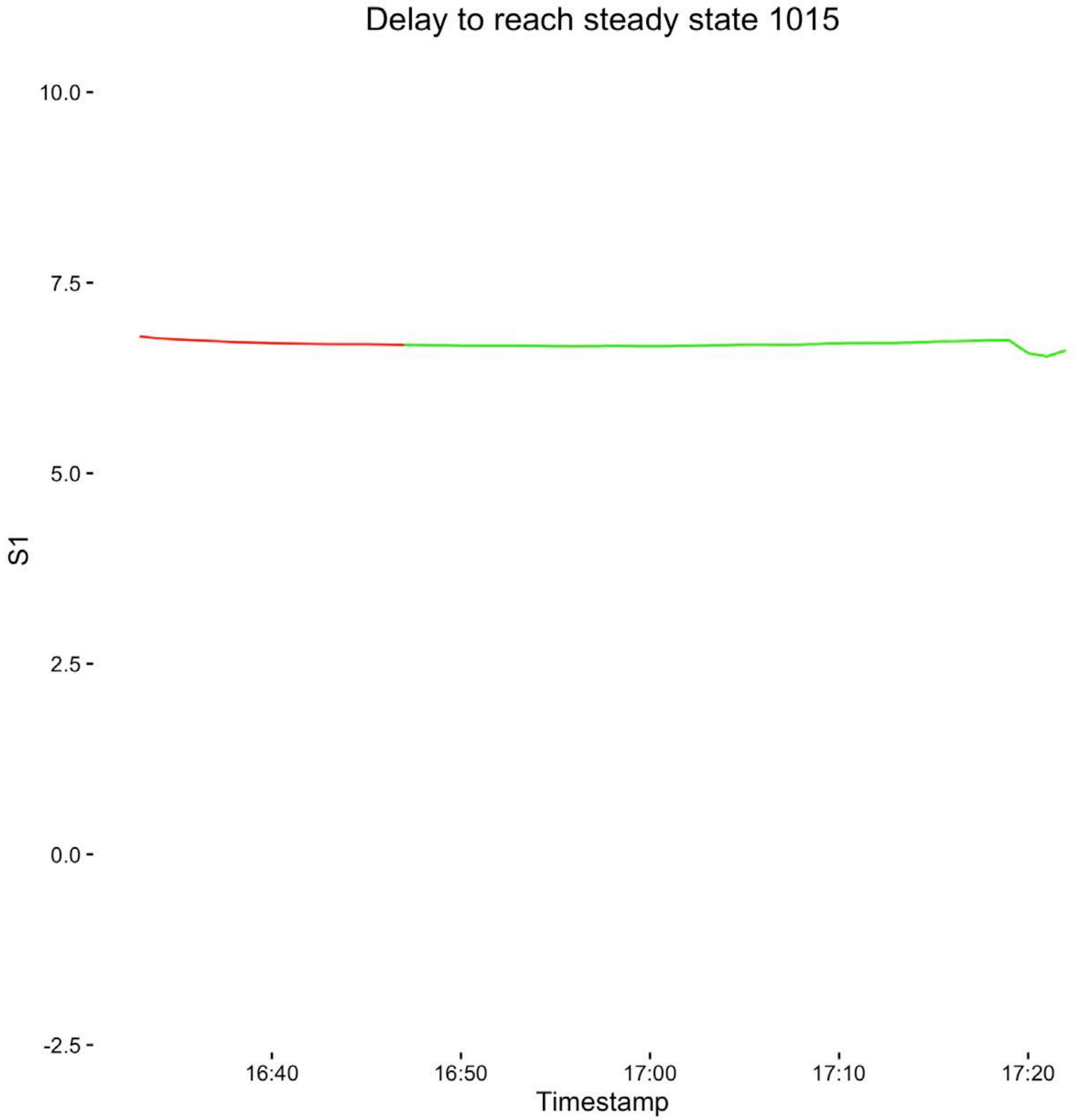
Delay to reach steady state 1015
10.0
7.5
5.0
2.5
0.0
-2.5
S1
16:40
16:50
17:00
17:10
17:20
Timestamp

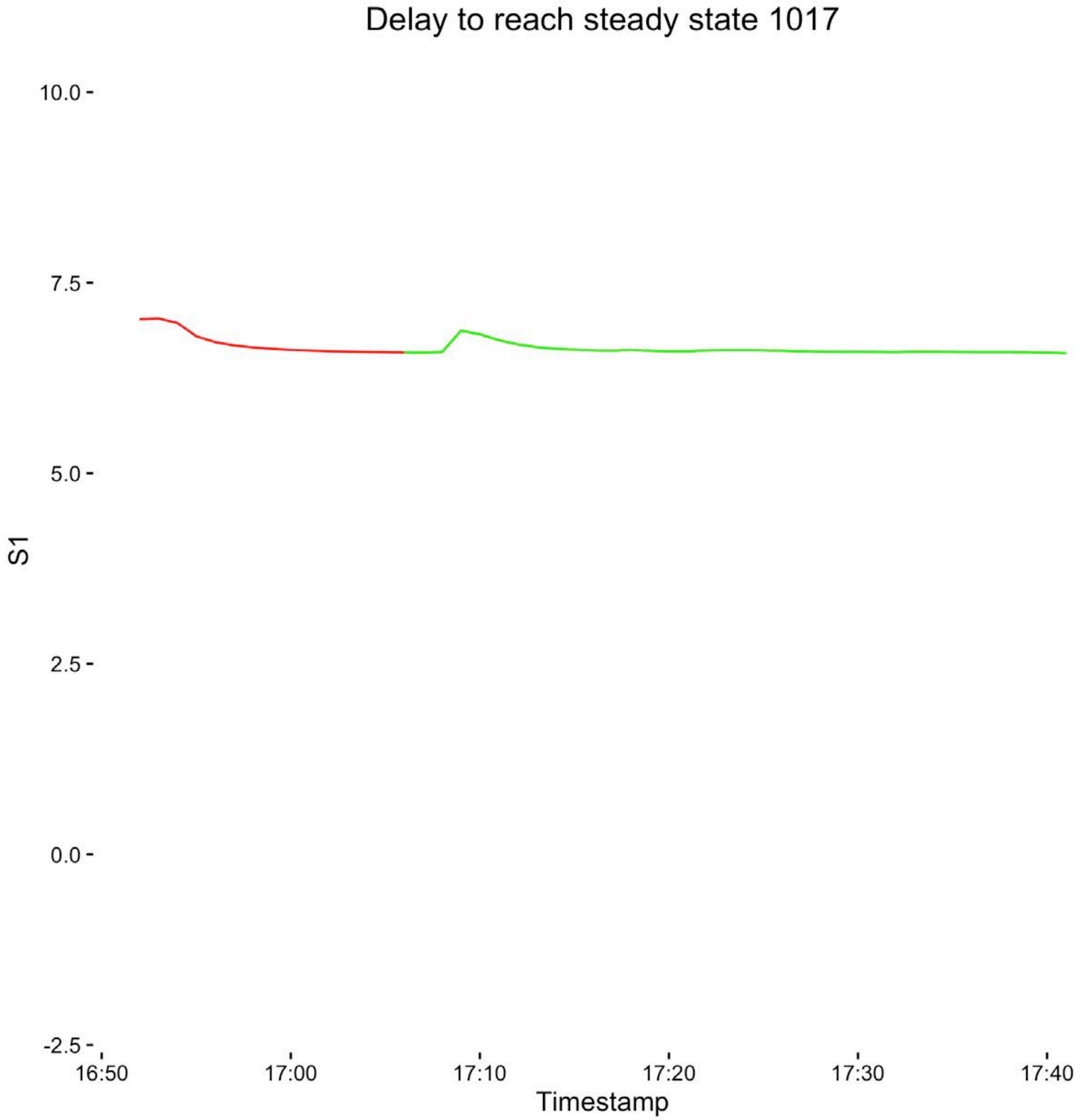

Delay to reach steady state 1017
10.0
7.5
5.0
2.5
0.0
-2.5
S1
16:50
17:00
17:10
17:20
17:30
17:40
Timestamp

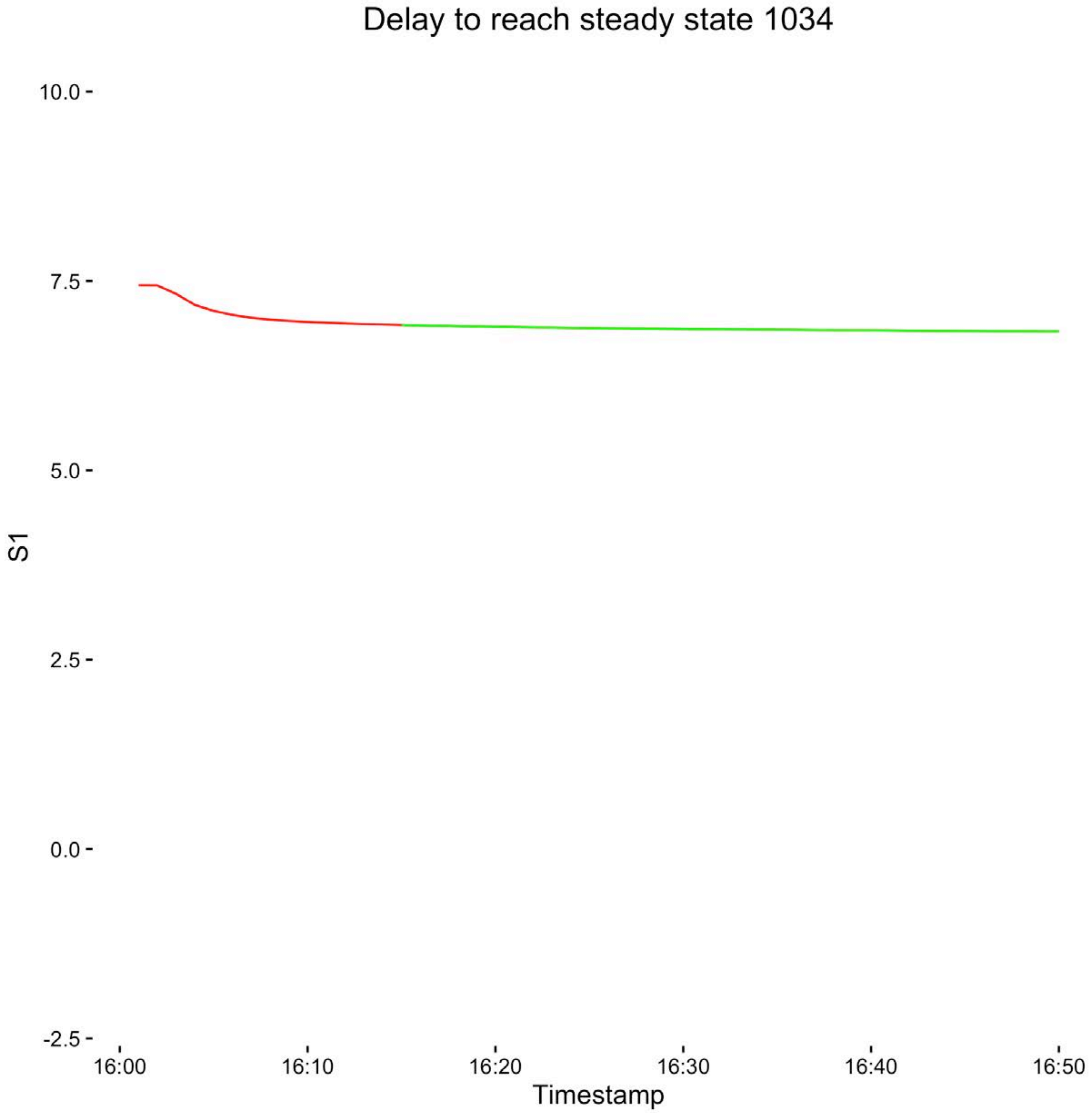

Delay to reach steady state 1034
10.0
7.5
5.0
2.5
0.0
-2.5
S1
16:00
16:10
16:20
16:30
16:40
16:50
Timestamp

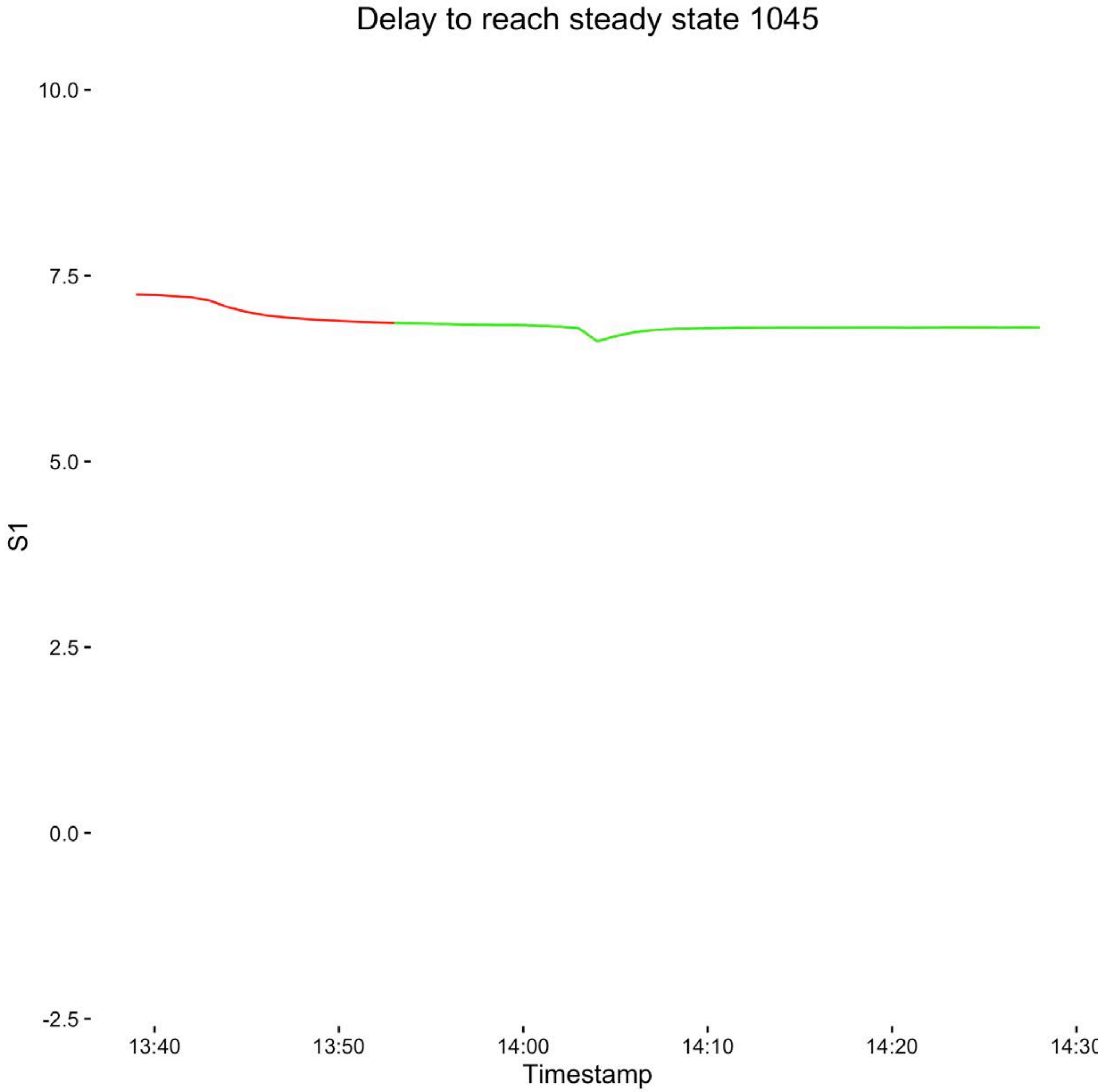
Delay to reach steady state 1045
10.0
7.5
5.0
2.5
0.0
-2.5
S1
13:40
13:50
14:00
14:10
14:20
14:30
Timestamp

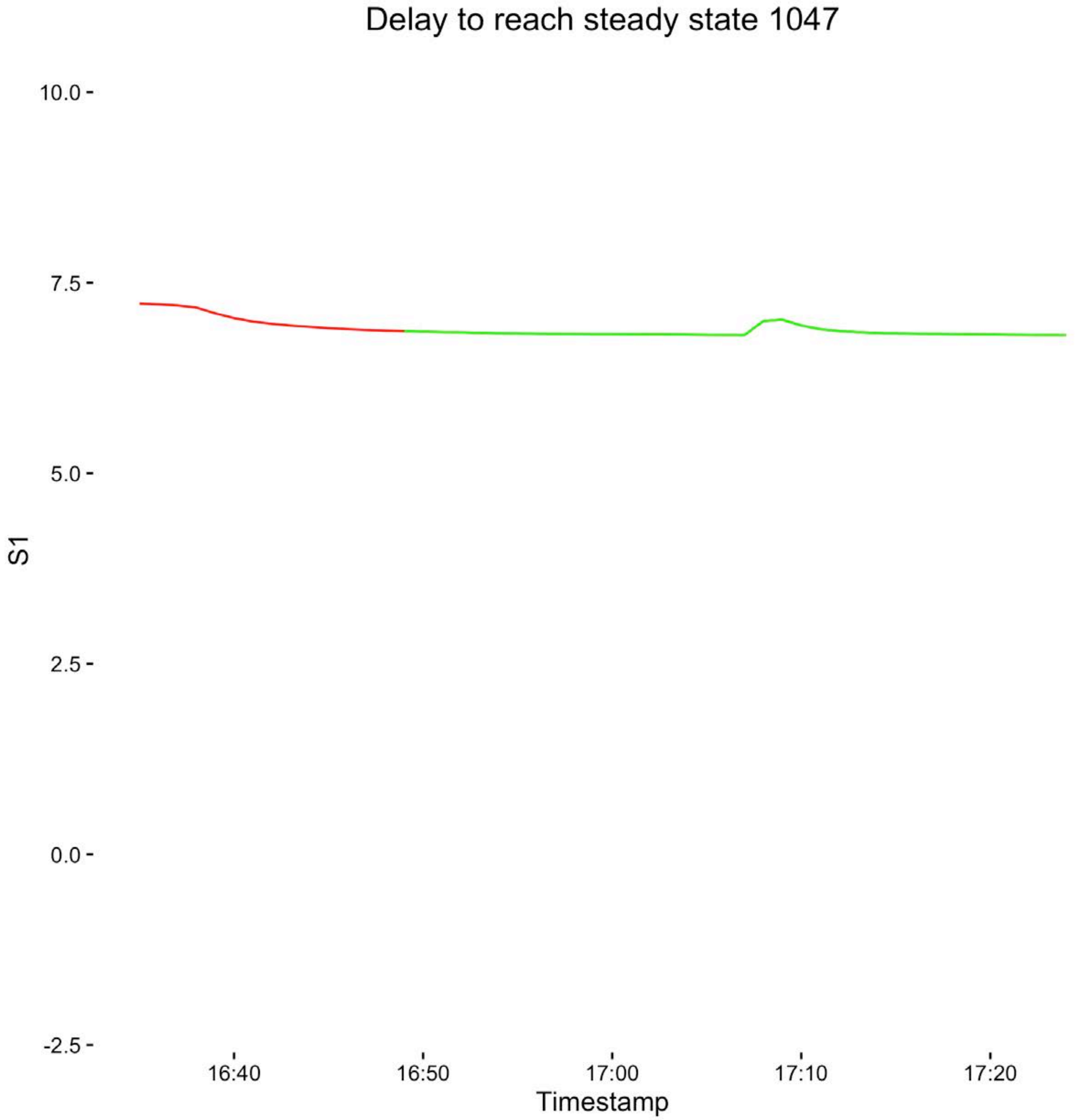

Delay to reach steady state 1047
10.0
7.5
5.0
2.5
0.0
-2.5
S1
16:40
16:50
17:00
17:10
17:20
Timestamp

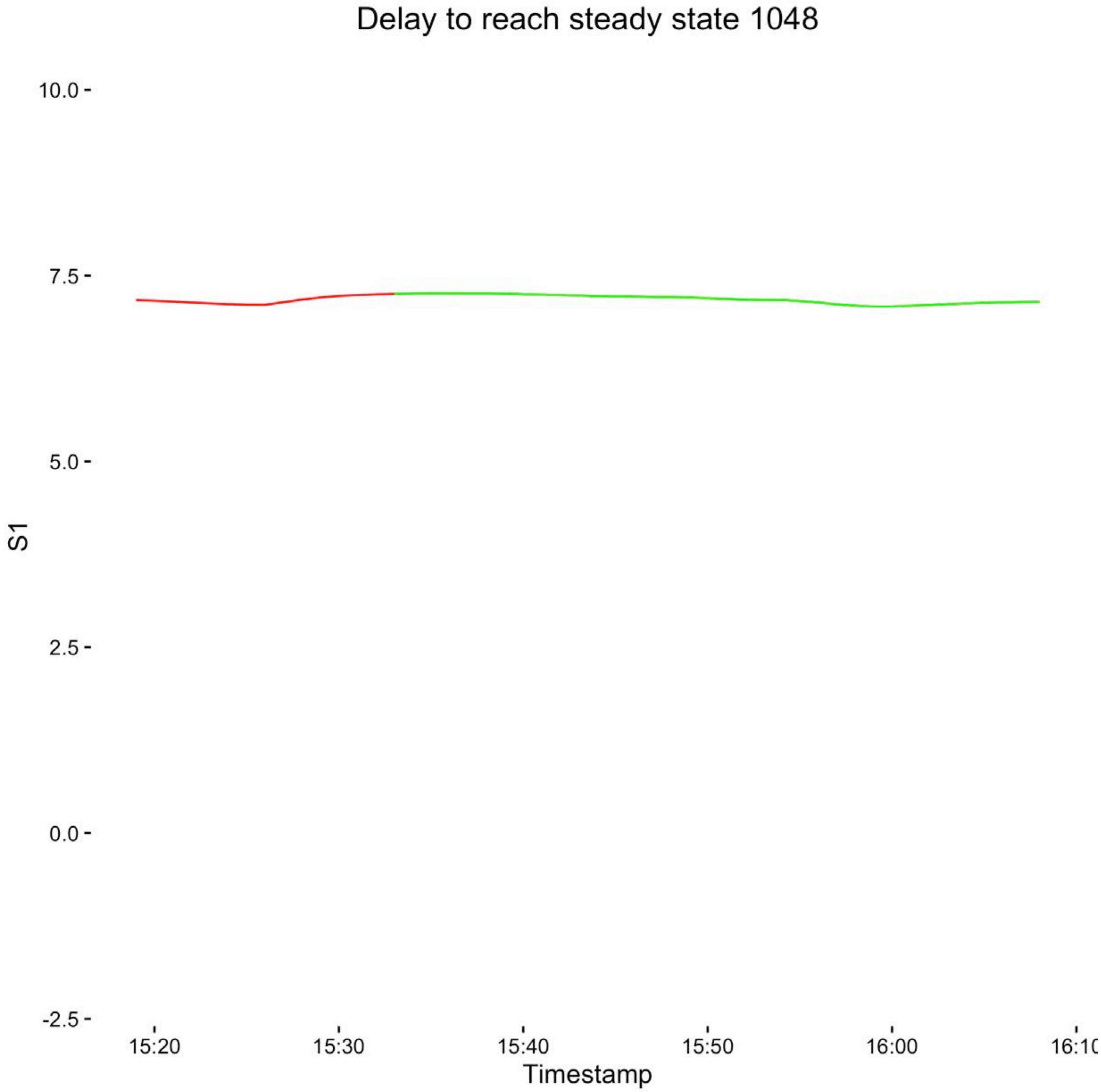
Delay to reach steady state 1048
10.0
7.5
5.0
2.5
0.0
-2.5
S1
15:20
15:30
15:40
15:50
16:00
Timestamp

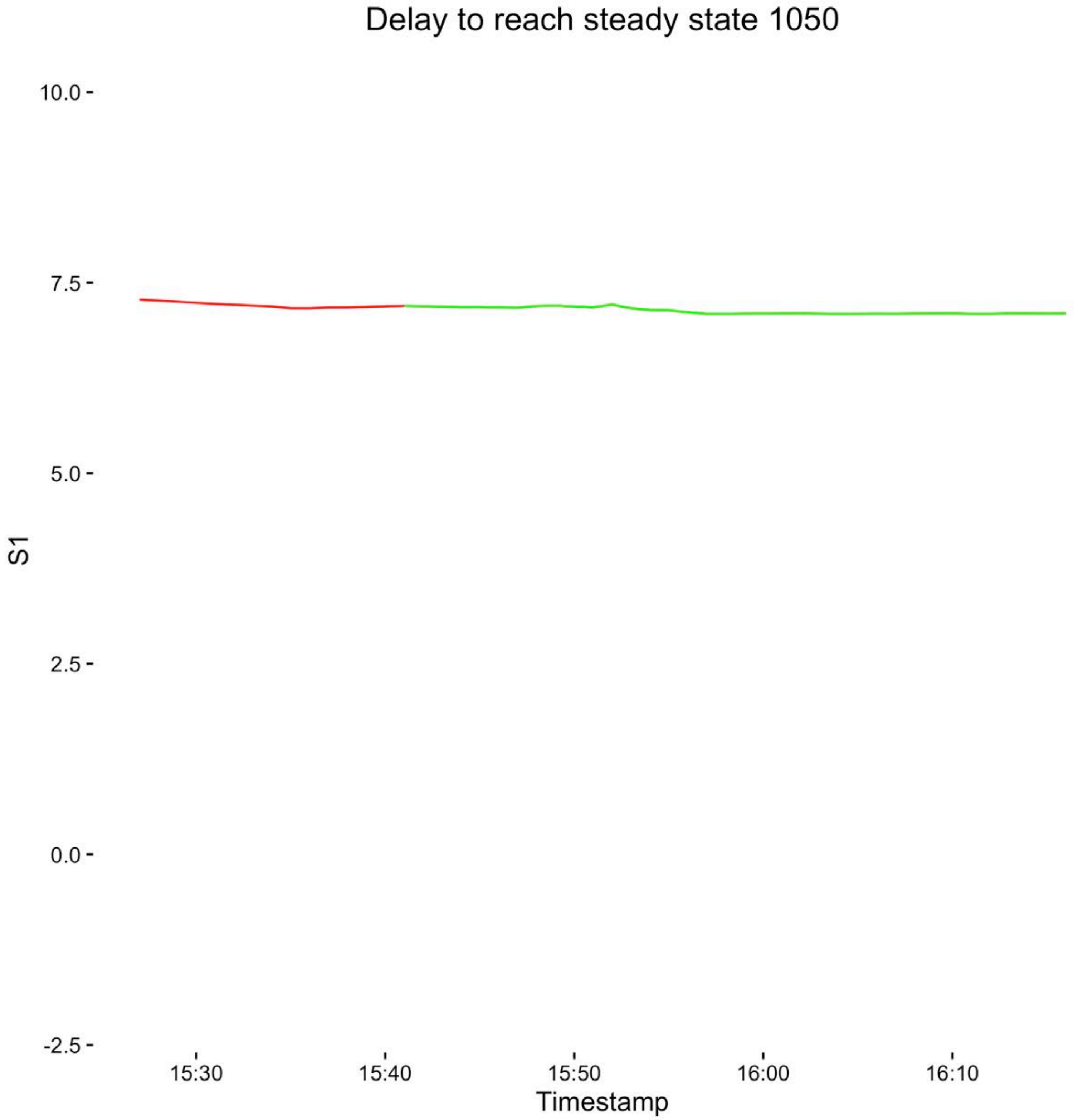
Delay to reach steady state 1050
10.0
7.5
5.0
2.5
0.0
-2.5
S1
15:30
15:40
15:50
16:00
16:10
Timestamp

correlation Sensors ~ Humidity 4

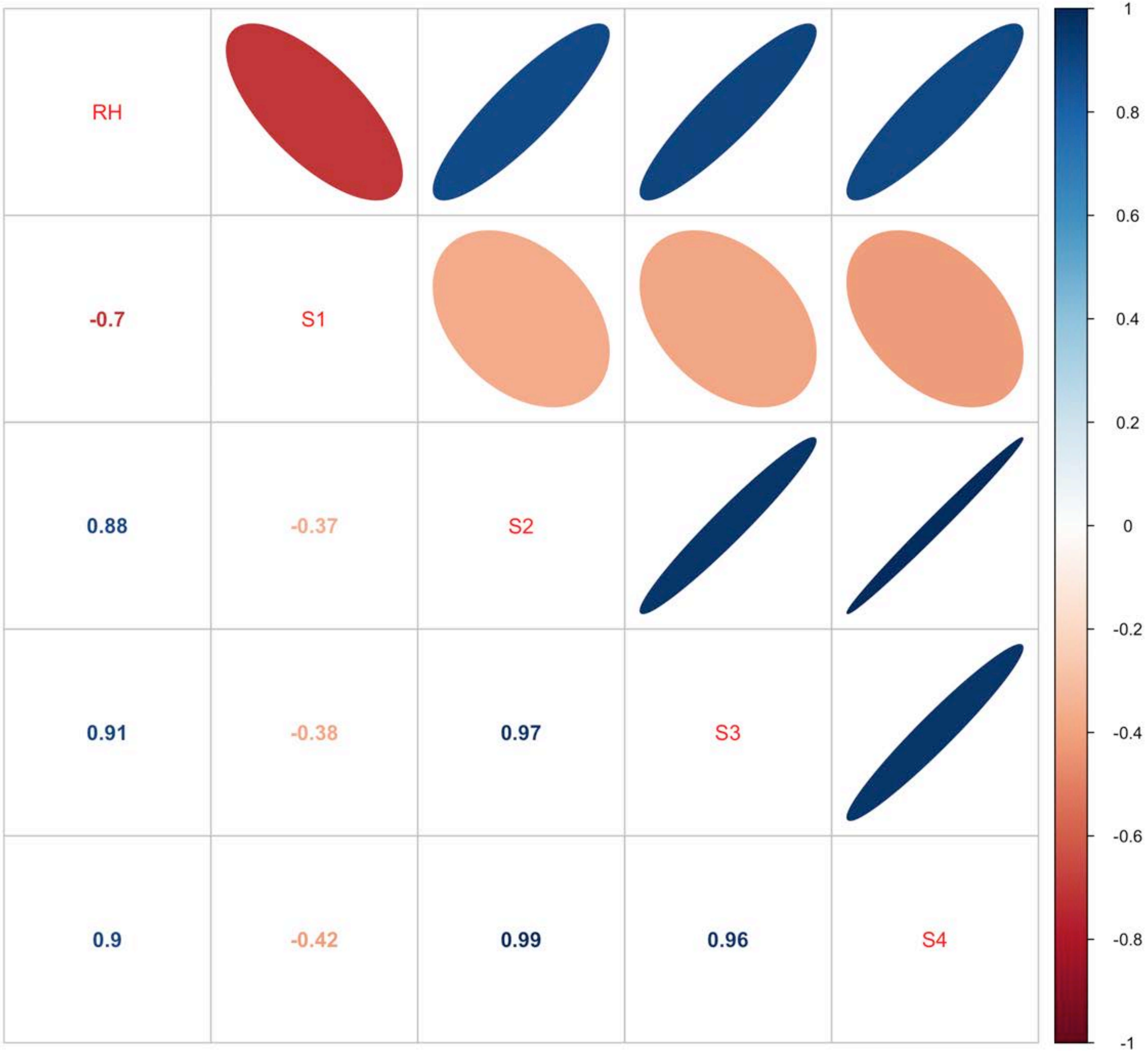

correlation Sensors ~ Humidity 5

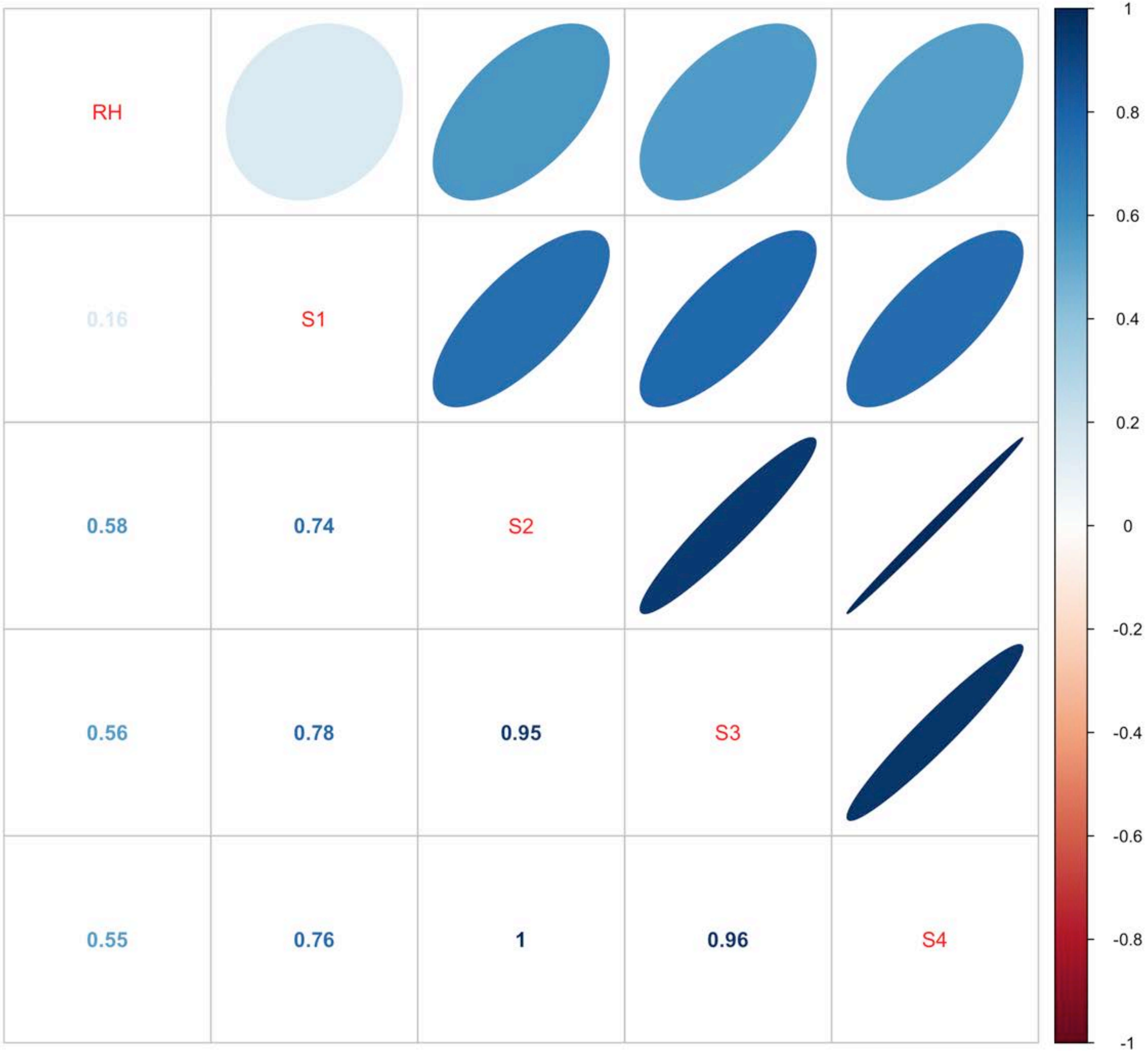

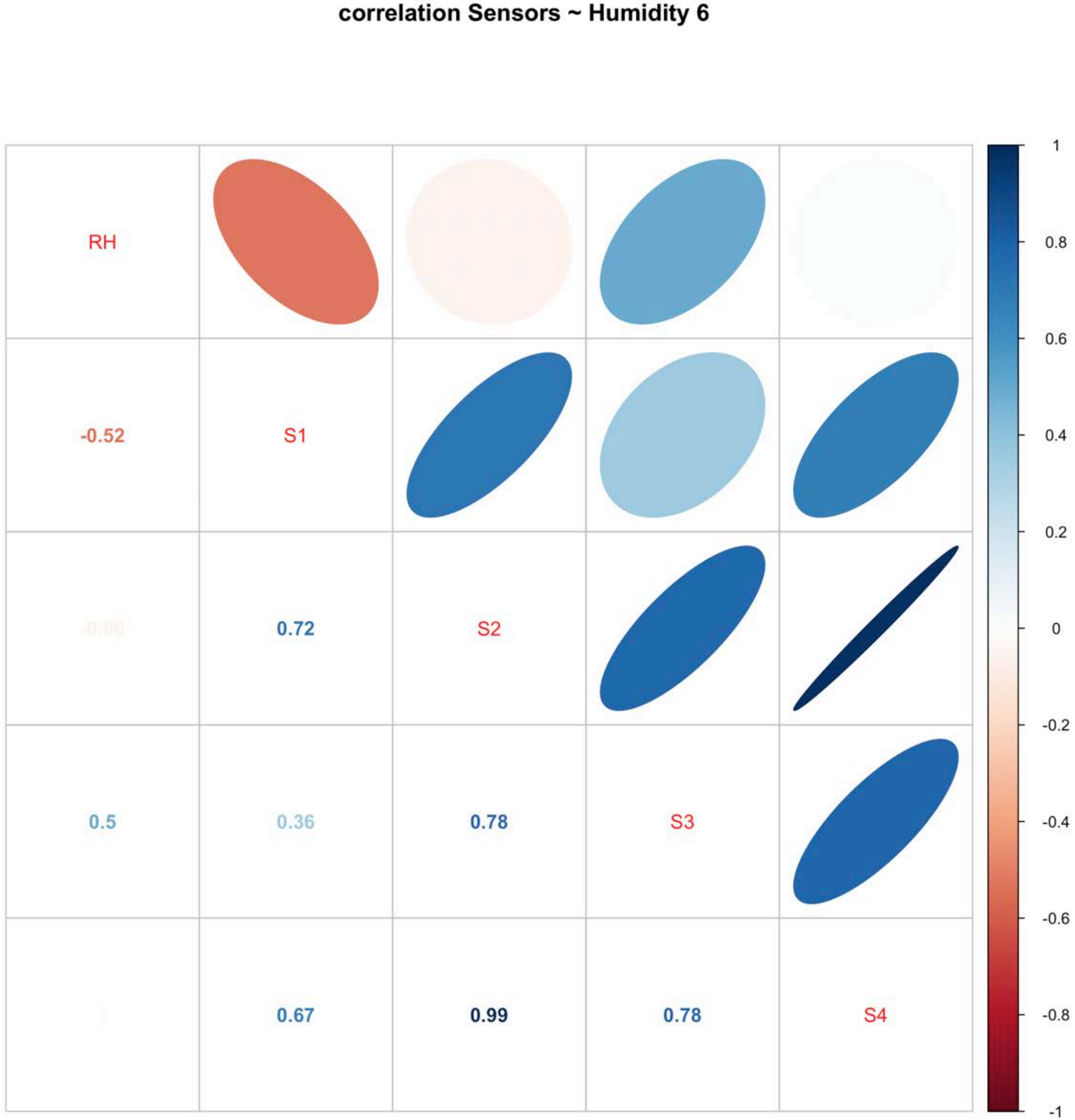
correlation Sensors ~ Humidity 6
RH
S1
S2
S3
S4
-0.52
-0.06
0.72
0.5
0.36
0.78
0.67
0.99
0.78
1
0.8
0.6
0.4
0.2
0
-0.2
-0.4
-0.6
-0.8
-1

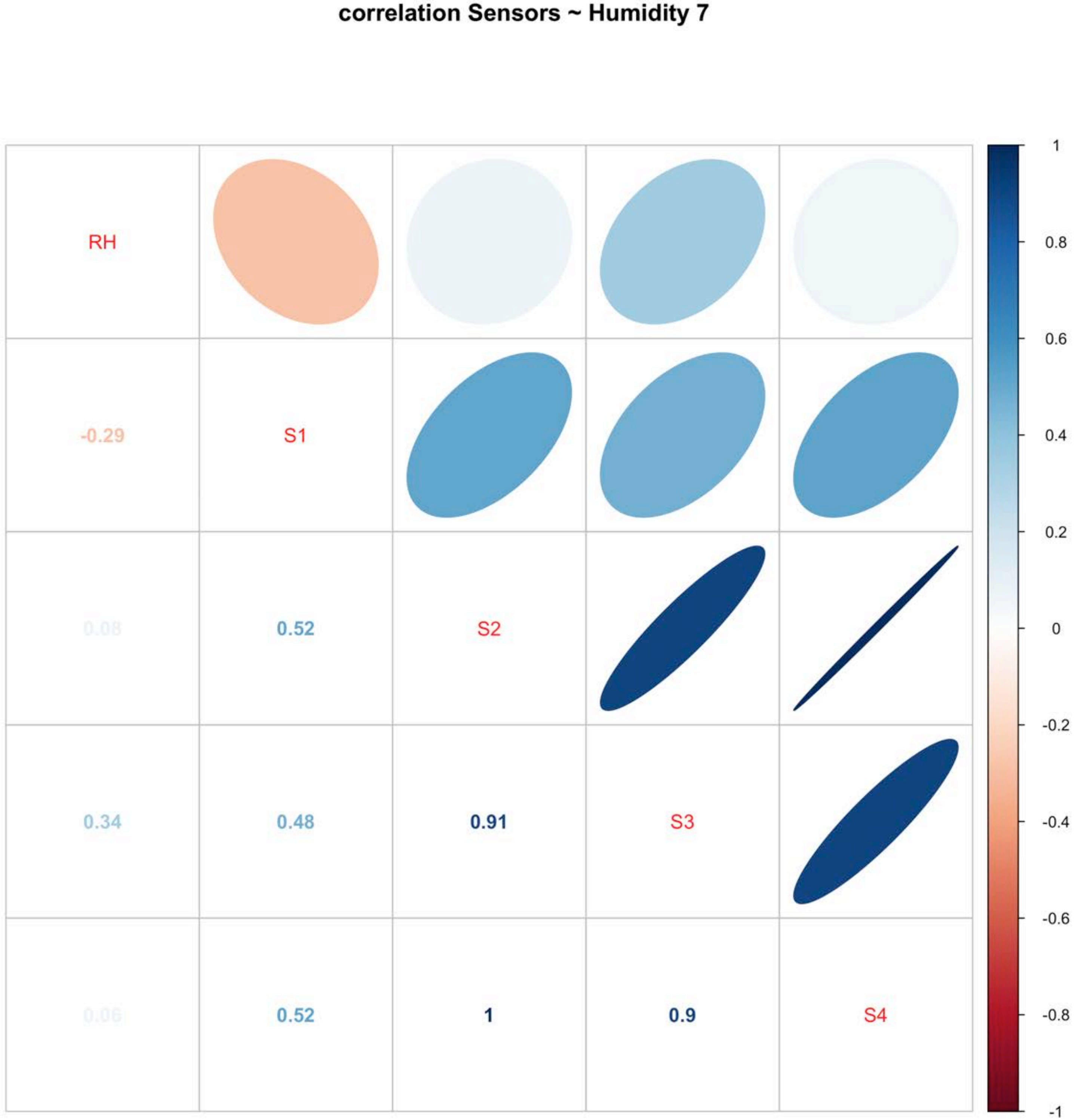
correlation Sensors ~ Humidity 7
RH
S1
S2
S3
S4
-0.29
0.08
0.52
0.34
0.48
0.91
0.06
0.52
1
0.9
1
0.8
0.6
0.4
0.2
0
-0.2
-0.4
-0.6
-0.8
-1

correlation Sensors ~ Humidity 8

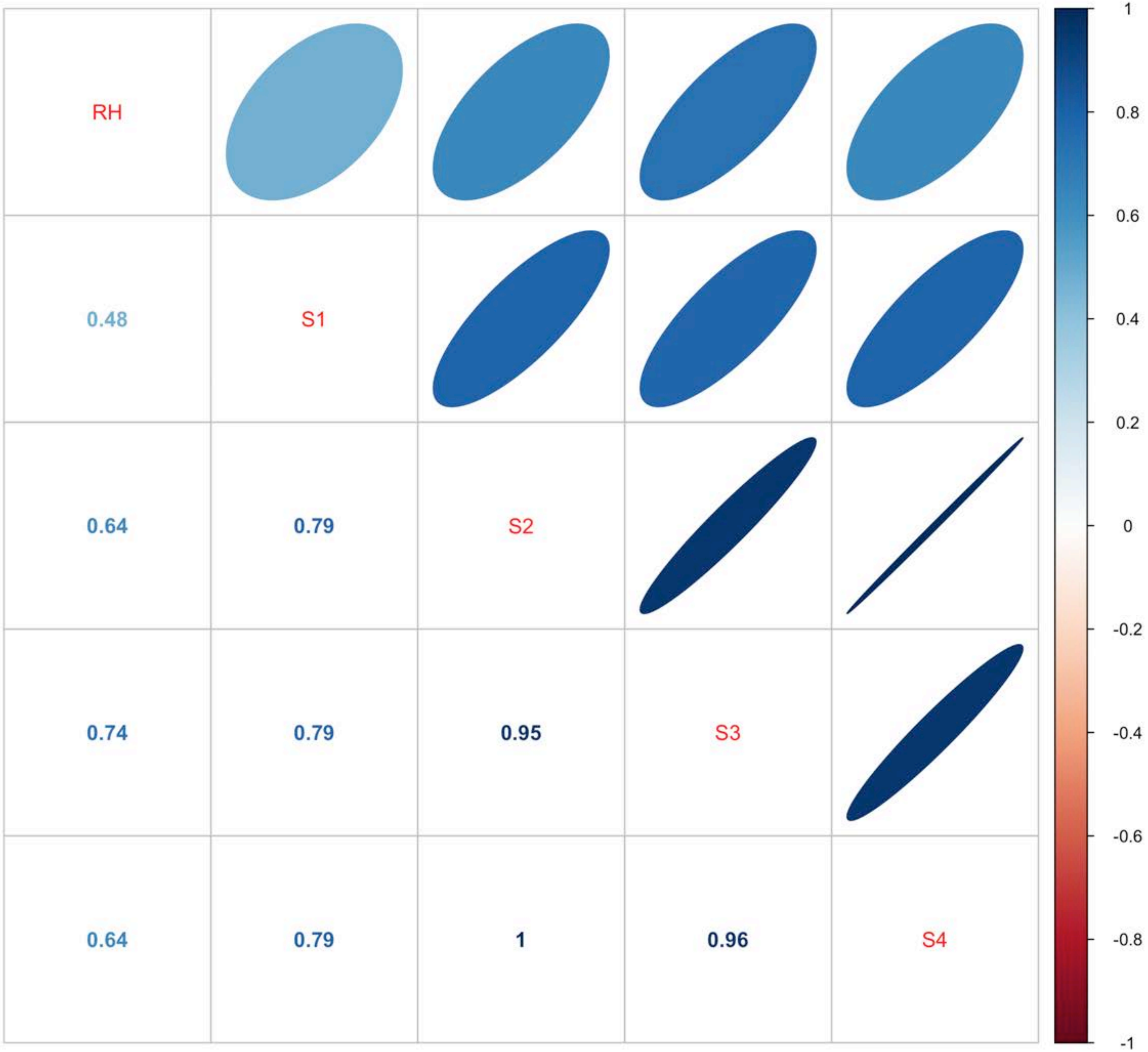

correlation Sensors ~ Humidity 9

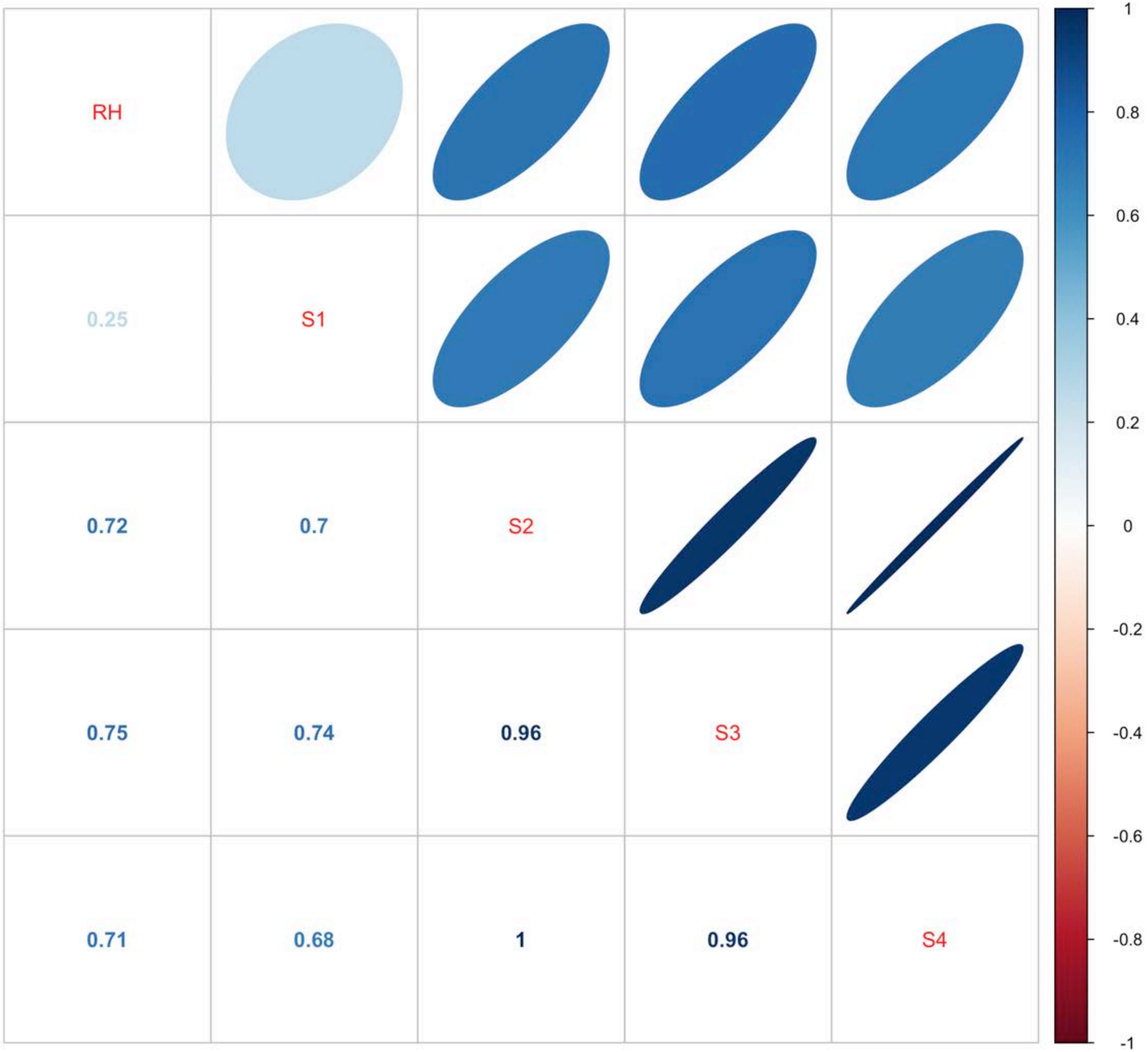

correlation Sensors ~ Humidity 11

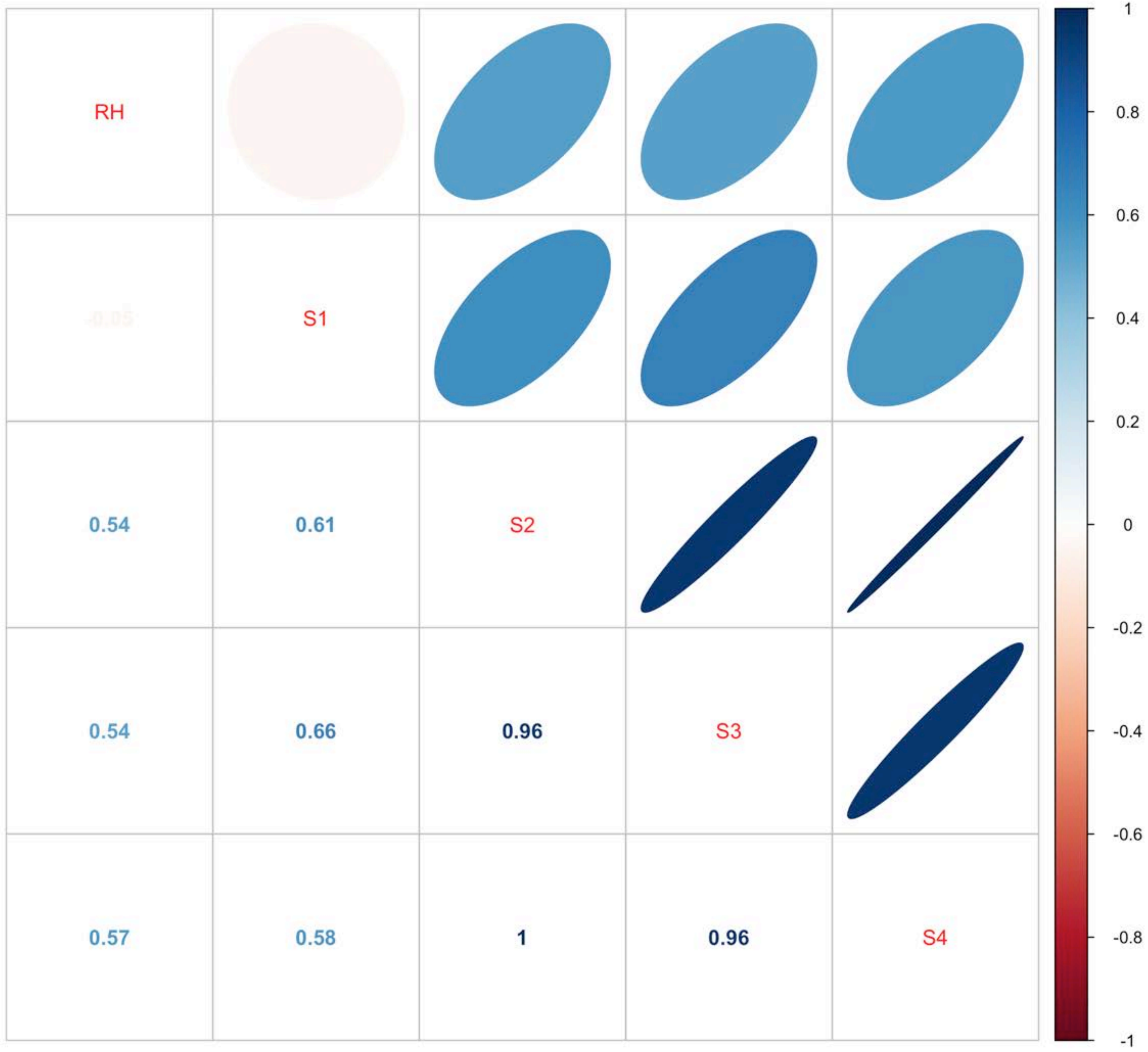

correlation Sensors ~ Humidity 12

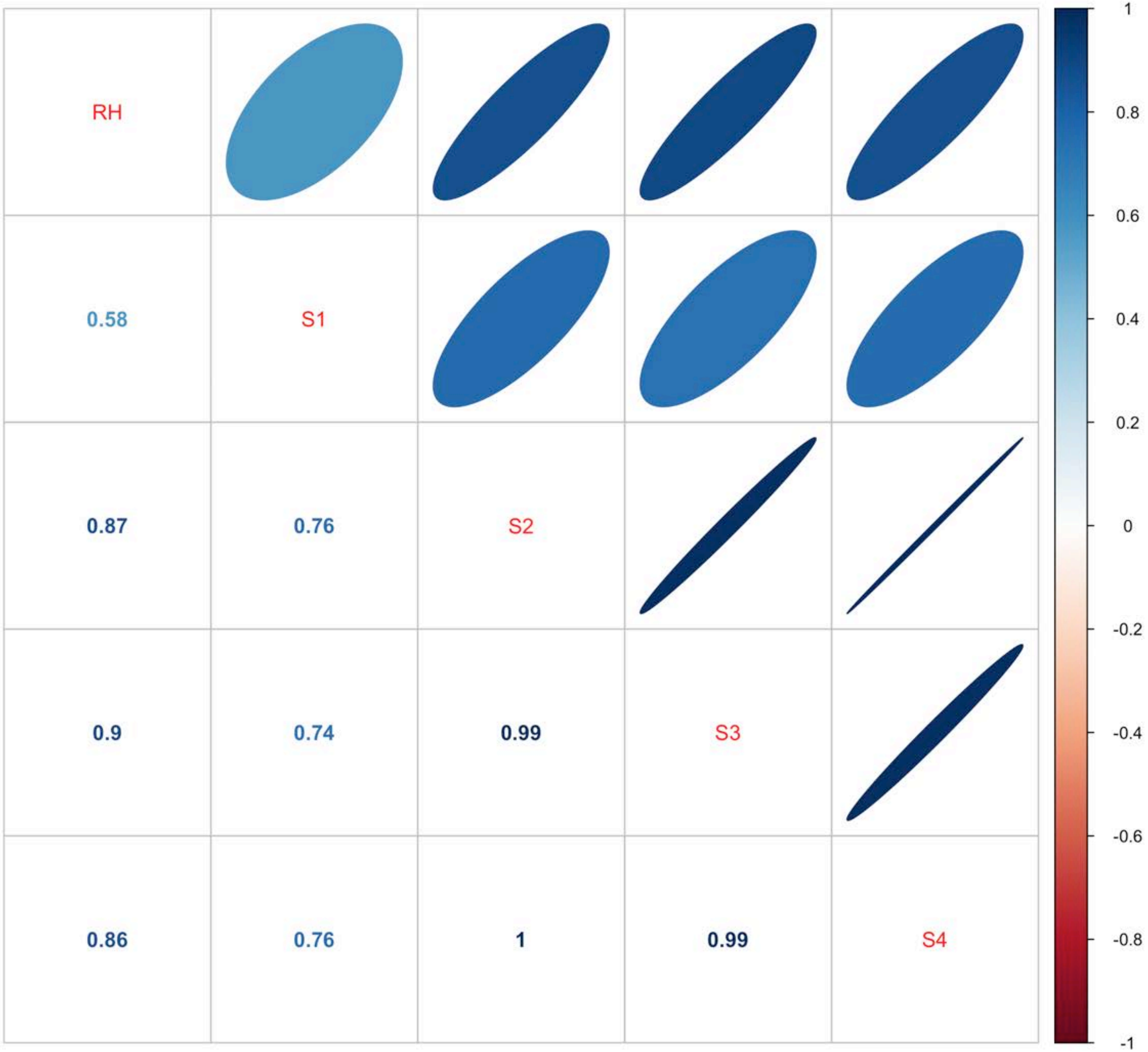

correlation Sensors ~ Humidity 103

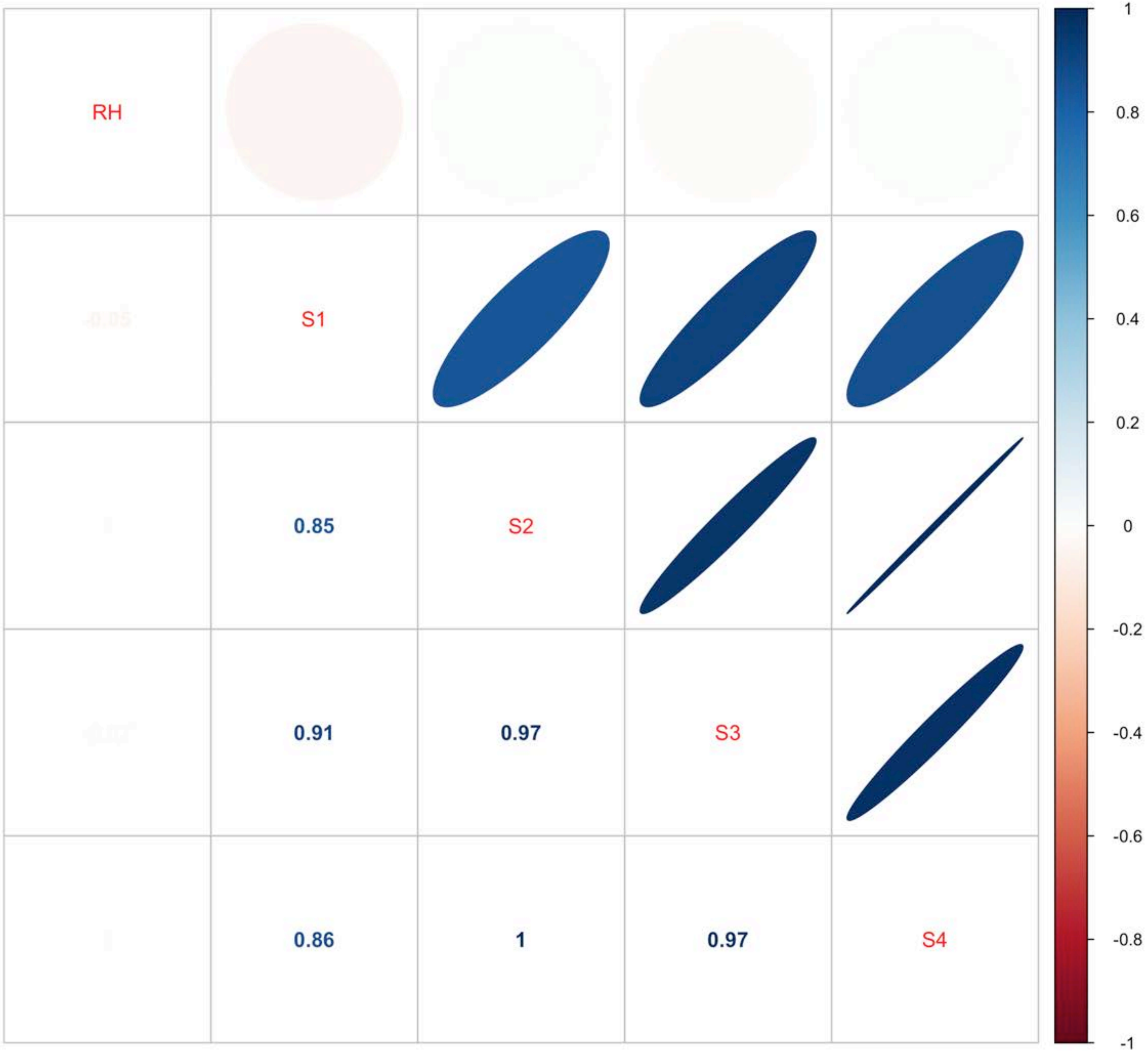

correlation Sensors ~ Humidity 104

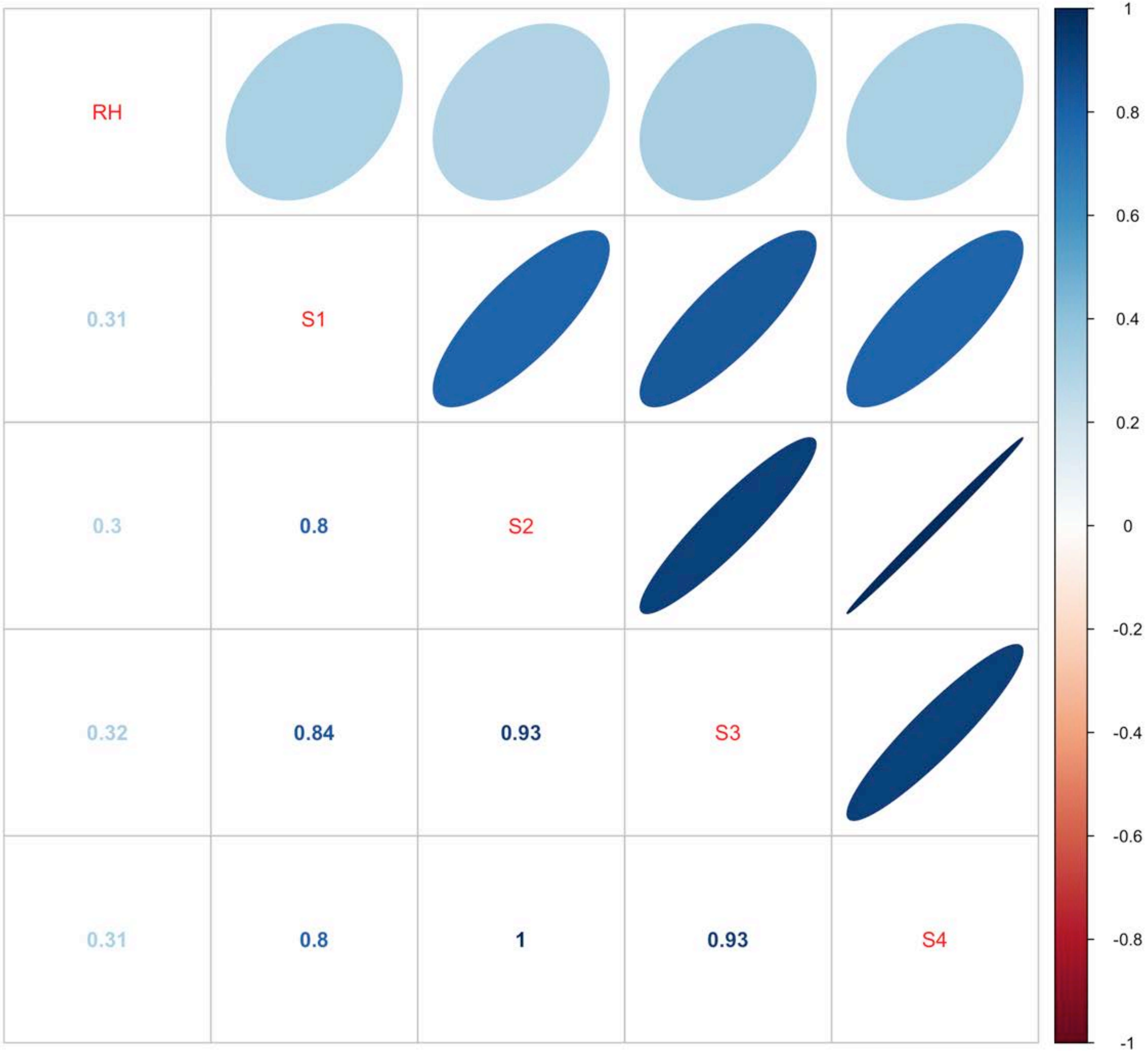

correlation Sensors ~ Humidity 106

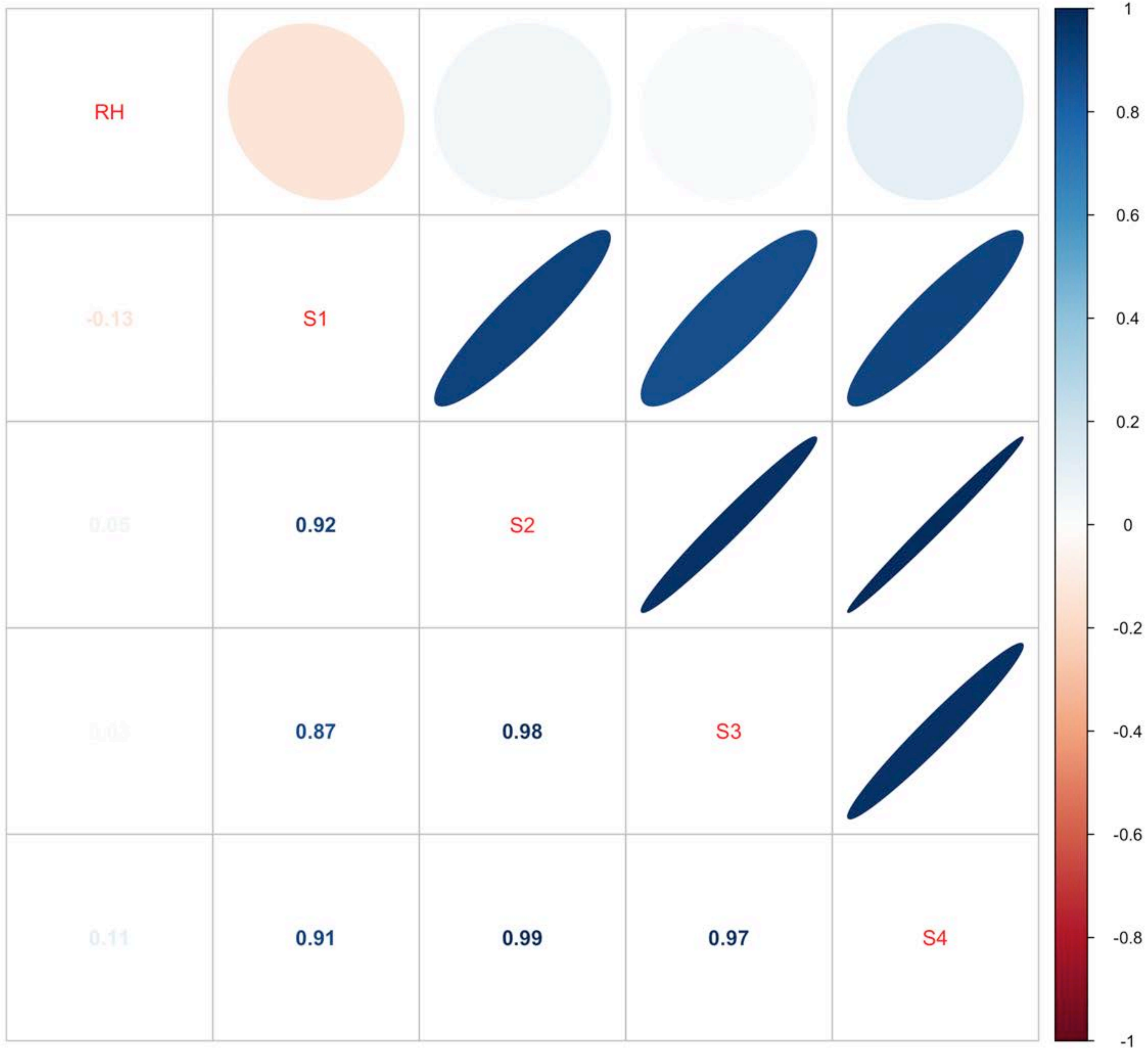

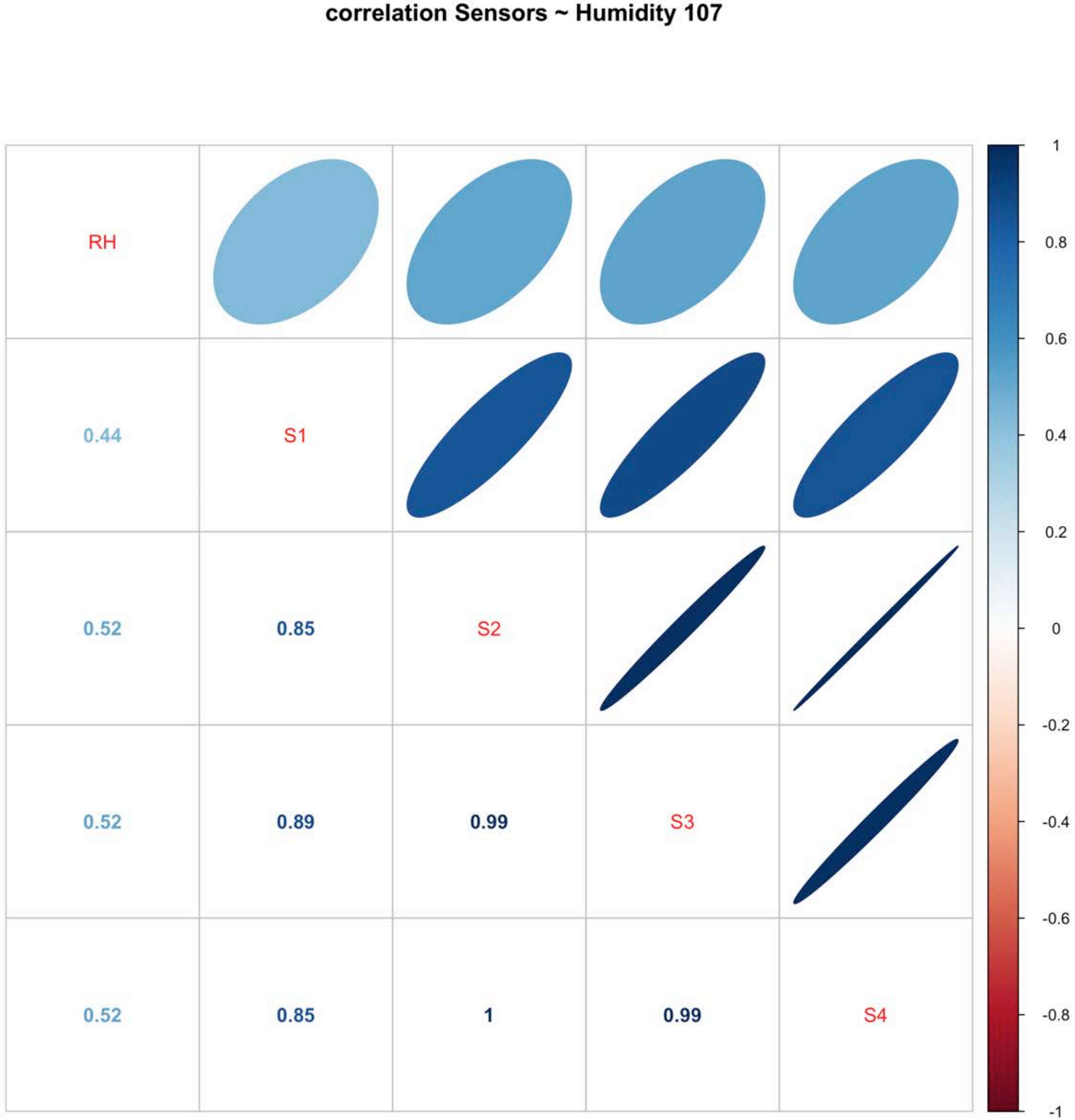
correlation Sensors ~ Humidity 107
RH
S1
S2
S3
S4
0.44
0.52
0.85
0.52
0.89
0.99
0.52
0.85
1
0.99
1
0.8
0.6
0.4
0.2
0
-0.2
-0.4
-0.6
-0.8
-1

correlation Sensors ~ Humidity 109

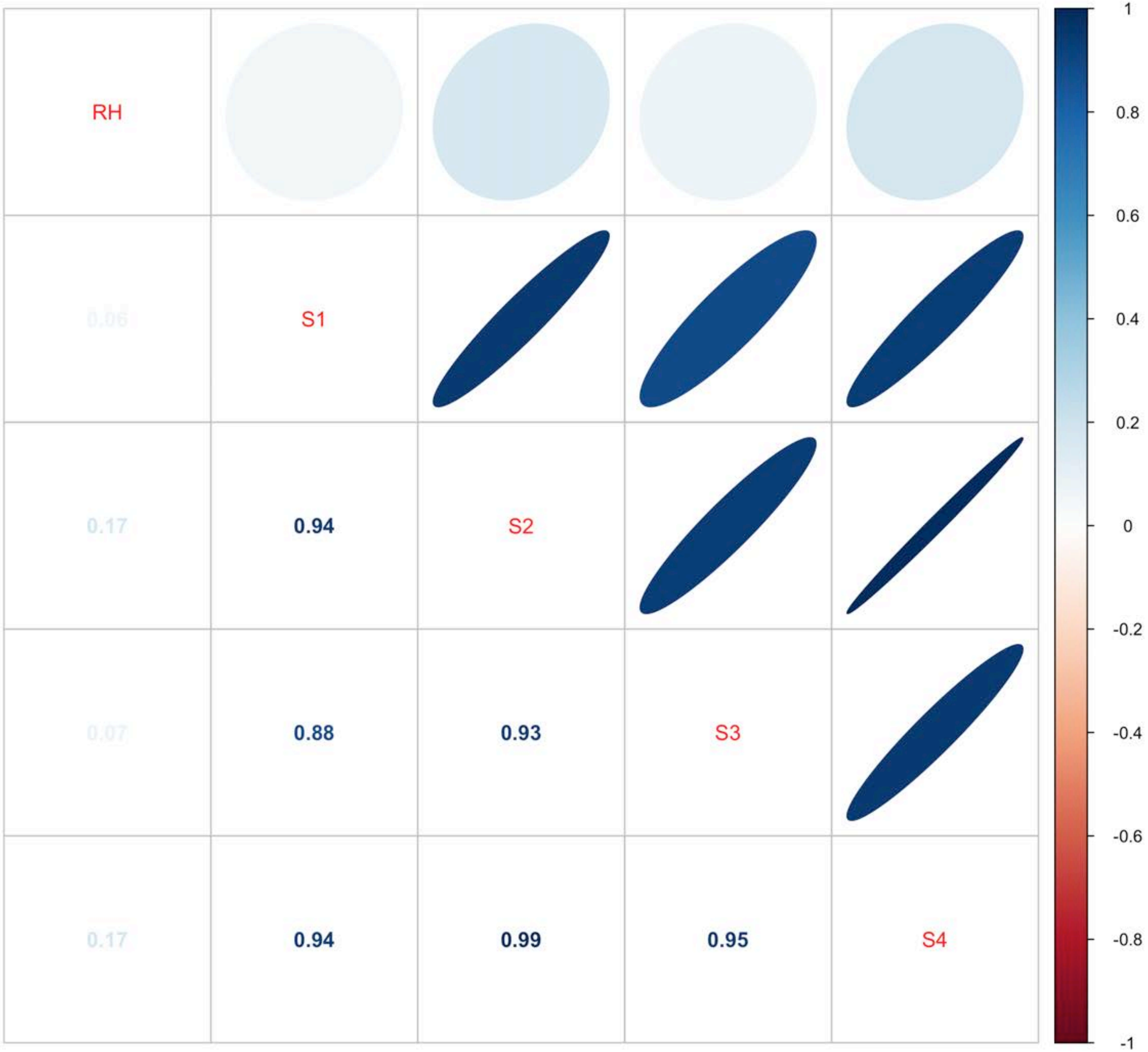

correlation Sensors ~ Humidity 1010

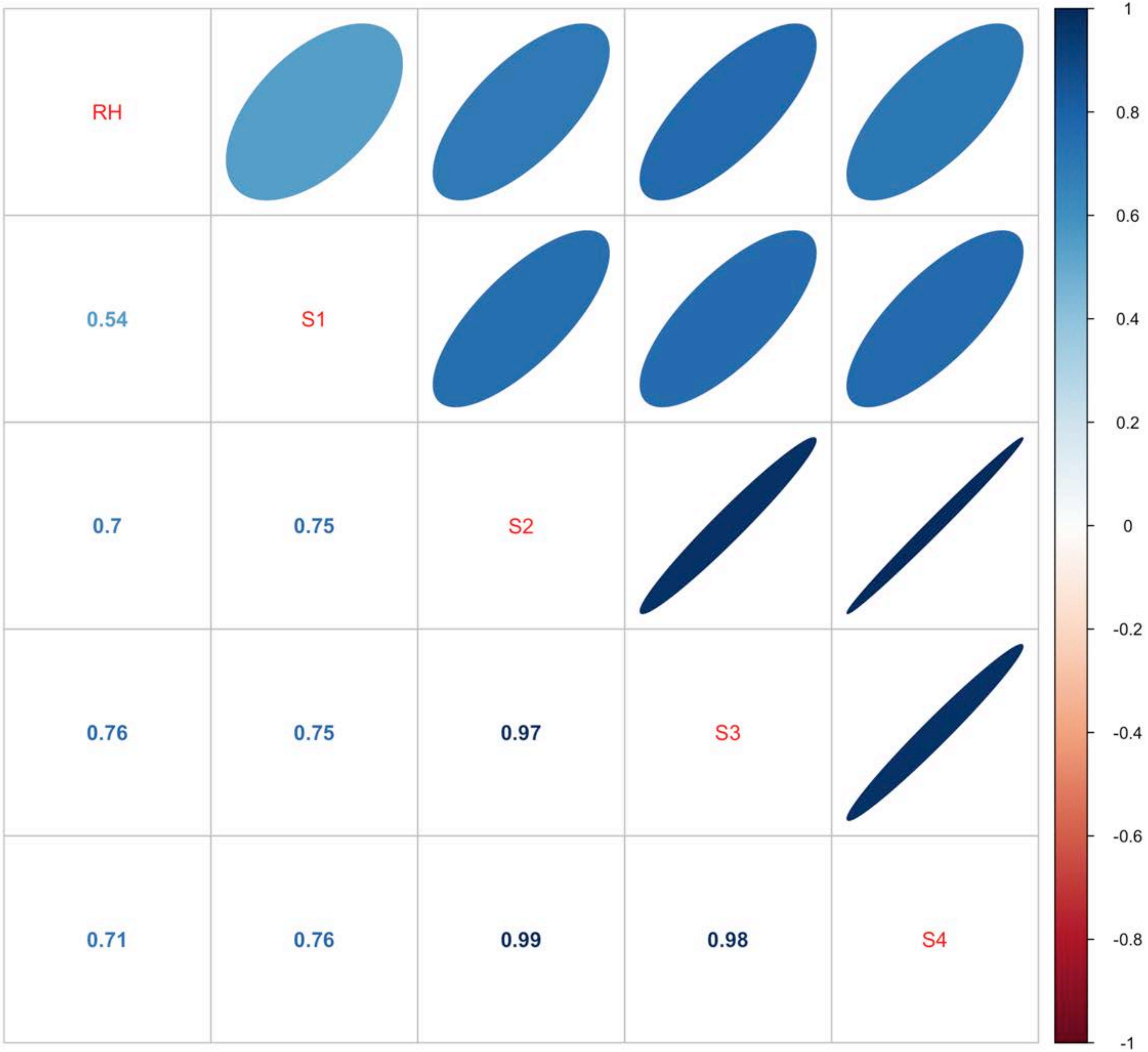

correlation Sensors ~ Humidity 1012

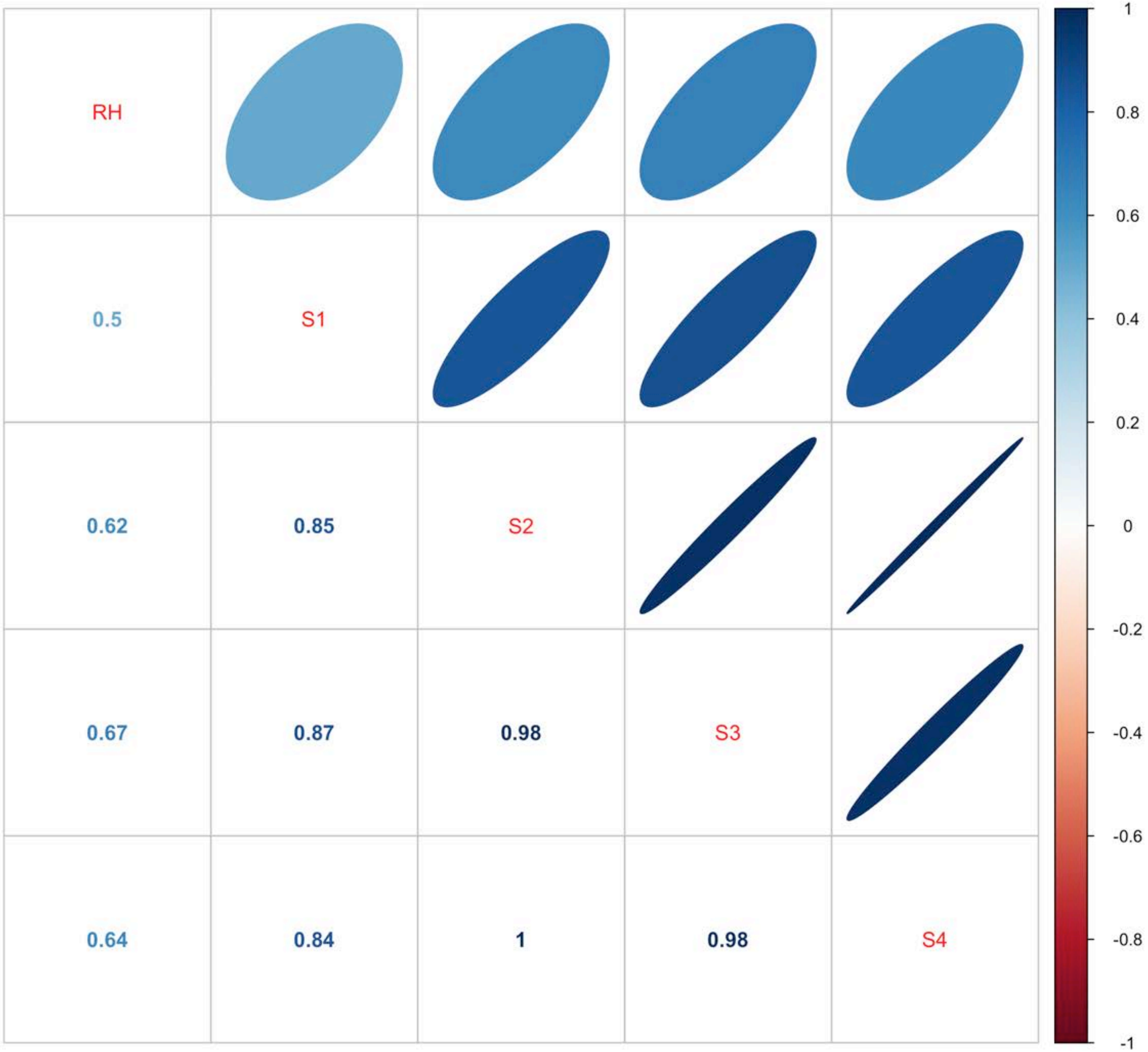

correlation Sensors ~ Humidity 1014

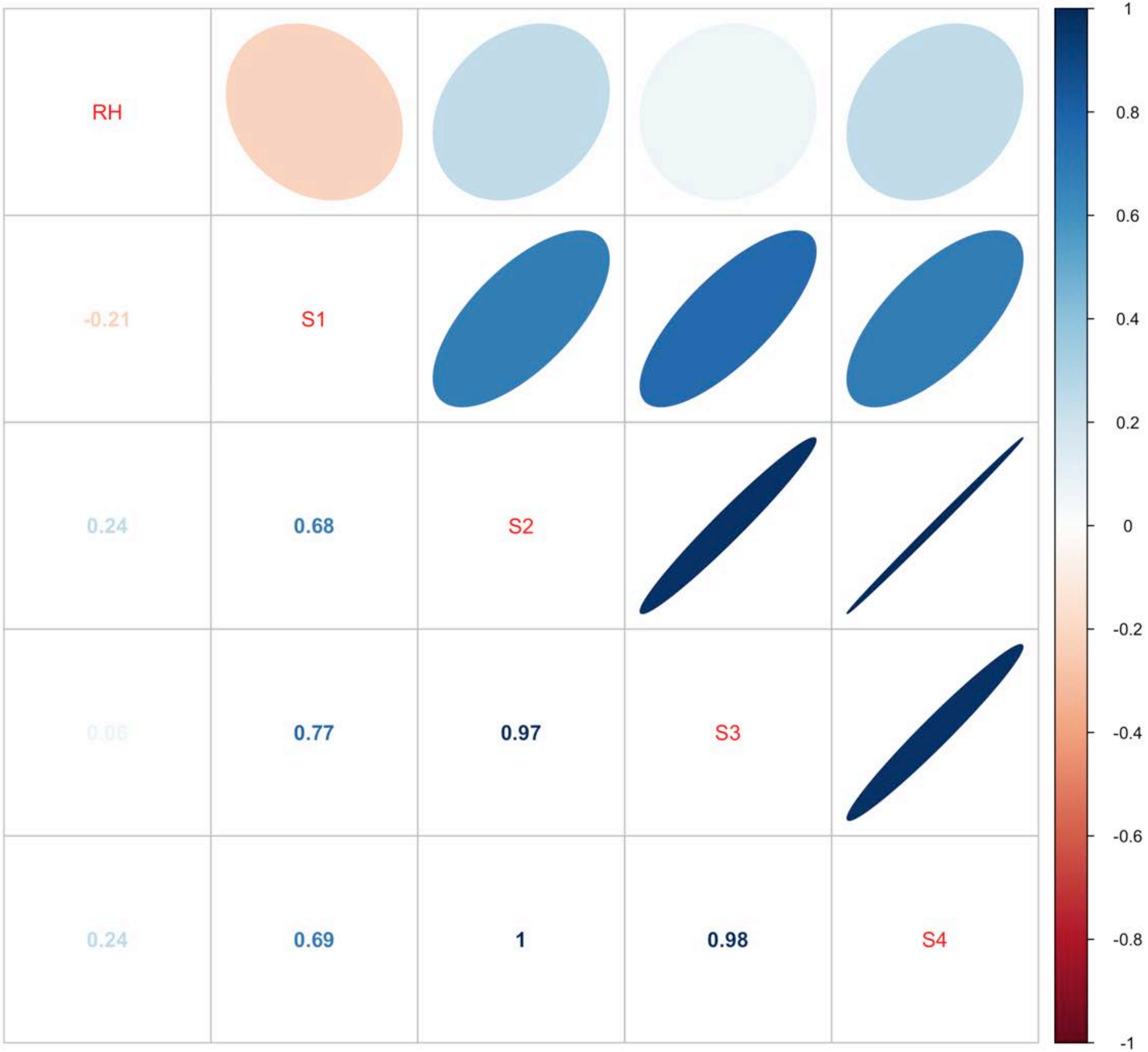

correlation Sensors ~ Humidity 1015

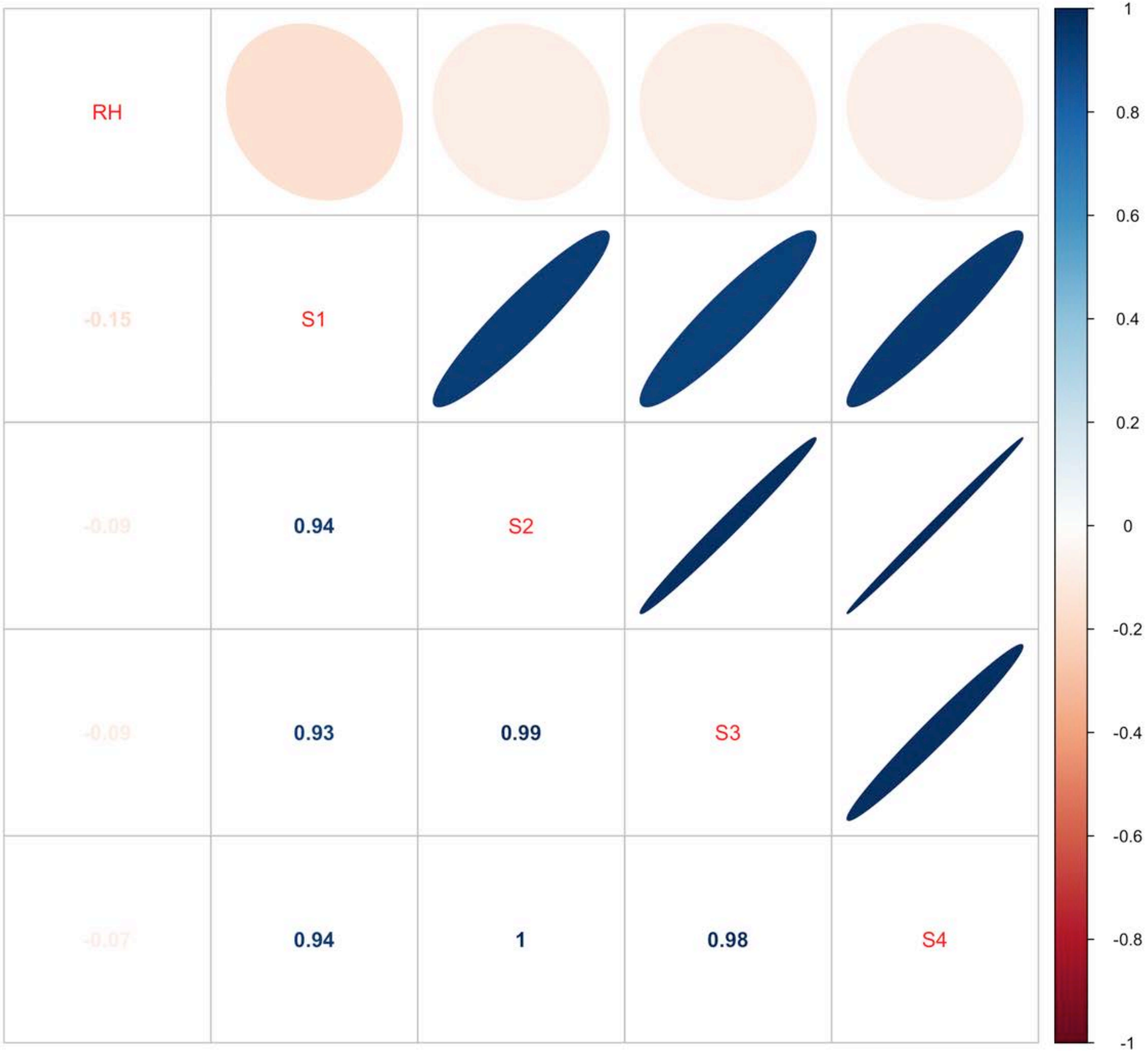

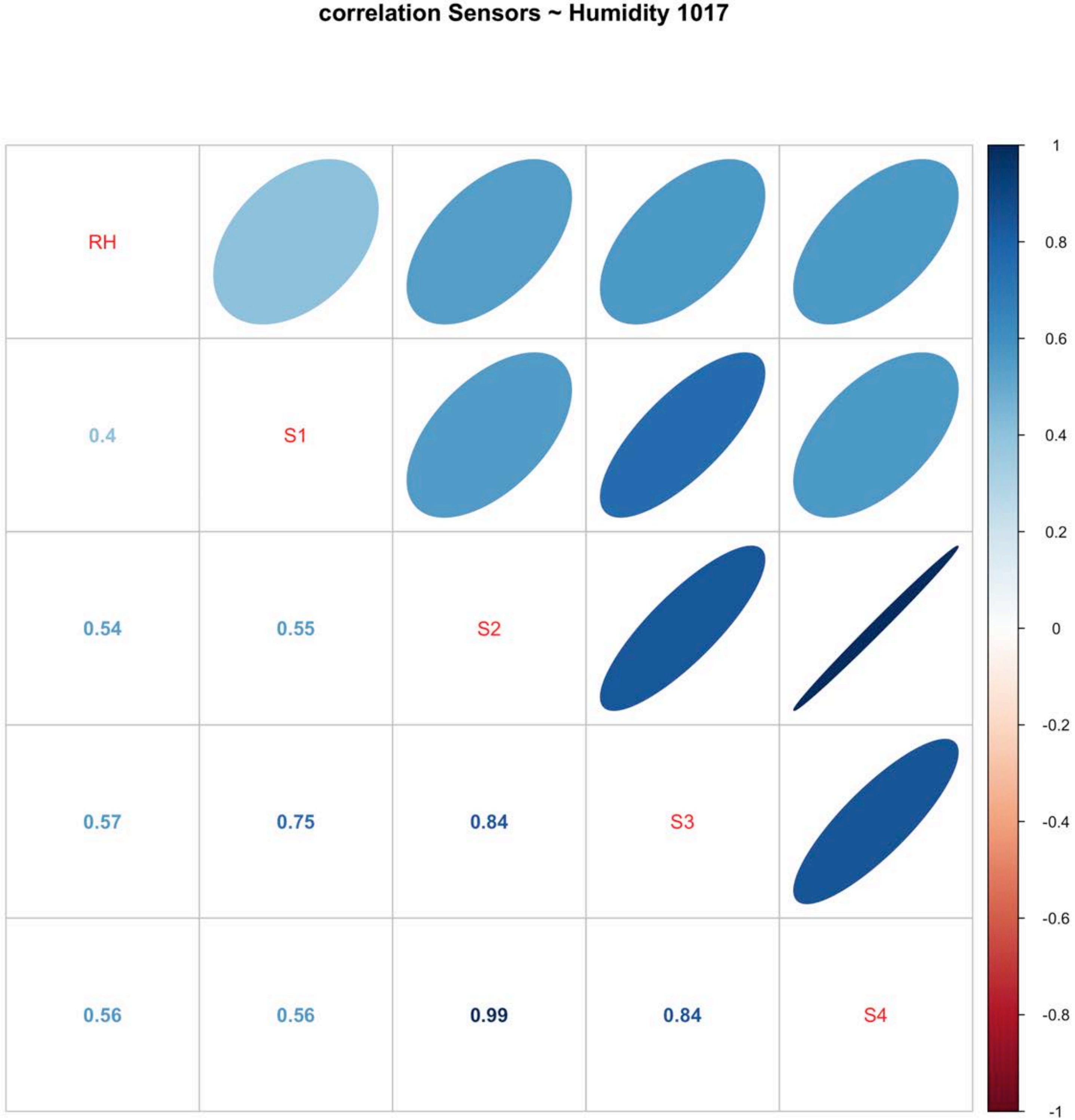
correlation Sensors ~ Humidity 1017
RH
S1
S2
S3
S4
0.4
0.54
0.55
0.57
0.75
0.84
0.56
0.56
0.99
0.84
1
0.8
0.6
0.4
0.2
0
-0.2
-0.4
-0.6
-0.8
-1

correlation Sensors ~ Humidity 1034

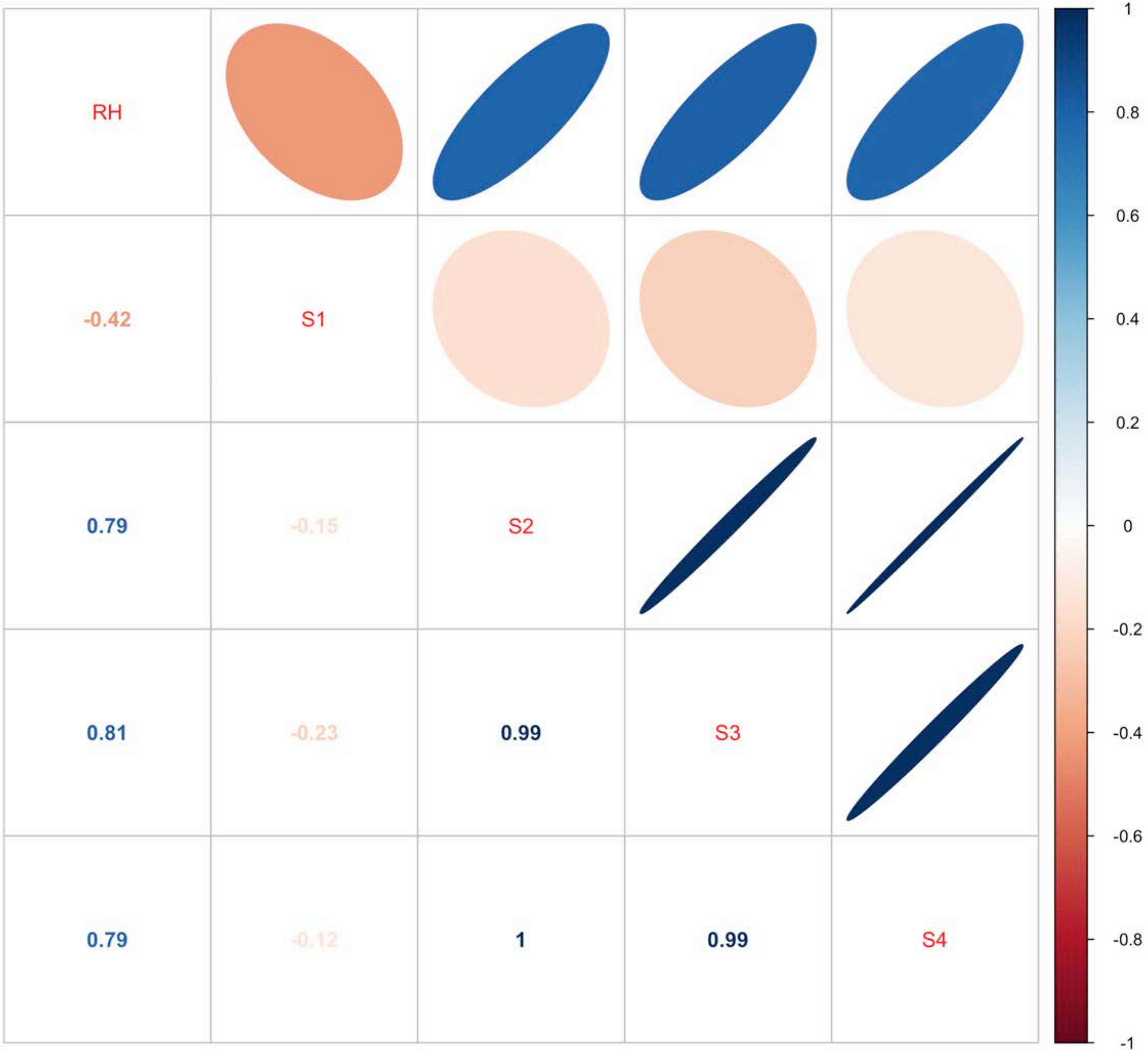

correlation Sensors ~ Humidity 1045

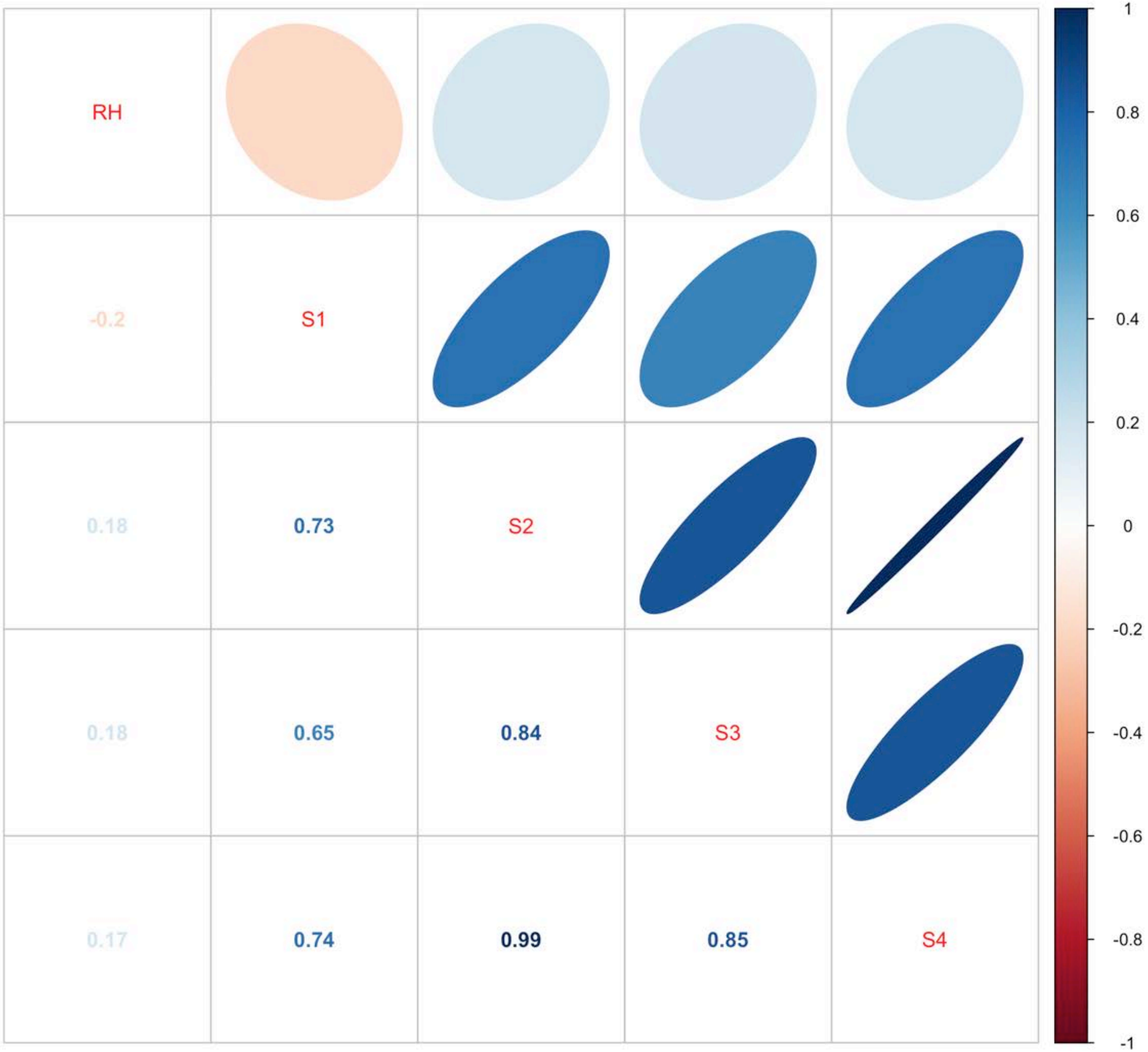

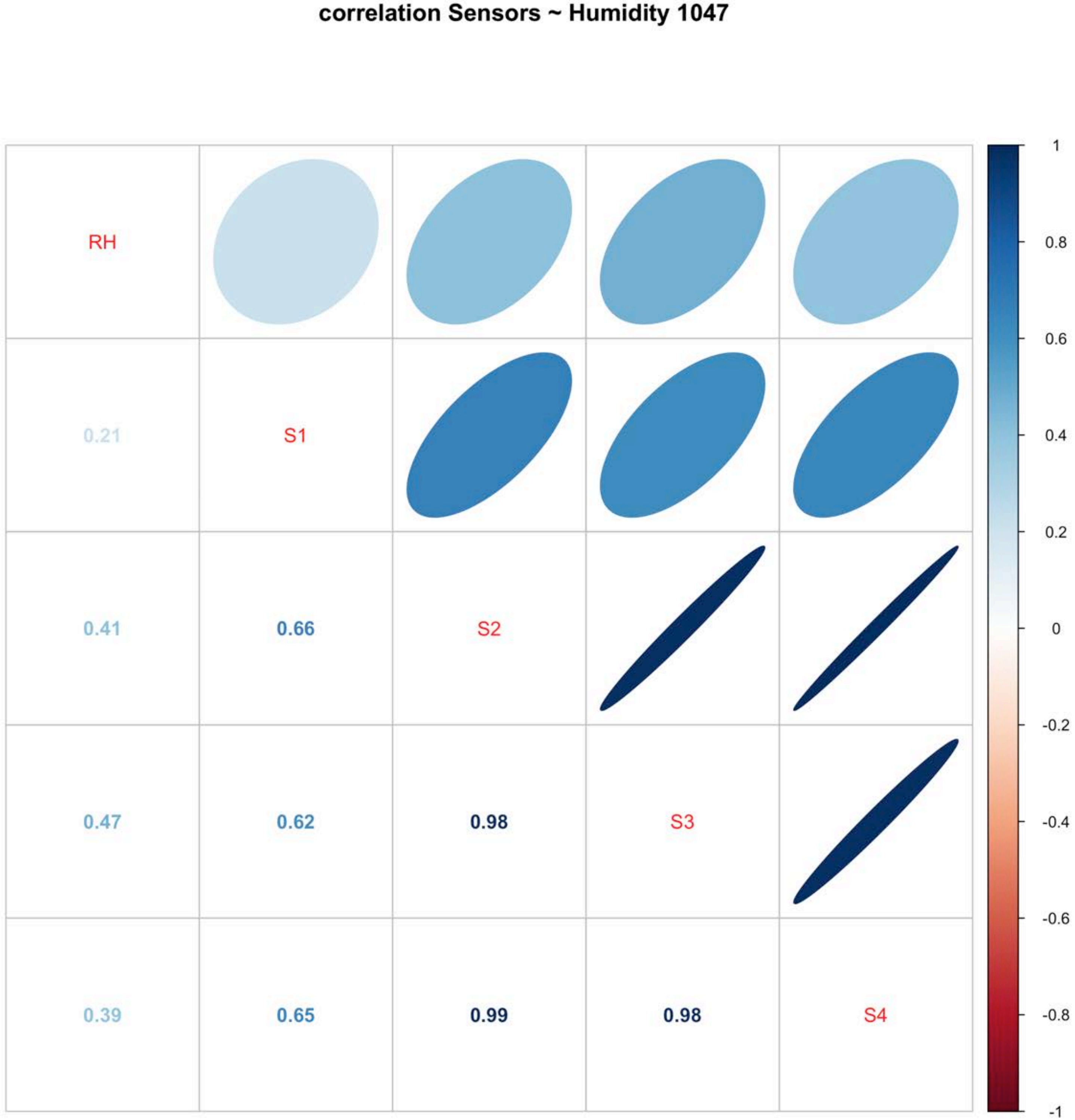
correlation Sensors ~ Humidity 1047
RH
S1
S2
S3
S4
0.21
0.41
0.66
0.47
0.62
0.98
0.39
0.65
0.99
0.98
1
0.8
0.6
0.4
0.2
0
-0.2
-0.4
-0.6
-0.8
-1

correlation Sensors ~ Humidity 1048

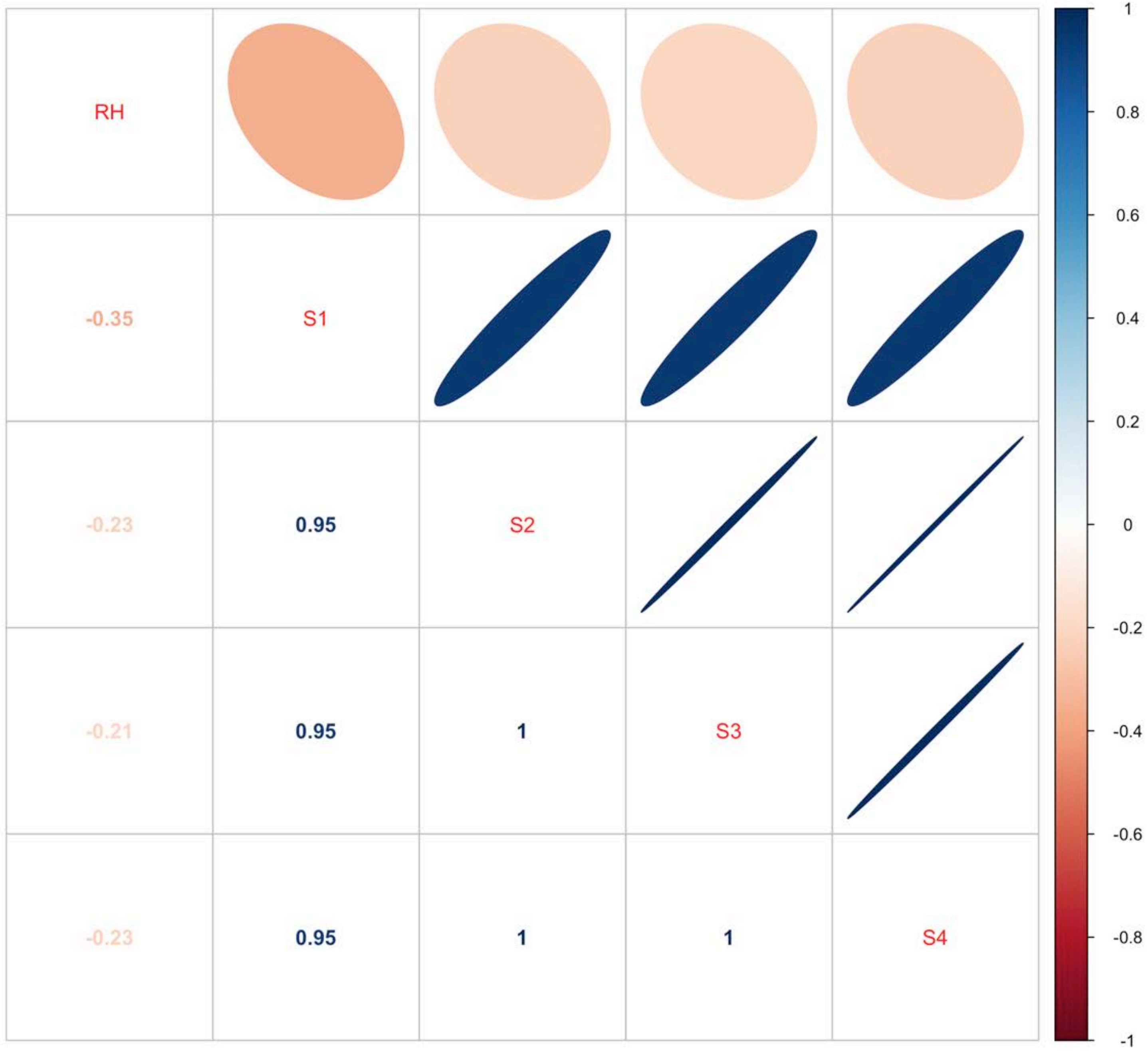

correlation Sensors ~ Humidity 1050

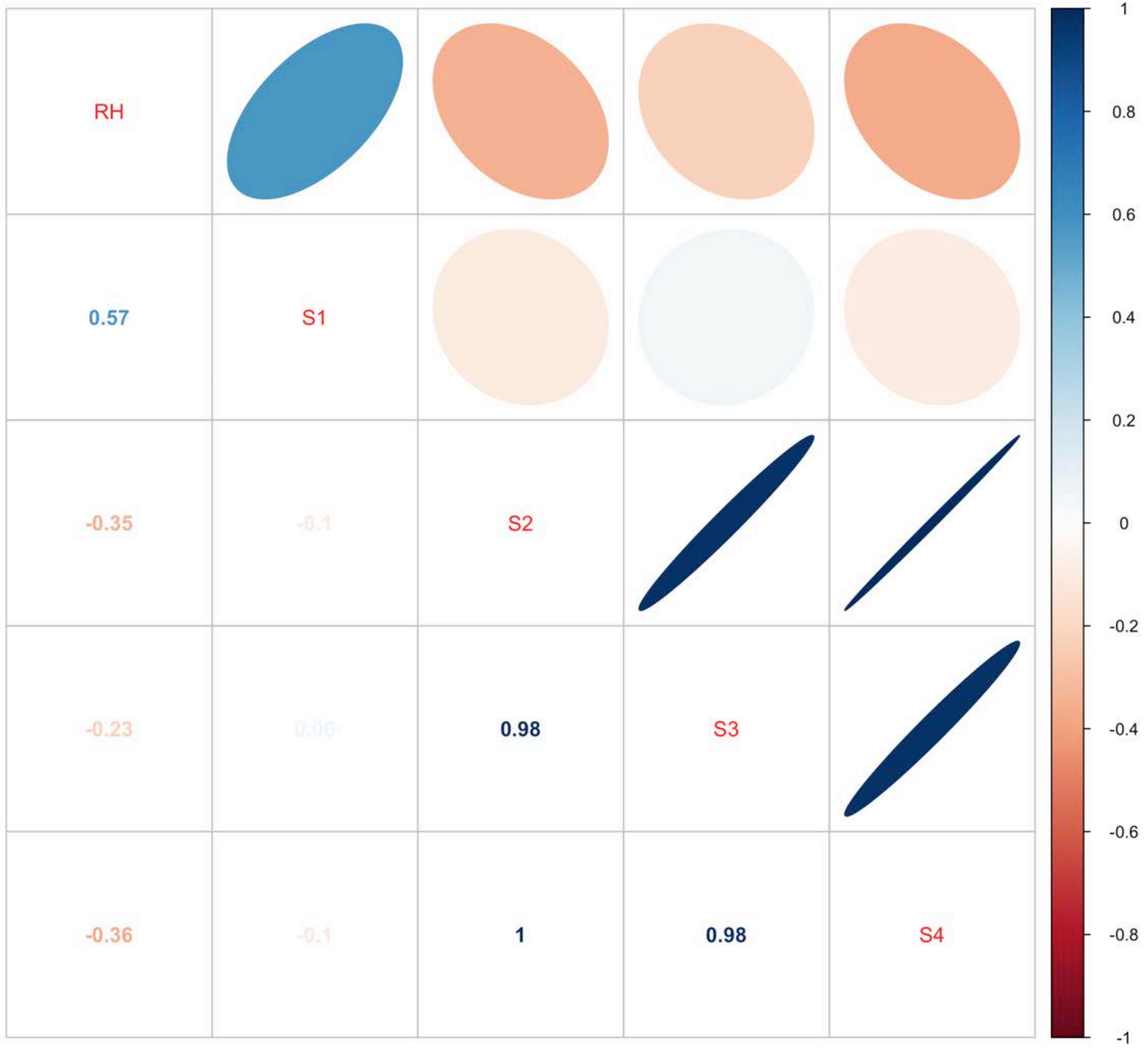

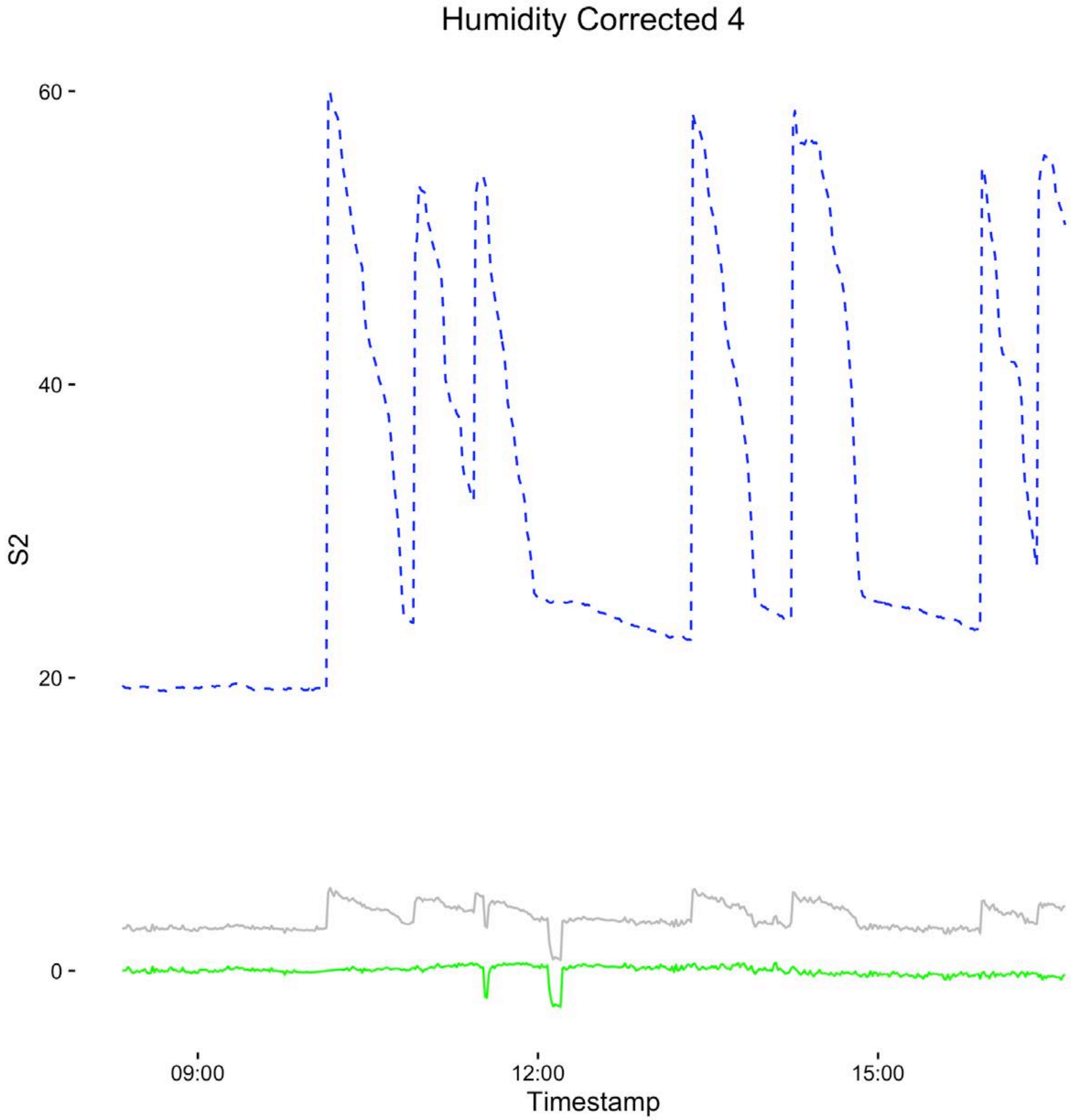
Humidity Corrected 4
60
40
20
0
S2
09:00
12:00
15:00
Timestamp

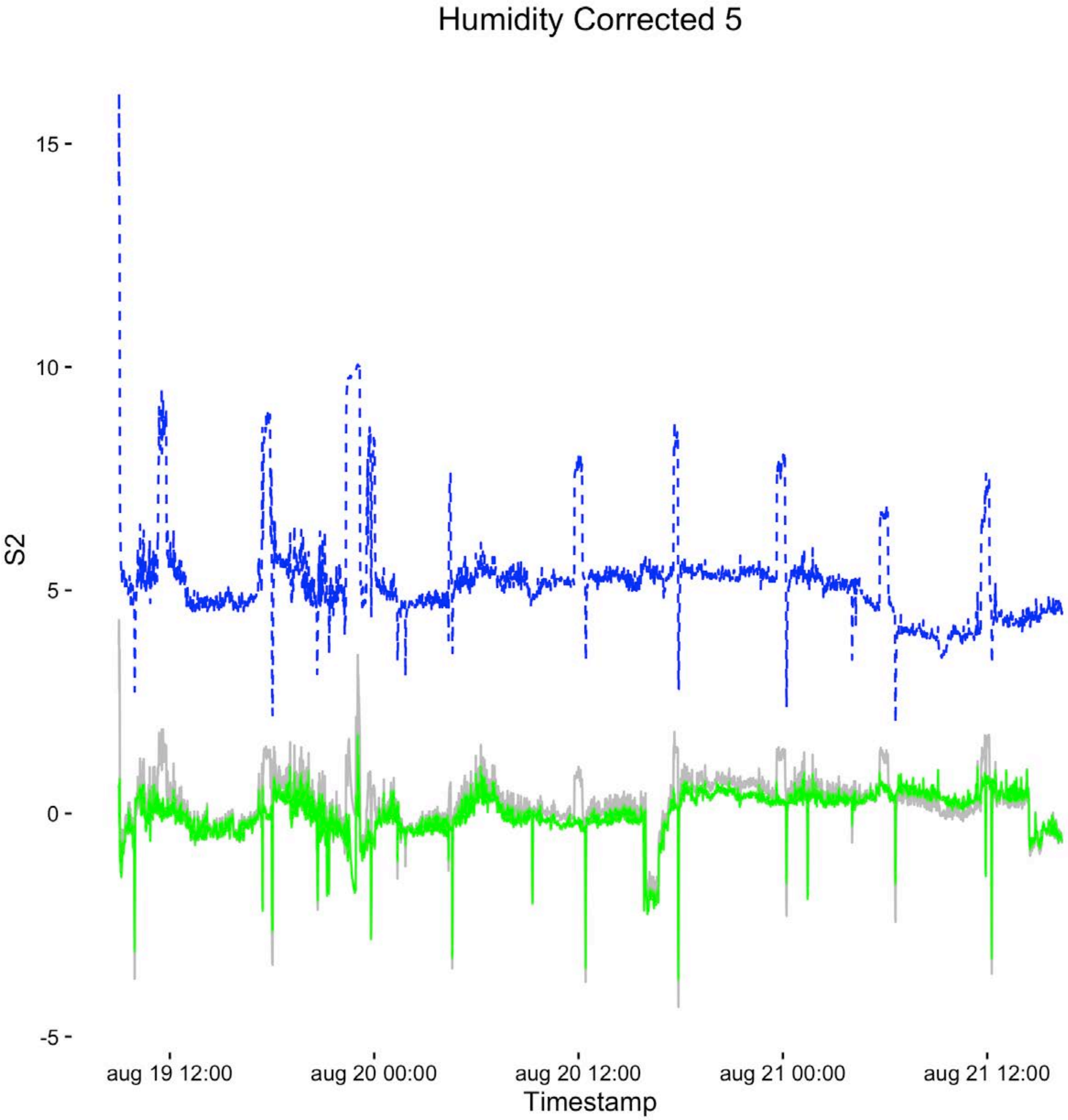
Humidity Corrected 5
15
10
5
0
-5
S2
aug 19 12:00
aug 20 00:00
aug 20 12:00
aug 21 00:00
aug 21 12:00
Timestamp

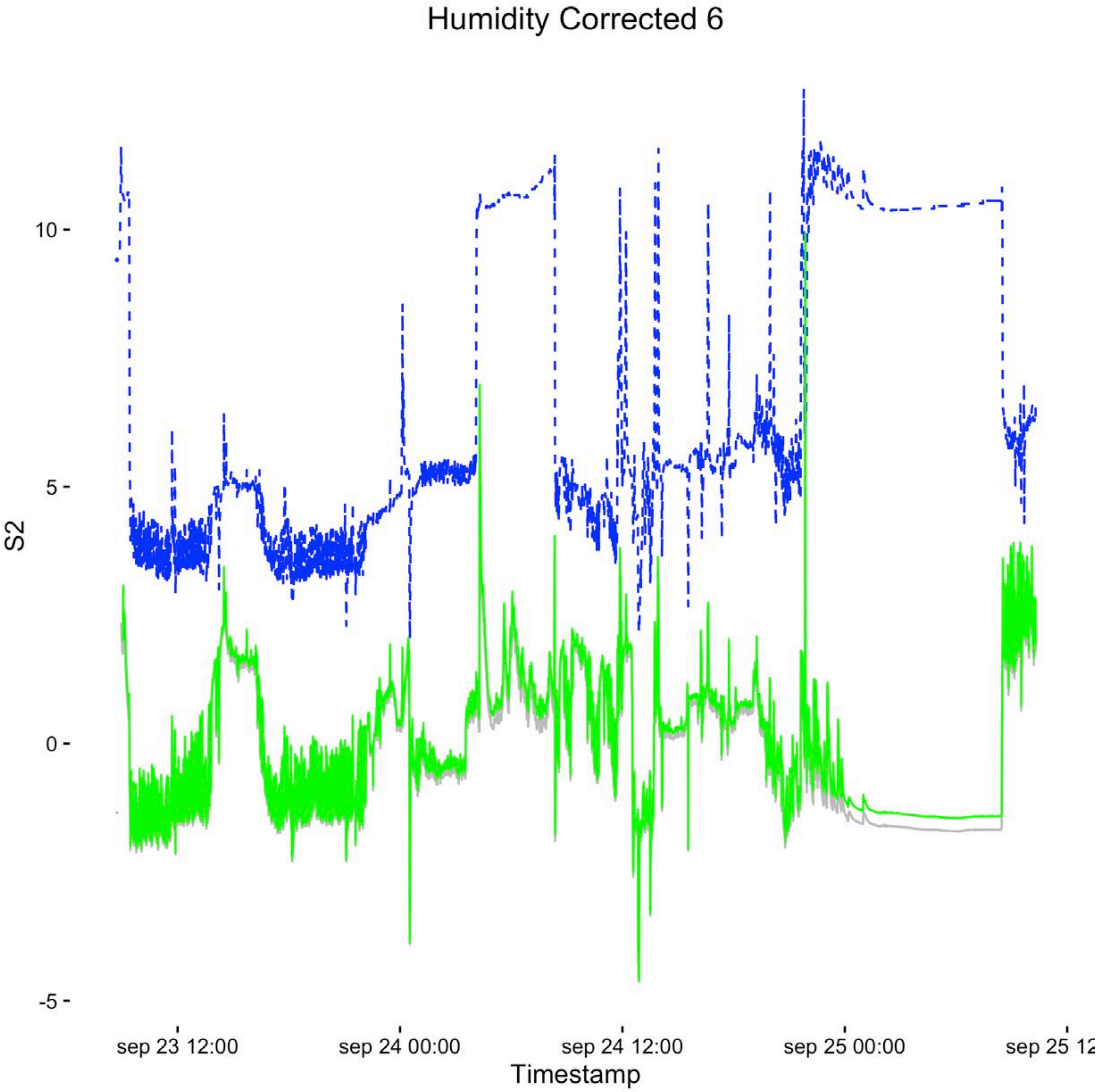
Humidity Corrected 6
S2
10
5
0
-5
sep 23 12:00
sep 24 00:00
sep 24 12:00
sep 25 00:00
sep 25 12
Timestamp

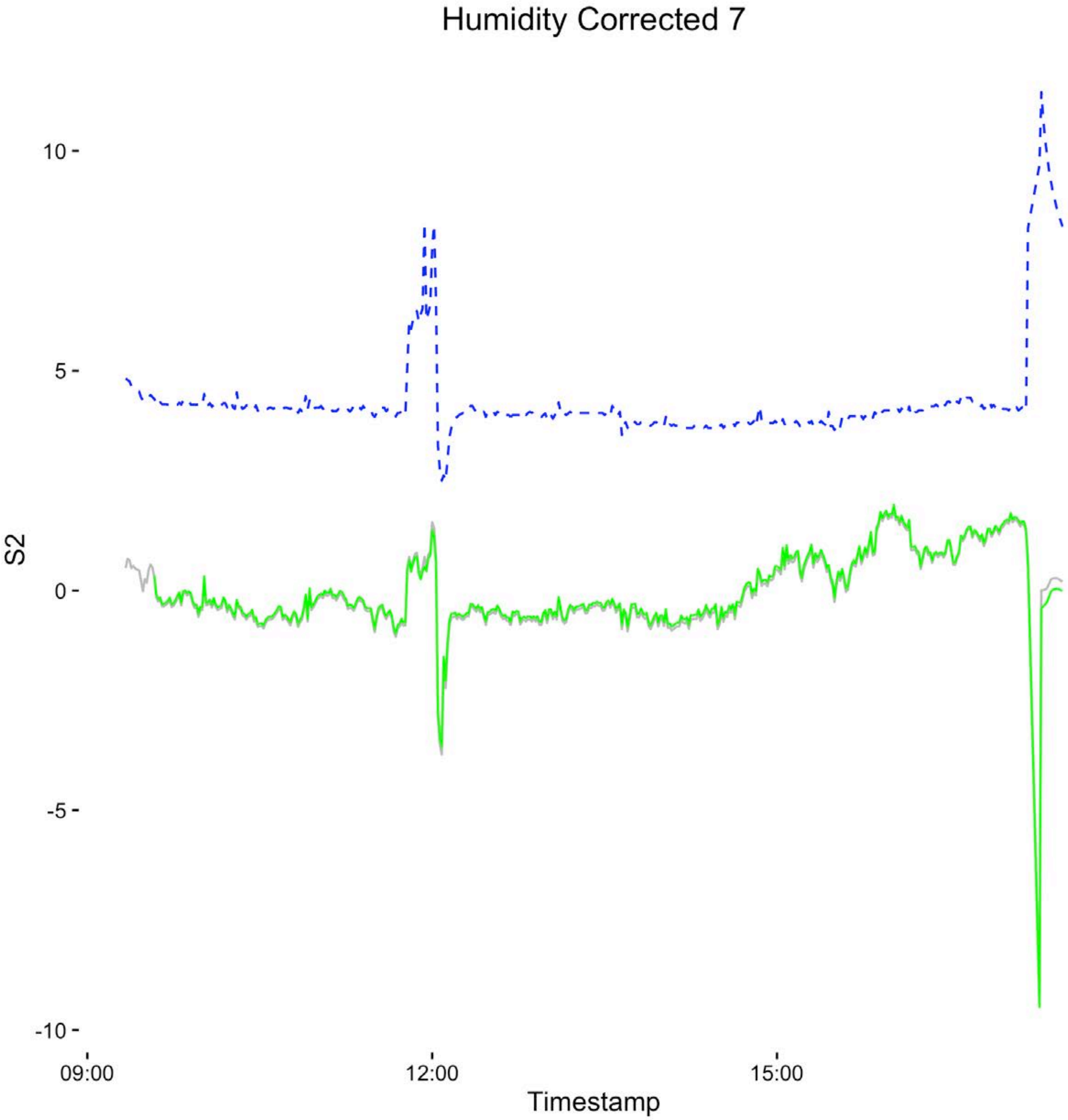
Humidity Corrected 7
S2
10
5
0
-5
-10
09:00
12:00
15:00
Timestamp

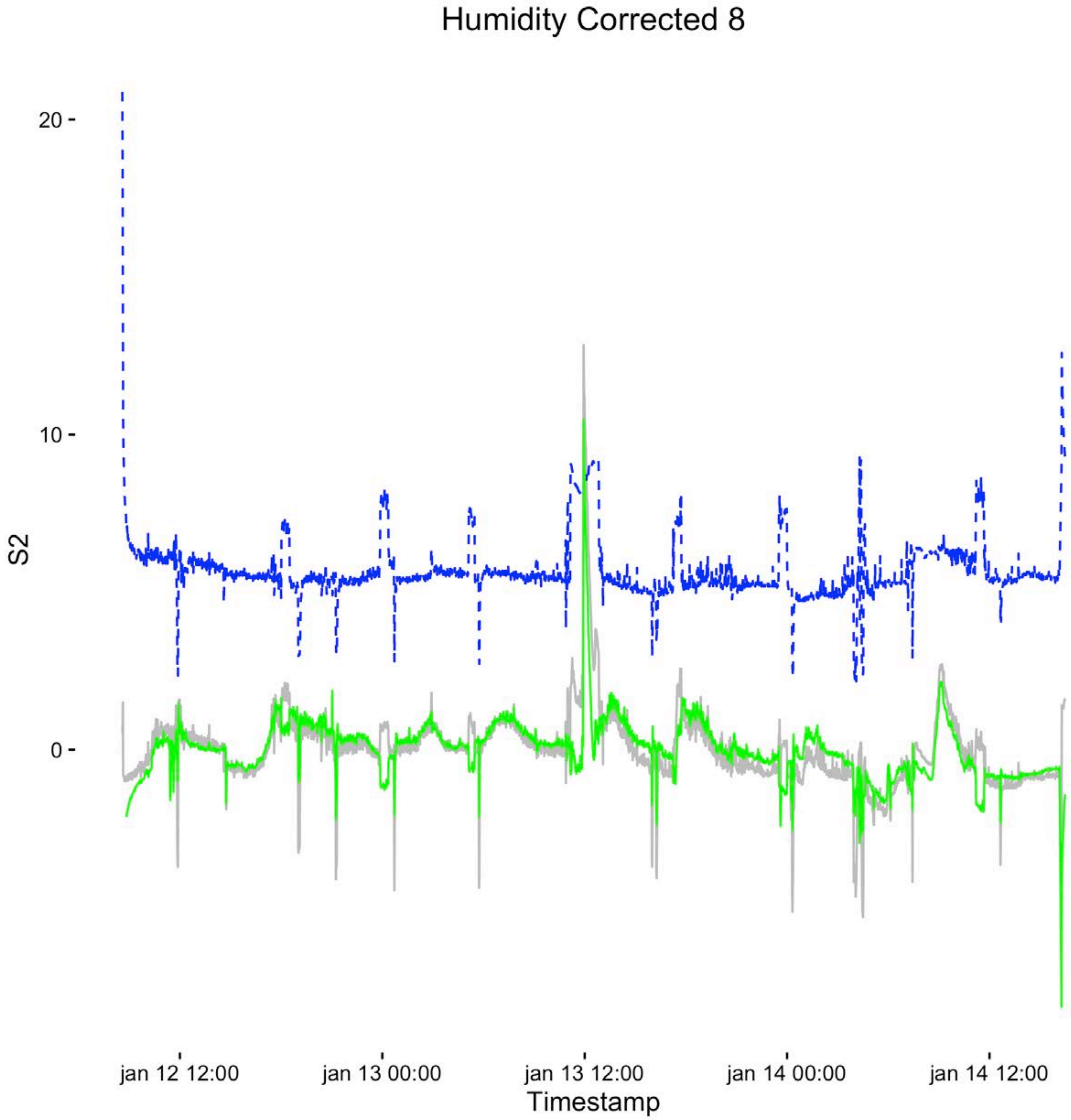

Humidity Corrected 8
S2
20
10
0
jan 12 12:00
jan 13 00:00
jan 13 12:00
jan 14 00:00
jan 14 12:00
Timestamp

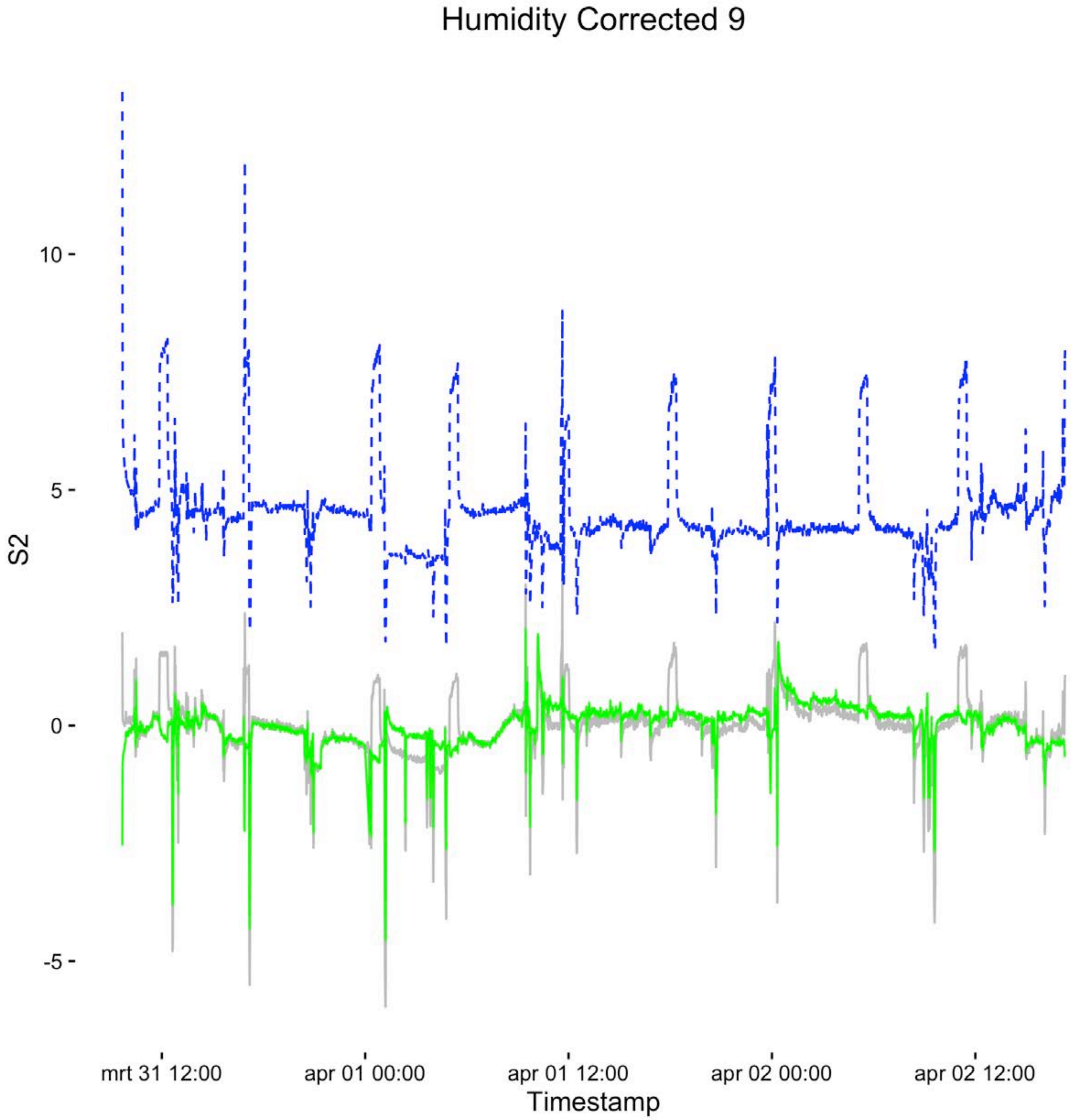
Humidity Corrected 9
S2
10
5
0
-5
mrt 31 12:00
apr 01 00:00
apr 01 12:00
apr 02 00:00
apr 02 12:00
Timestamp

Humidity Corrected 11

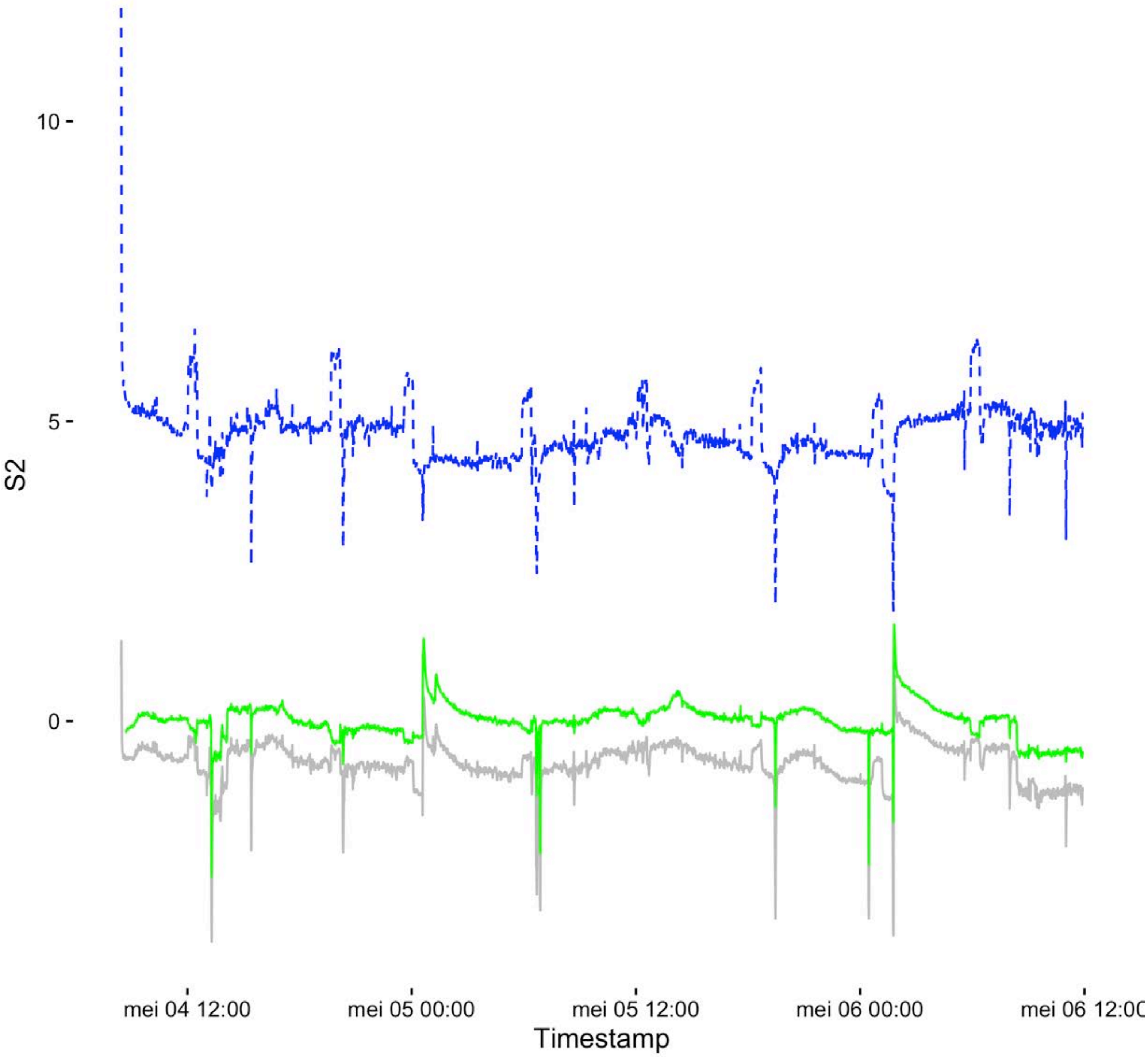

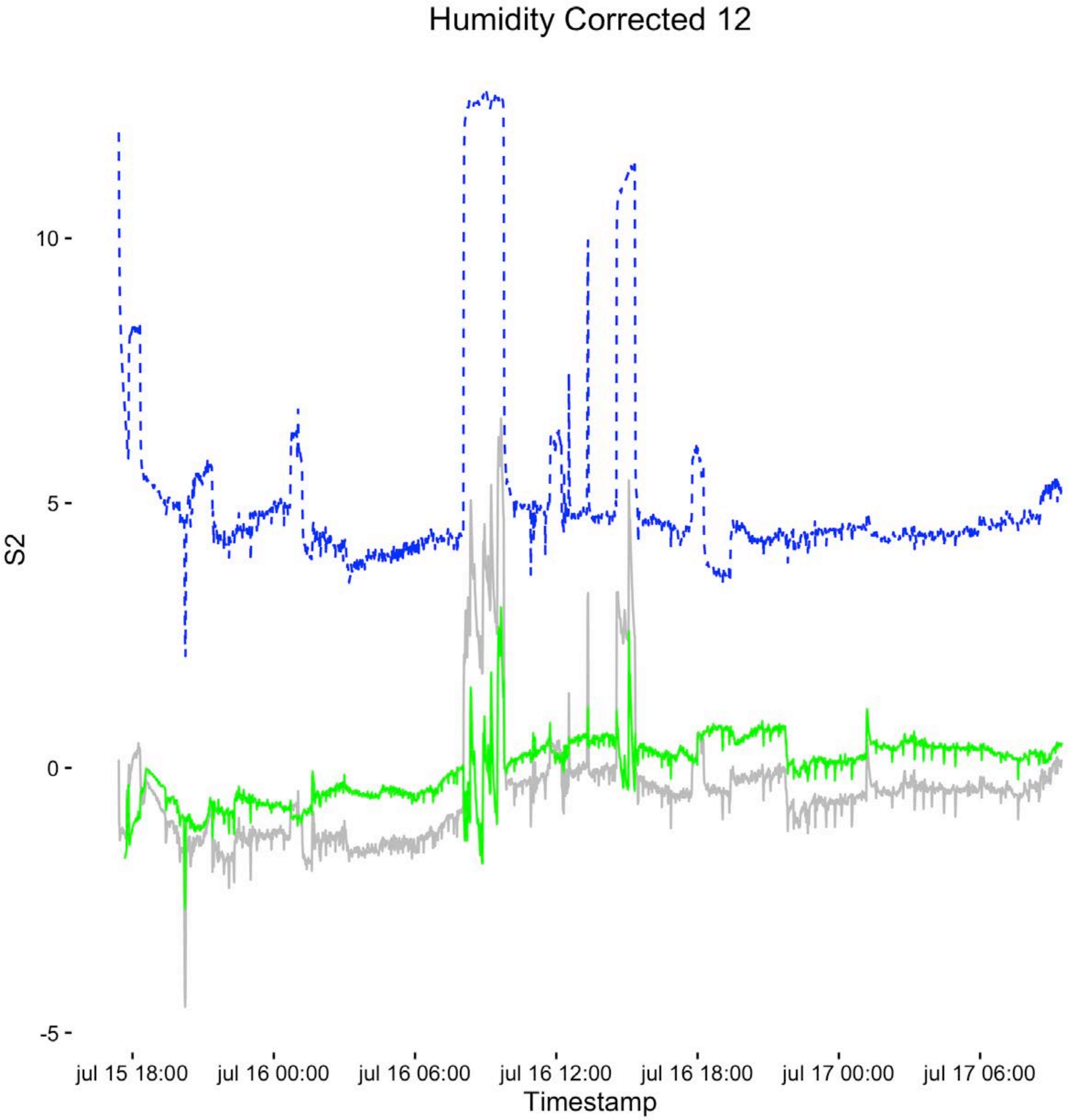
Humidity Corrected 12
10
5
0
-5
S2
jul 15 18:00
jul 16 00:00
jul 16 06:00
jul 16 12:00
jul 16 18:00
jul 17 00:00
jul 17 06:00
Timestamp

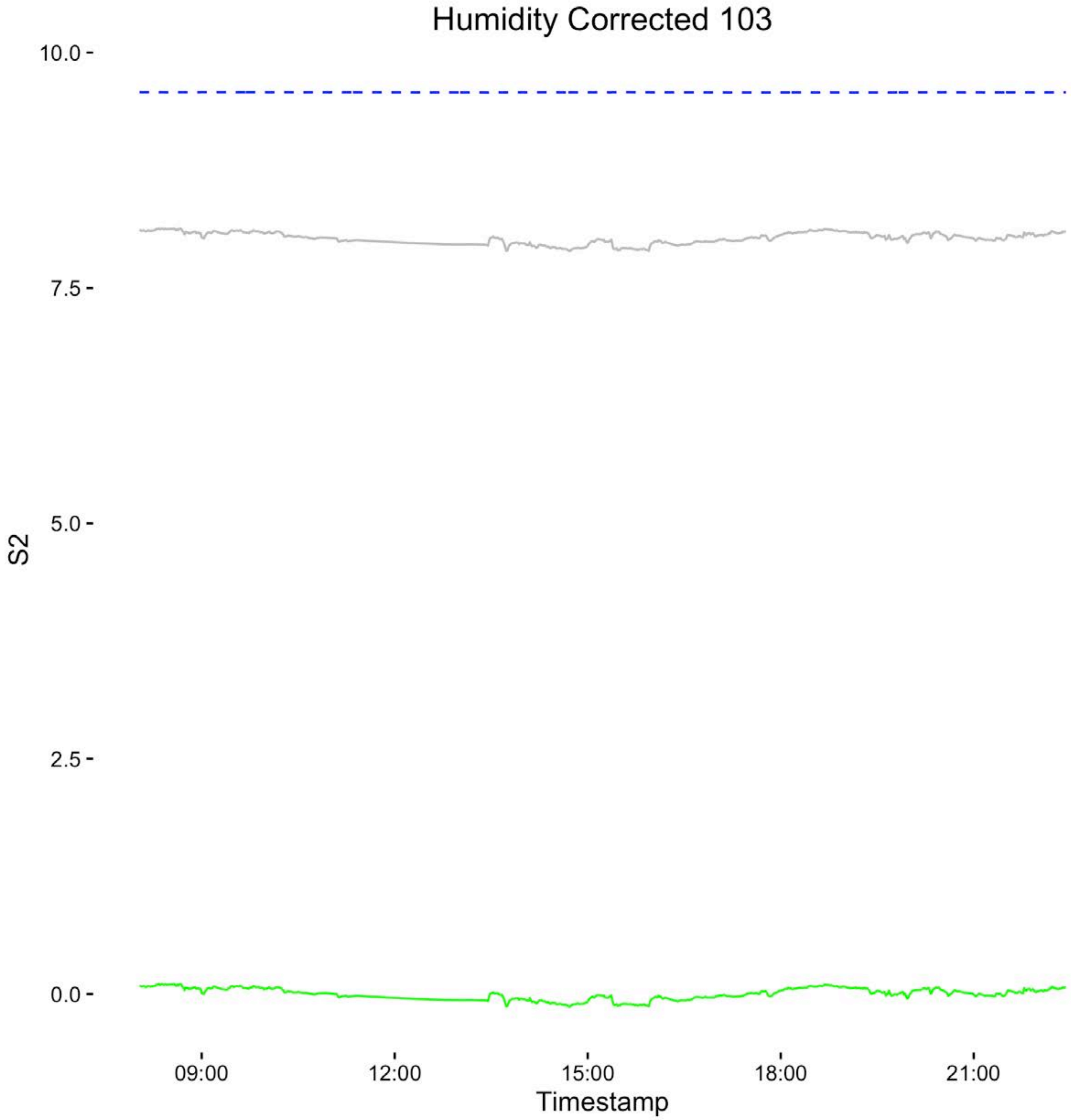

Humidity Corrected 103
10.0
7.5
5.0
2.5
0.0
S2
09:00
12:00
15:00
18:00
21:00
Timestamp

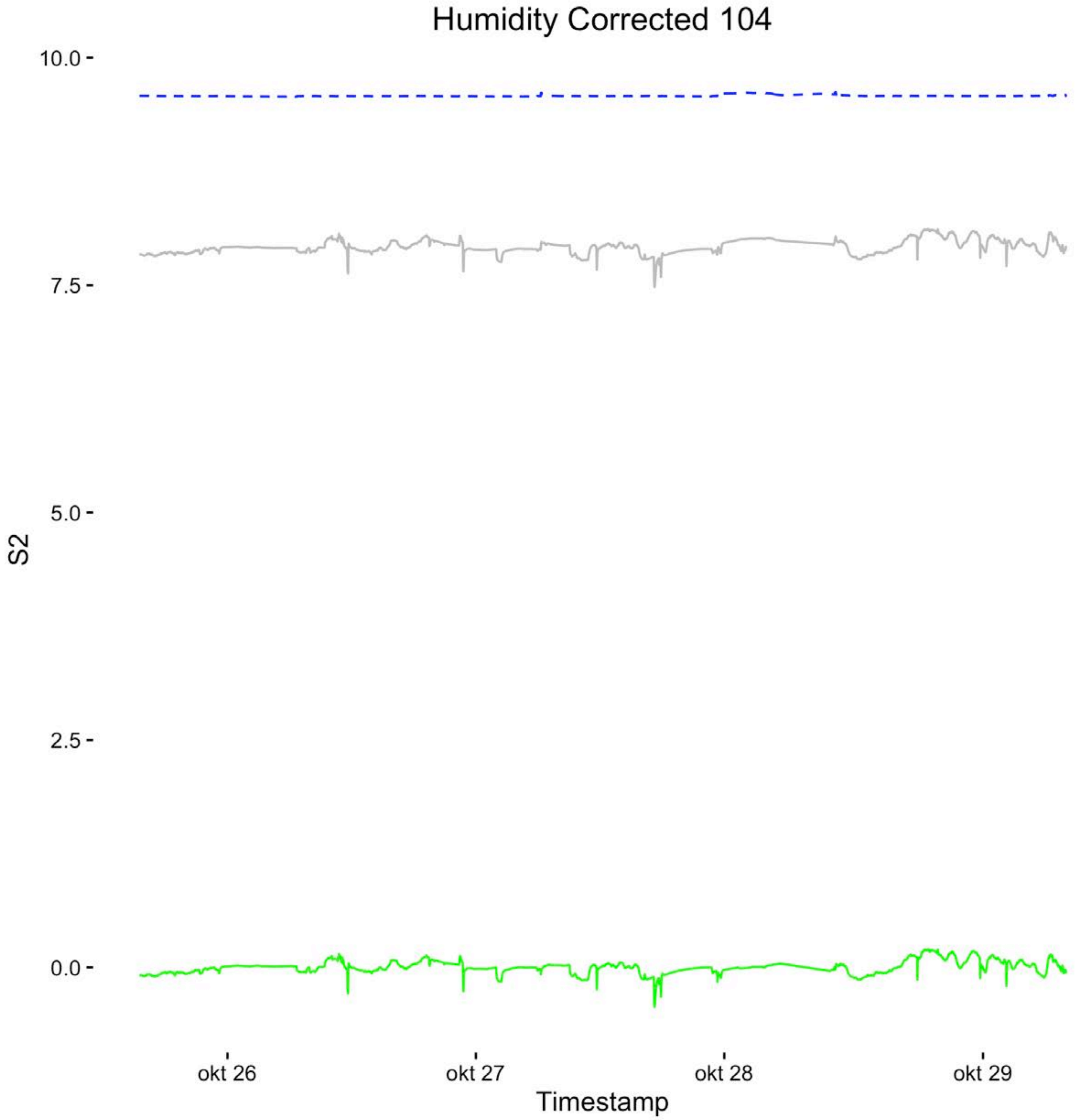

Humidity Corrected 104
10.0
7.5
5.0
2.5
0.0
S2
okt 26
okt 27
okt 28
okt 29
Timestamp

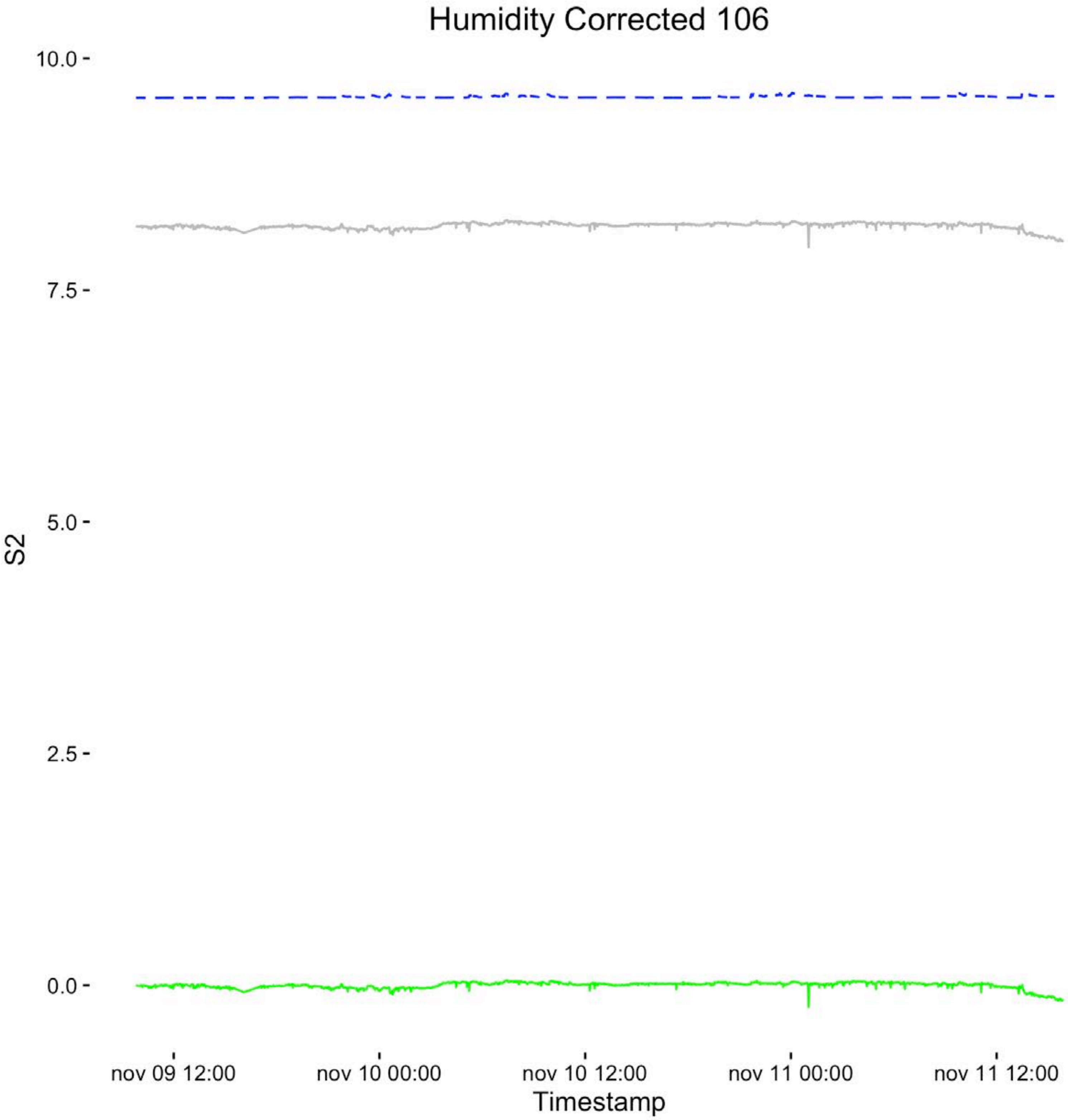

Humidity Corrected 106
10.0
7.5
5.0
2.5
0.0
S2
nov 09 12:00
nov 10 00:00
nov 10 12:00
nov 11 00:00
nov 11 12:00
Timestamp

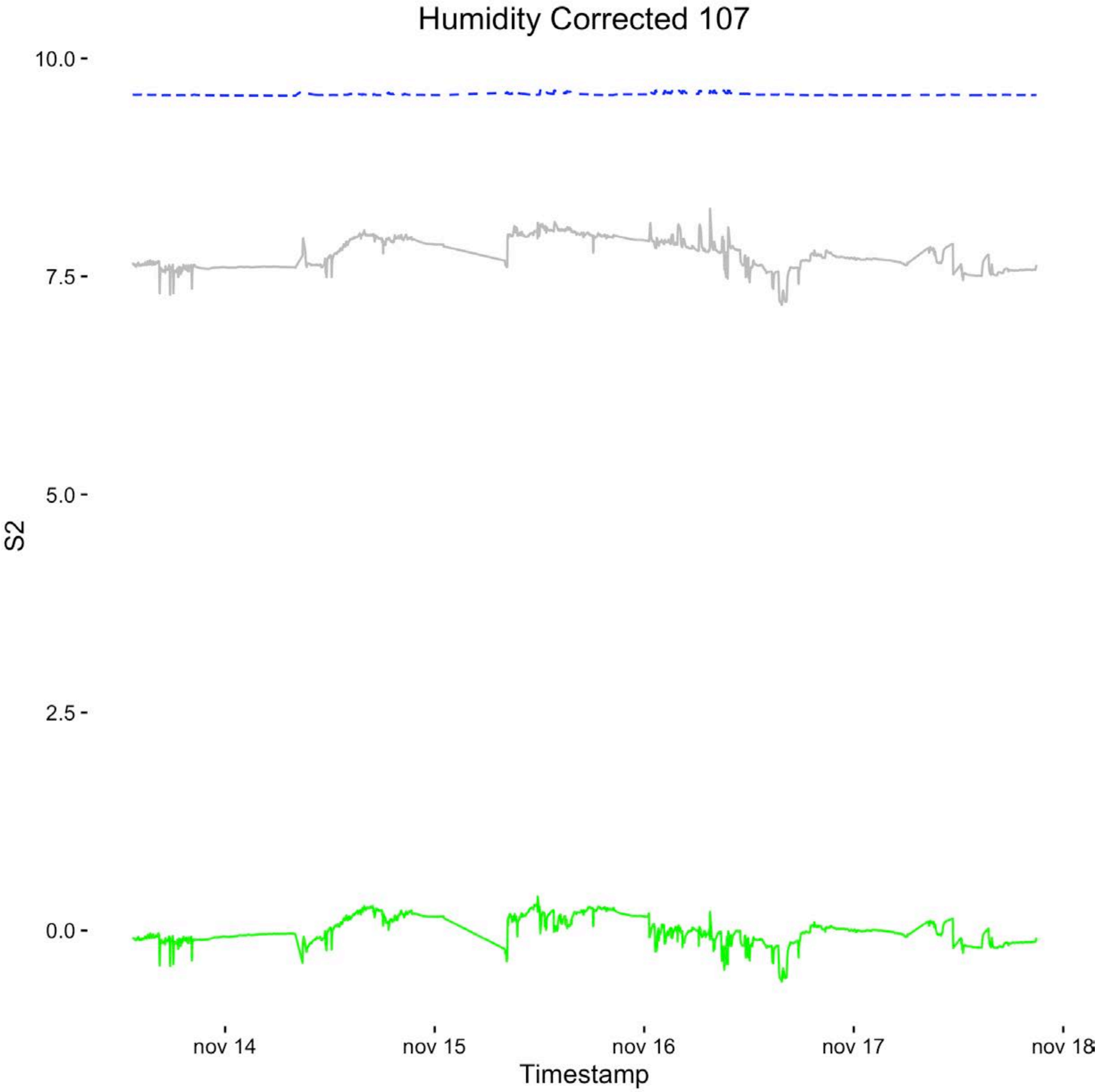
Humidity Corrected 107
10.0
7.5
5.0
2.5
0.0
S2
nov 14
nov 15
nov 16
nov 17
nov 18
Timestamp

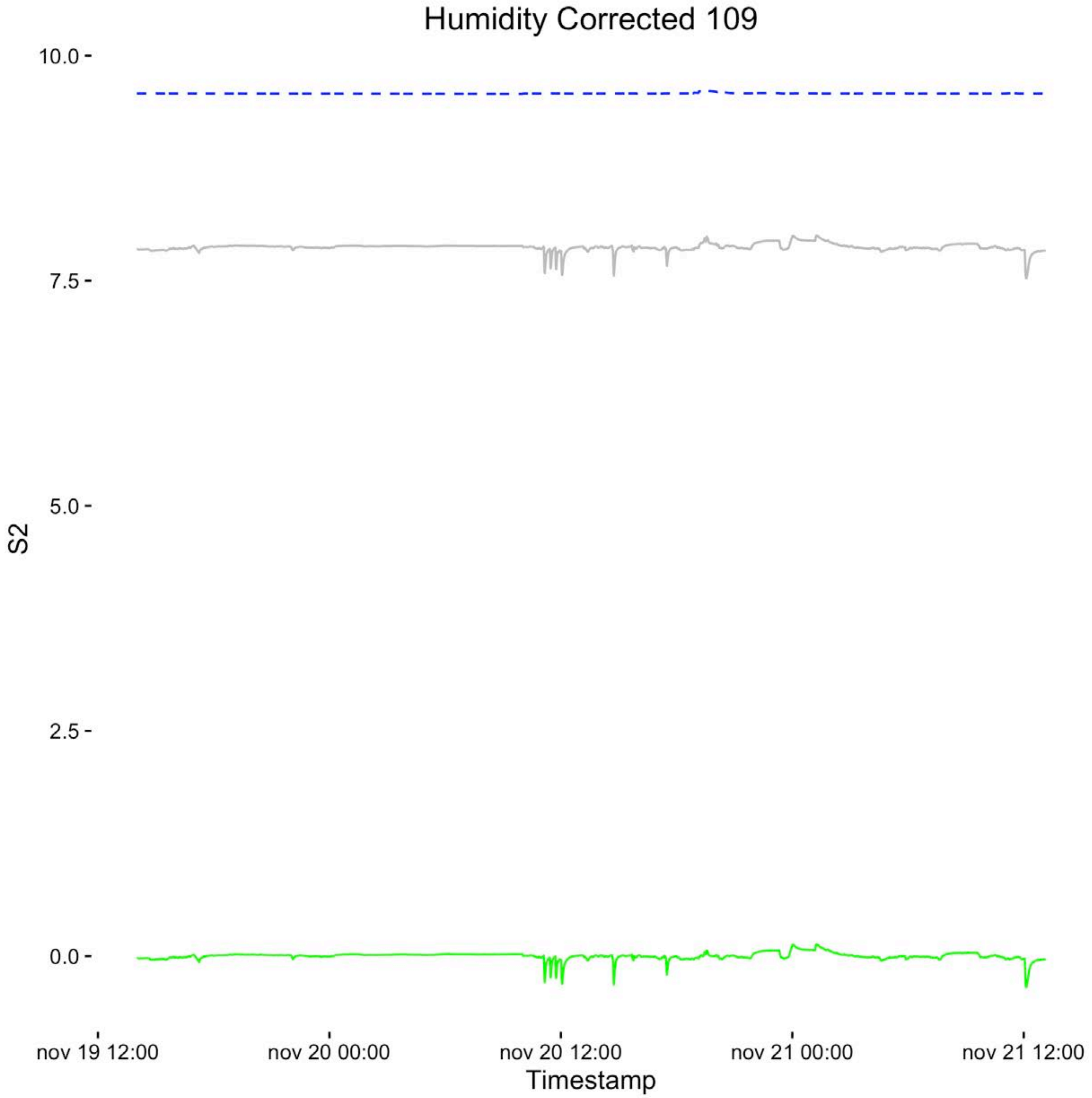

Humidity Corrected 109
10.0
7.5
5.0
2.5
0.0
S2
nov 19 12:00
nov 20 00:00
nov 20 12:00
nov 21 00:00
nov 21 12:00
Timestamp

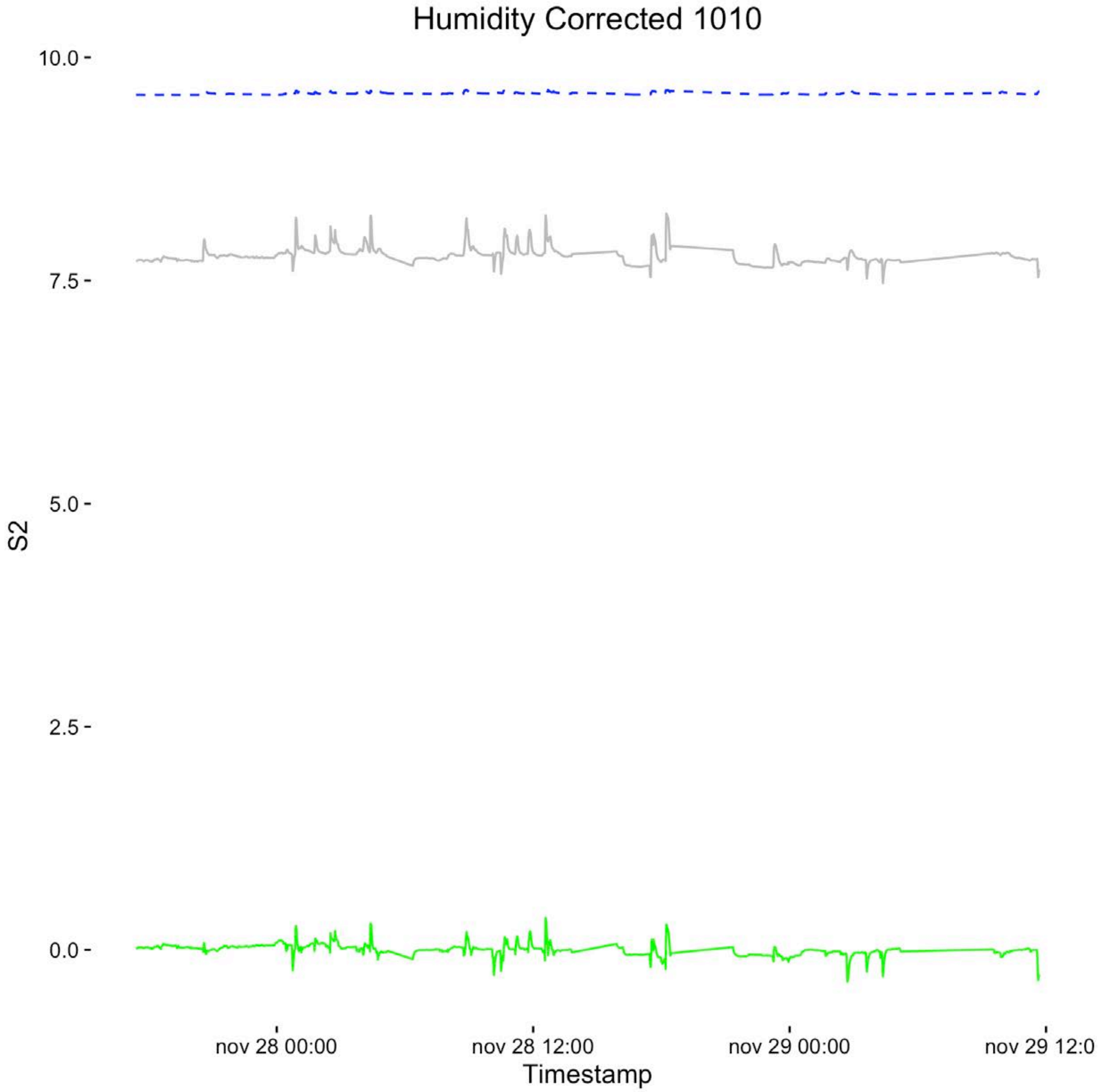
Humidity Corrected 1010
10.0
7.5
5.0
2.5
0.0
S2
nov 28 00:00
nov 28 12:00
nov 29 00:00
nov 29 12:0
Timestamp

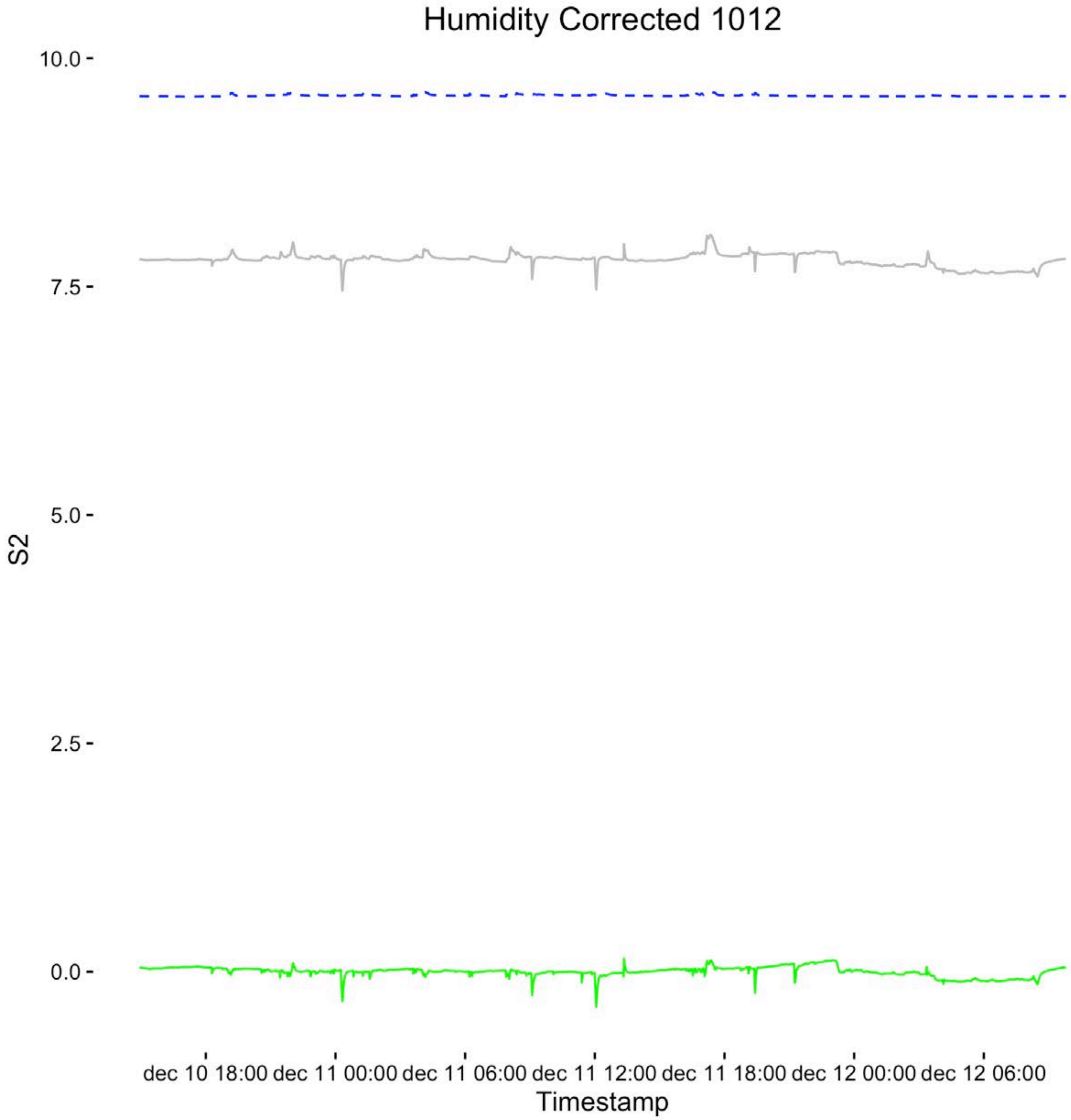
Humidity Corrected 1012
10.0
7.5
5.0
2.5
0.0
S2
dec 10 18:00
dec 11 00:00
dec 11 06:00
dec 11 12:00
dec 11 18:00
dec 12 00:00
dec 12 06:00
Timestamp

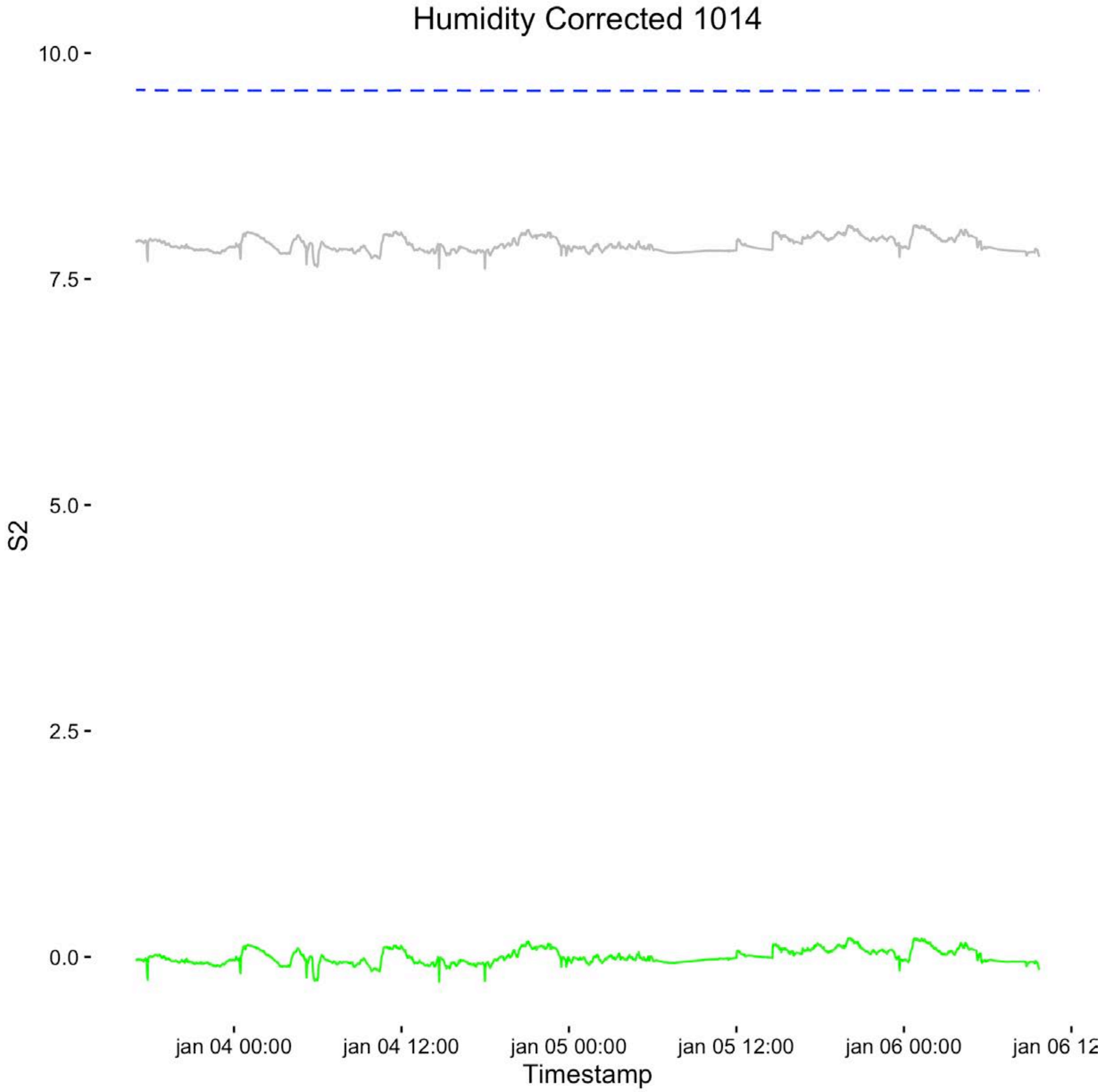
Humidity Corrected 1014
10.0
7.5
5.0
2.5
0.0
S2
jan 04 00:00
jan 04 12:00
jan 05 00:00
jan 05 12:00
jan 06 00:00
jan 06 12
Timestamp

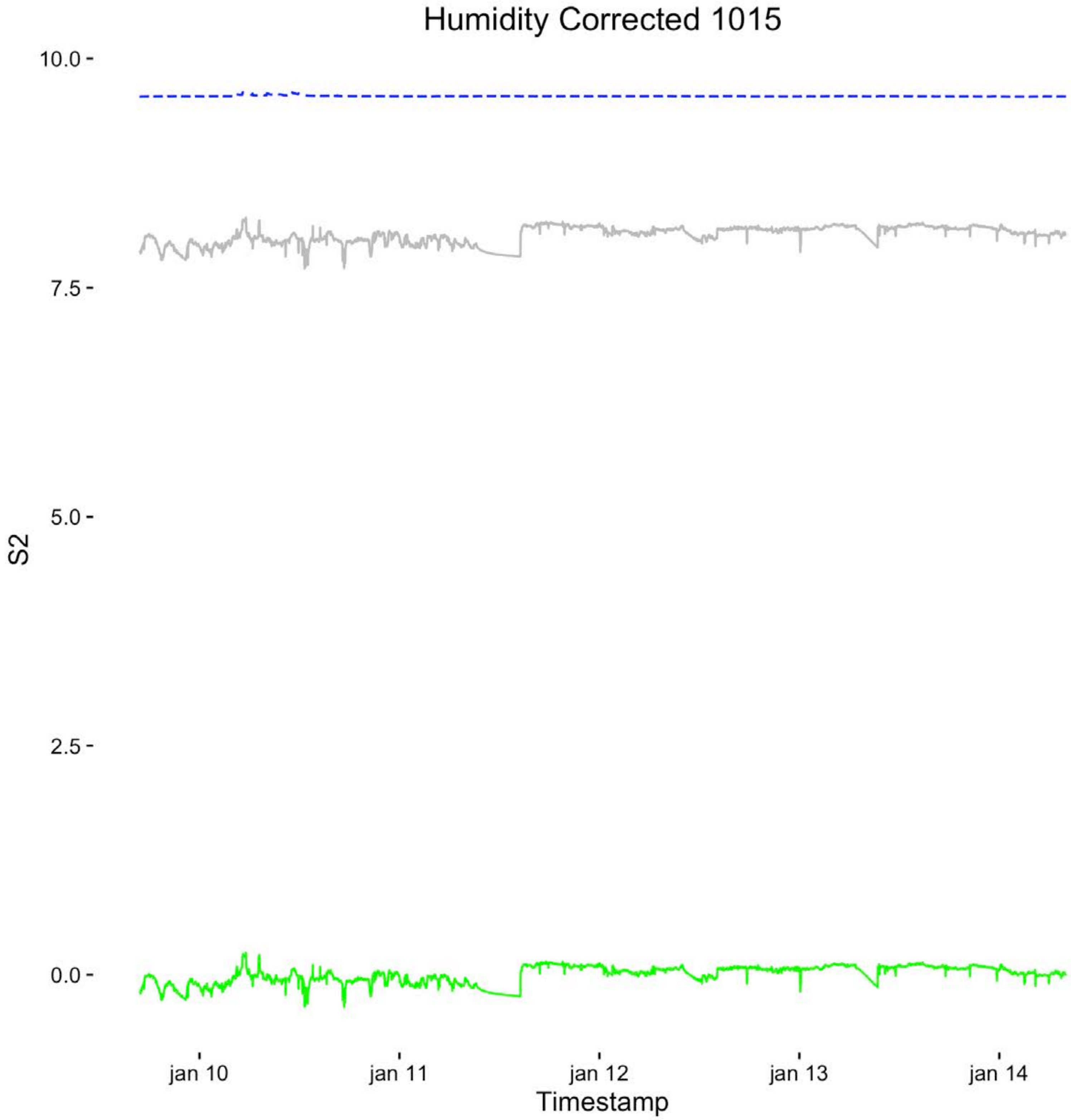

Humidity Corrected 1015
10.0
7.5
5.0
2.5
0.0
S2
jan 10
jan 11
jan 12
jan 13
jan 14
Timestamp

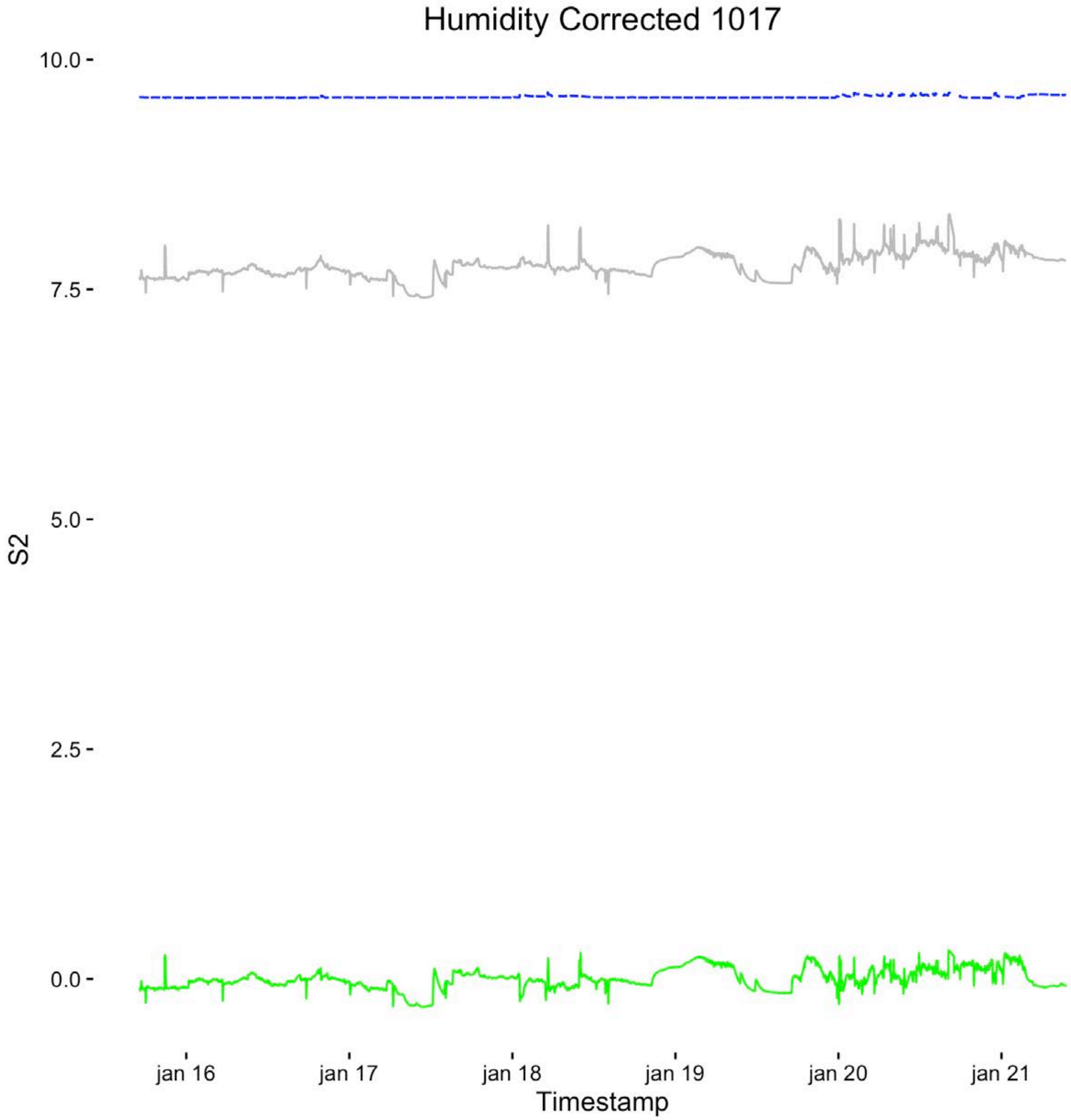
Humidity Corrected 1017
10.0
7.5
5.0
2.5
0.0
S2
jan 16
jan 17
jan 18
jan 19
jan 20
jan 21
Timestamp

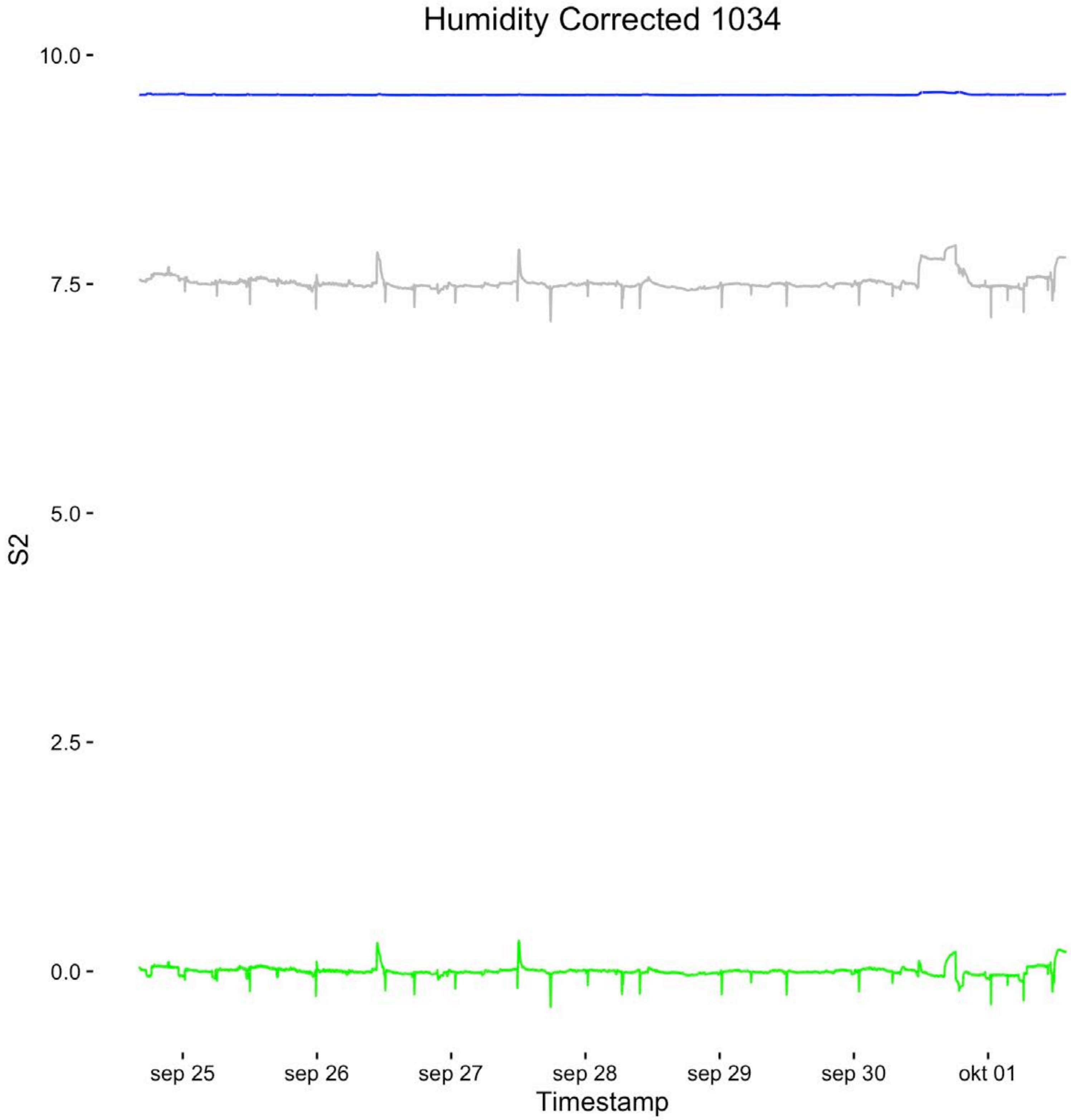
Humidity Corrected 1034
10.0
7.5
5.0
2.5
0.0
S2
sep 25
sep 26
sep 27
sep 28
sep 29
sep 30
okt 01
Timestamp

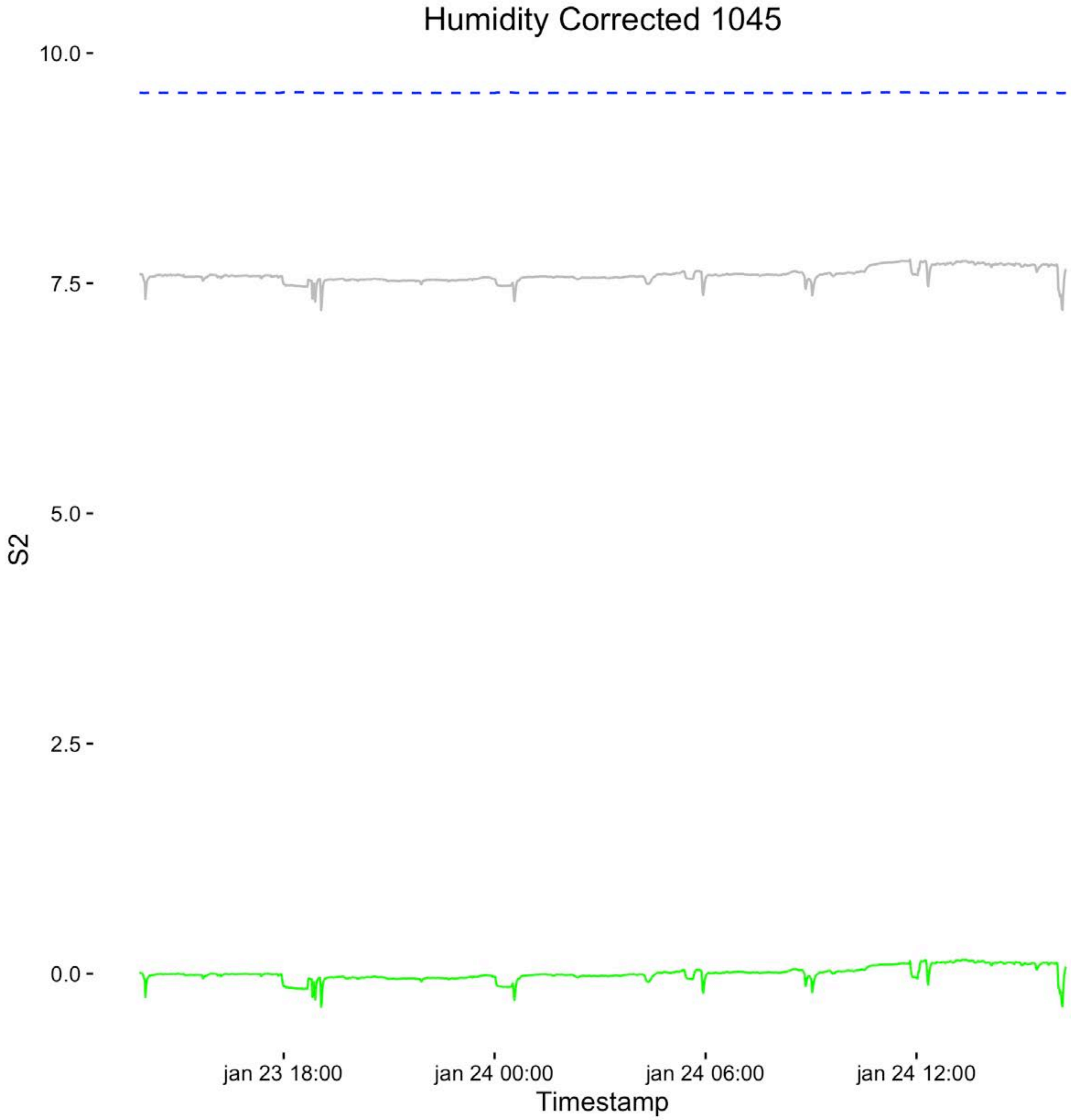
Humidity Corrected 1045
10.0
7.5
5.0
2.5
0.0
S2
jan 23 18:00
jan 24 00:00
jan 24 06:00
jan 24 12:00
Timestamp

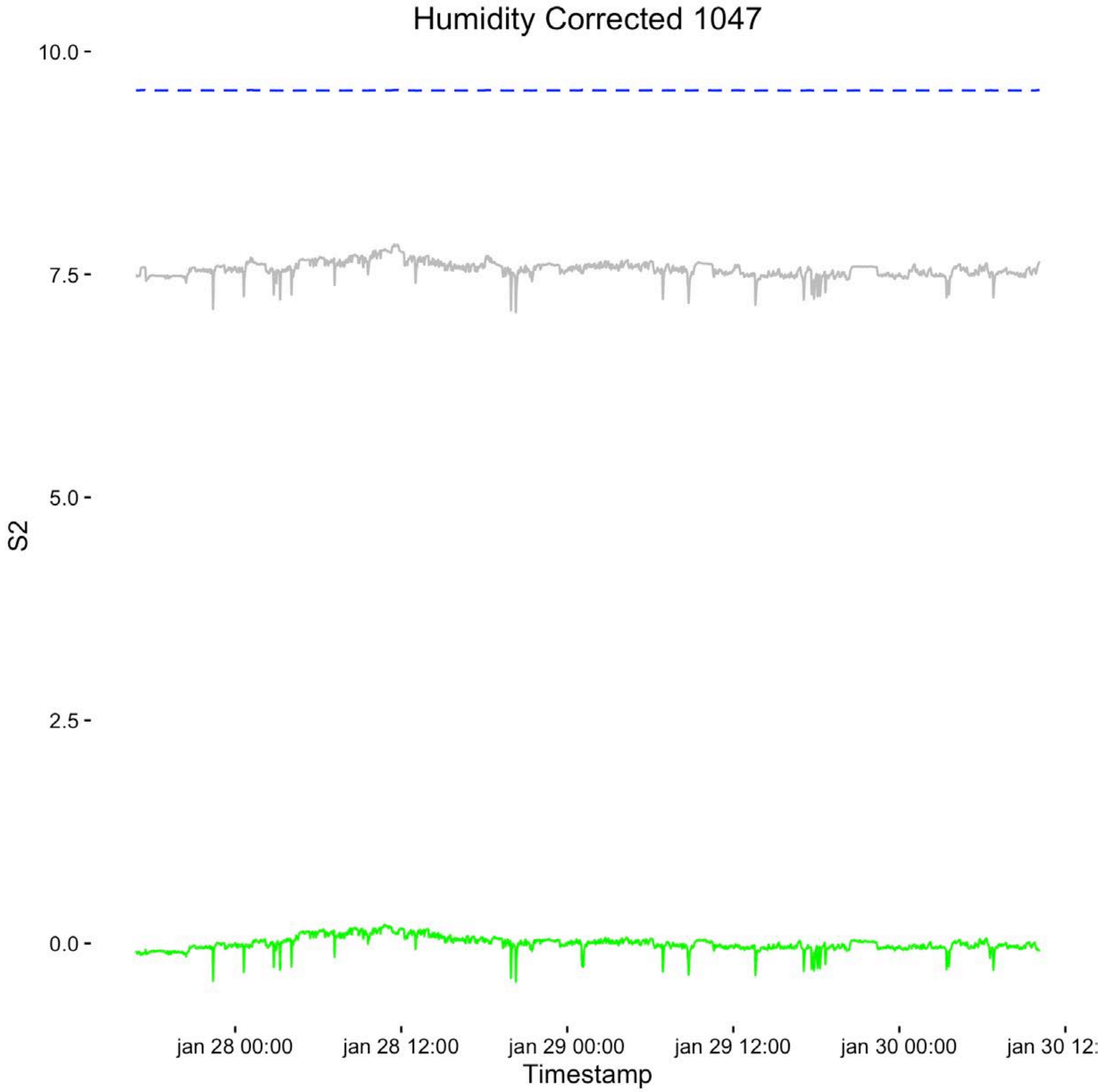
Humidity Corrected 1047
10.0
7.5
5.0
2.5
0.0
S2
jan 28 00:00
jan 28 12:00
jan 29 00:00
jan 29 12:00
jan 30 00:00
Timestamp

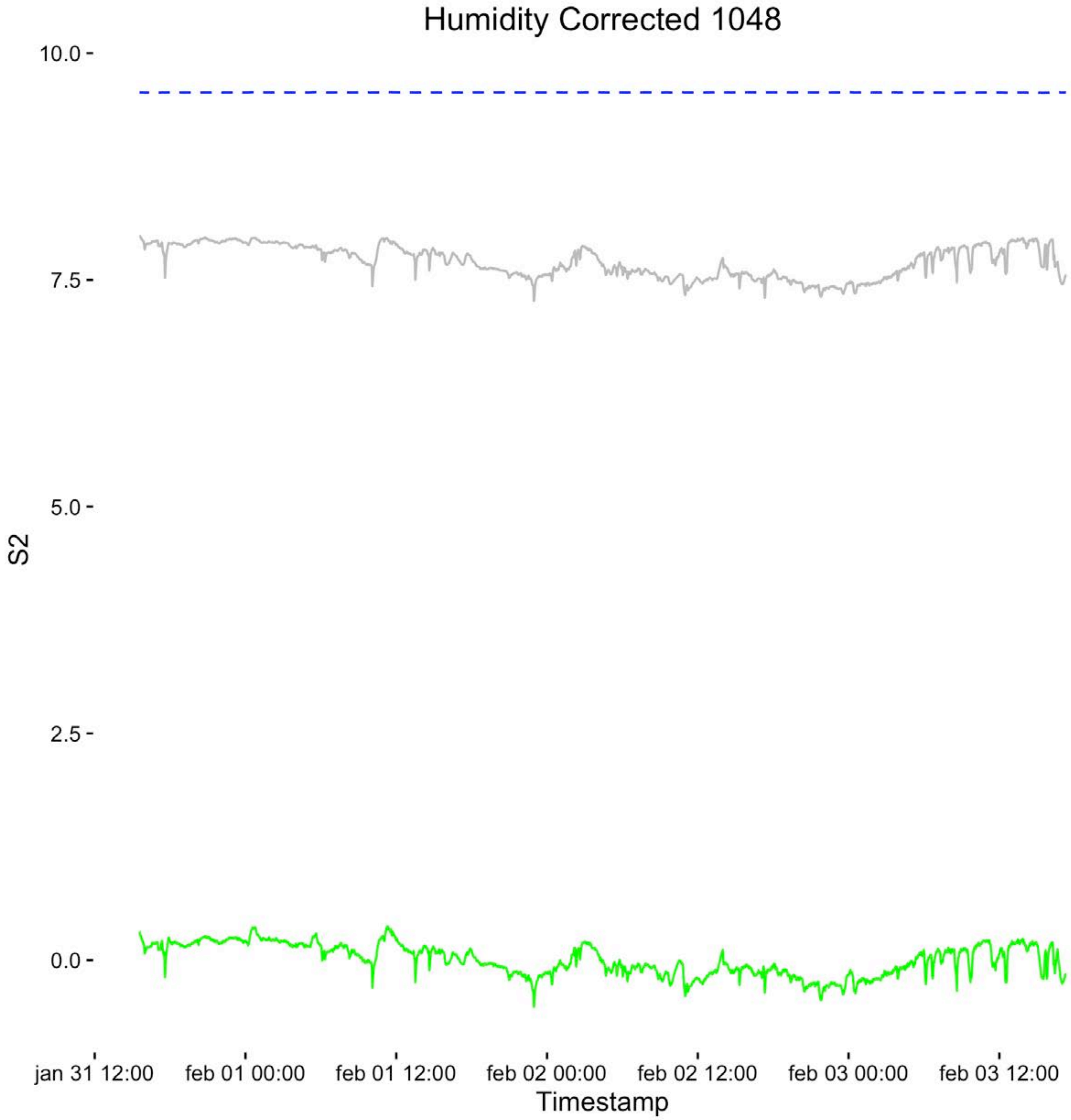
Humidity Corrected 1048
10.0
7.5
5.0
2.5
0.0
S2
jan 31 12:00
feb 01 00:00
feb 01 12:00
feb 02 00:00
feb 02 12:00
feb 03 00:00
feb 03 12:00
Timestamp

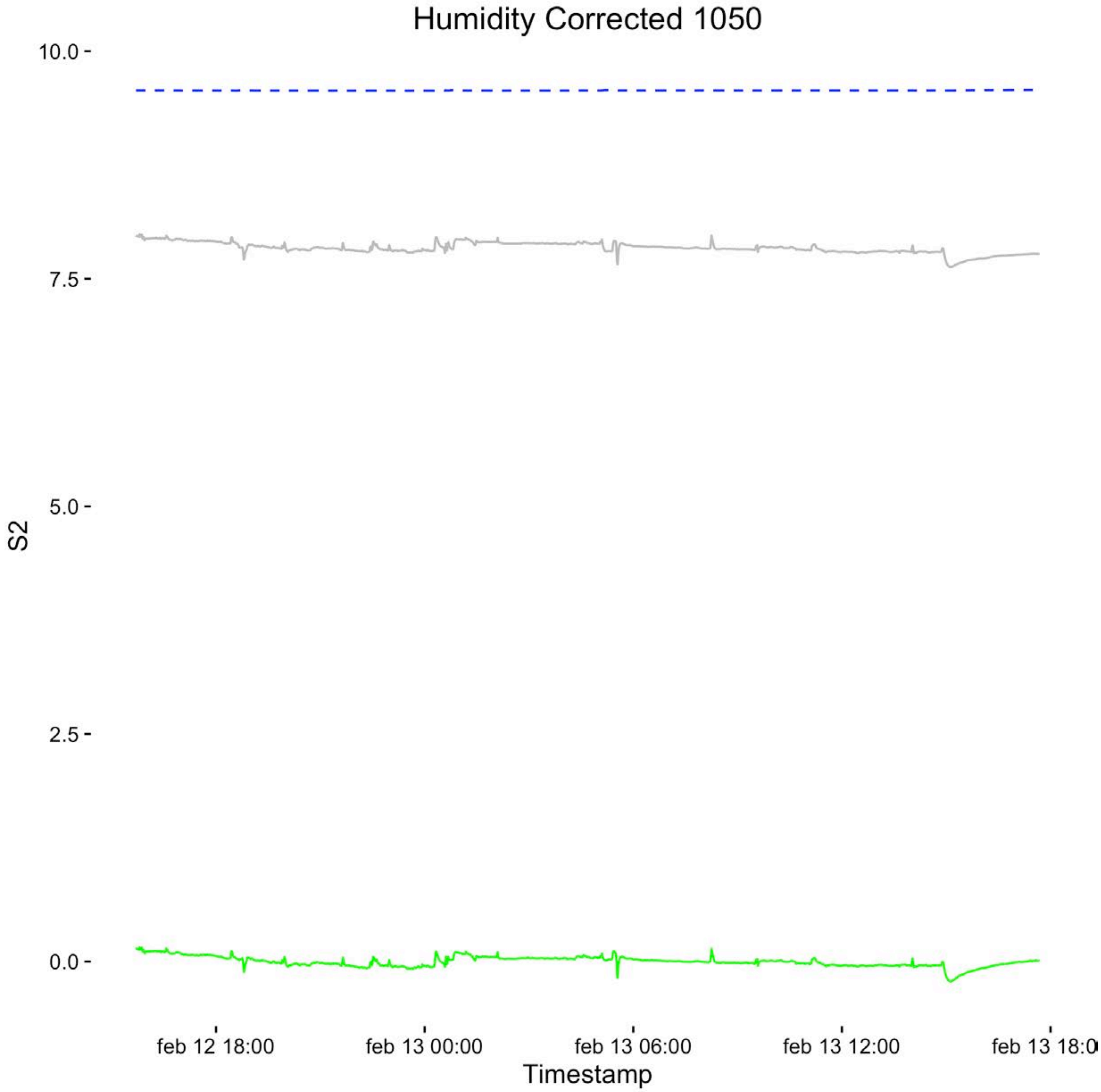

Humidity Corrected 1050
10.0
7.5
5.0
2.5
0.0
S2
feb 12 18:00
feb 13 00:00
feb 13 06:00
feb 13 12:00
feb 13 18:0
Timestamp

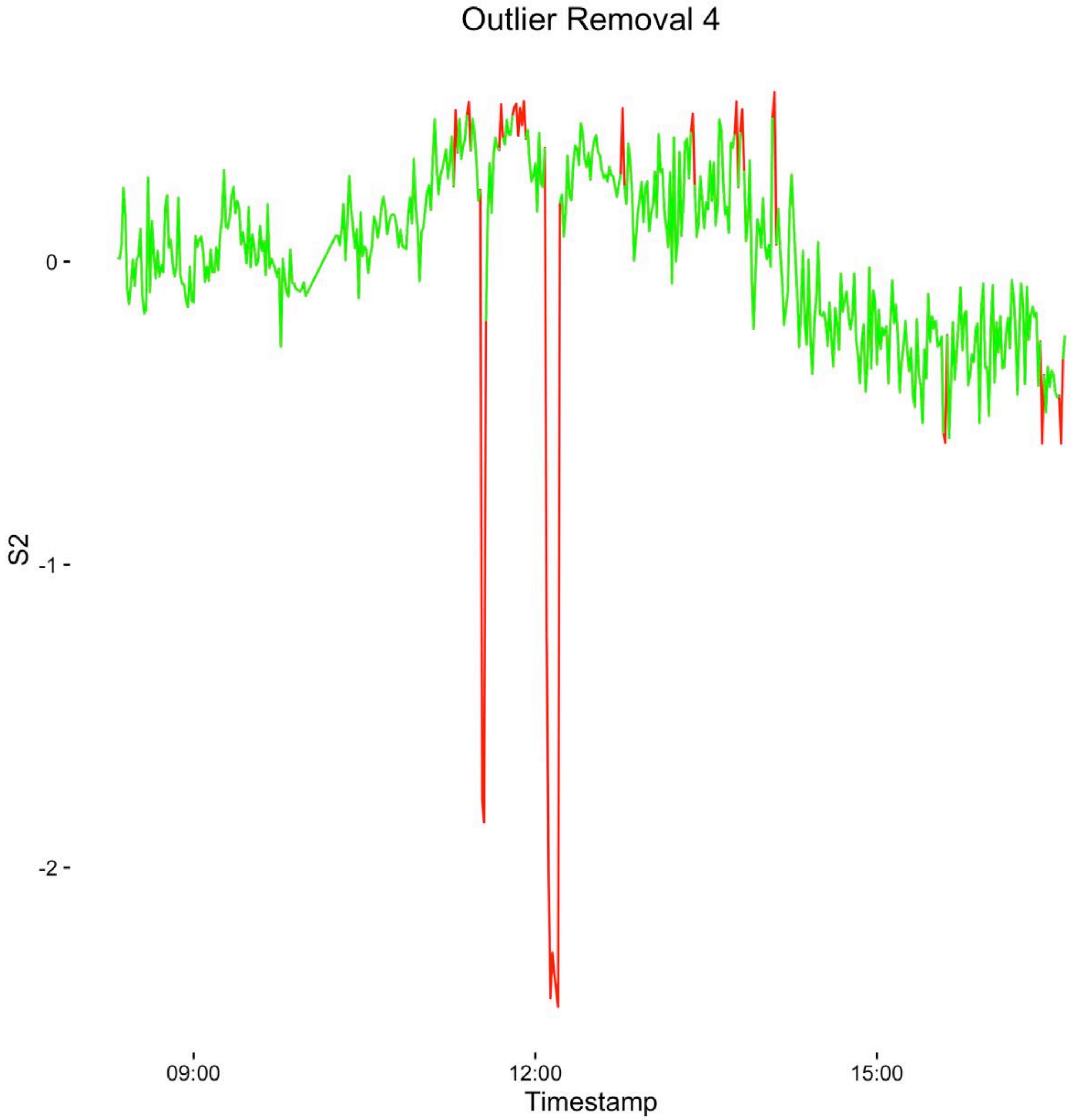
Outlier Removal 4
S2
0
-1
-2
09:00
12:00
15:00
Timestamp

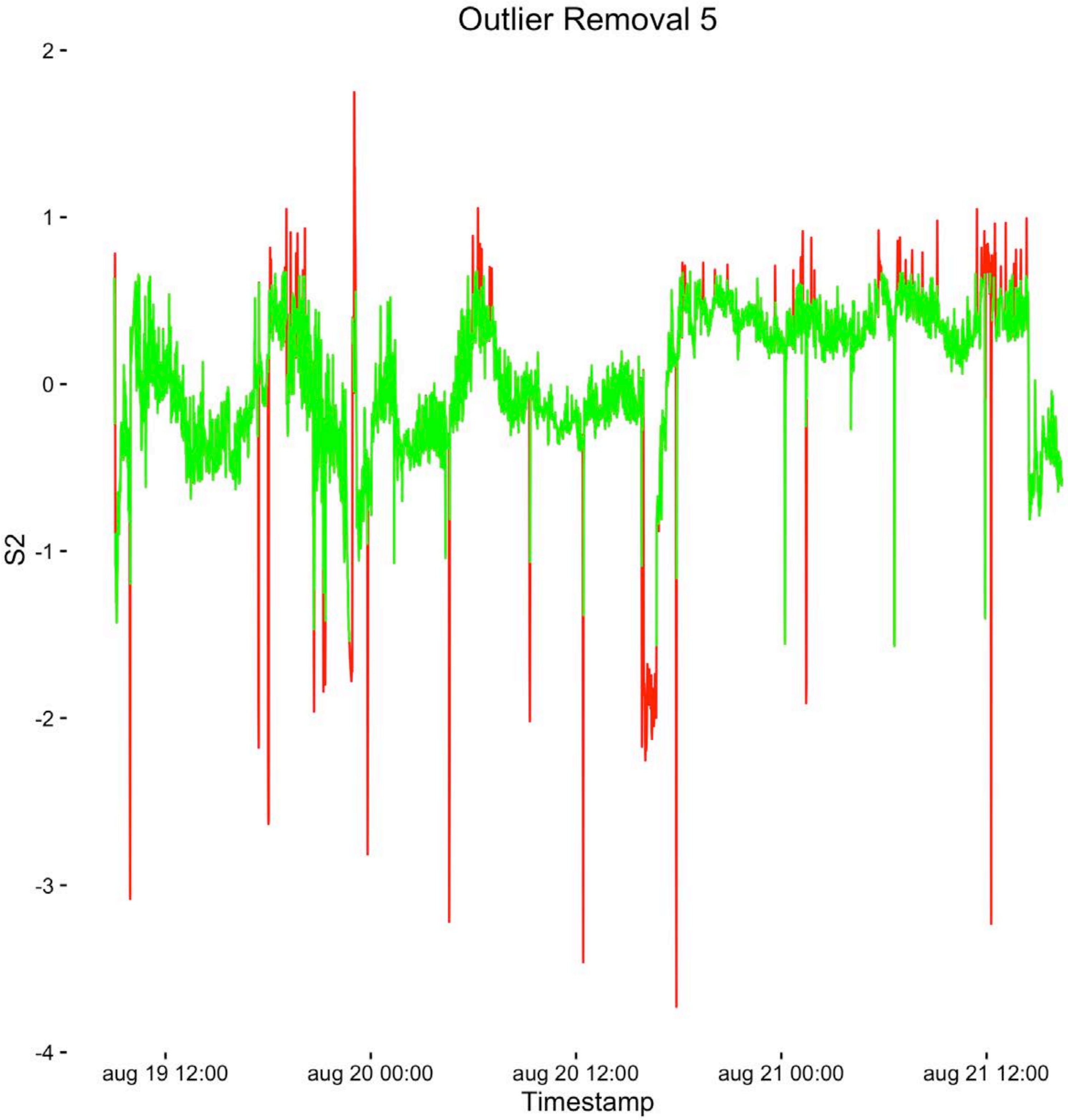

Outlier Removal 5
2
1
0
-1
-2
-3
-4
S2
aug 19 12:00
aug 20 00:00
aug 20 12:00
aug 21 00:00
aug 21 12:00
Timestamp

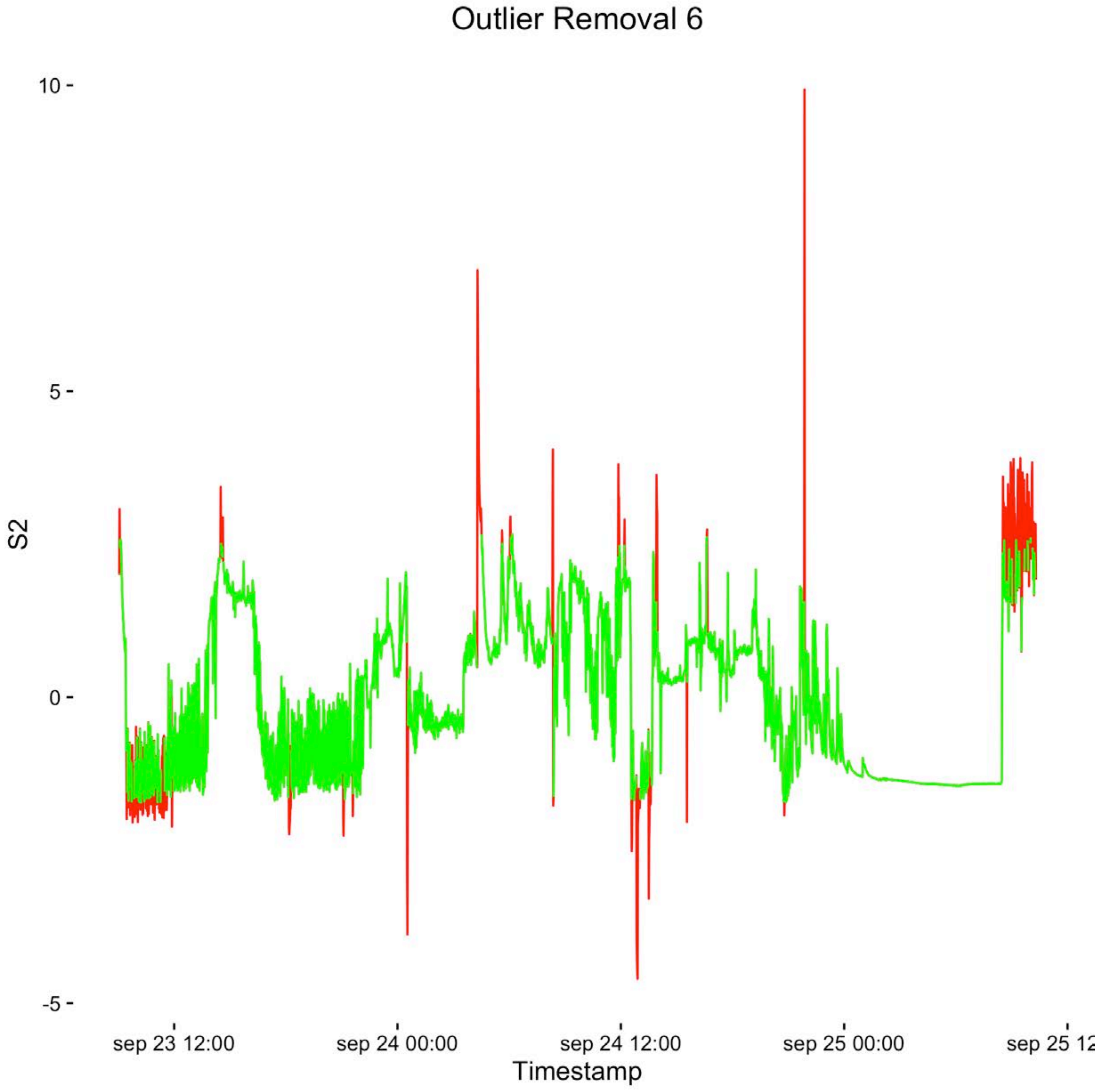

Outlier Removal 6
10
5
0
-5
S2
sep 23 12:00
sep 24 00:00
sep 24 12:00
sep 25 00:00
sep 25 12
Timestamp

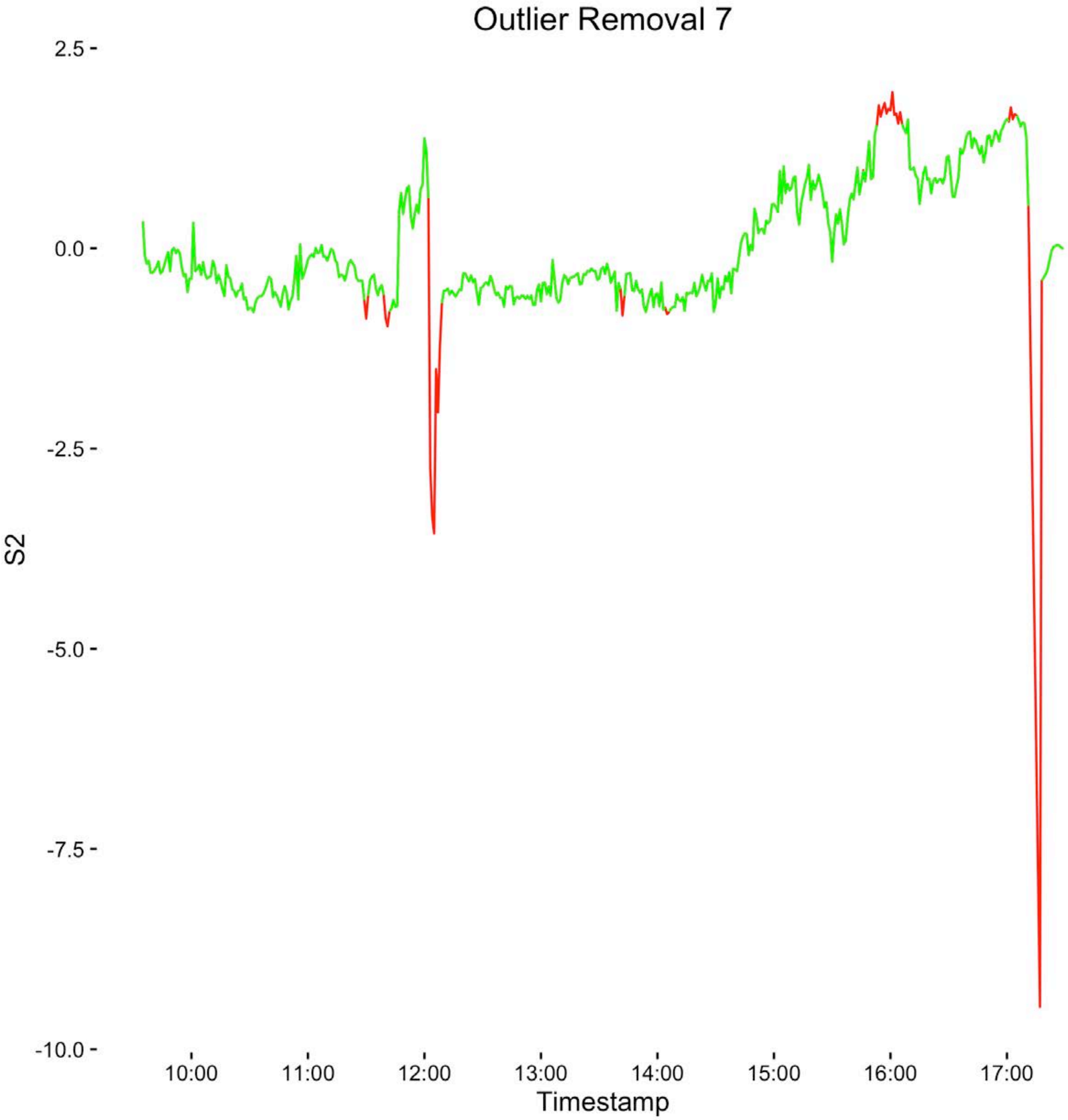
Outlier Removal 7
2.5
0.0
-2.5
-5.0
-7.5
-10.0
S2
10:00
11:00
12:00
13:00
14:00
15:00
16:00
17:00
Timestamp

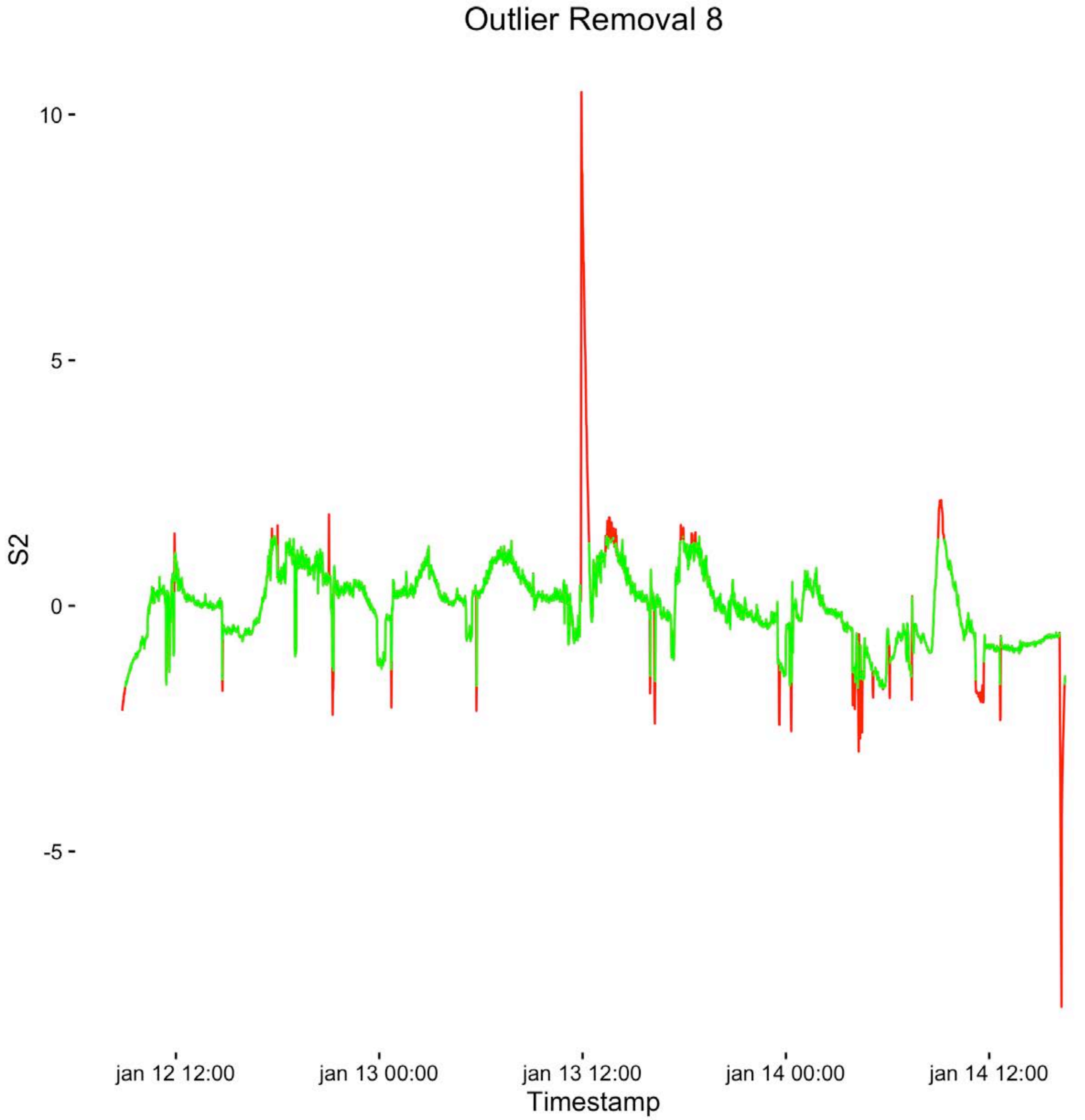

Outlier Removal 8
10
5
0
-5
S2
jan 12 12:00
jan 13 00:00
jan 13 12:00
jan 14 00:00
jan 14 12:00
Timestamp

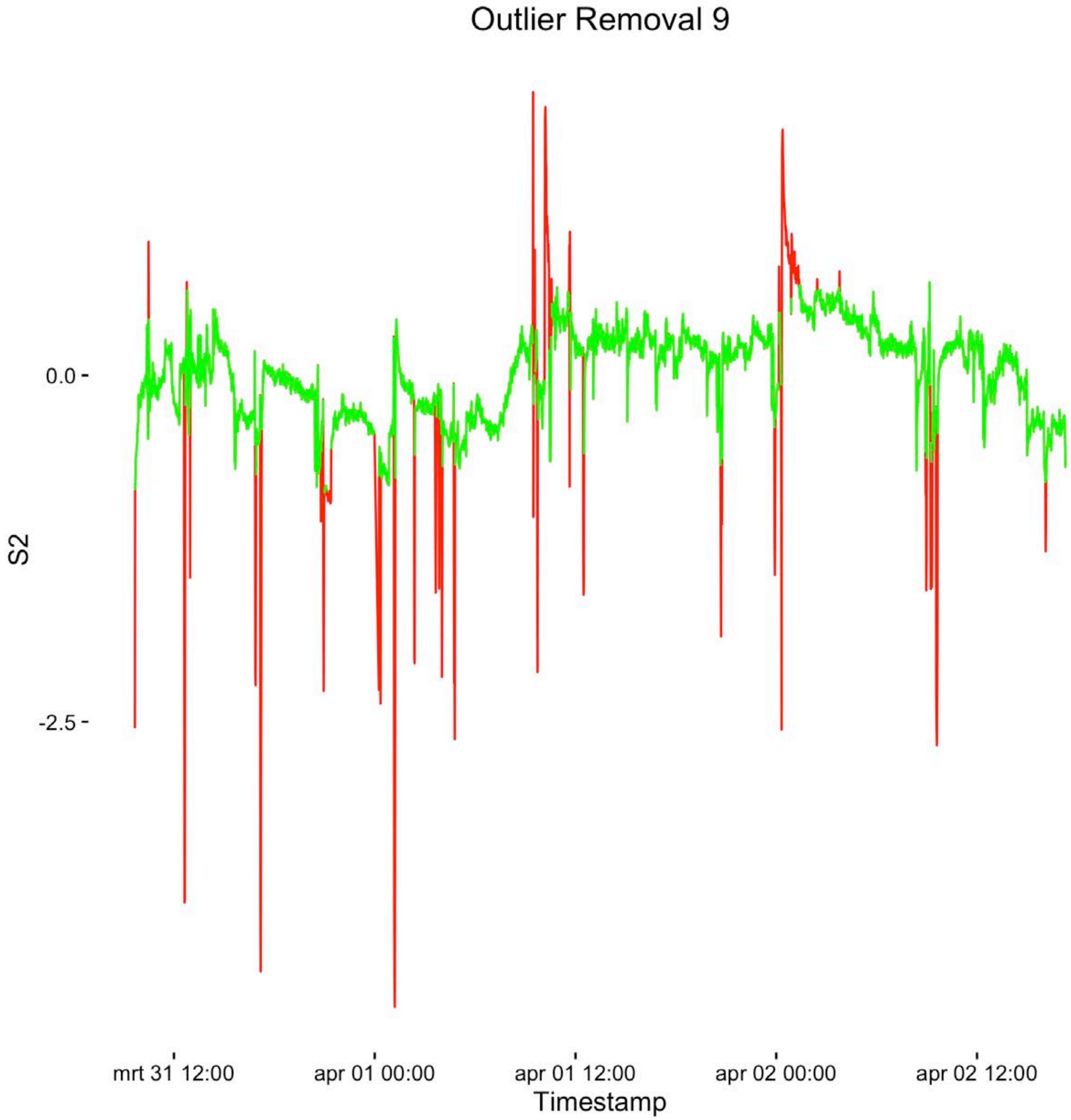

Outlier Removal 9
S2
0.0
-2.5
mrt 31 12:00
apr 01 00:00
apr 01 12:00
apr 02 00:00
apr 02 12:00
Timestamp

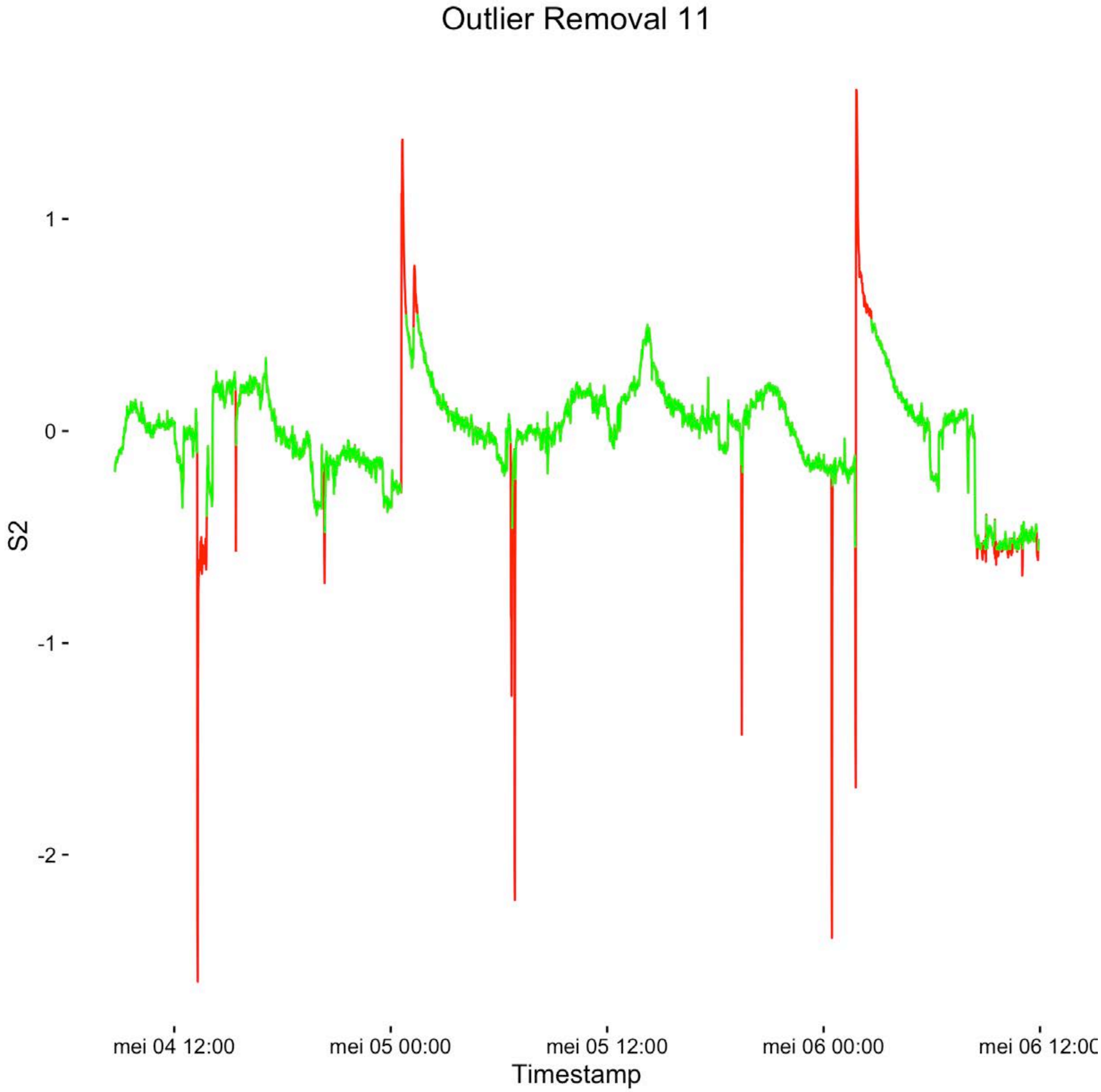
Outlier Removal 11
S2
1
0
-1
-2
mei 04 12:00
mei 05 00:00
mei 05 12:00
mei 06 00:00
mei 06 12:00
Timestamp

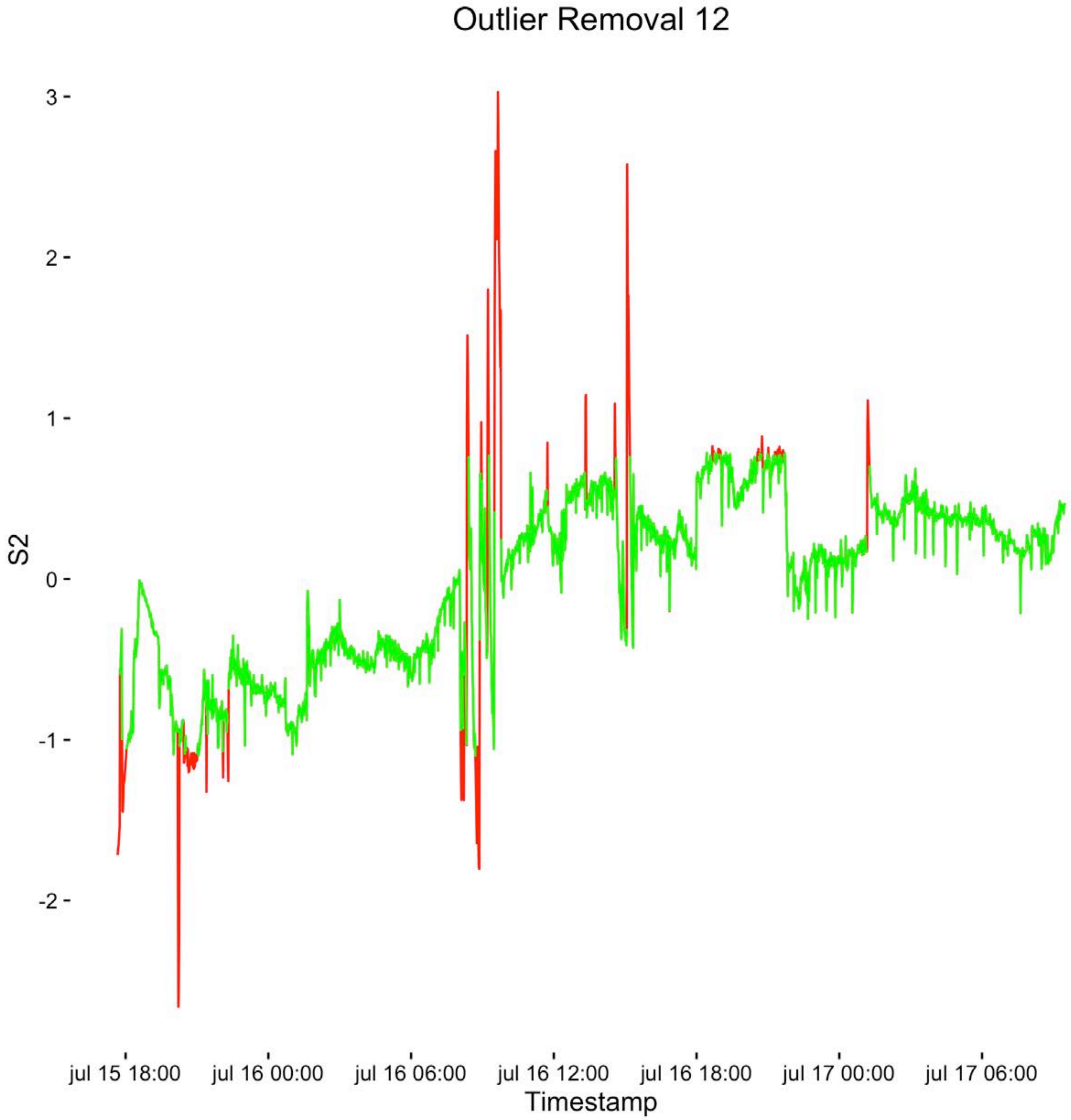
Outlier Removal 12
S2
Timestamp
3
2
1
0
-1
-2
jul 15 18:00
jul 16 00:00
jul 16 06:00
jul 16 12:00
jul 16 18:00
jul 17 00:00
jul 17 06:00

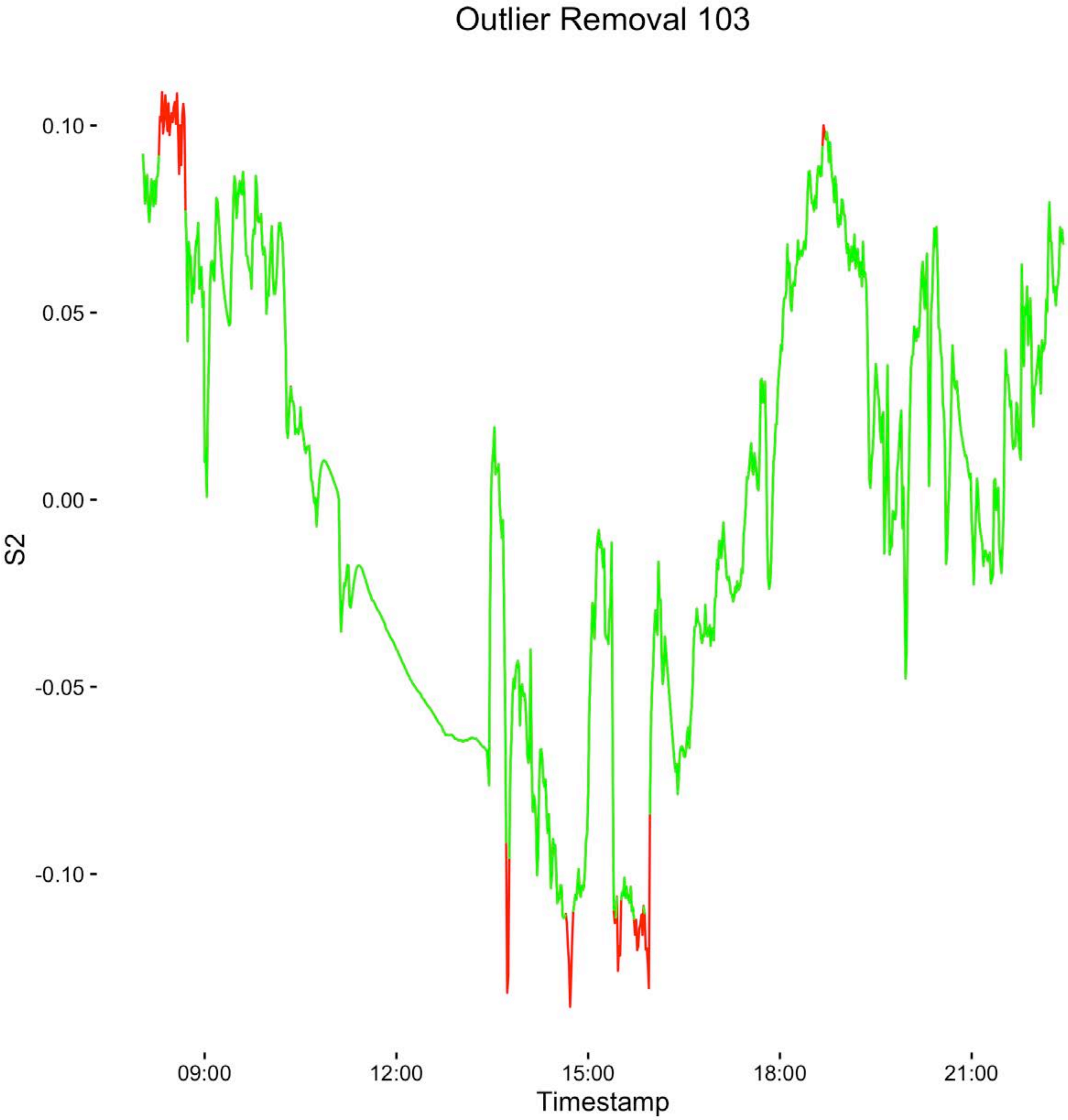
Outlier Removal 103
0.10
0.05
0.00
-0.05
-0.10
S2
09:00
12:00
15:00
18:00
21:00
Timestamp

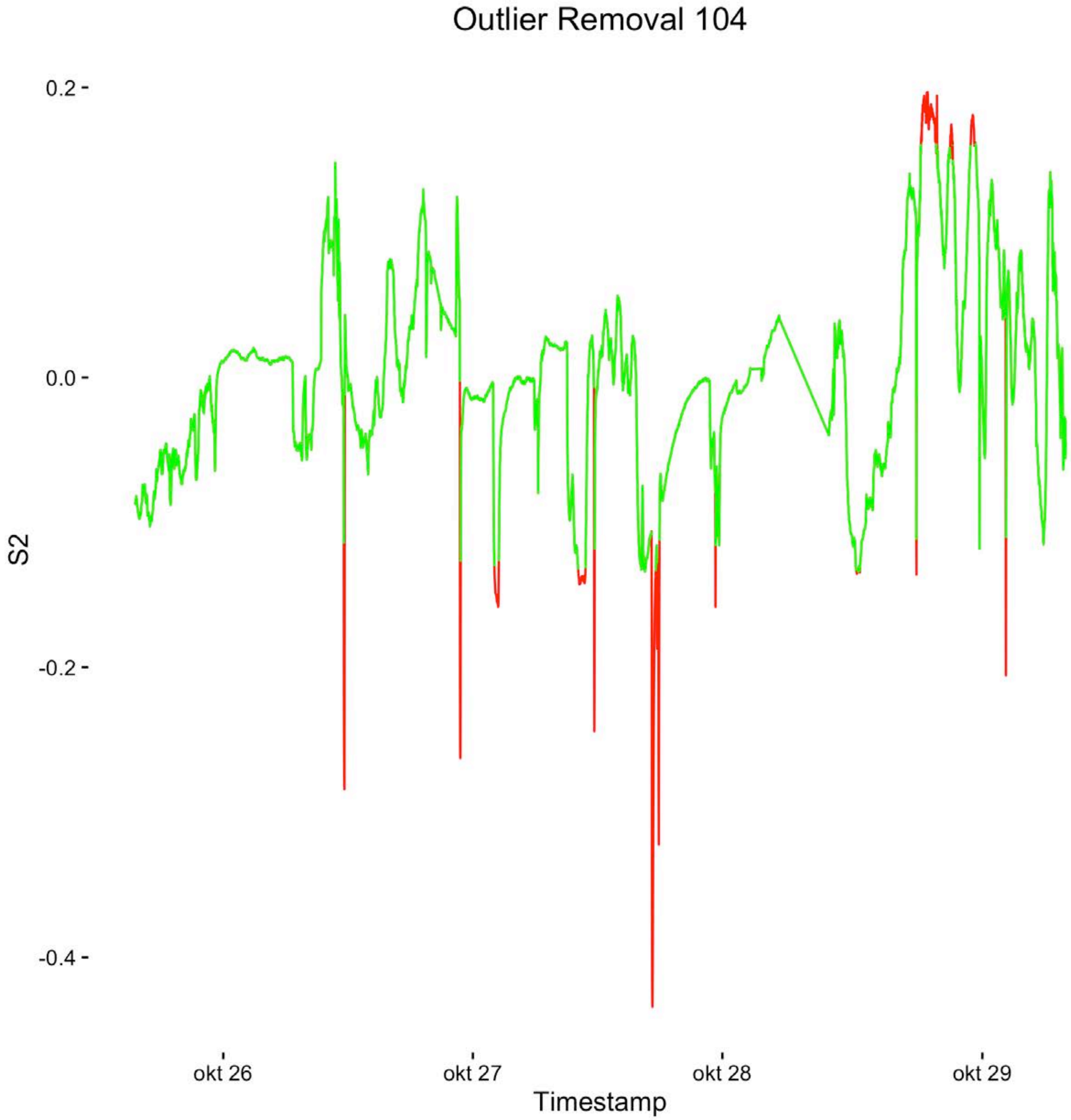
Outlier Removal 104
0.2
0.0
-0.2
-0.4
S2
okt 26
okt 27
okt 28
okt 29
Timestamp

Outlier Removal 106

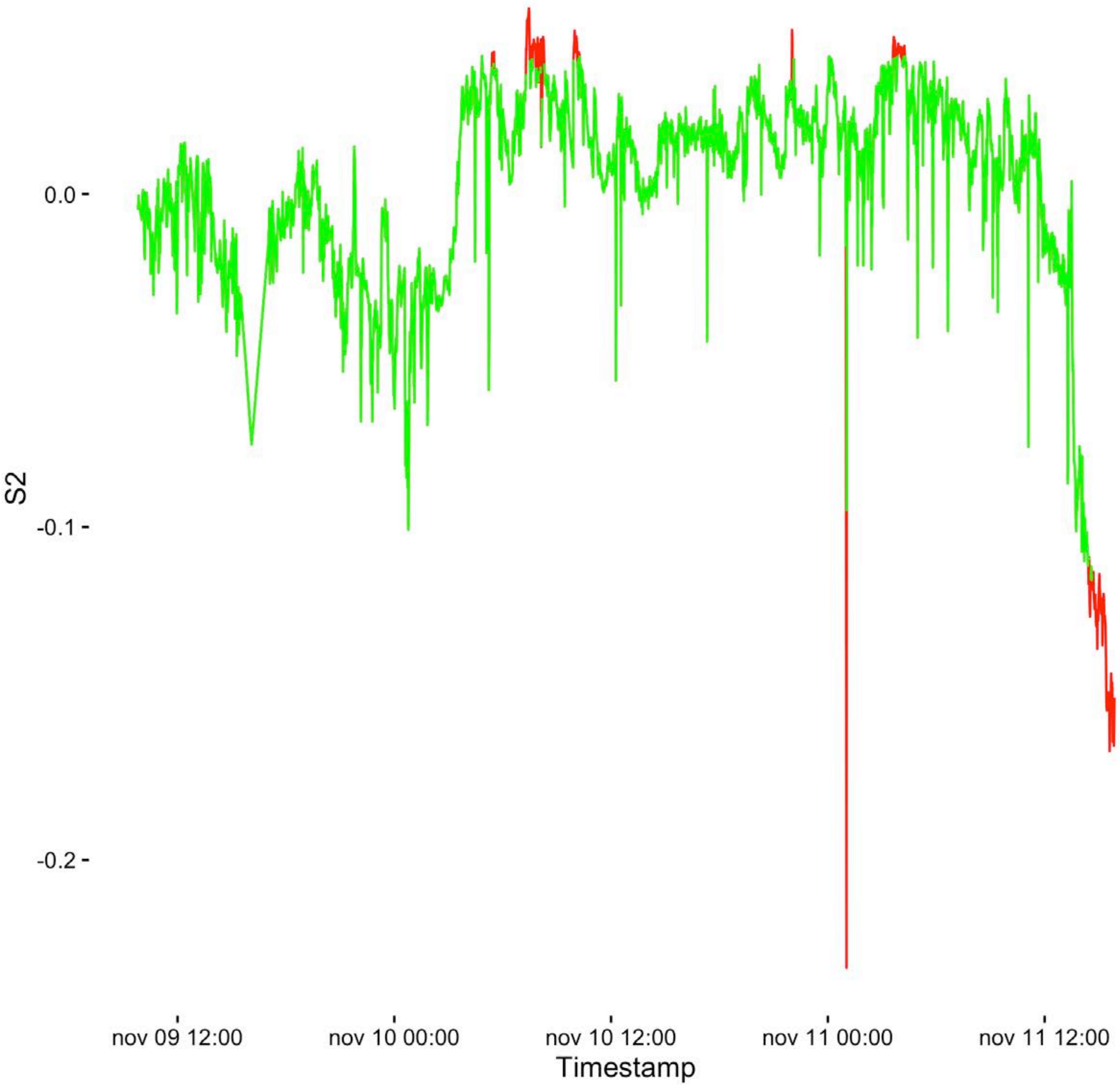

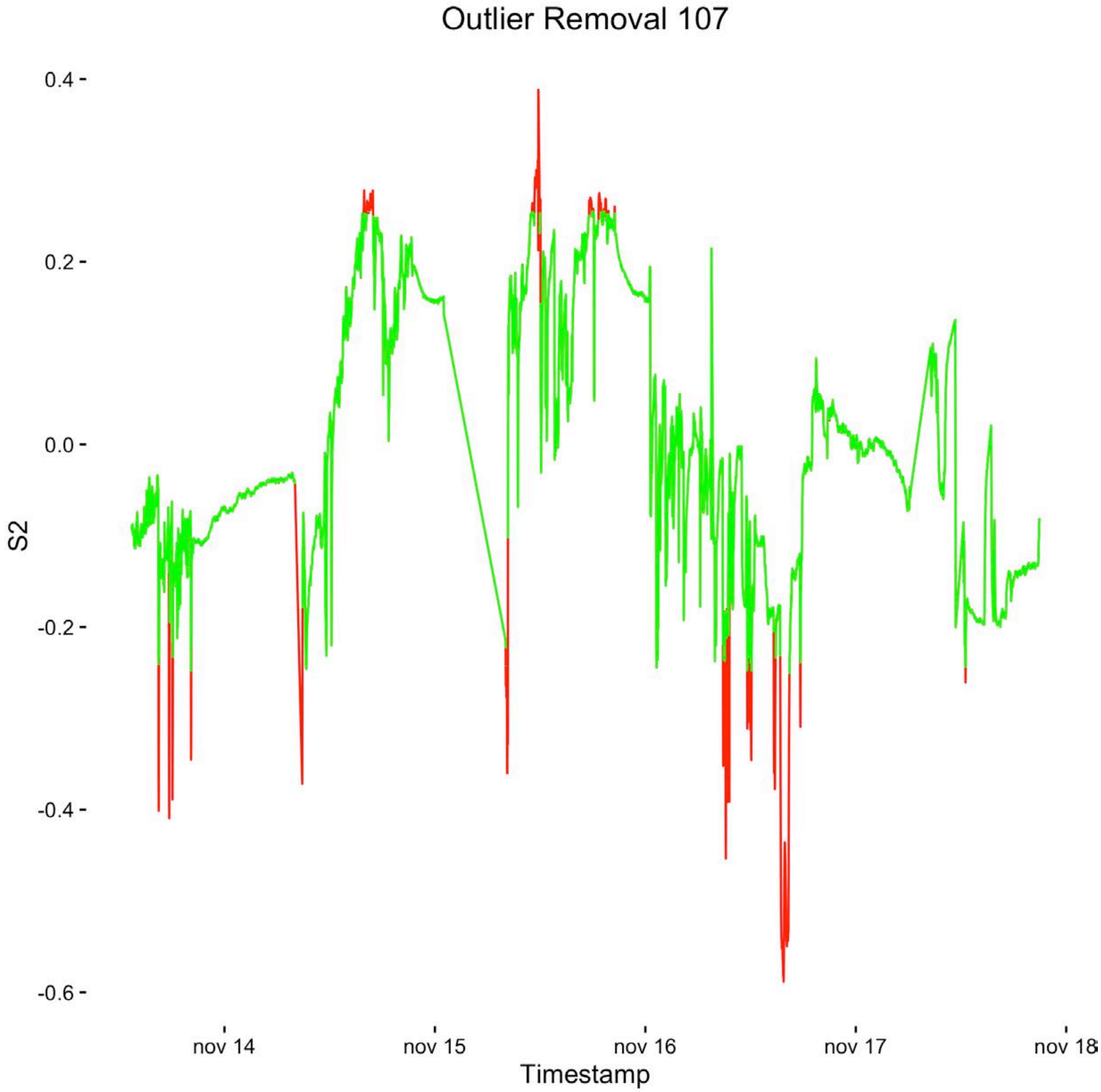

Outlier Removal 107
0.4
0.2
0.0
-0.2
-0.4
-0.6
S2
nov 14
nov 15
nov 16
nov 17
nov 18
Timestamp

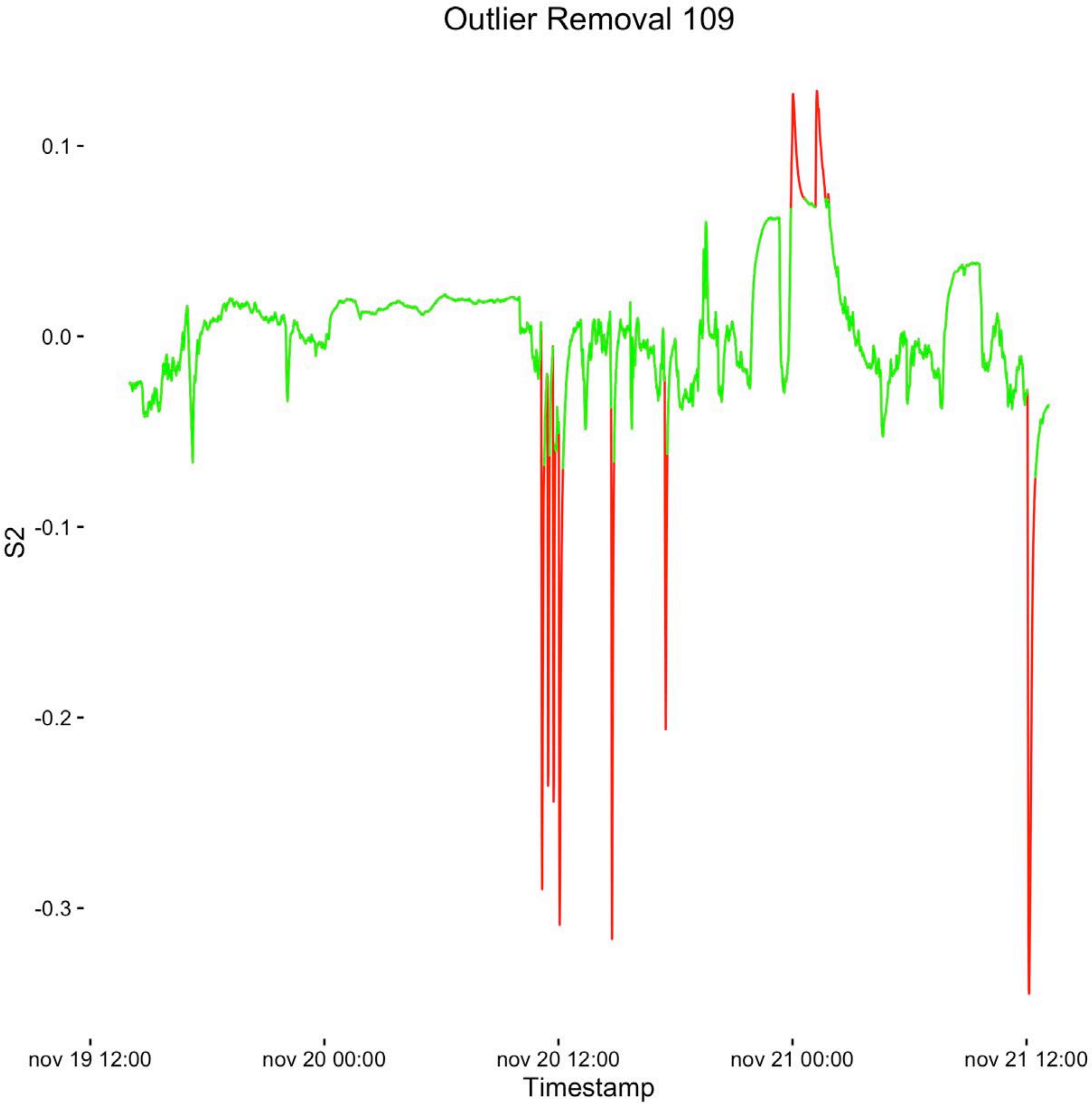

Outlier Removal 109
0.1
0.0
-0.1
-0.2
-0.3
S2
nov 19 12:00
nov 20 00:00
nov 20 12:00
nov 21 00:00
nov 21 12:00
Timestamp

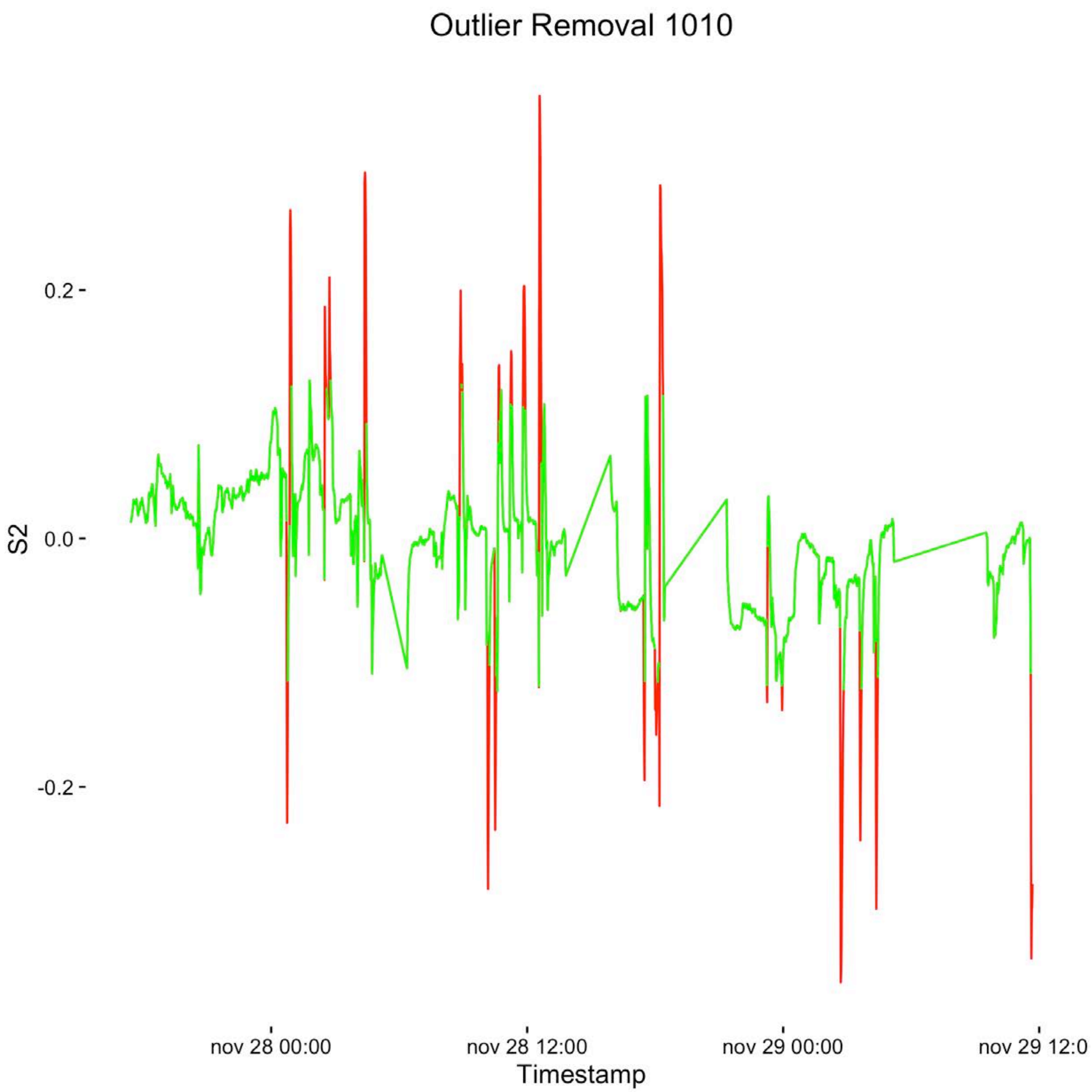
Outlier Removal 1010
S2
0.2
0.0
-0.2
nov 28 00:00
nov 28 12:00
nov 29 00:00
nov 29 12:0
Timestamp

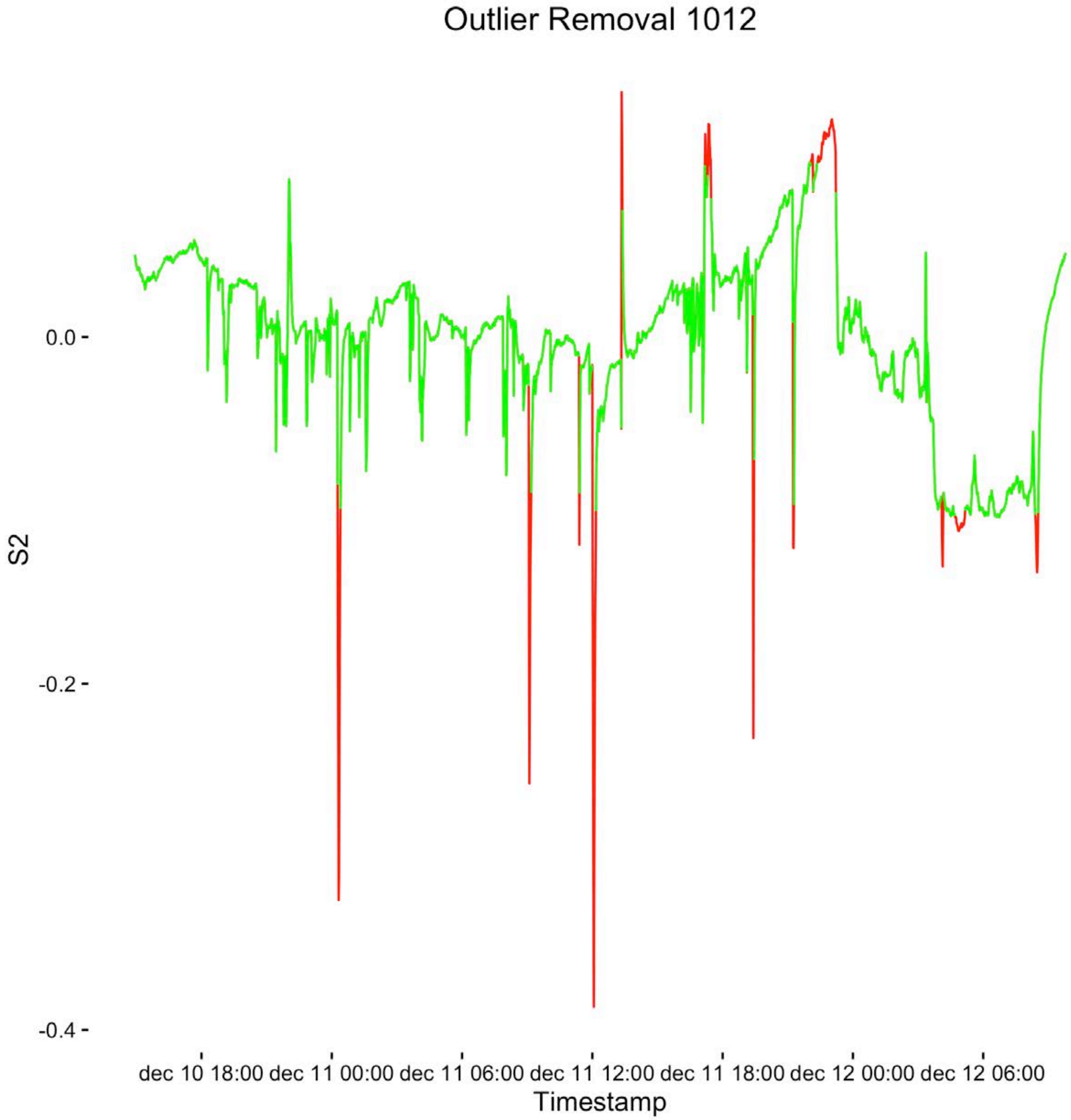
Outlier Removal 1012
S2
0.0
-0.2
-0.4
dec 10 18:00
dec 11 00:00
dec 11 06:00
dec 11 12:00
dec 11 18:00
dec 12 00:00
dec 12 06:00
Timestamp

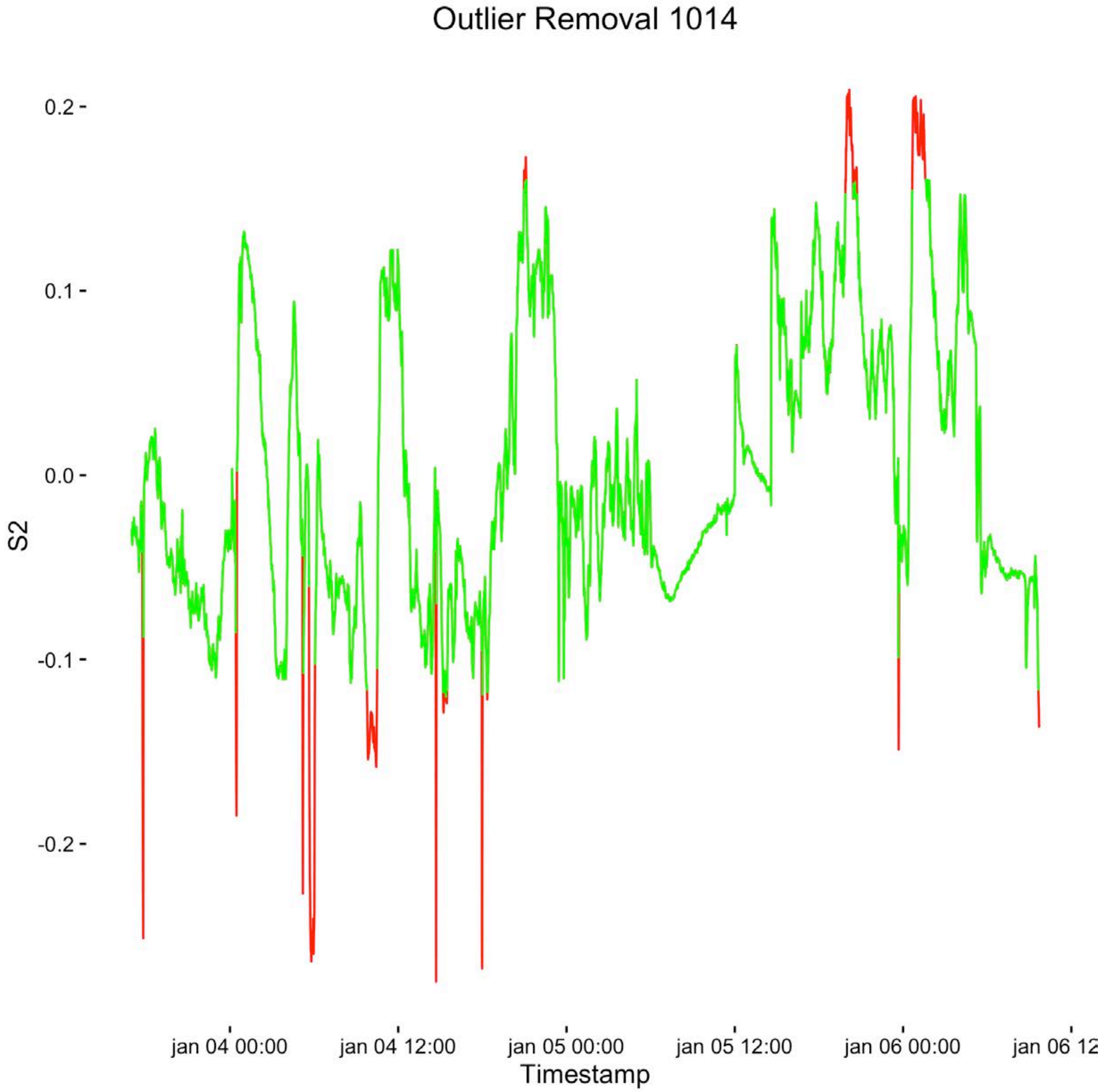

Outlier Removal 1014
S2
0.2
0.1
0.0
-0.1
-0.2
jan 04 00:00
jan 04 12:00
jan 05 00:00
jan 05 12:00
jan 06 00:00
jan 06 12
Timestamp

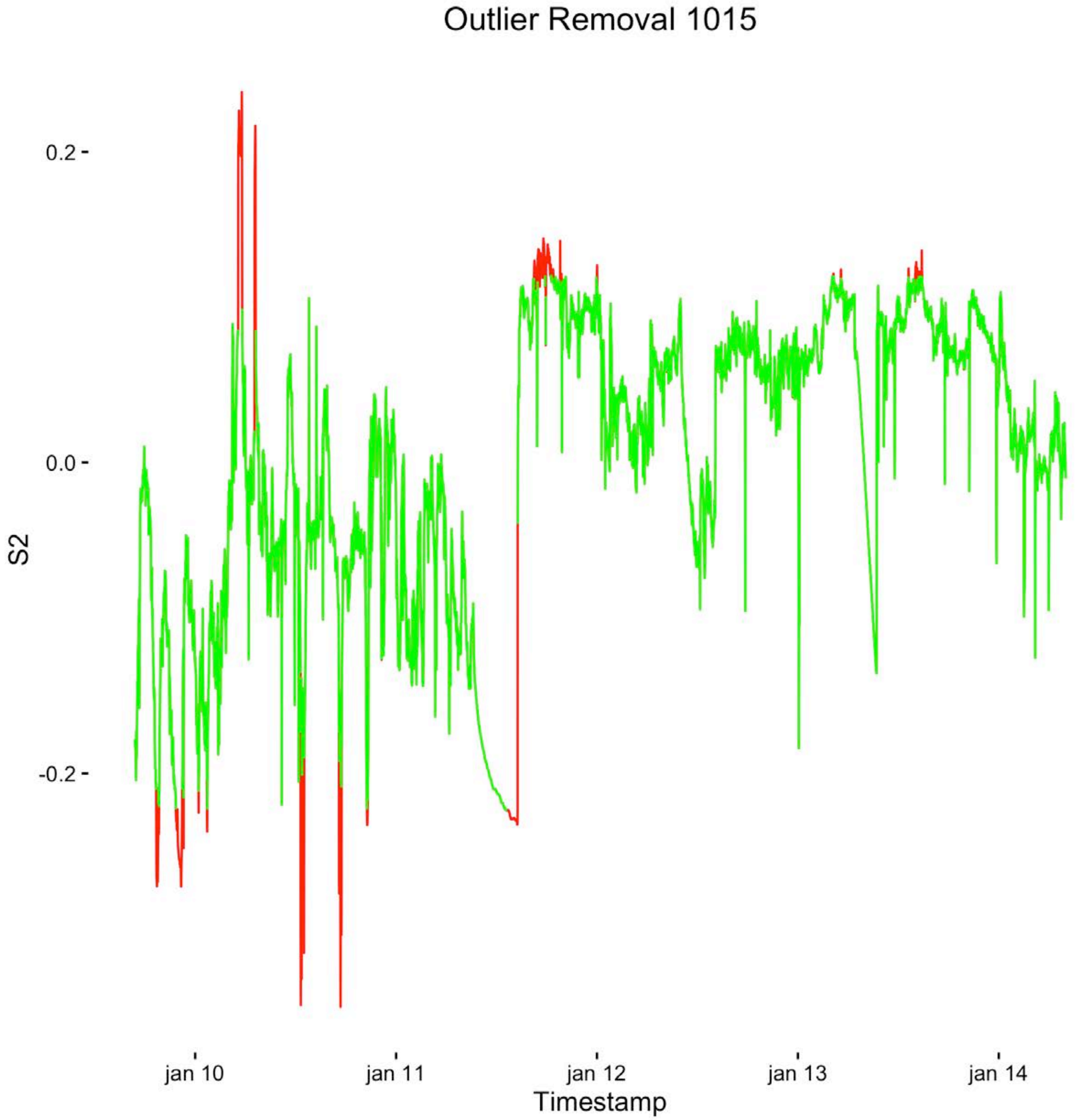

Outlier Removal 1015
S2
0.2
0.0
-0.2
jan 10
jan 11
jan 12
jan 13
jan 14
Timestamp

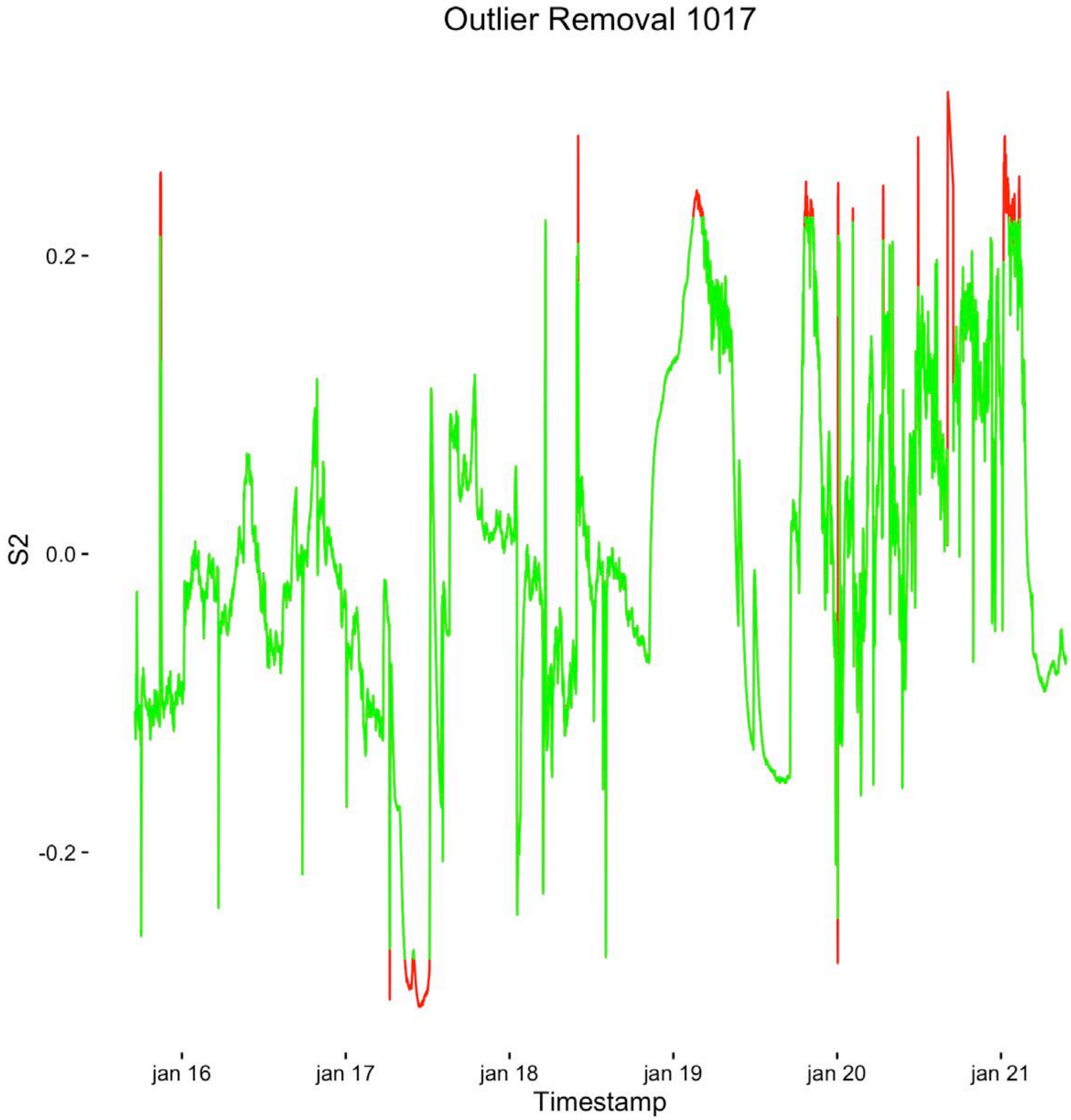
Outlier Removal 1017
S2
0.2
0.0
-0.2
jan 16
jan 17
jan 18
jan 19
jan 20
jan 21
Timestamp

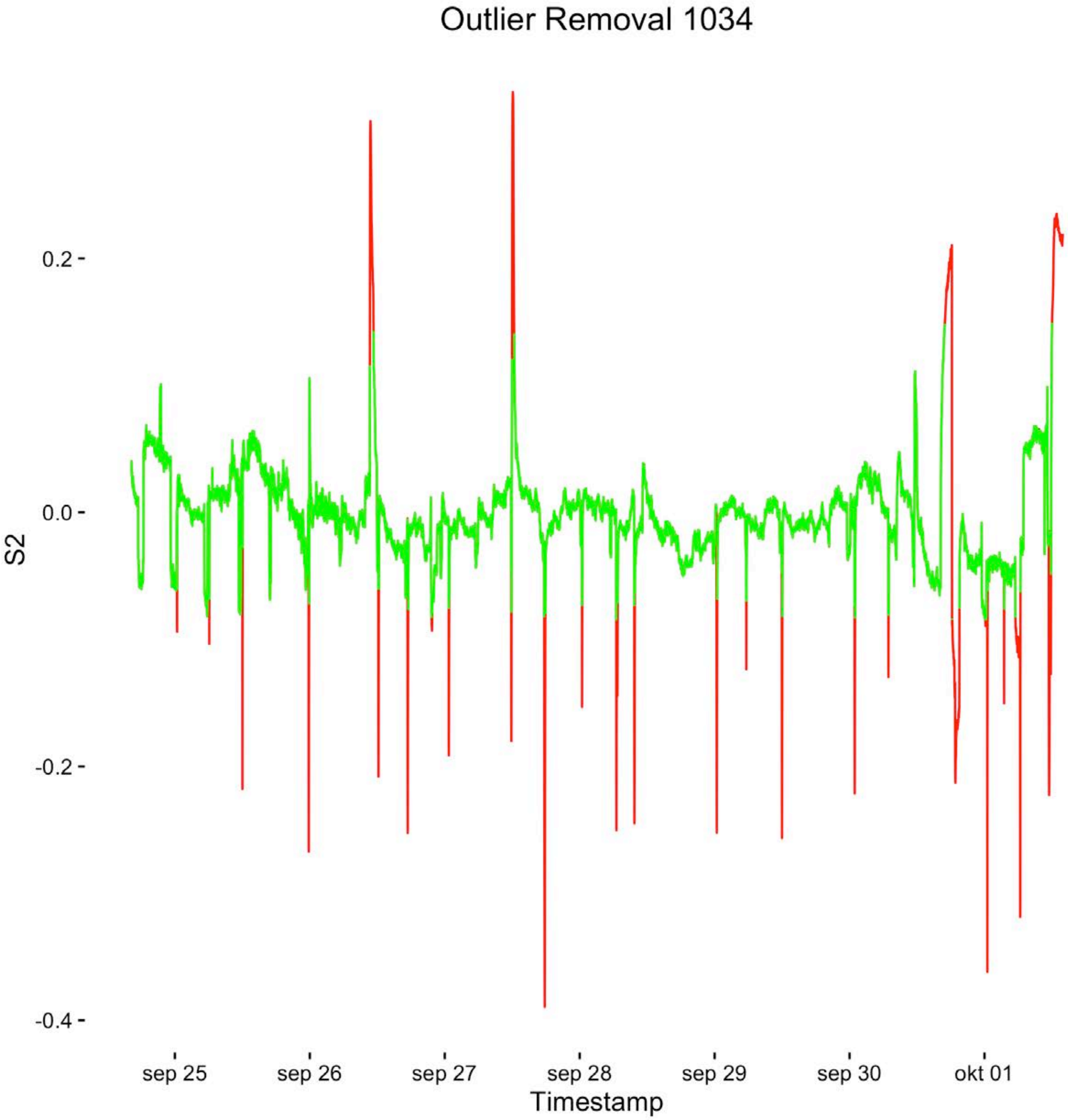

Outlier Removal 1034
S2
0.2
0.0
-0.2
-0.4
sep 25
sep 26
sep 27
sep 28
sep 29
sep 30
okt 01
Timestamp

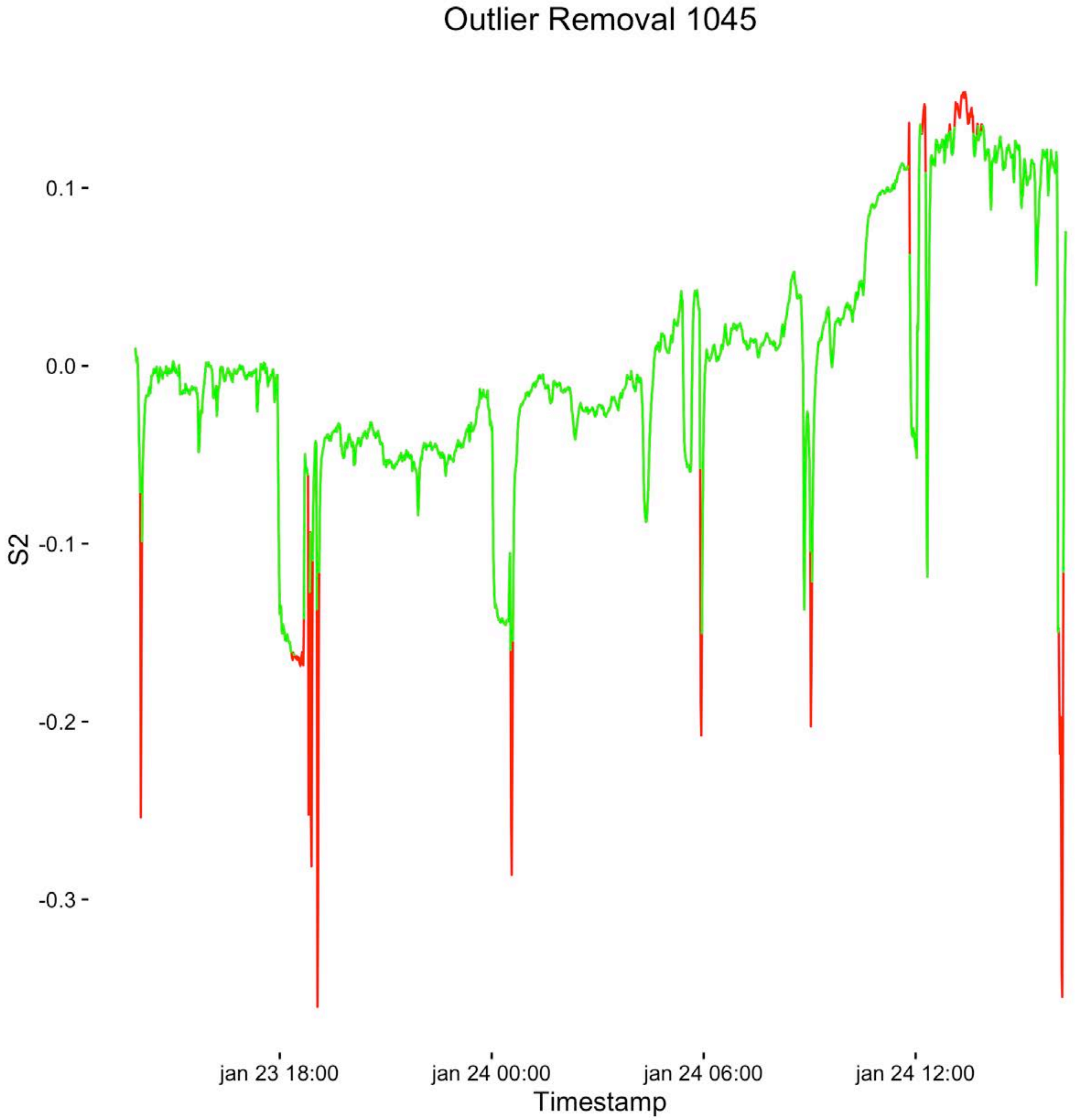

Outlier Removal 1045
S2
0.1
0.0
-0.1
-0.2
-0.3
jan 23 18:00
jan 24 00:00
jan 24 06:00
jan 24 12:00
Timestamp

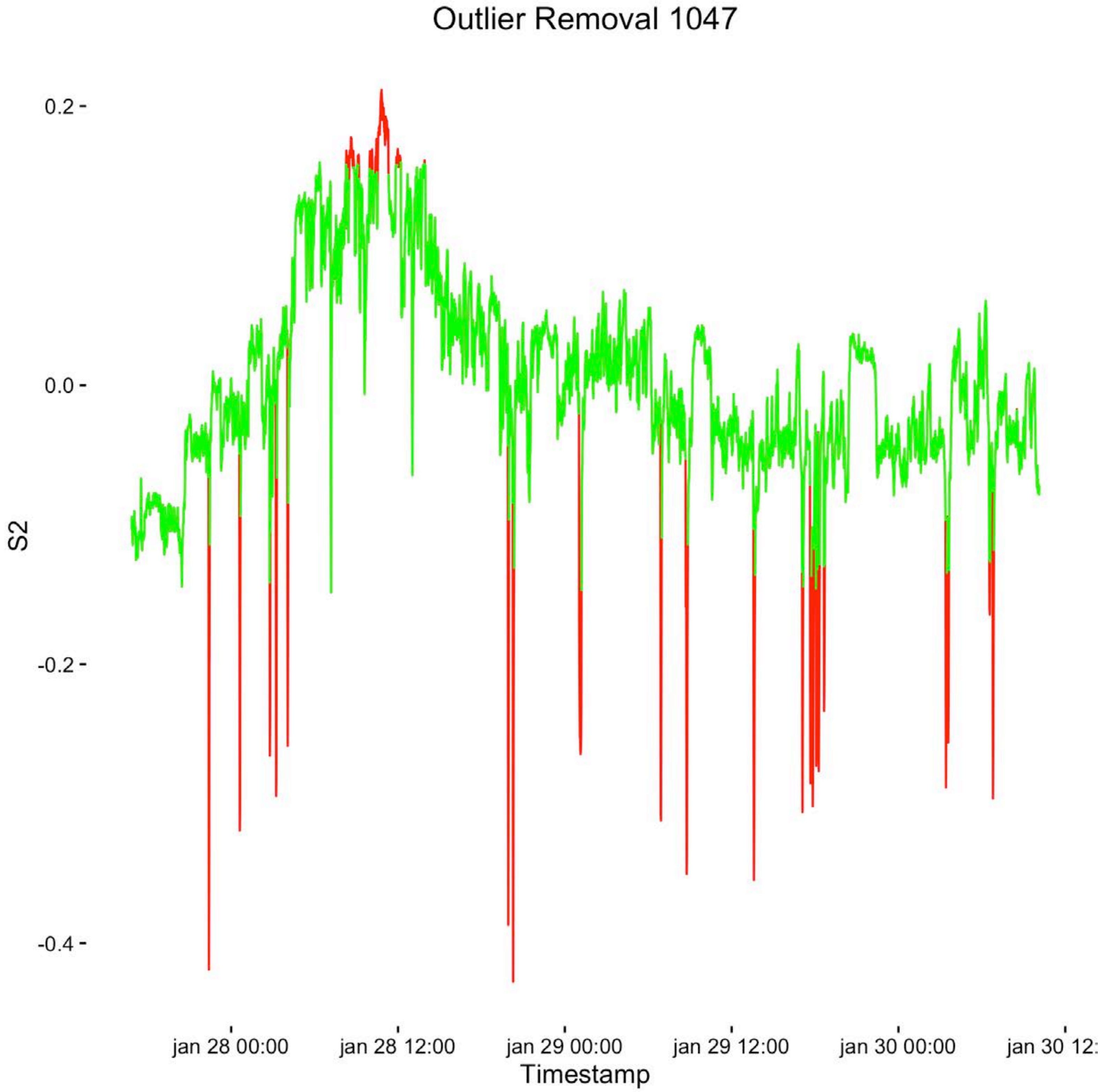
Outlier Removal 1047
0.2
0.0
-0.2
-0.4
S2
jan 28 00:00
jan 28 12:00
jan 29 00:00
jan 29 12:00
jan 30 00:00
Timestamp

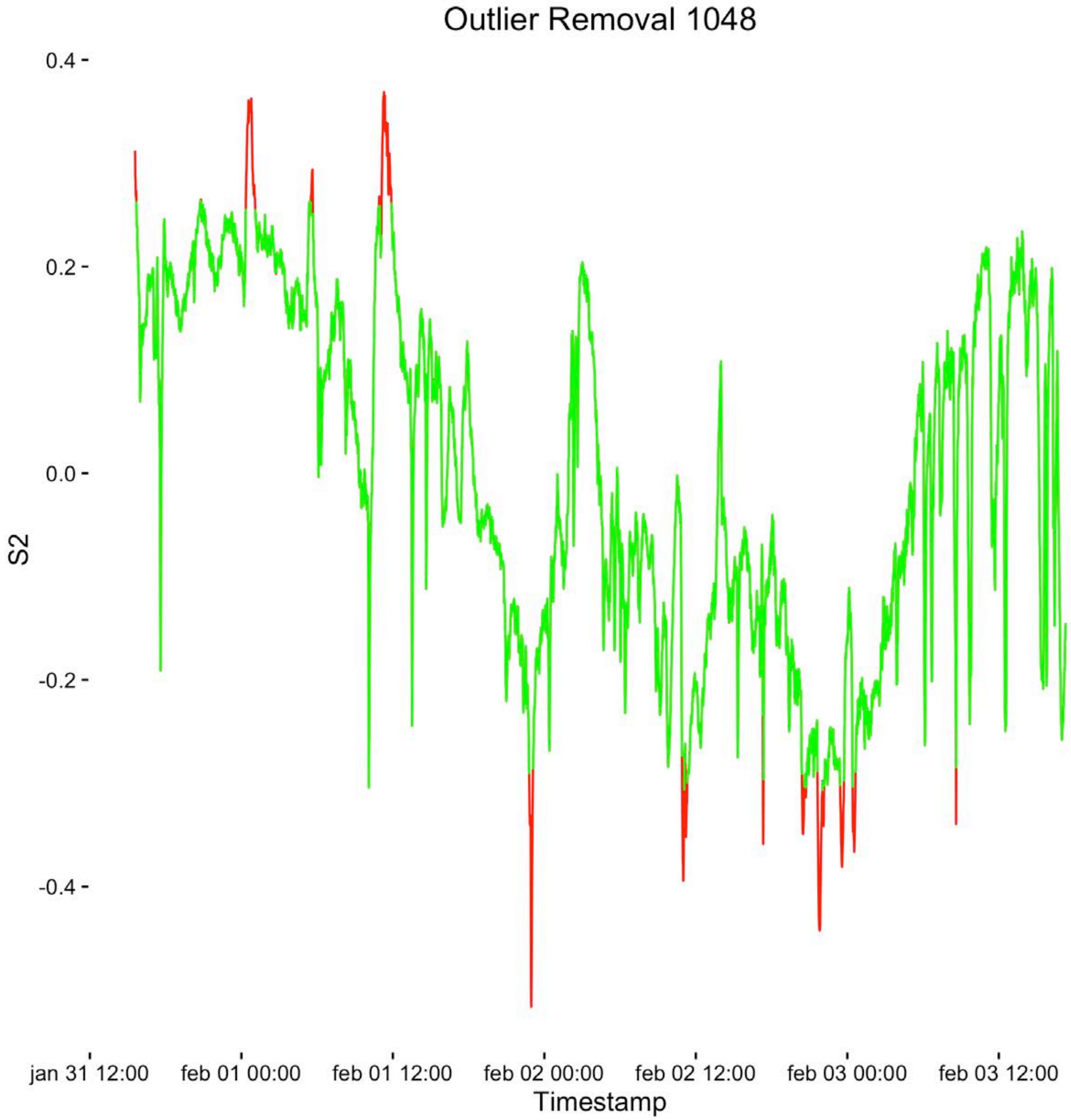

Outlier Removal 1048
0.4
0.2
0.0
-0.2
-0.4
S2
jan 31 12:00
feb 01 00:00
feb 01 12:00
feb 02 00:00
feb 02 12:00
feb 03 00:00
feb 03 12:00
Timestamp

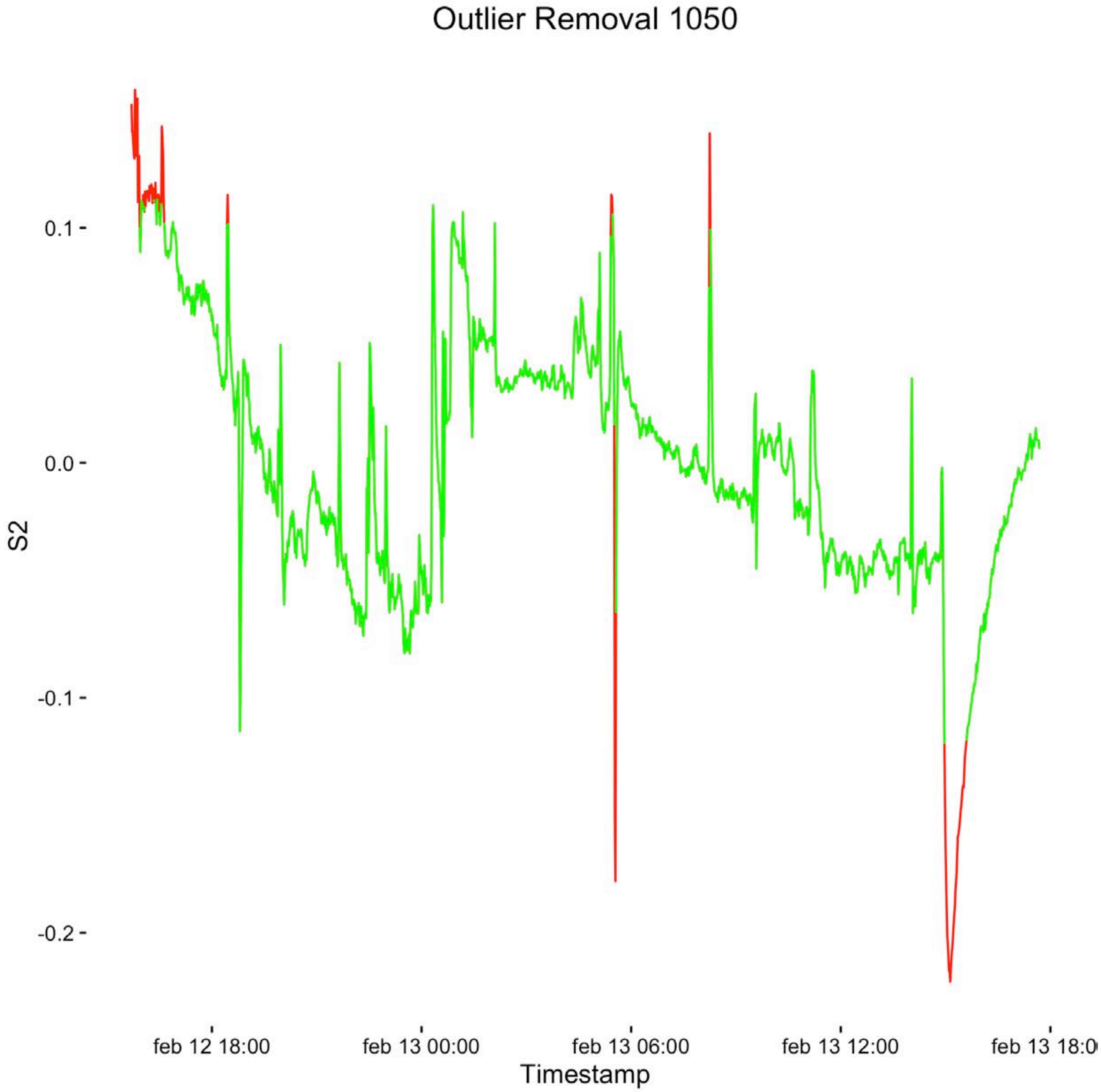
Outlier Removal 1050
S2
0.1
0.0
-0.1
-0.2
feb 12 18:00
feb 13 00:00
feb 13 06:00
feb 13 12:00
feb 13 18:0
Timestamp

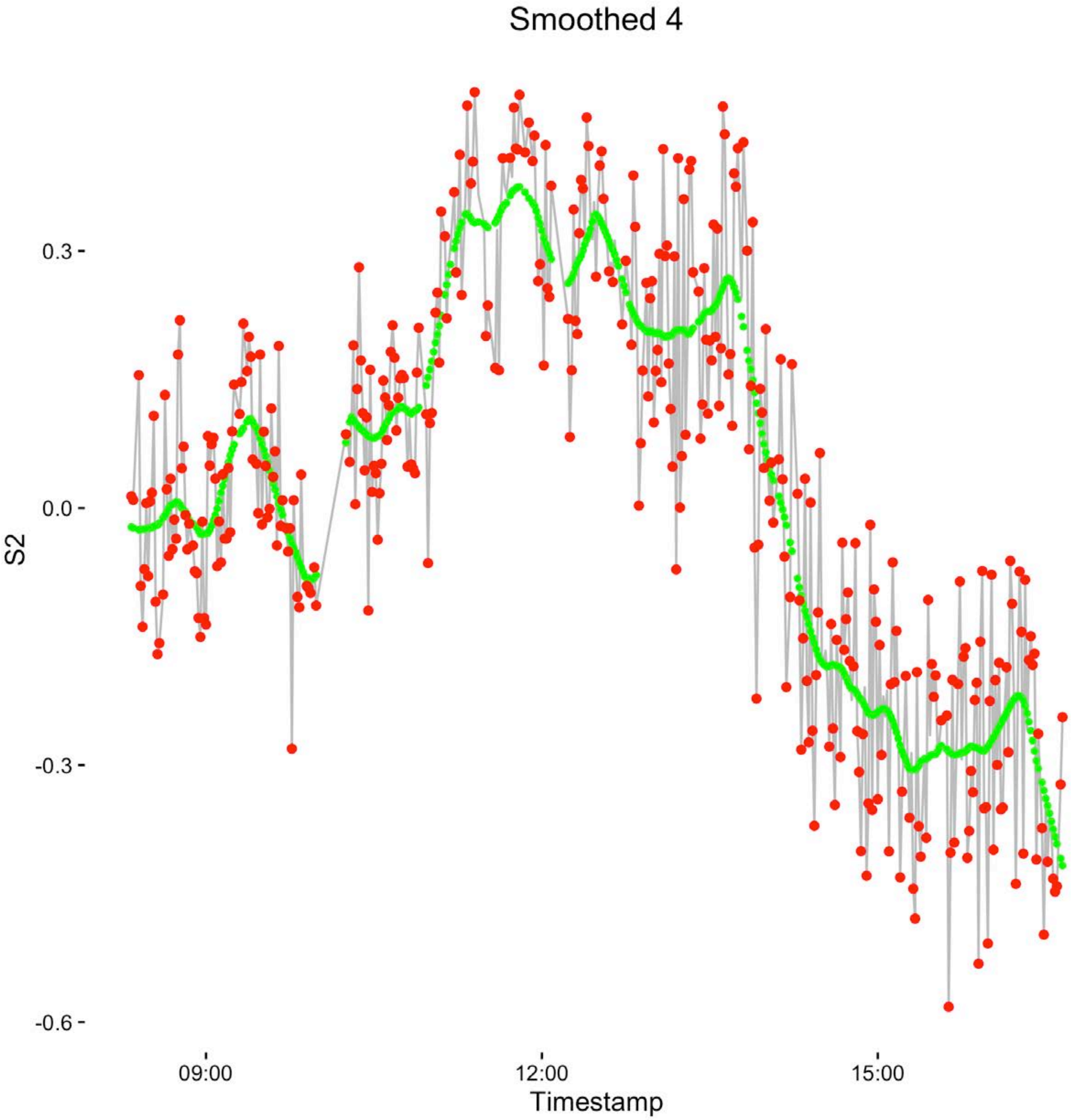
Smoothed 4
S2
0.3
0.0
-0.3
-0.6
09:00
12:00
15:00
Timestamp

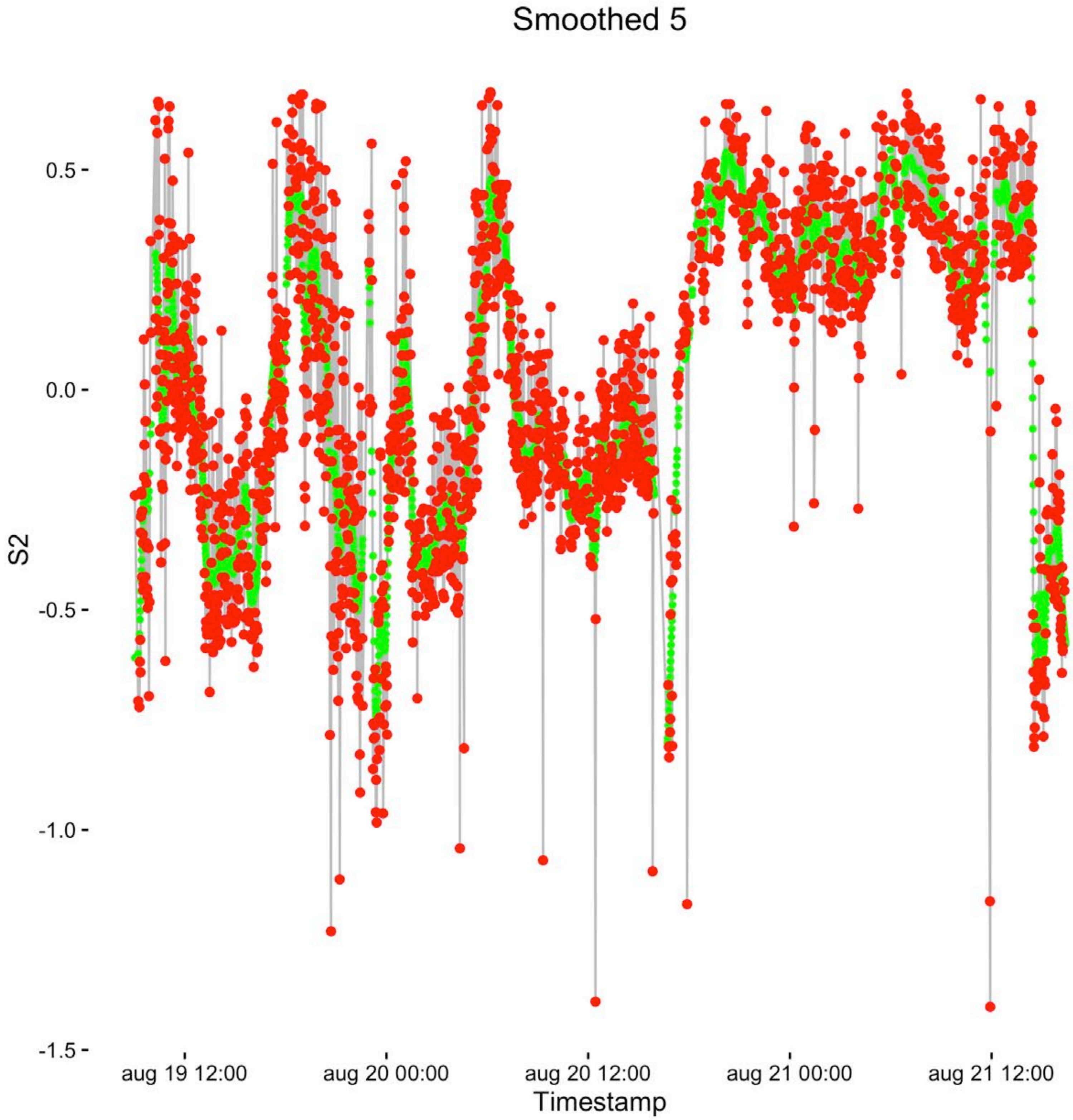

Smoothed 5
S2
0.5
0.0
-0.5
-1.0
-1.5
aug 19 12:00
aug 20 00:00
aug 20 12:00
aug 21 00:00
aug 21 12:00
Timestamp

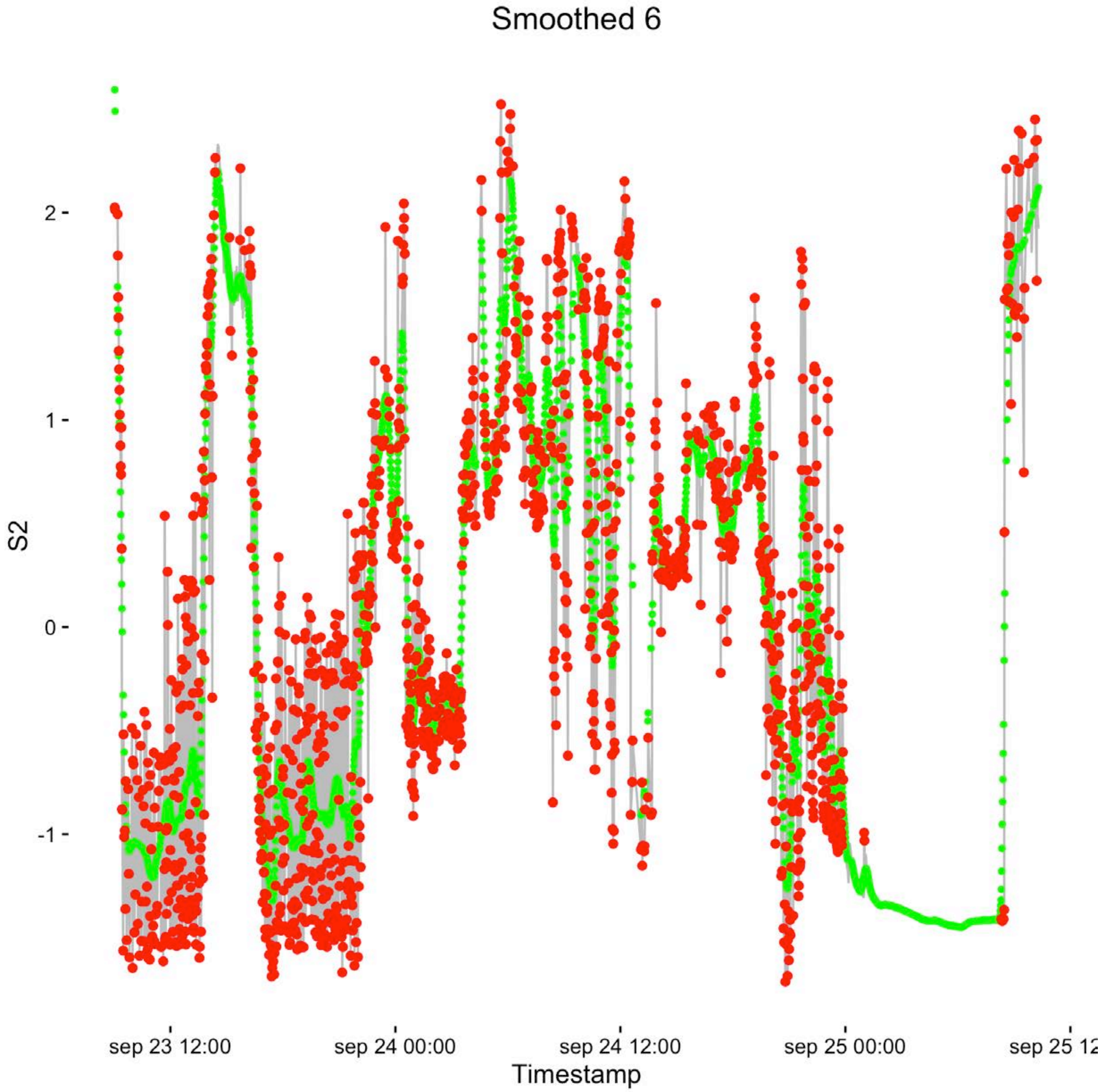

Smoothed 6
S2
2
1
0
-1
sep 23 12:00
sep 24 00:00
sep 24 12:00
sep 25 00:00
sep 25 12
Timestamp

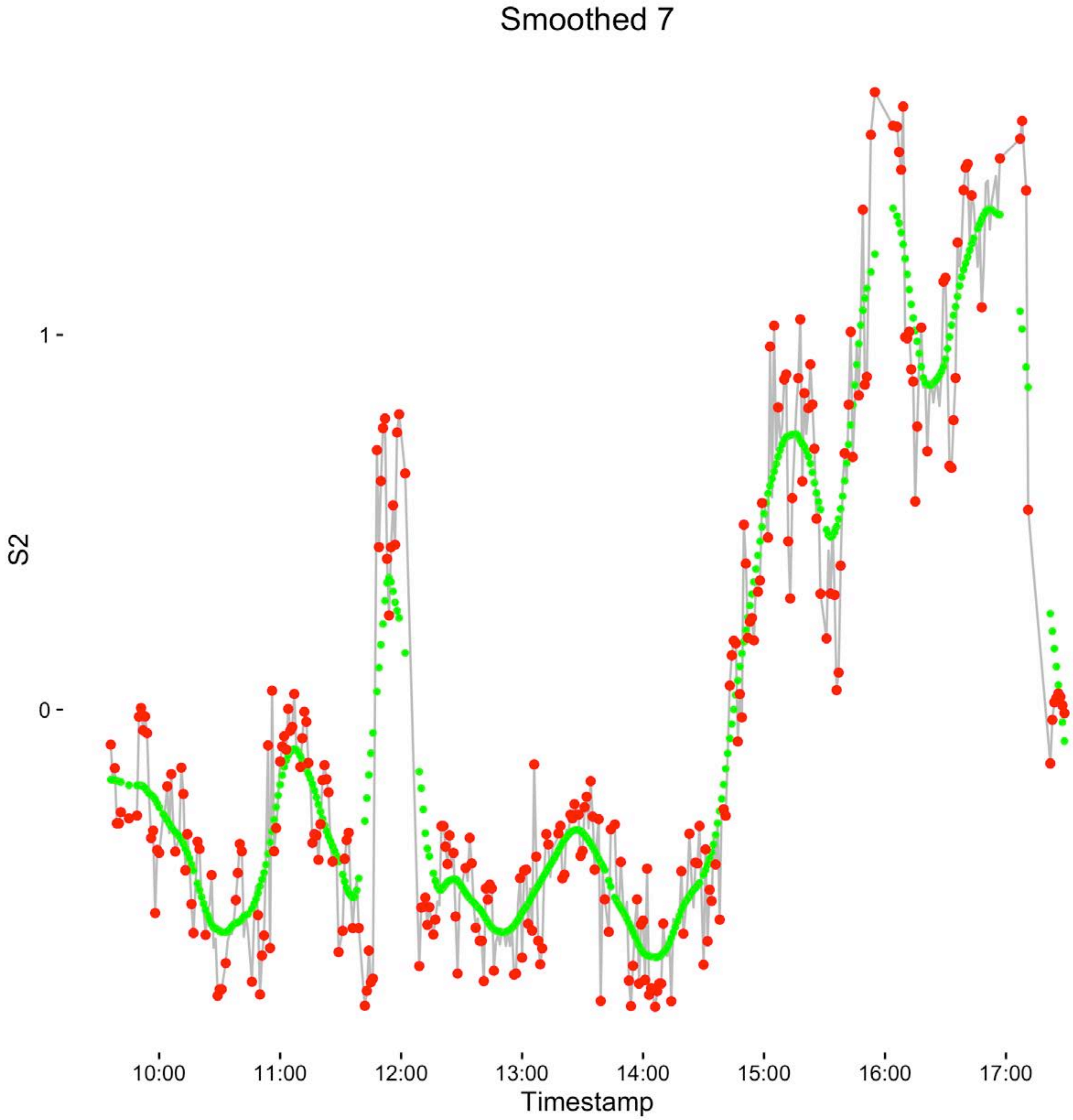

Smoothed 7
S2
1
0
10:00
11:00
12:00
13:00
14:00
15:00
16:00
17:00
Timestamp

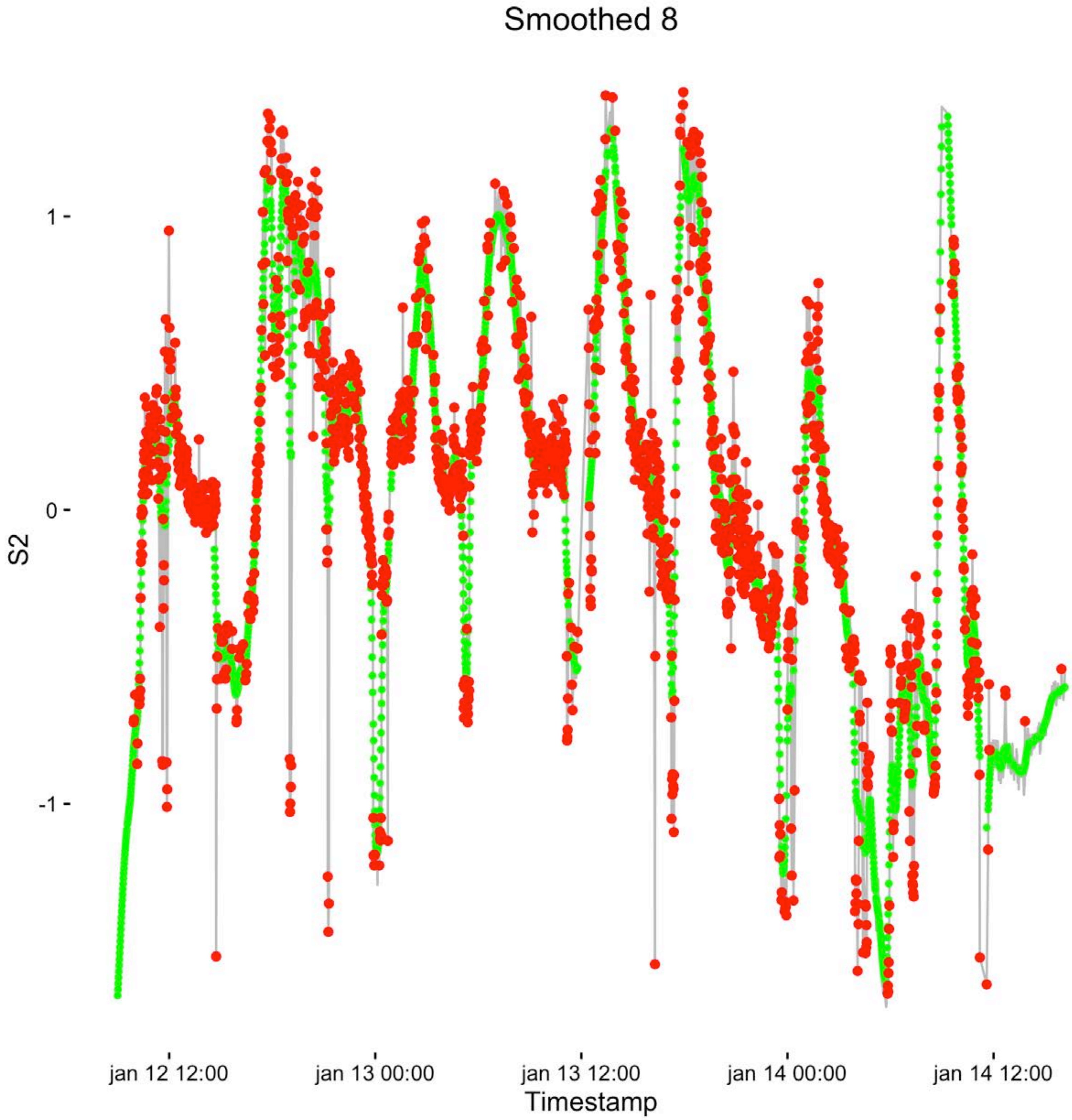

Smoothed 8
S2
1
0
-1
jan 12 12:00
jan 13 00:00
jan 13 12:00
jan 14 00:00
jan 14 12:00
Timestamp

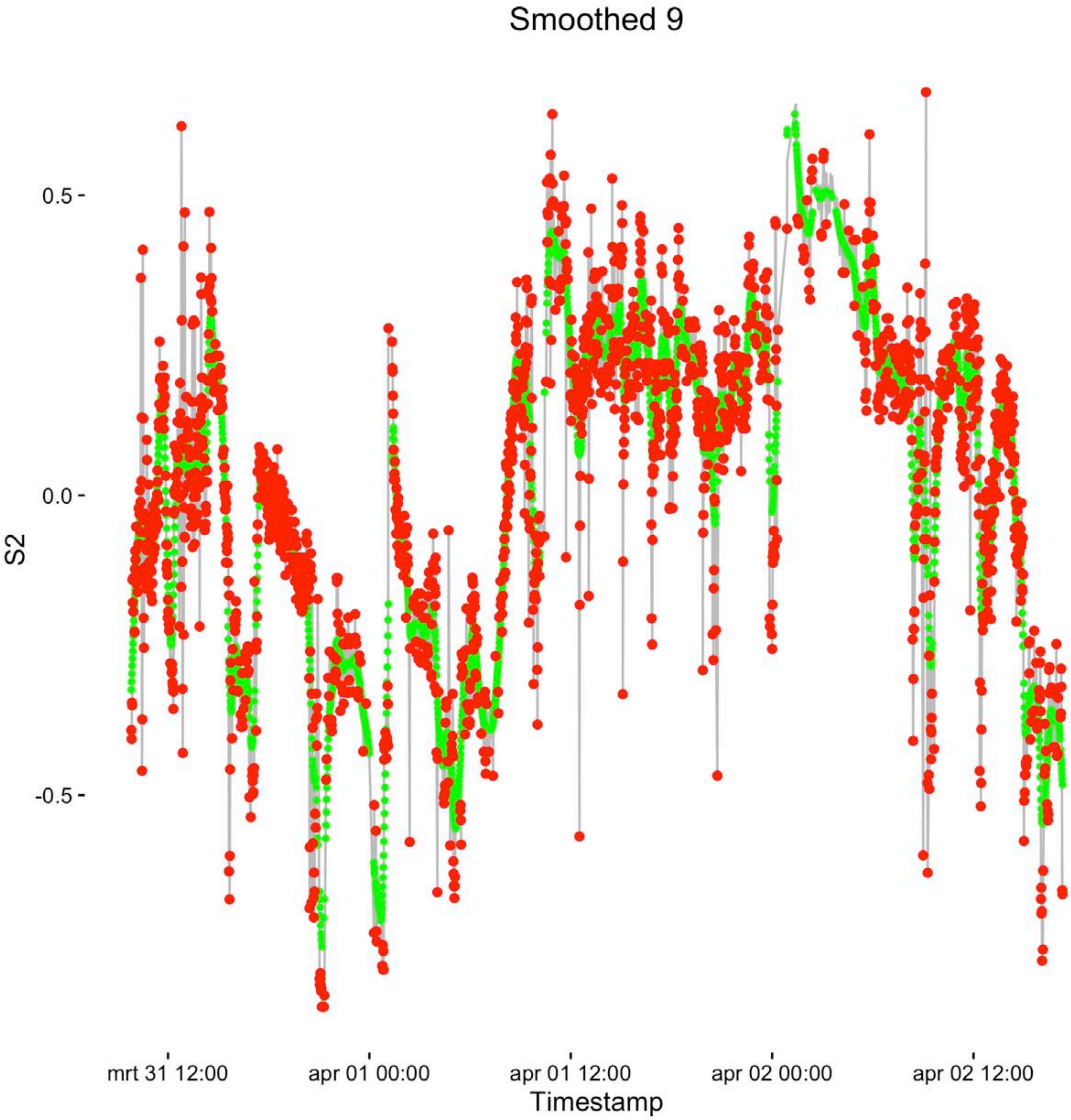
Smoothed 9
0.5
0.0
-0.5
S2
mrt 31 12:00
apr 01 00:00
apr 01 12:00
apr 02 00:00
apr 02 12:00
Timestamp

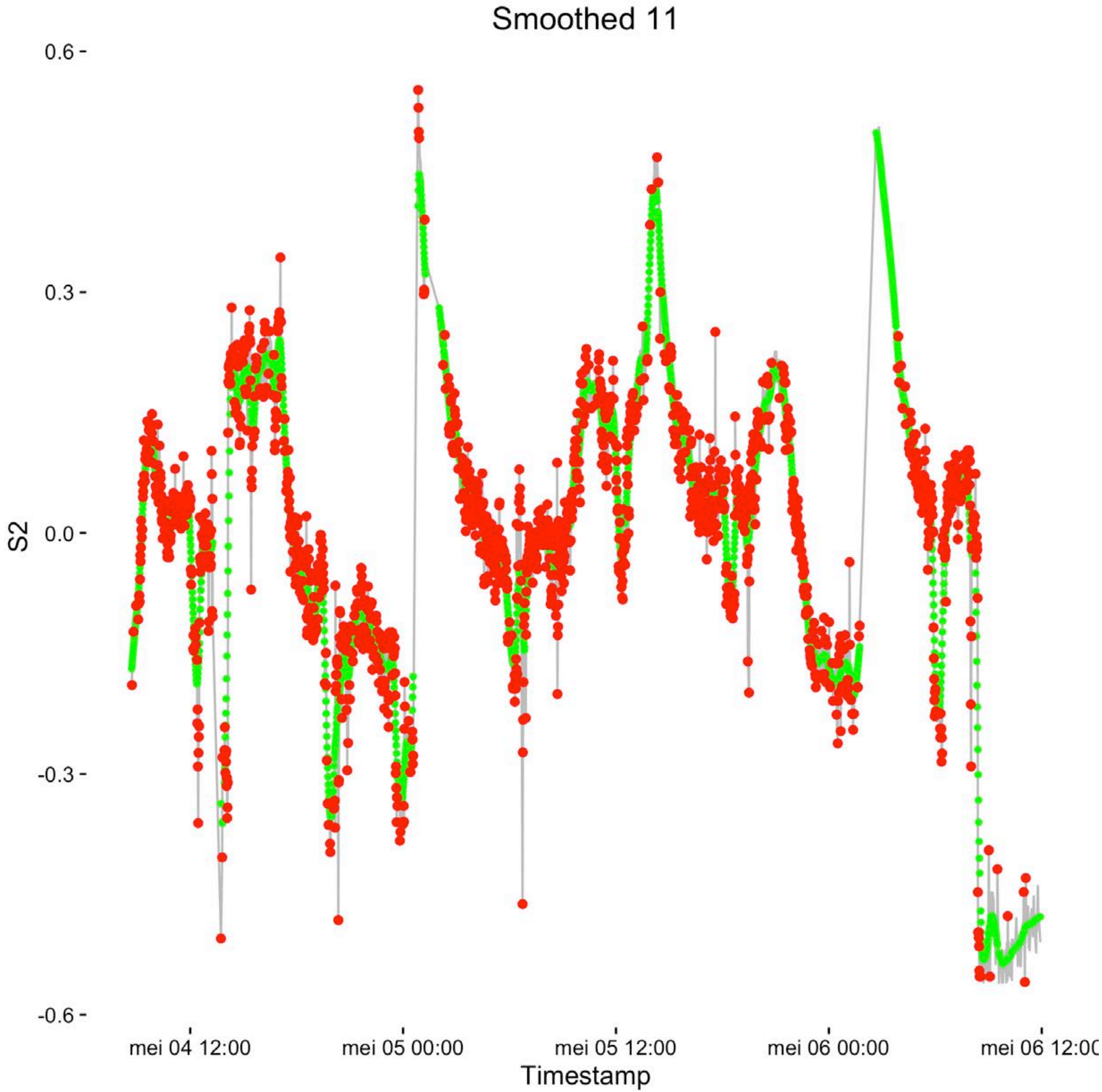

Smoothed 11
0.6
0.3
0.0
-0.3
-0.6
S2
mei 04 12:00
mei 05 00:00
mei 05 12:00
mei 06 00:00
mei 06 12:00
Timestamp

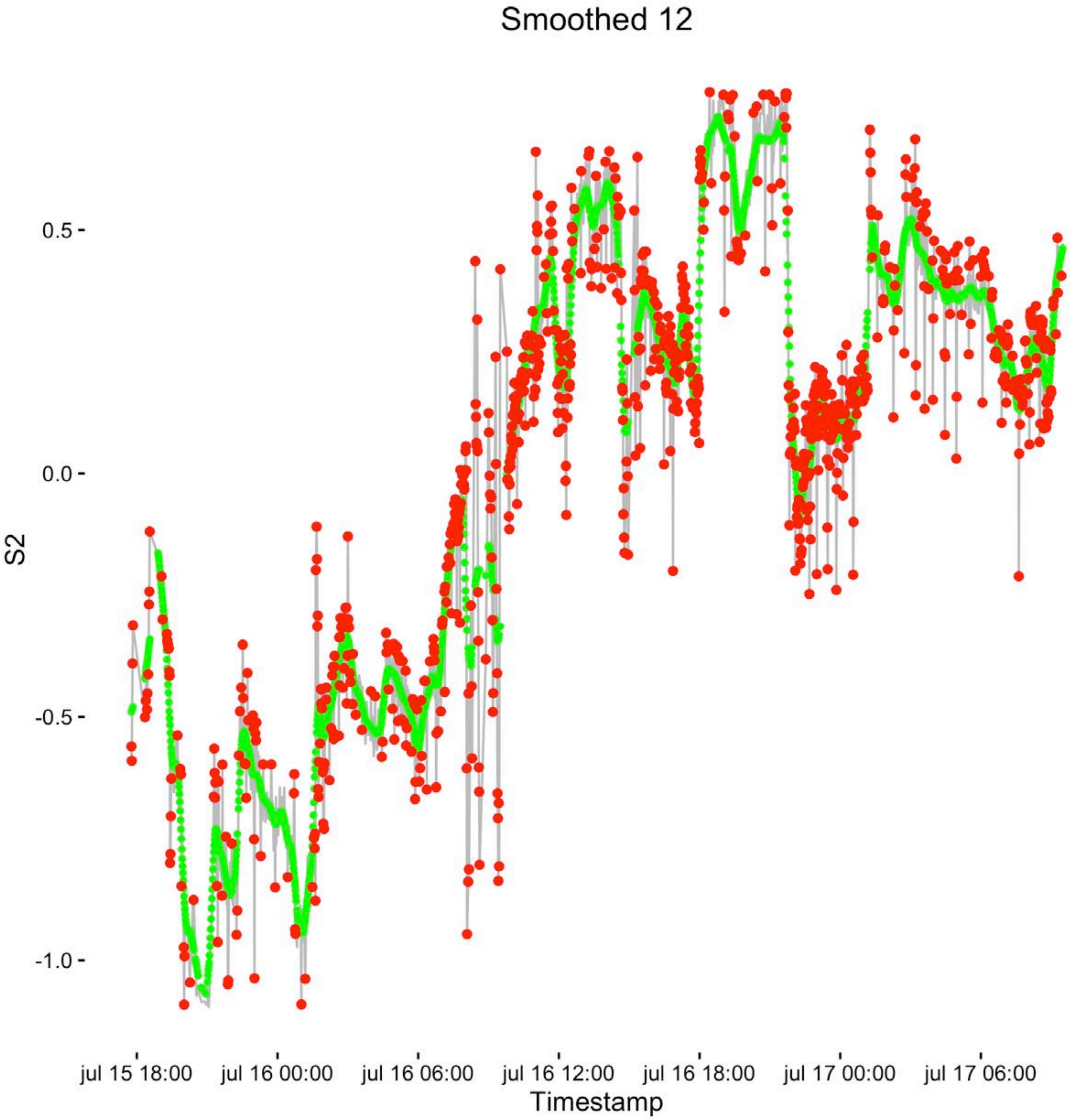
Smoothed 12
S2
0.5
0.0
-0.5
-1.0
jul 15 18:00
jul 16 00:00
jul 16 06:00
jul 16 12:00
jul 16 18:00
jul 17 00:00
jul 17 06:00
Timestamp

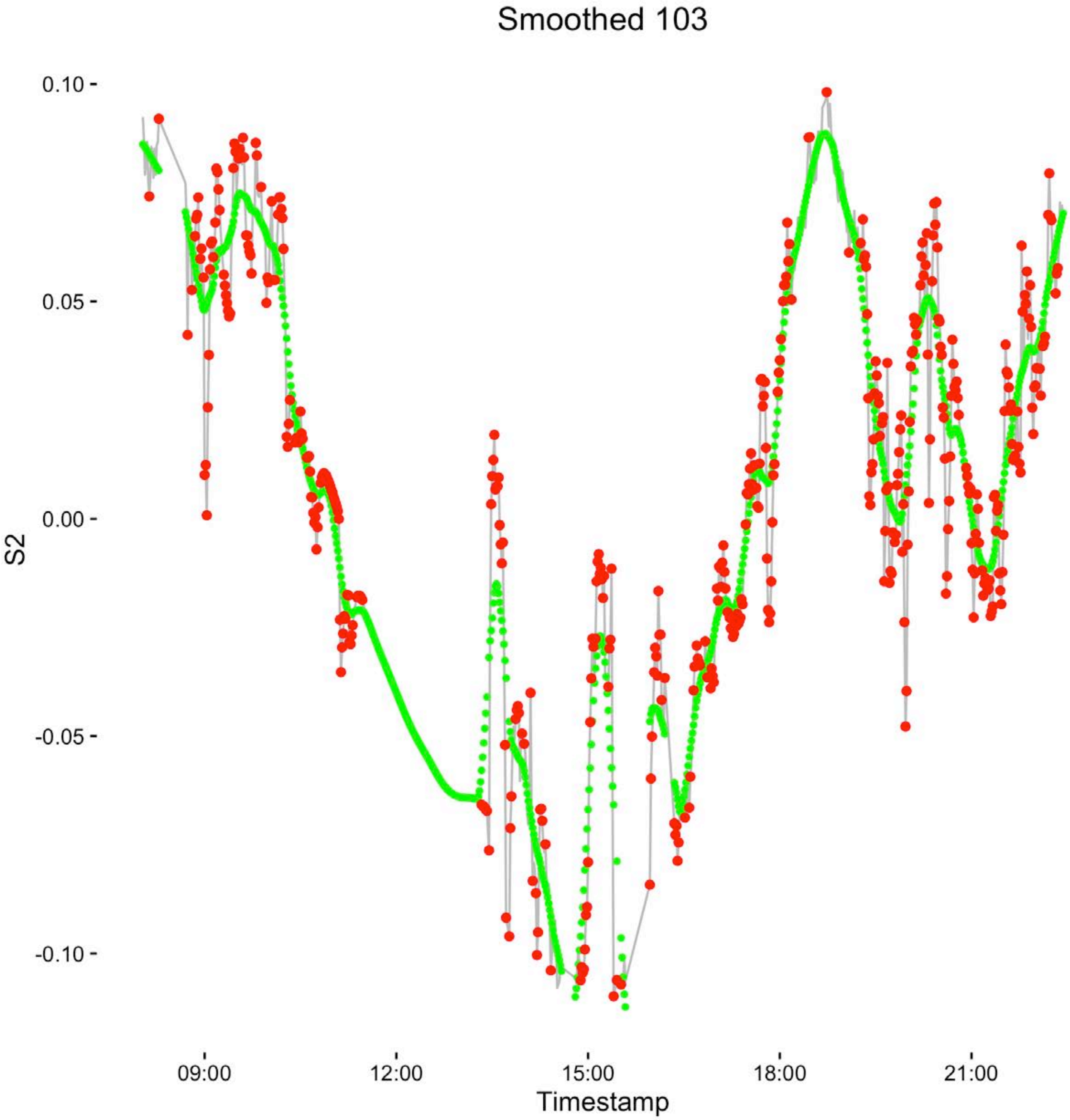
Smoothed 103
0.10
0.05
0.00
-0.05
-0.10
S2
09:00
12:00
15:00
18:00
21:00
Timestamp

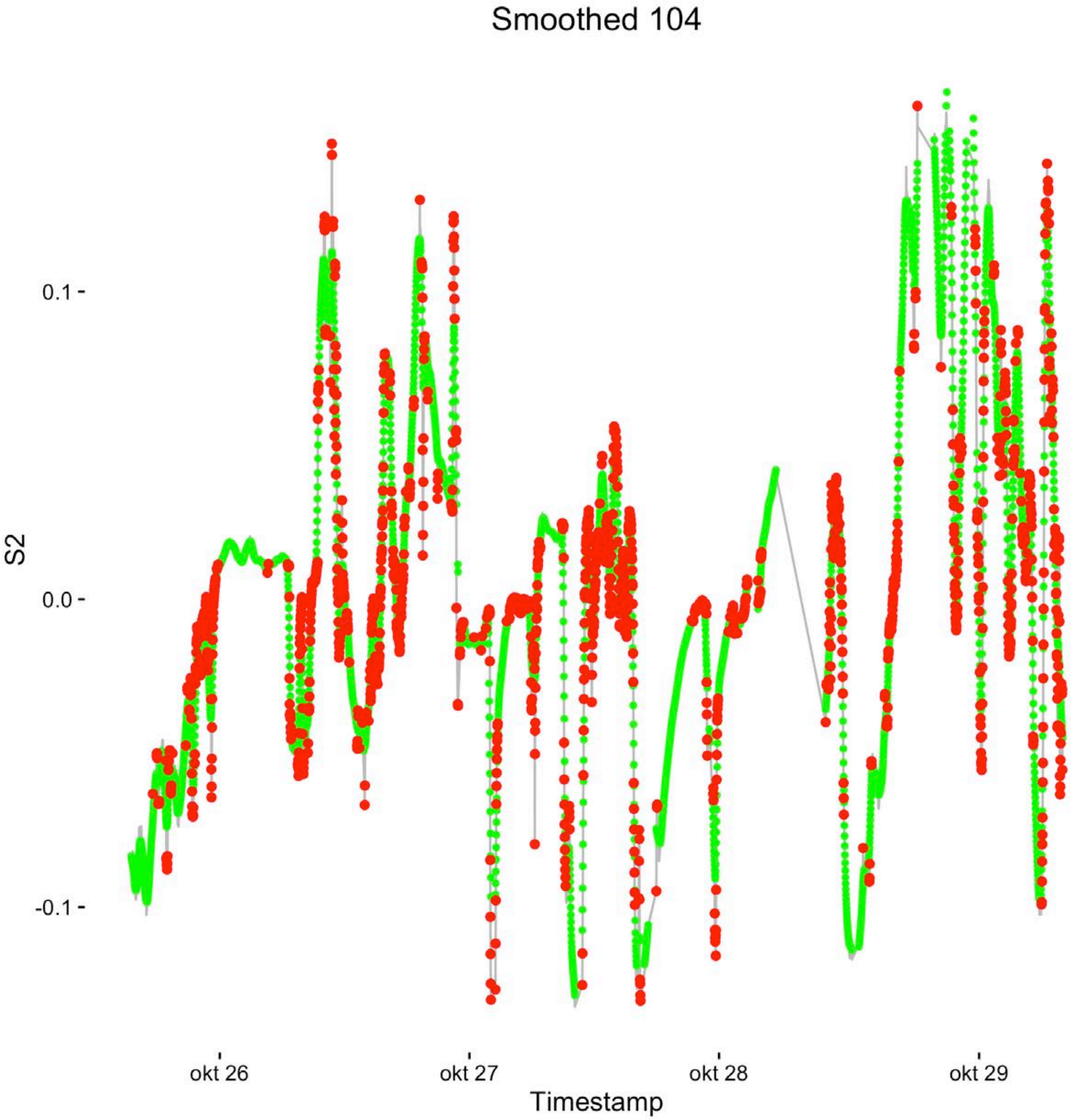
Smoothed 104
S2
0.1
0.0
-0.1
okt 26
okt 27
okt 28
okt 29
Timestamp

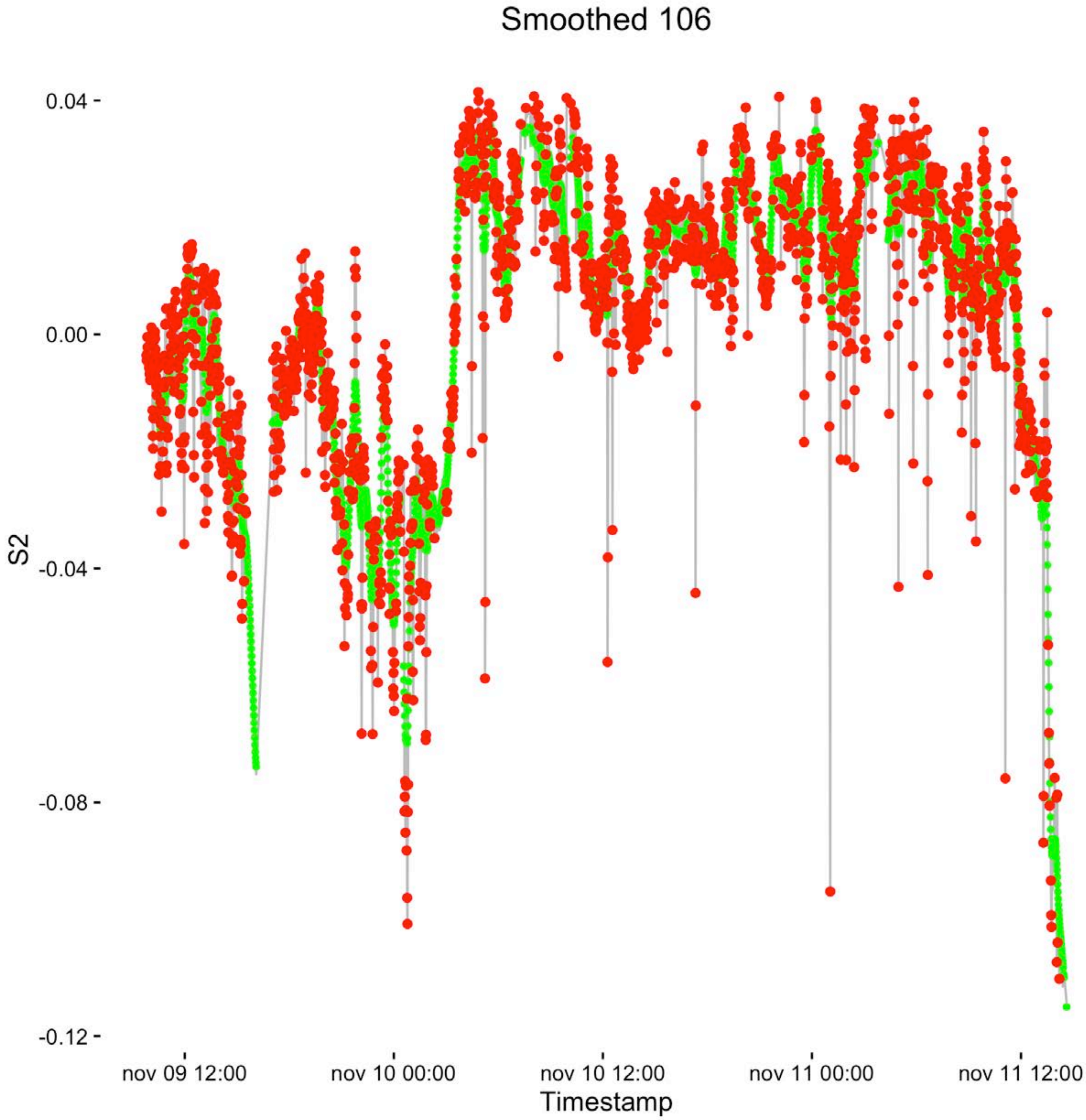
Smoothed 106
0.04
0.00
-0.04
-0.08
-0.12
S2
nov 09 12:00
nov 10 00:00
nov 10 12:00
nov 11 00:00
nov 11 12:00
Timestamp

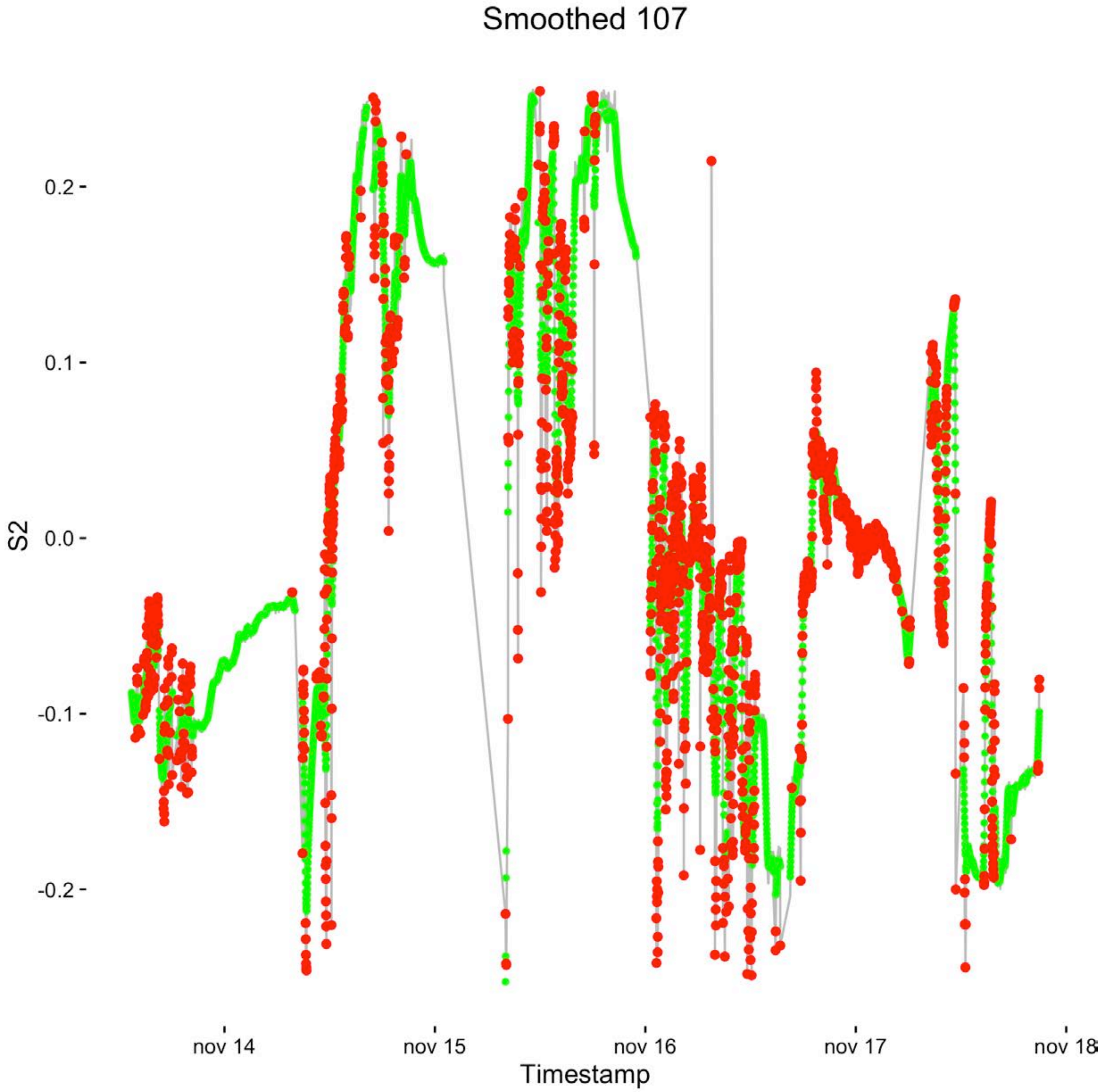
Smoothed 107
S2
0.2
0.1
0.0
-0.1
-0.2
nov 14
nov 15
nov 16
nov 17
nov 18
Timestamp

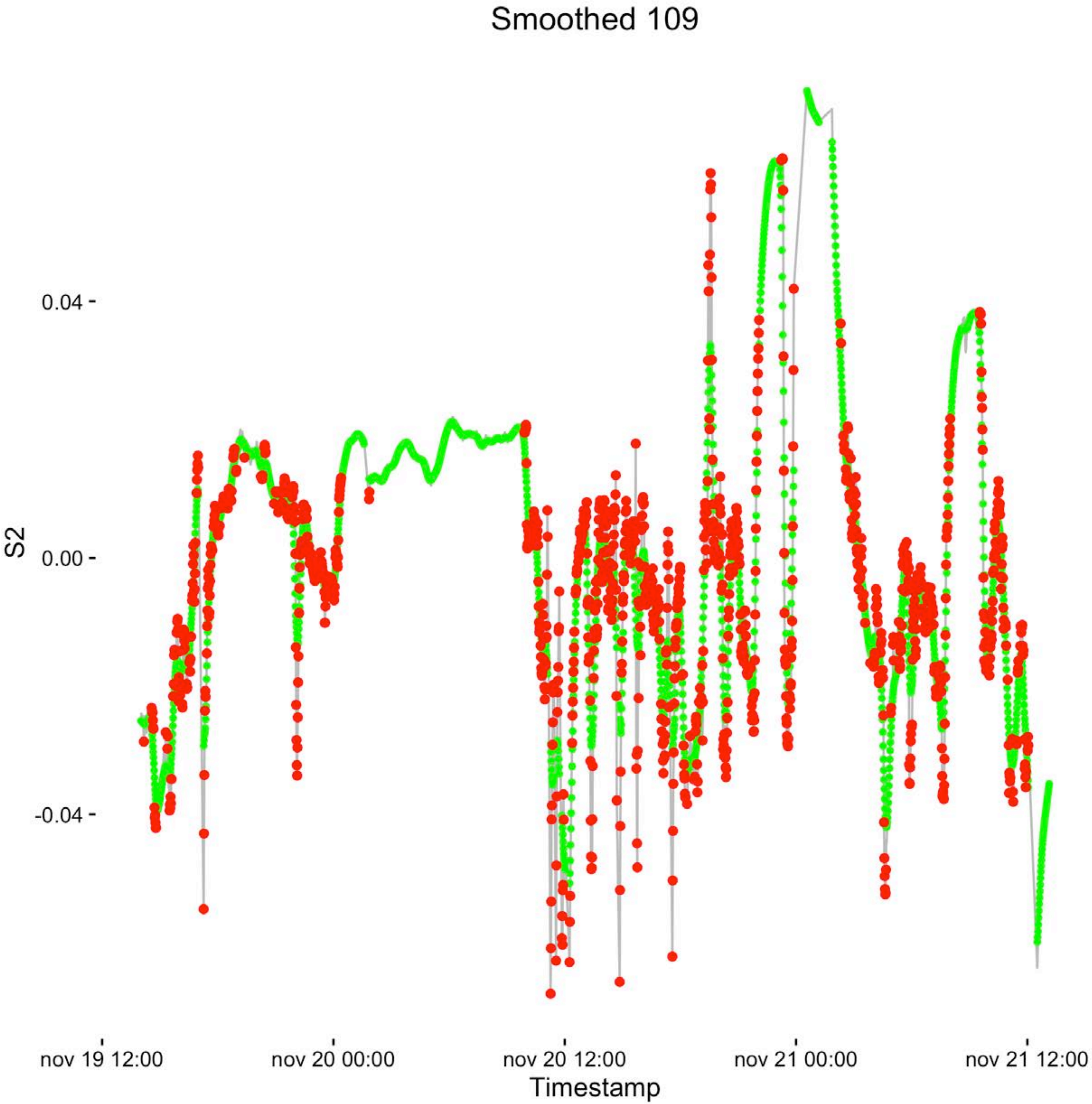
Smoothed 109
S2
0.04
0.00
-0.04
nov 19 12:00
nov 20 00:00
nov 20 12:00
nov 21 00:00
nov 21 12:00
Timestamp

Smoothed 1010

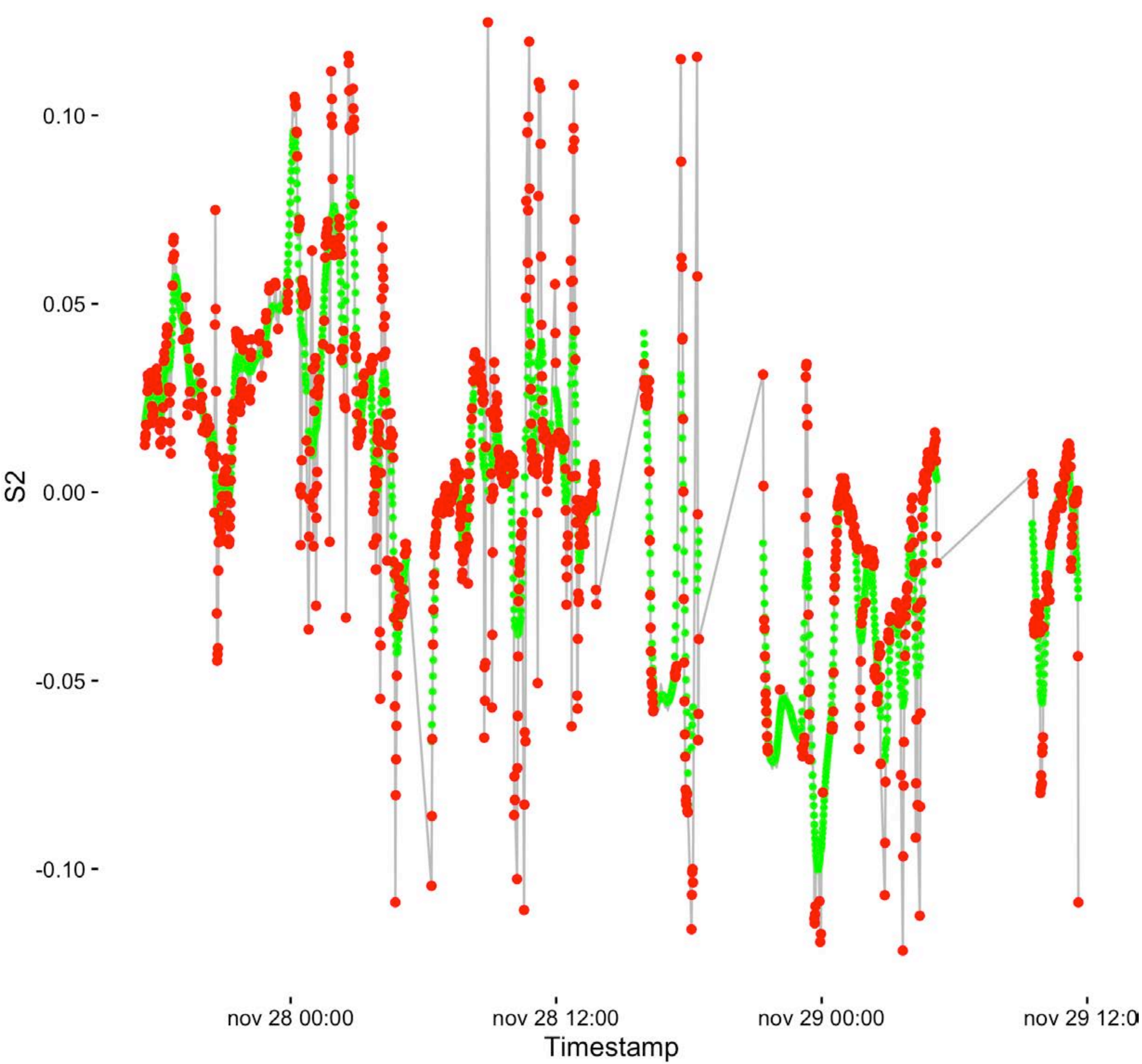

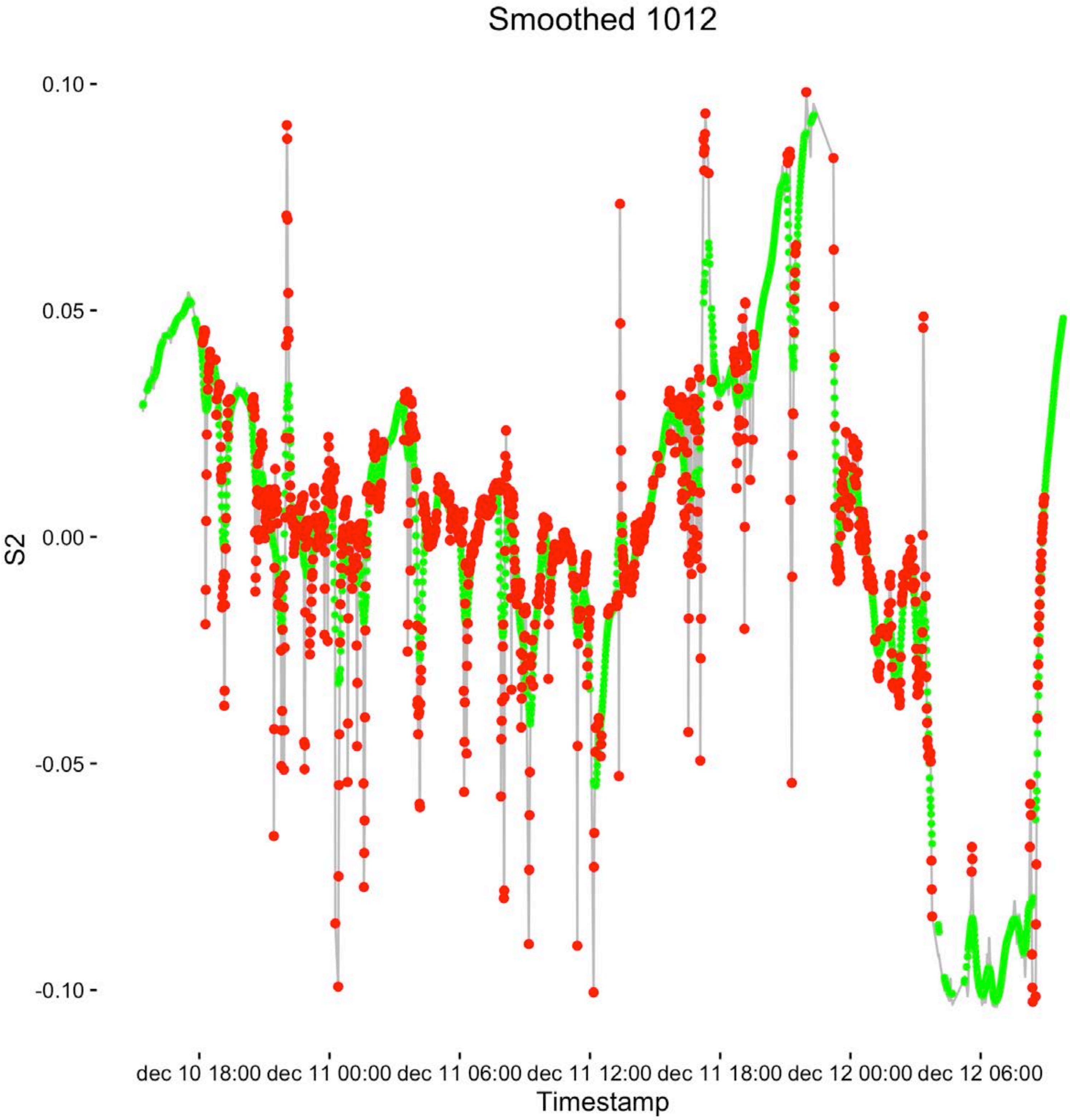
Smoothed 1012
0.10
0.05
0.00
-0.05
-0.10
S2
dec 10 18:00
dec 11 00:00
dec 11 06:00
dec 11 12:00
dec 11 18:00
dec 12 00:00
dec 12 06:00
Timestamp

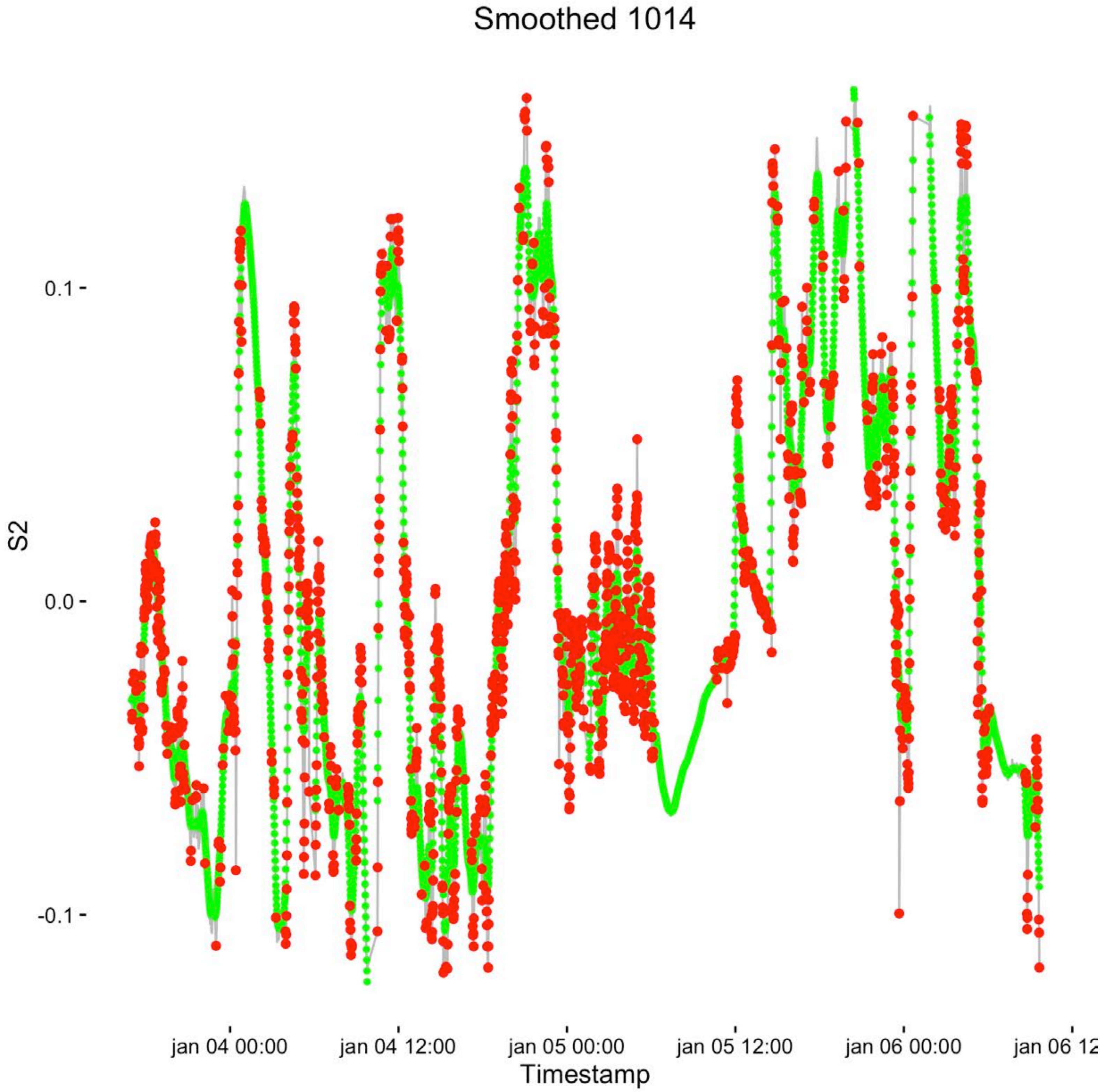

Smoothed 1014
0.1
0.0
-0.1
S2
jan 04 00:00
jan 04 12:00
jan 05 00:00
jan 05 12:00
jan 06 00:00
jan 06 12
Timestamp

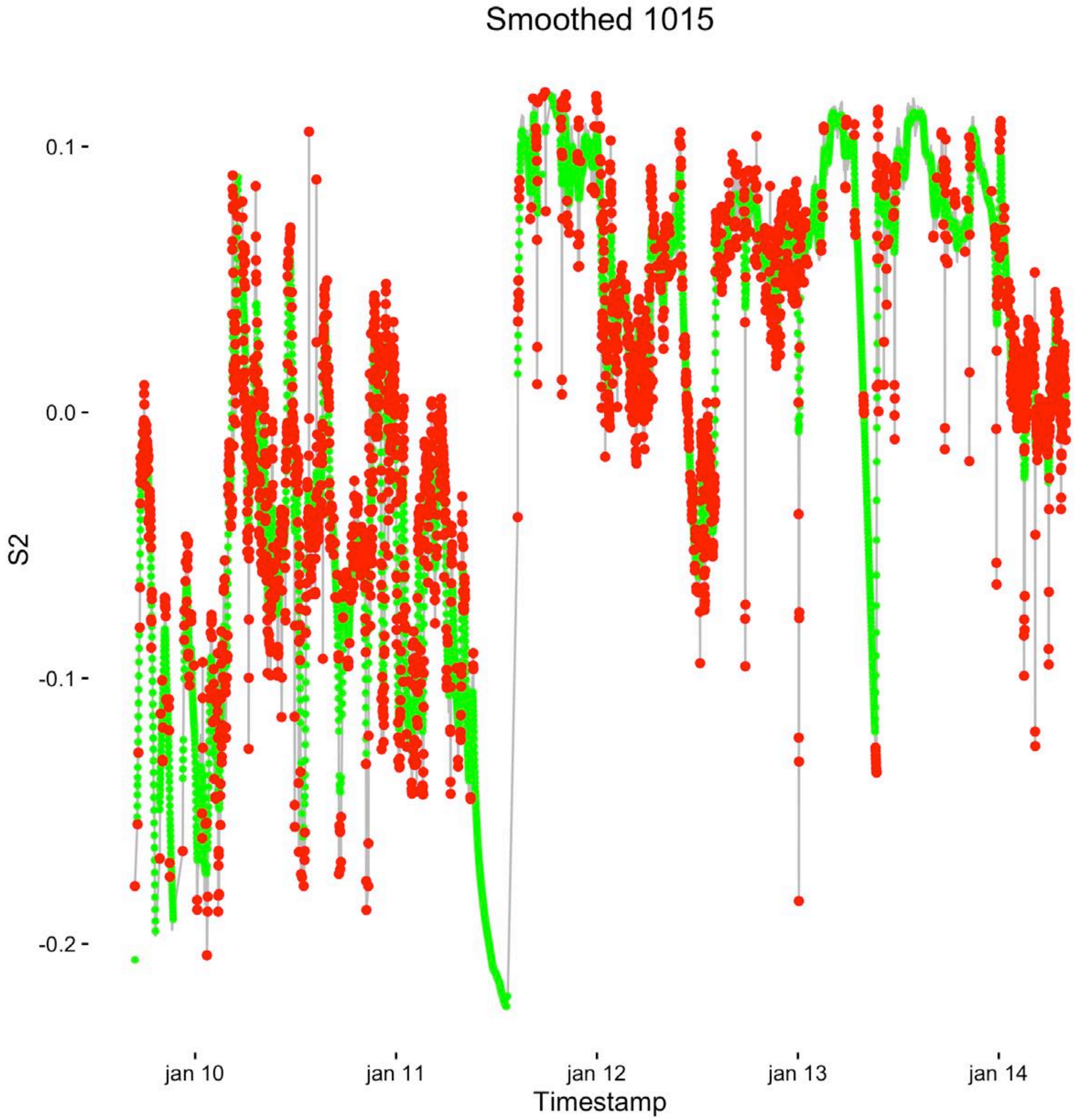

Smoothed 1015
0.1
0.0
-0.1
-0.2
S2
jan 10
jan 11
jan 12
jan 13
jan 14
Timestamp

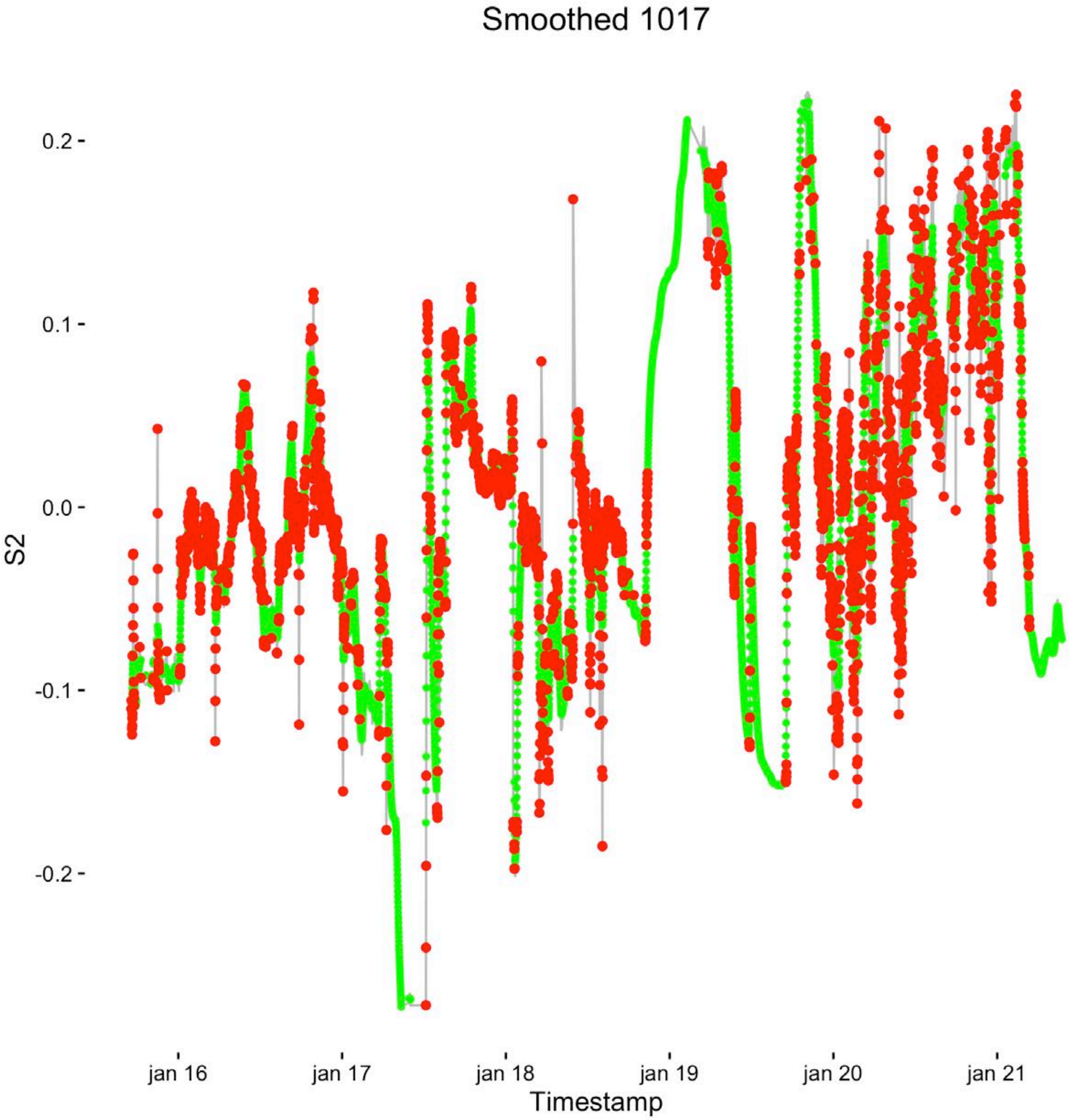

Smoothed 1017
S2
0.2
0.1
0.0
-0.1
-0.2
jan 16
jan 17
jan 18
jan 19
jan 20
jan 21
Timestamp

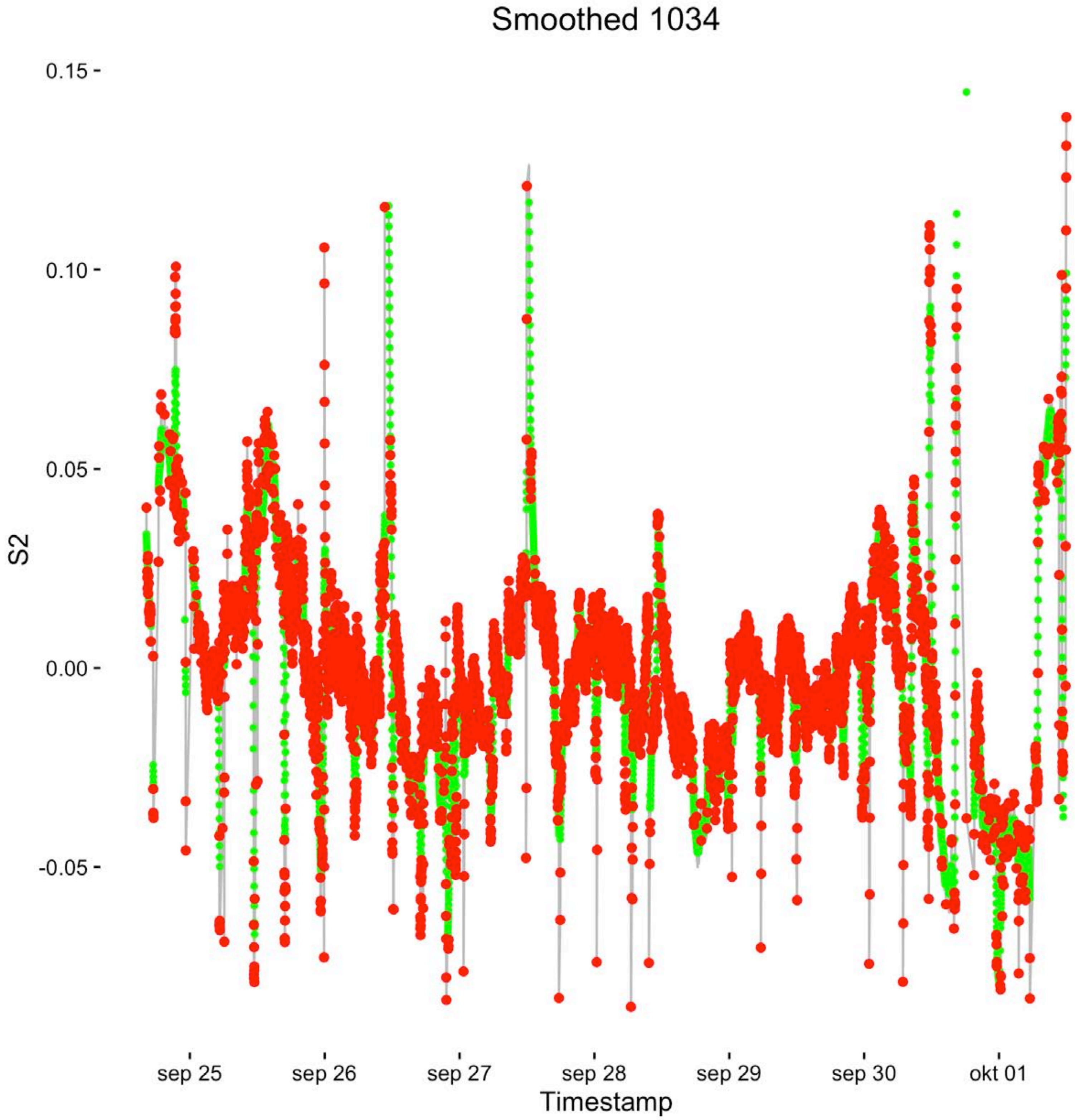
Smoothed 1034
0.15
0.10
0.05
0.00
-0.05
S2
sep 25
sep 26
sep 27
sep 28
sep 29
sep 30
okt 01
Timestamp

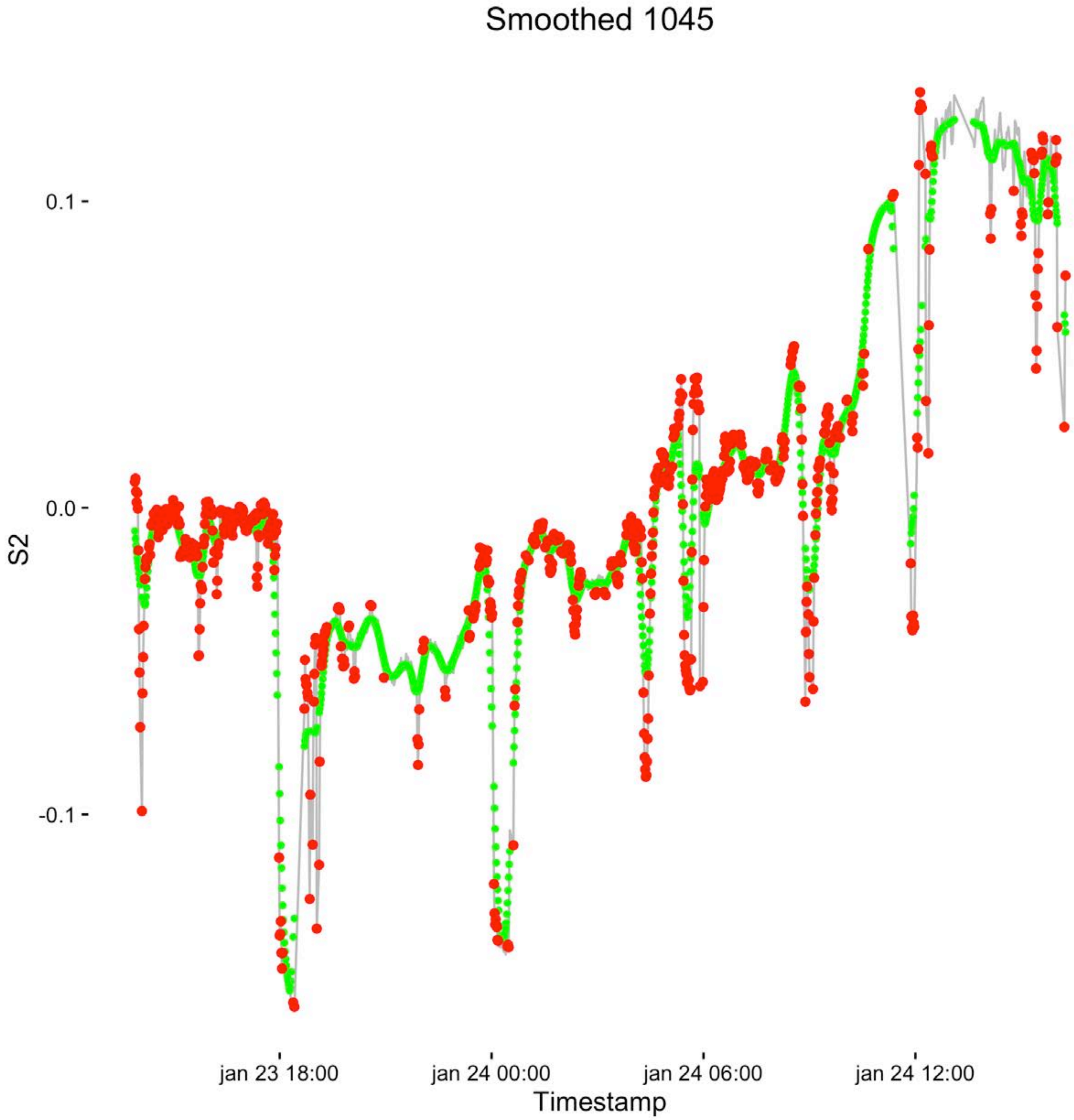
Smoothed 1045
0.1
0.0
-0.1
S2
jan 23 18:00
jan 24 00:00
jan 24 06:00
jan 24 12:00
Timestamp

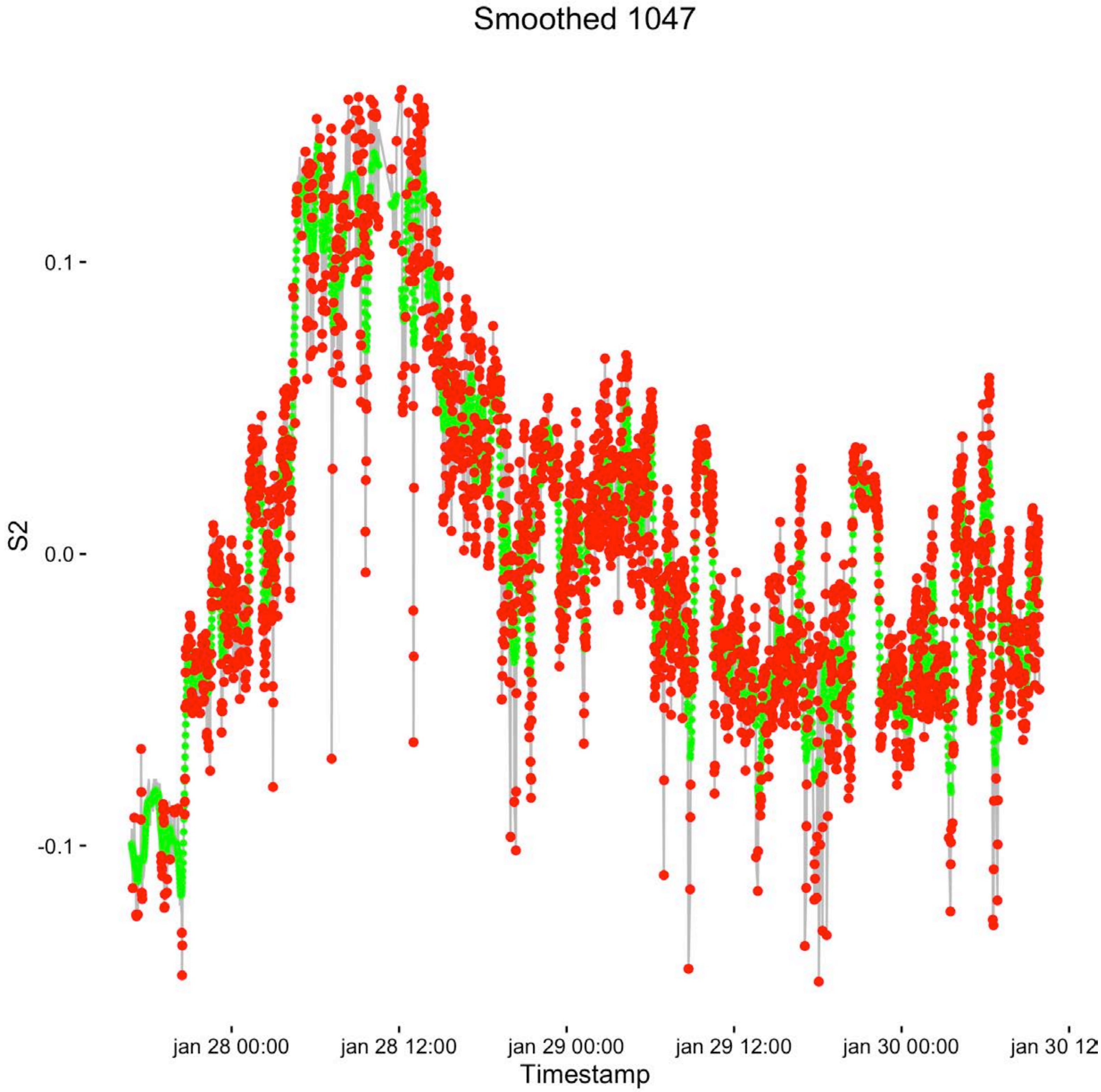
Smoothed 1047
S2
0.1
0.0
-0.1
jan 28 00:00
jan 28 12:00
jan 29 00:00
jan 29 12:00
jan 30 00:00
jan 30 12
Timestamp

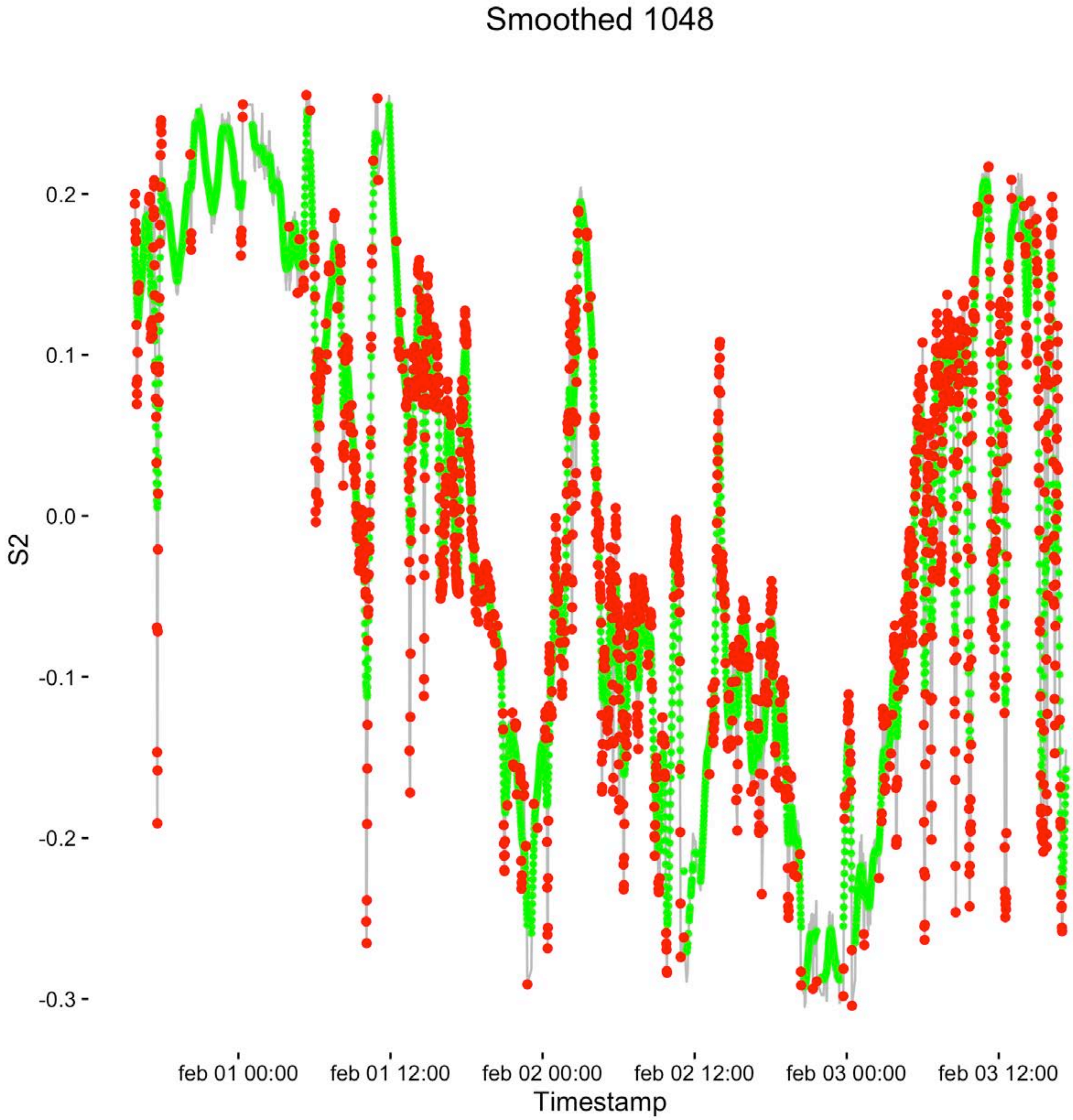

Smoothed 1048
0.2
0.1
0.0
-0.1
-0.2
-0.3
S2
feb 01 00:00
feb 01 12:00
feb 02 00:00
feb 02 12:00
feb 03 00:00
feb 03 12:00
Timestamp

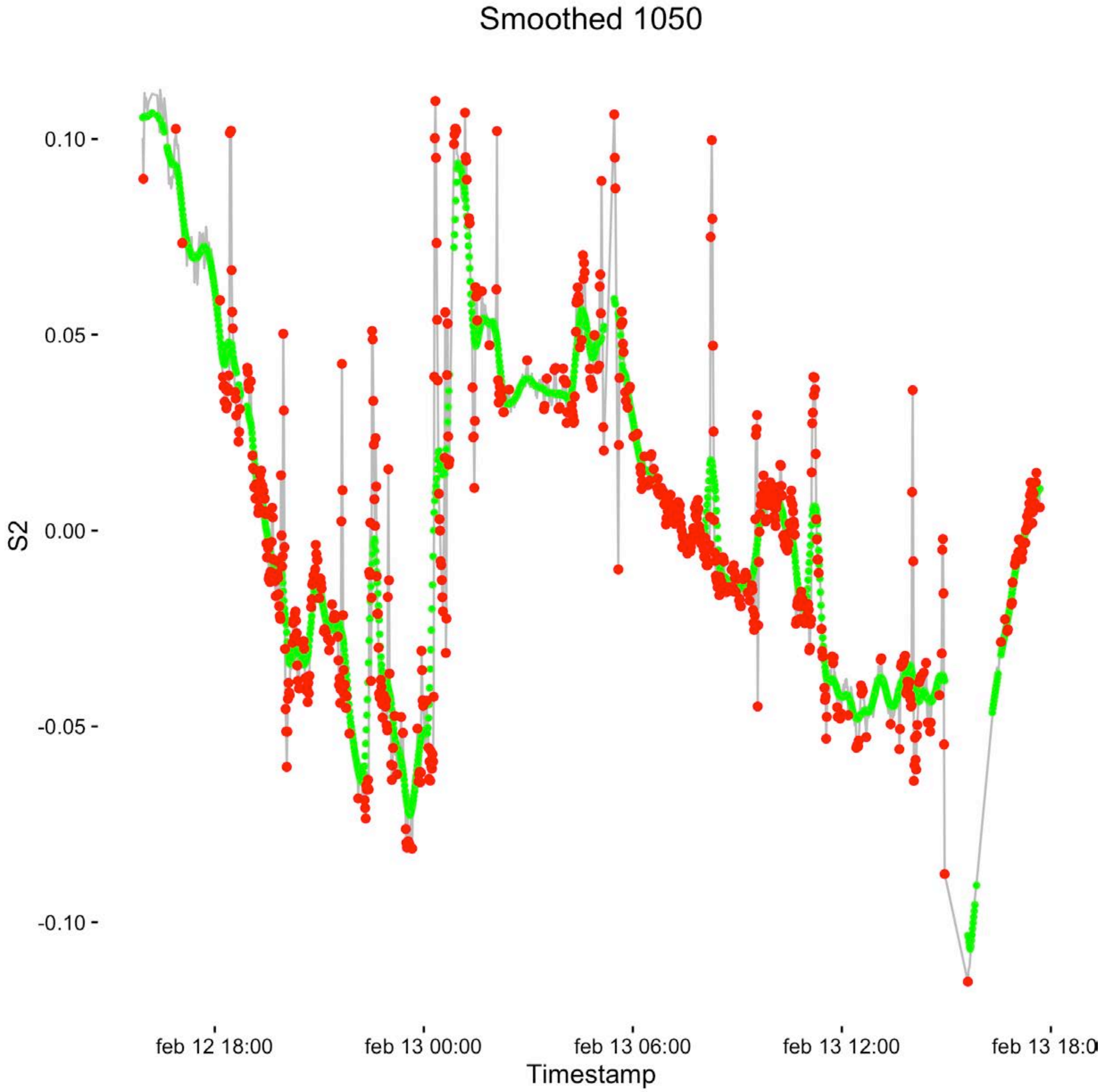

Smoothed 1050
S2
0.10
0.05
0.00
-0.05
-0.10
feb 12 18:00
feb 13 00:00
feb 13 06:00
feb 13 12:00
feb 13 18:0
Timestamp

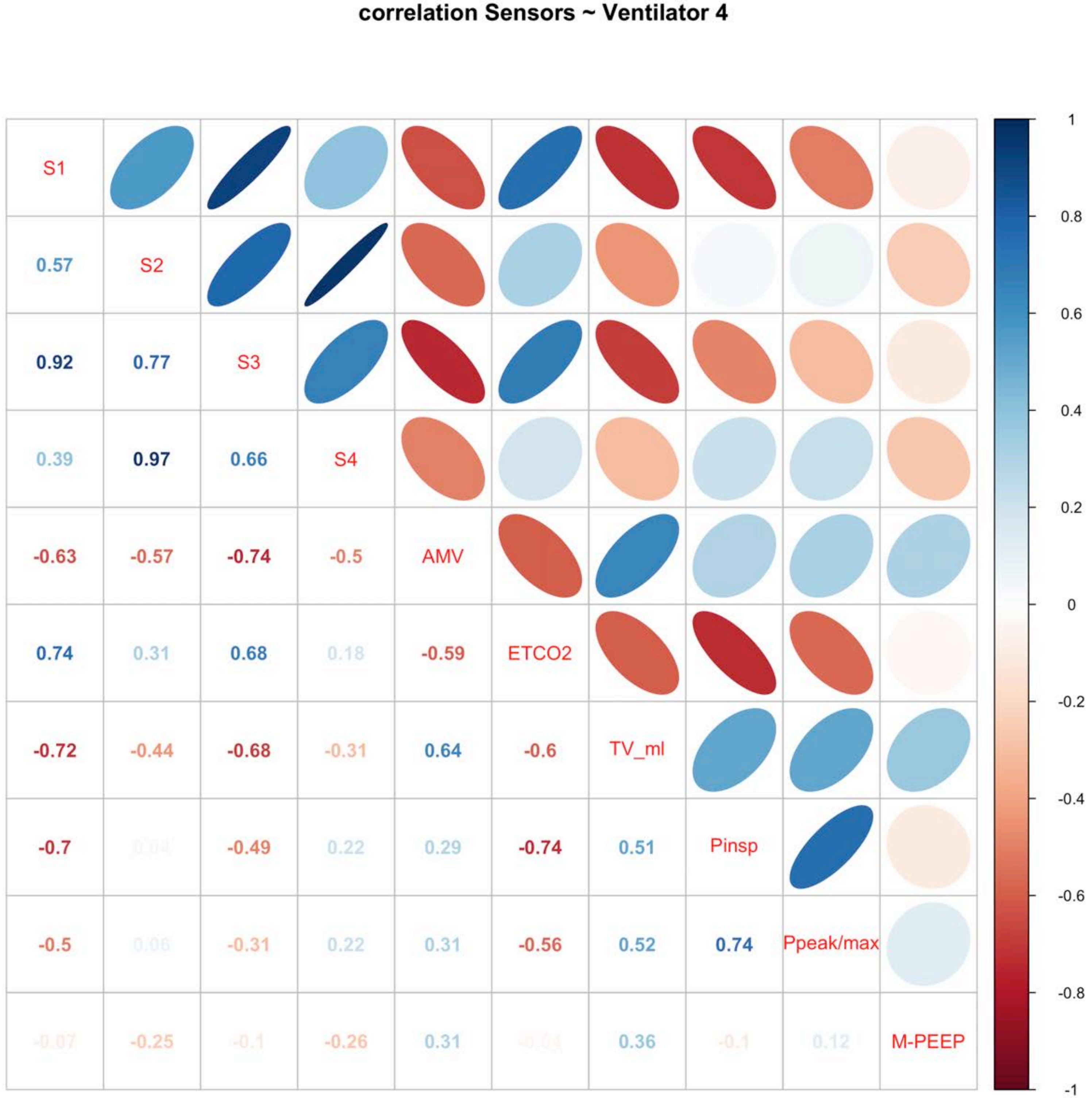

correlation Sensors ~ Ventilator 4
S1
S2
S3
S4
AMV
ETCO2
TV_ml
Pinsp
Ppeak/max
M-PEEP
0.57
0.92 0.77
0.39 0.97 0.66
-0.63 -0.57 -0.74 -0.5
0.74 0.31 0.68 0.18 -0.59
-0.72 -0.44 -0.68 -0.31 0.64 -0.6
-0.7 0.04 -0.49 0.22 0.29 -0.74 0.51
-0.5 0.06 -0.31 0.22 0.31 -0.56 0.52 0.74
-0.07 -0.25 -0.1 -0.26 0.31 -0.04 0.36 -0.1 0.12
1
0.8
0.6
0.4
0.2
0
-0.2
-0.4
-0.6
-0.8
-1

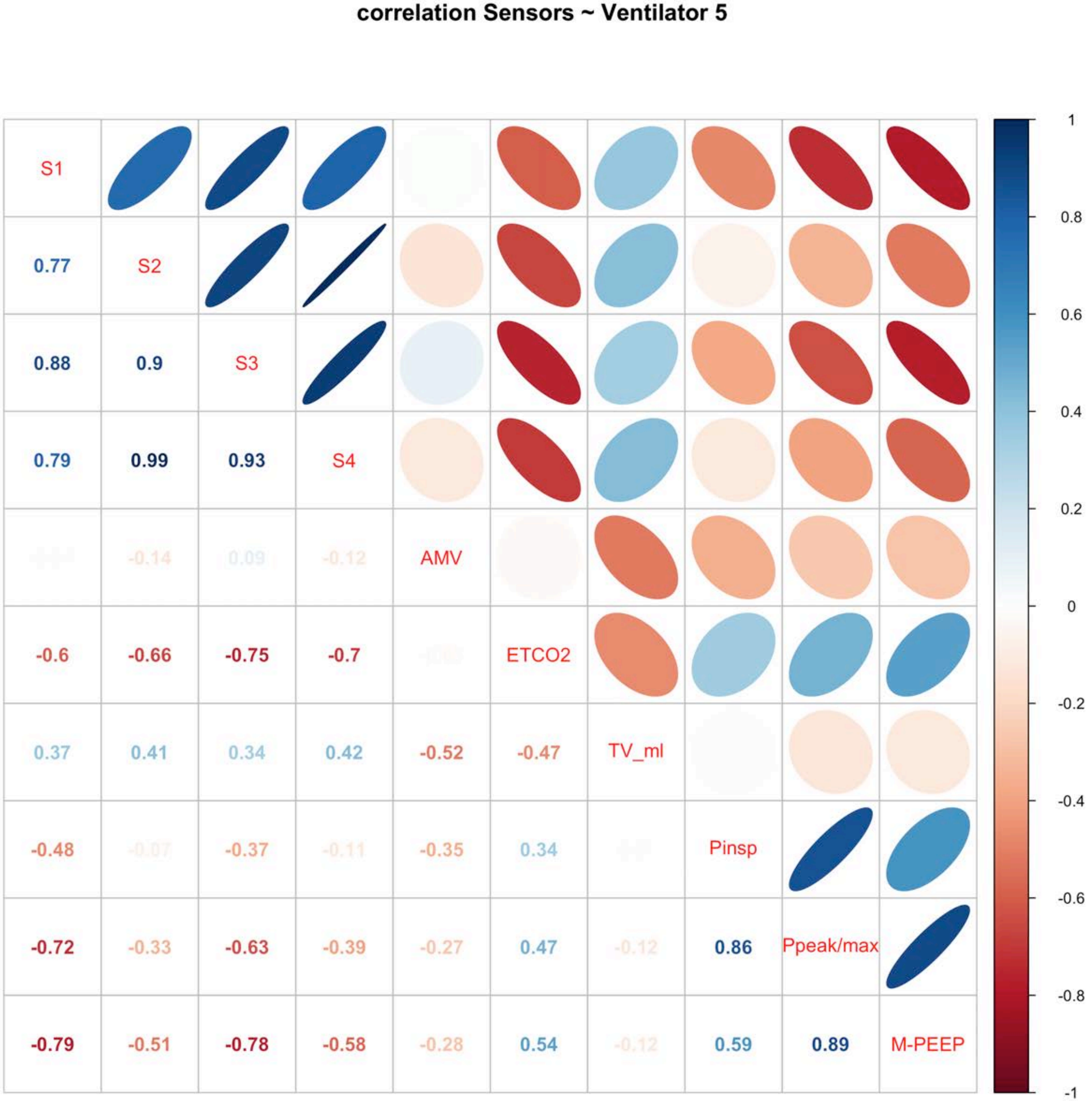
correlation Sensors ~ Ventilator 5
S1
S2
S3
S4
AMV
ETCO2
TV_ml
Pinsp
Ppeak/max
M-PEEP
0.77
0.88 0.9
0.79 0.99 0.93
-0.14 0.09 -0.12
-0.6 -0.66 -0.75 -0.7
0.37 0.41 0.34 0.42 -0.52 -0.47
-0.48 -0.07 -0.37 -0.11 -0.35 0.34
-0.72 -0.33 -0.63 -0.39 -0.27 0.47 -0.12 0.86
-0.79 -0.51 -0.78 -0.58 -0.28 0.54 -0.12 0.59 0.89
1
0.8
0.6
0.4
0.2
0
-0.2
-0.4
-0.6
-0.8
-1

correlation Sensors ~ Ventilator 6

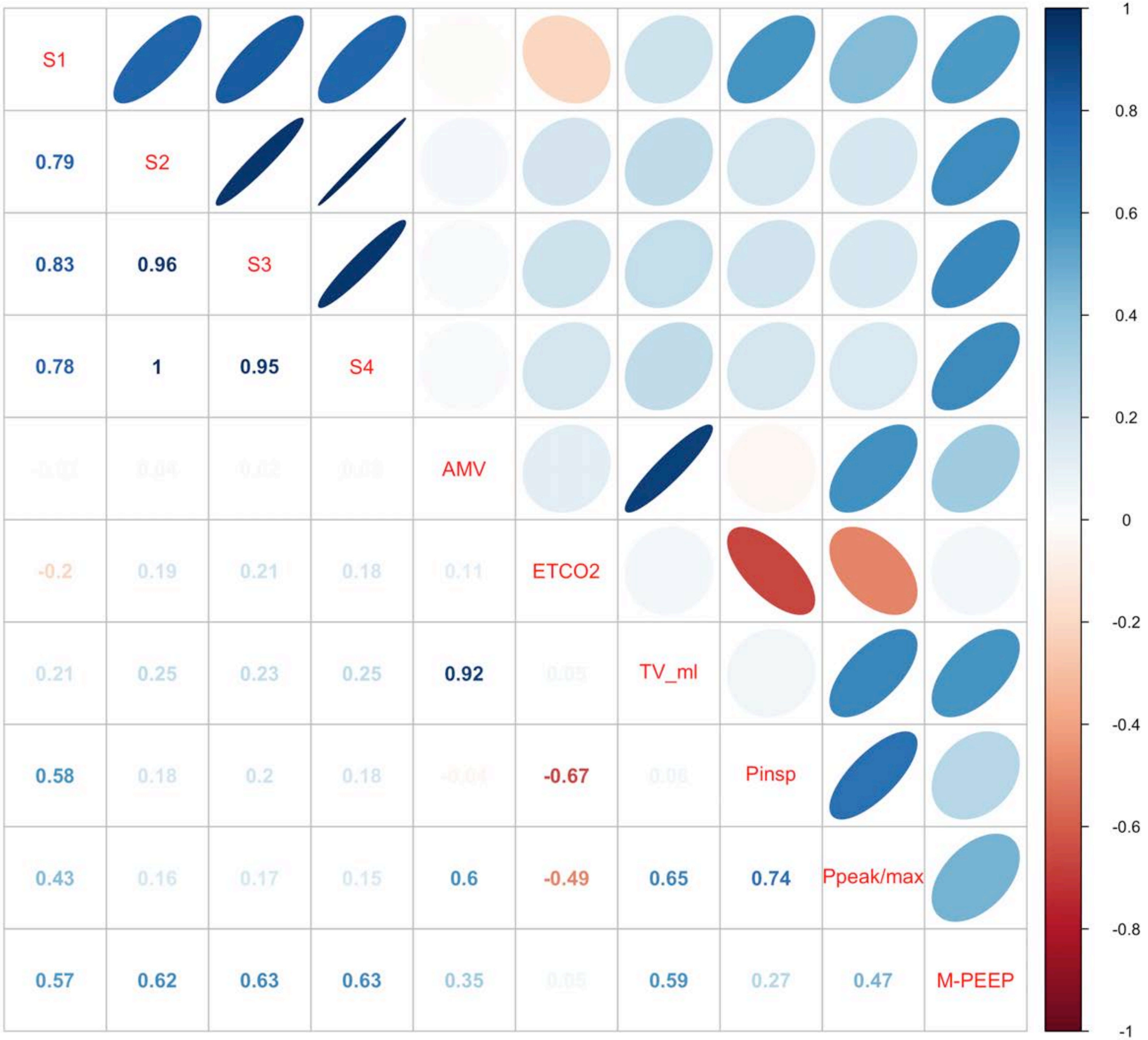

correlation Sensors ~ Ventilator 7

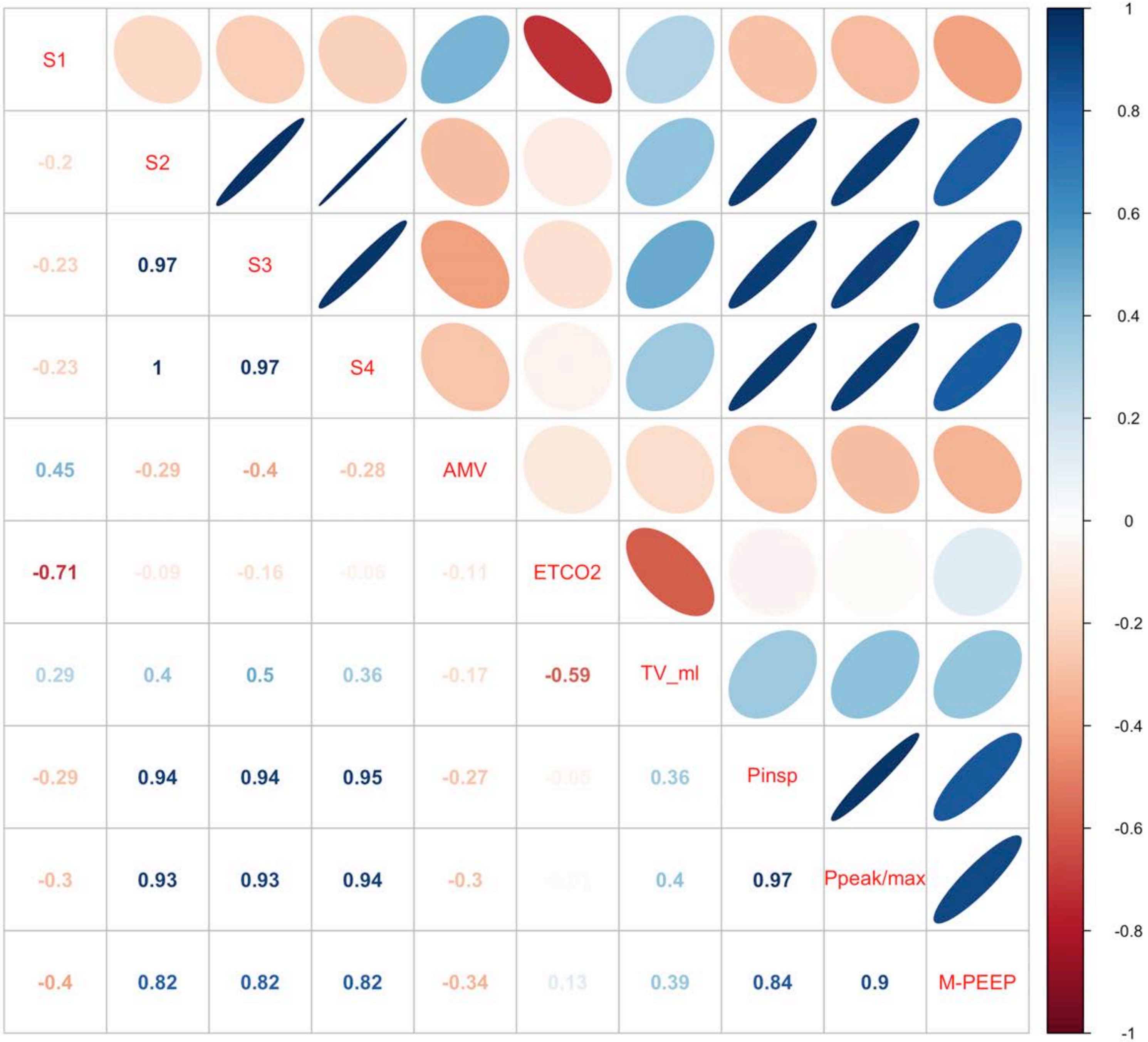

correlation Sensors ~ Ventilator 8

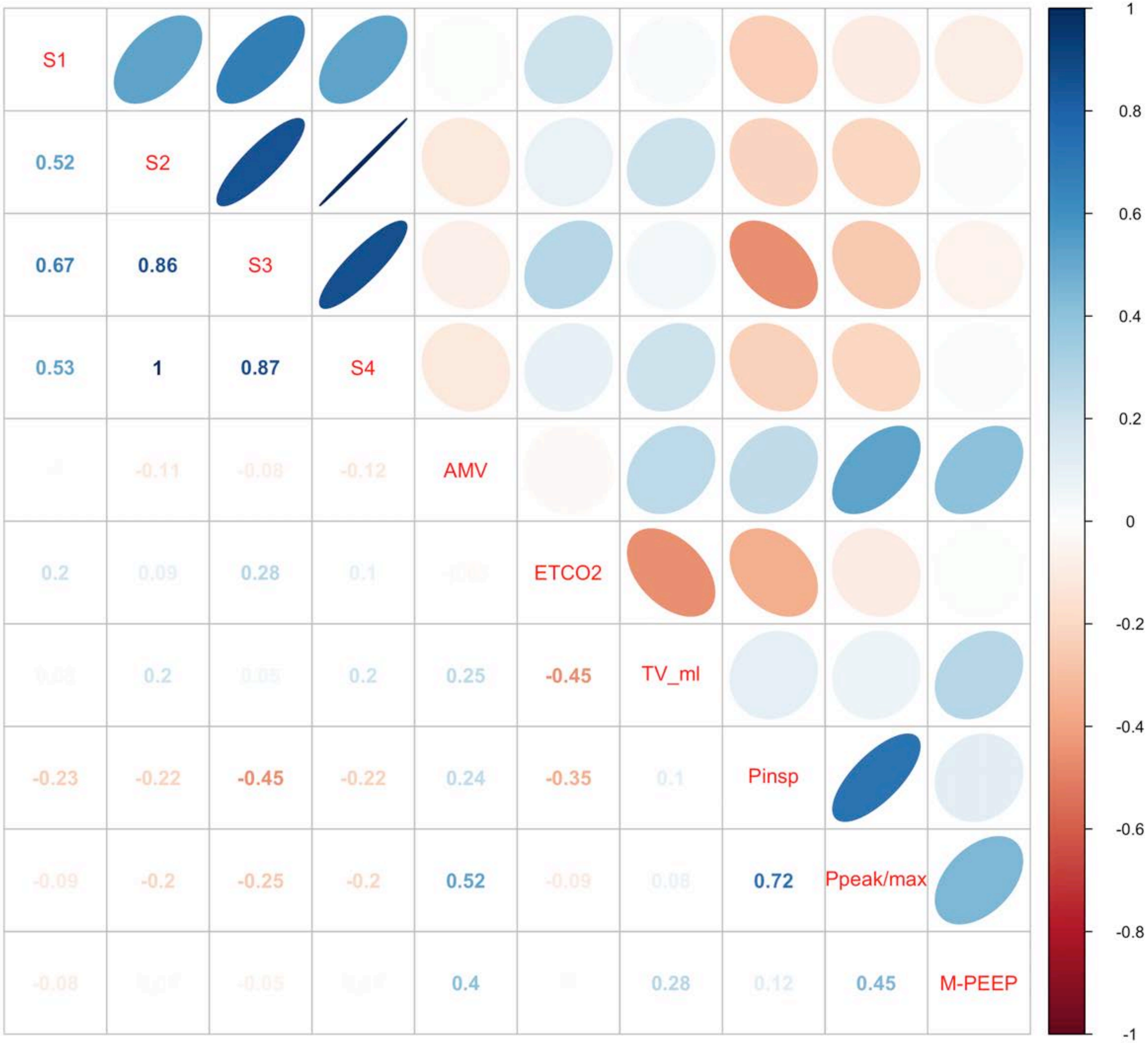

correlation Sensors ~ Ventilator 9

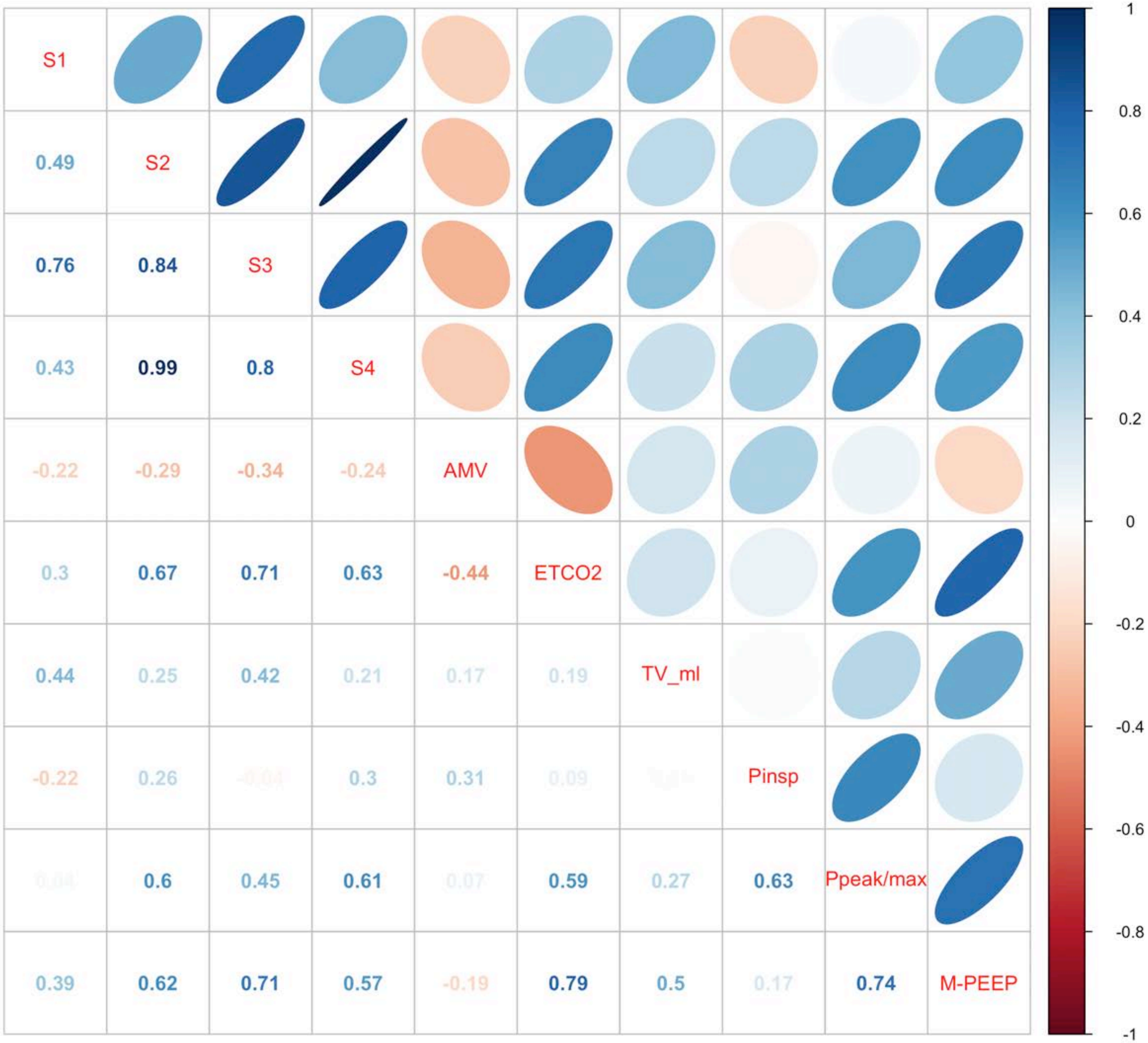

correlation Sensors ~ Ventilator 11

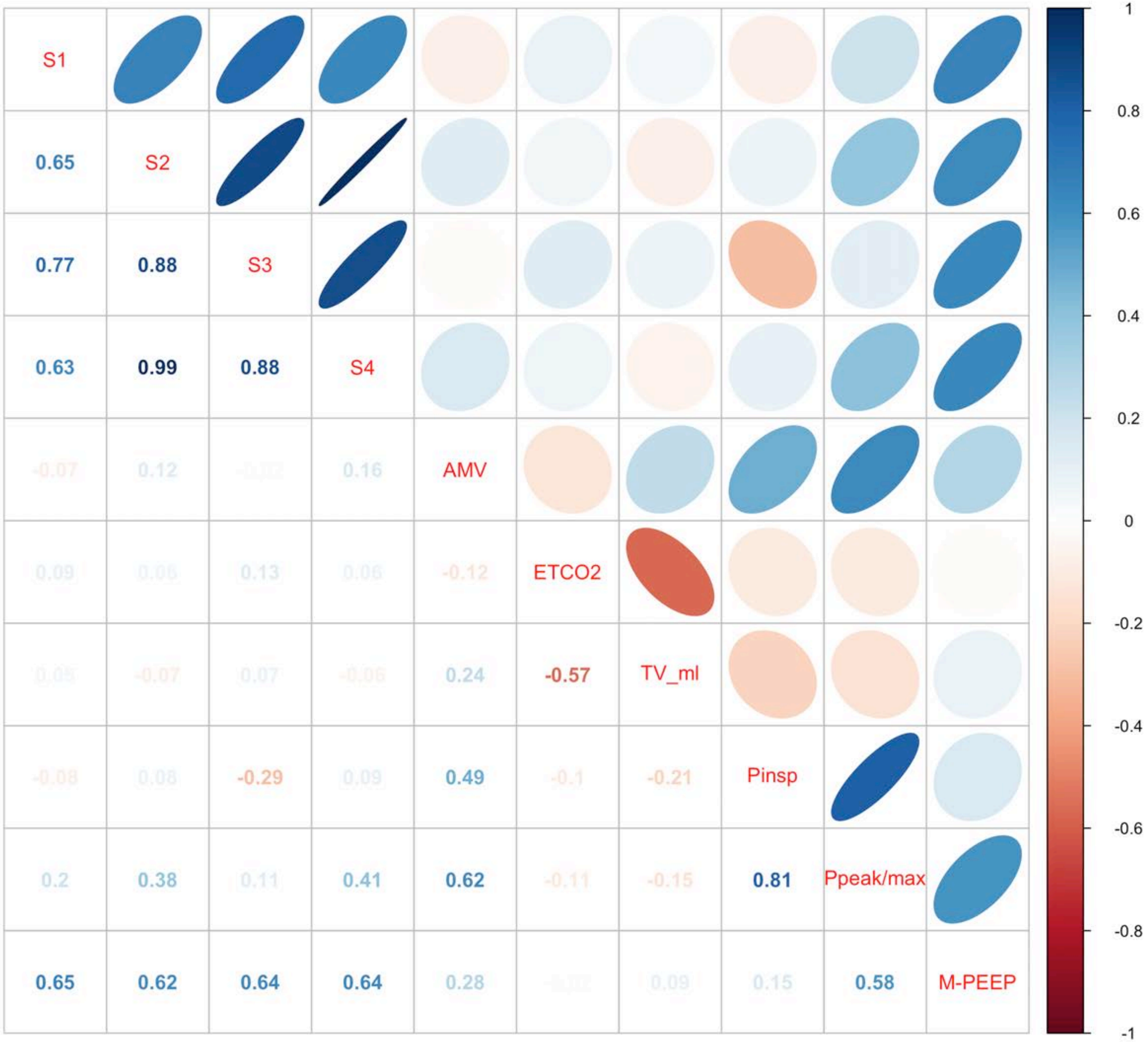

correlation Sensors ~ Ventilator 12

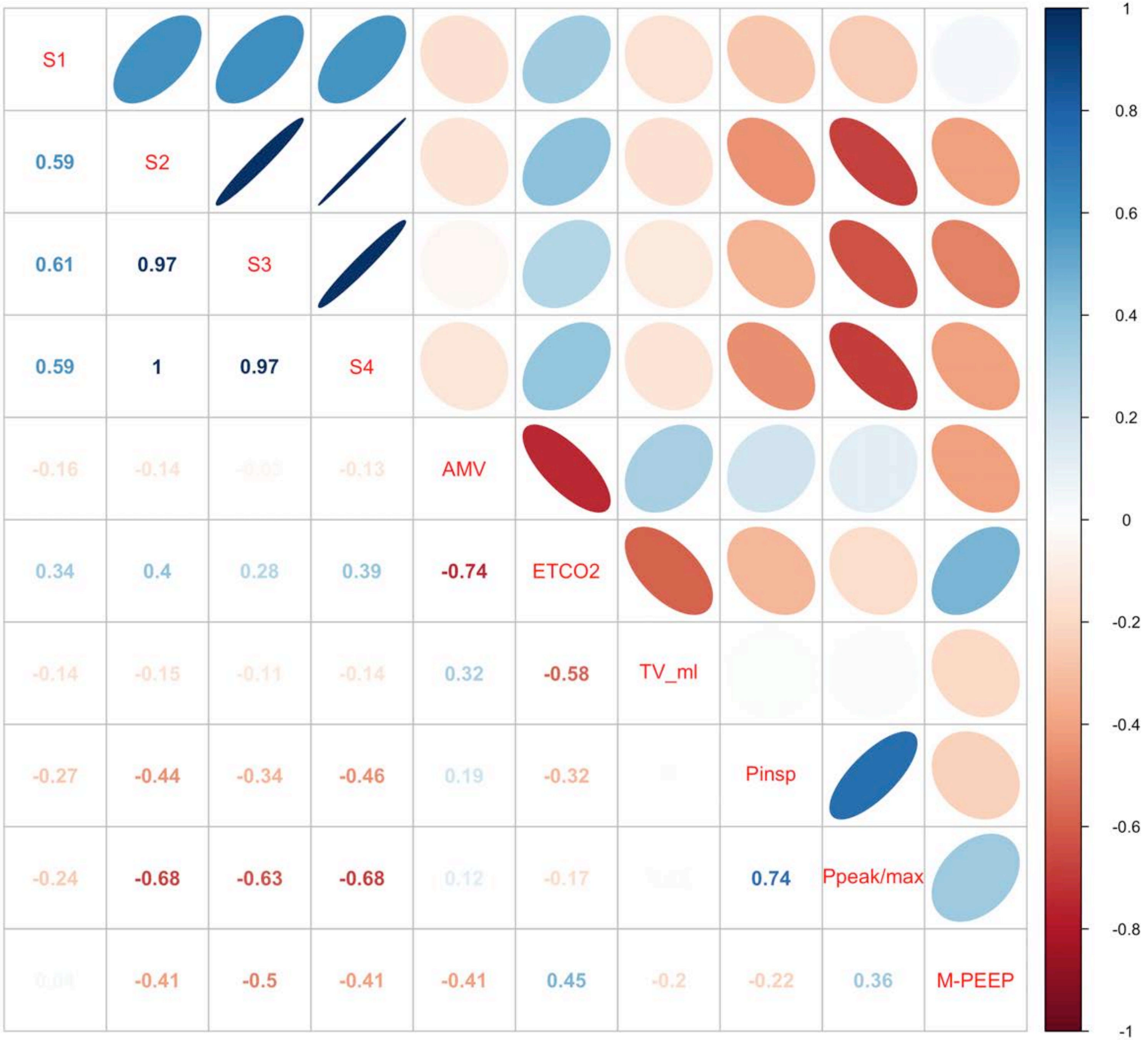

correlation Sensors ~ ventilator 103

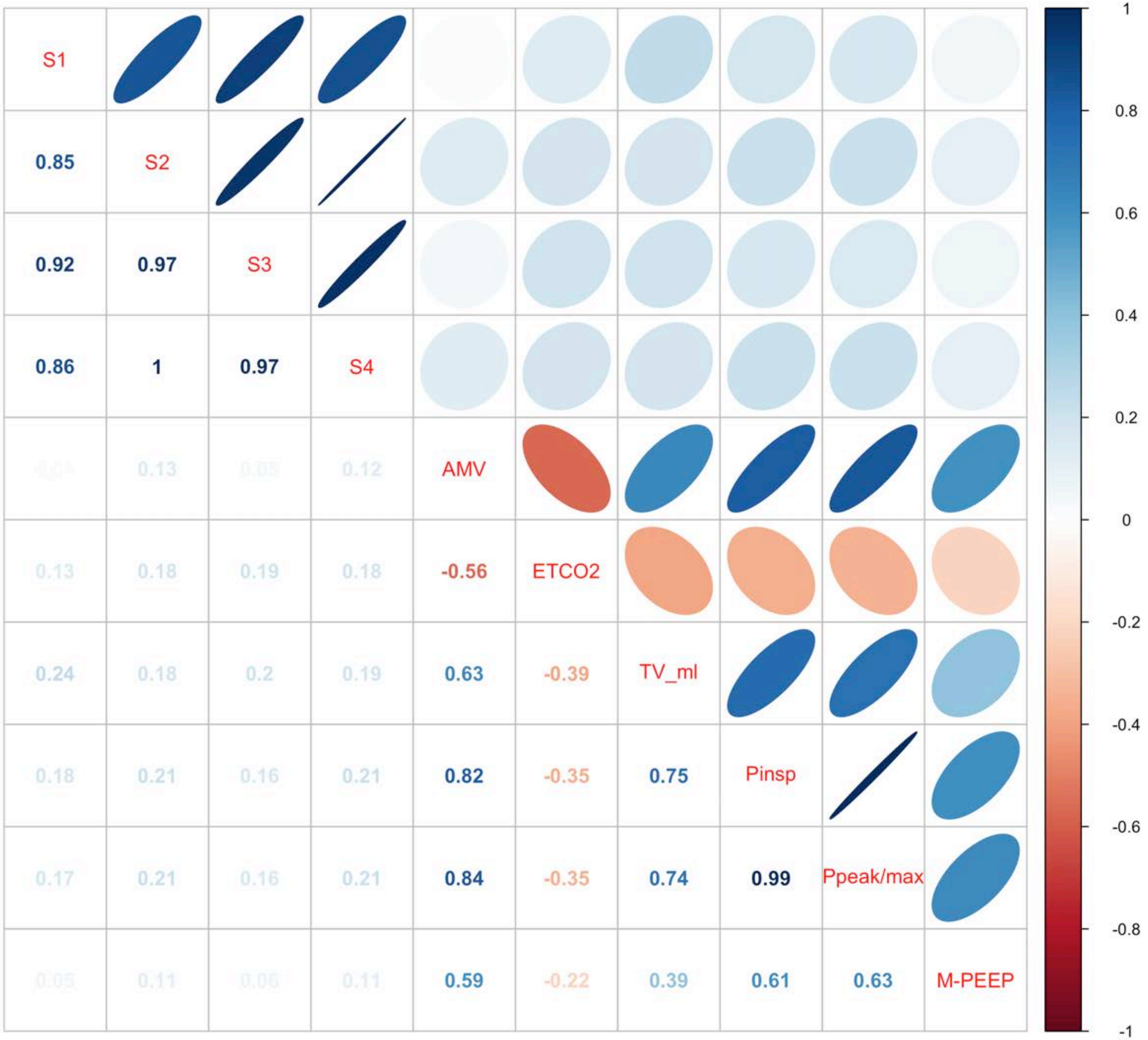

correlation Sensors ~ ventilator 104

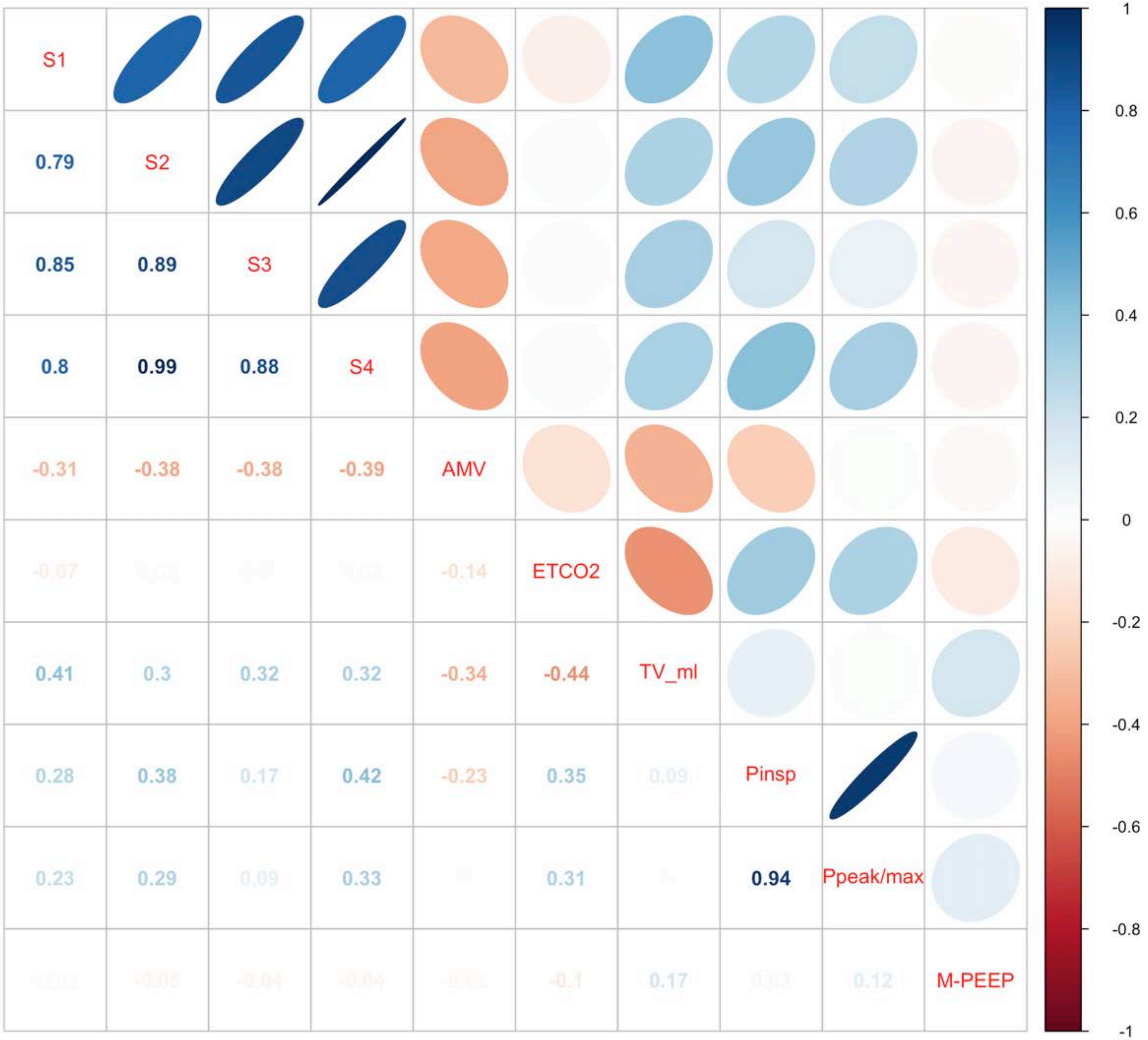

correlation Sensors ~ ventilator 106

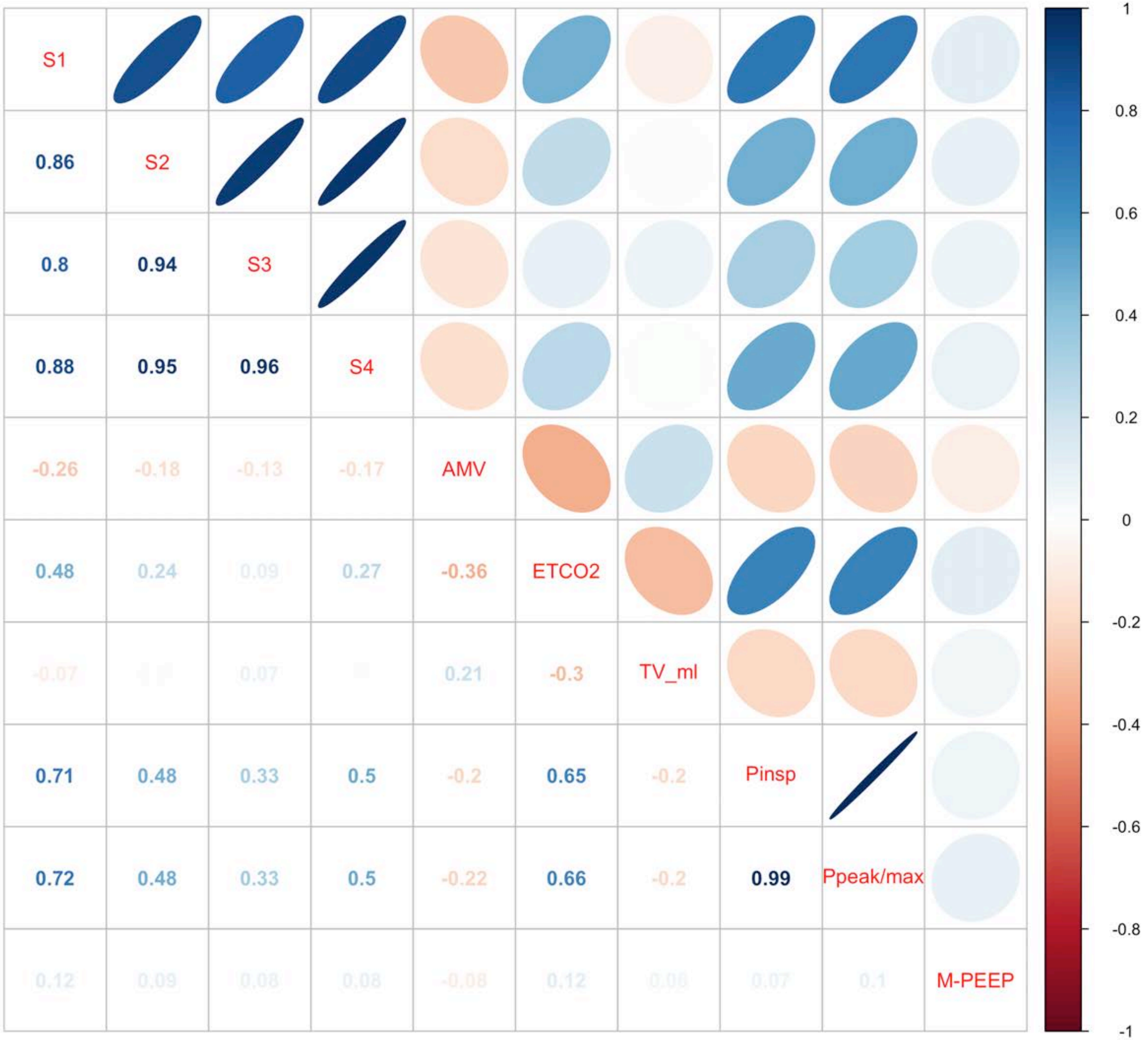

correlation Sensors ~ ventilator 107

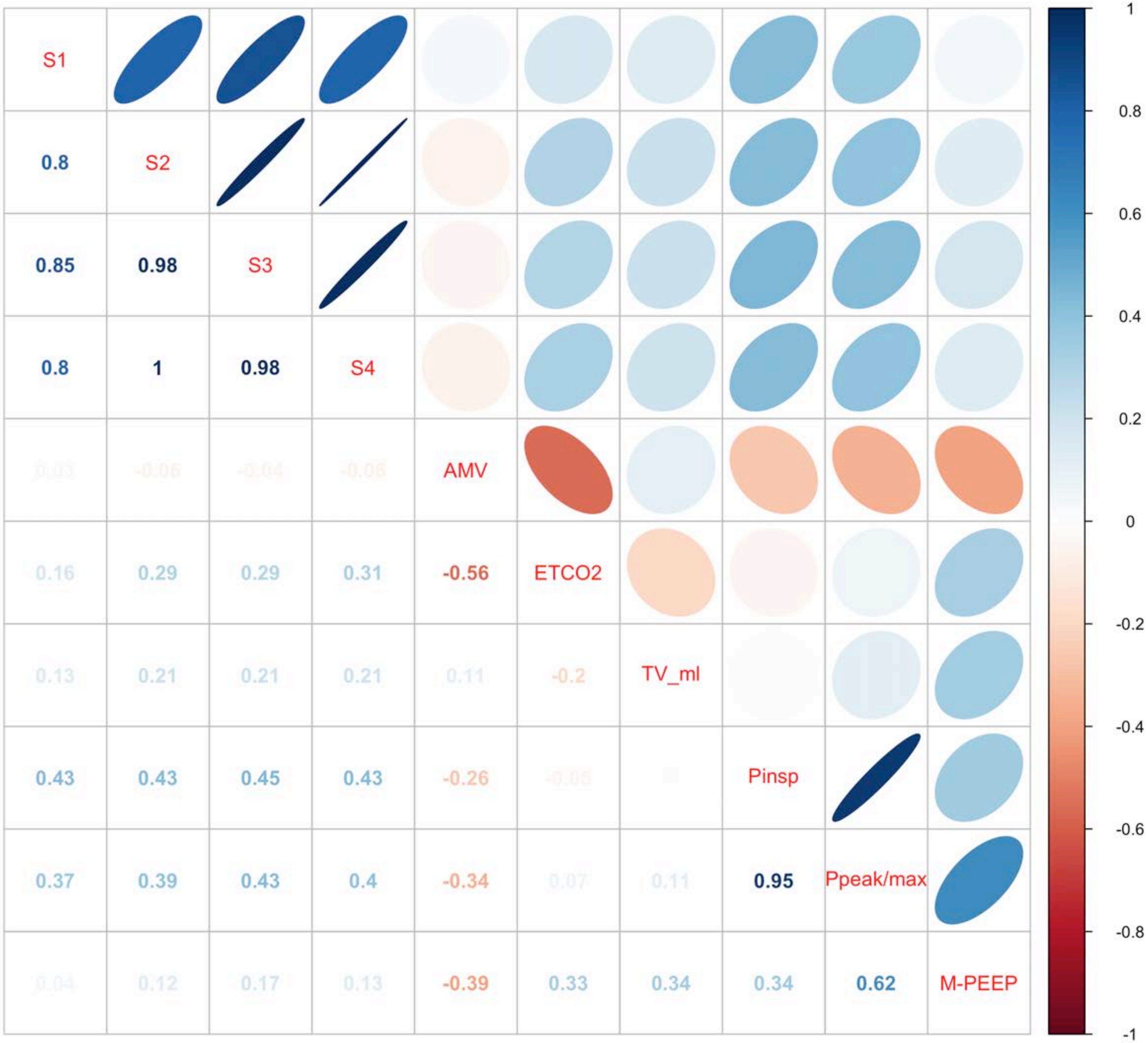

correlation Sensors ~ ventilator 109

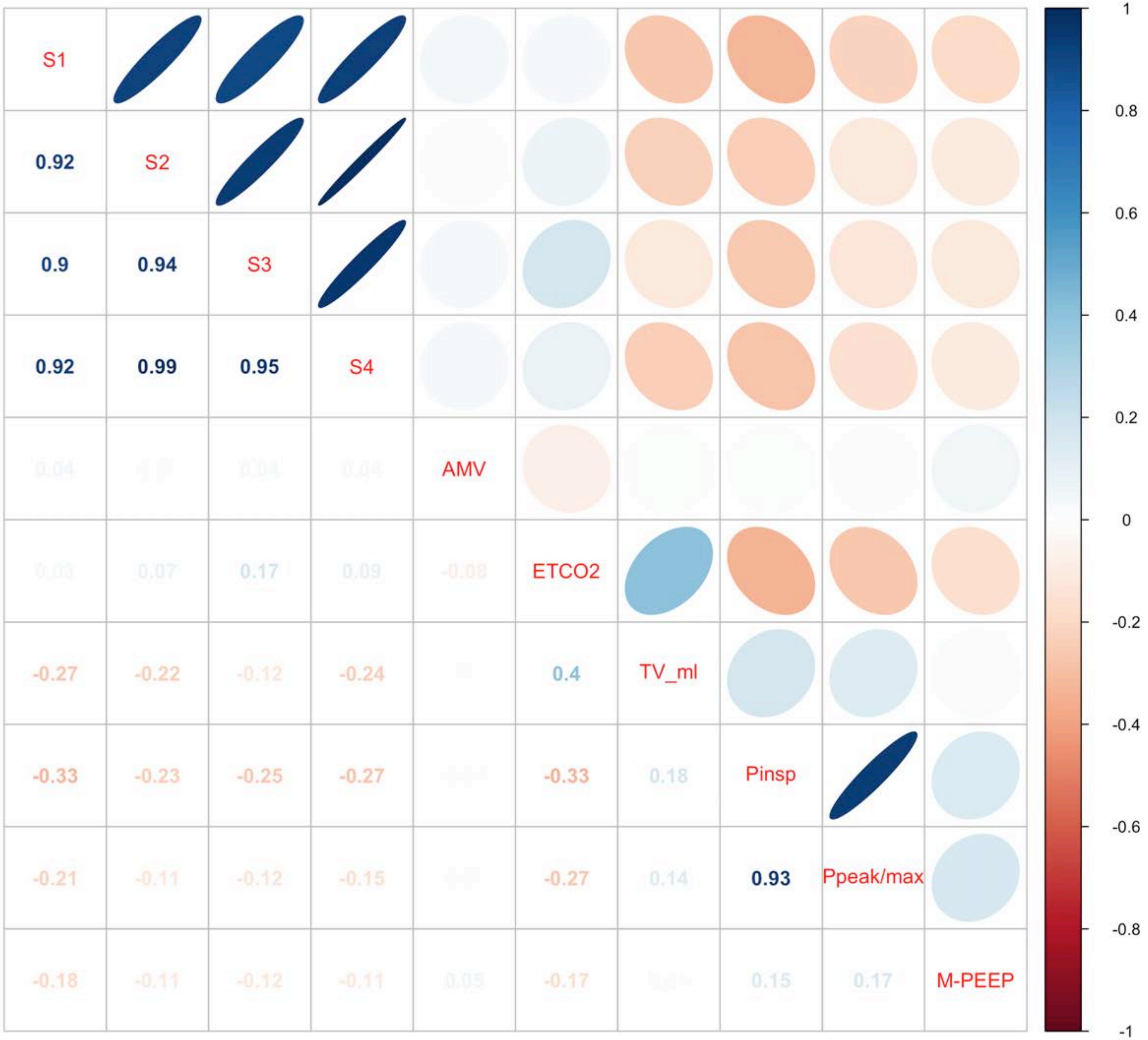

correlation Sensors ~ ventilator 1010

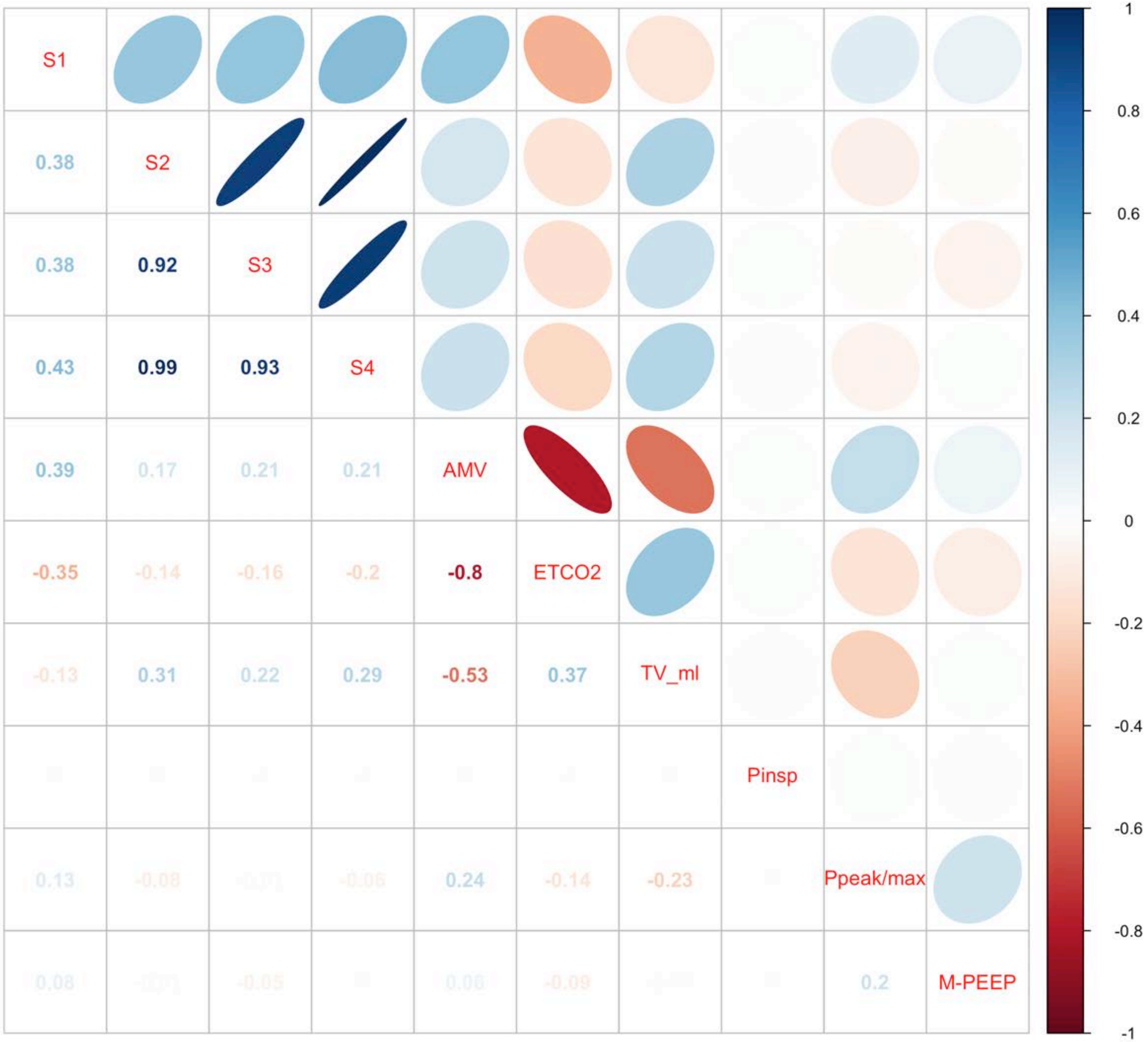

correlation Sensors ~ ventilator 1012

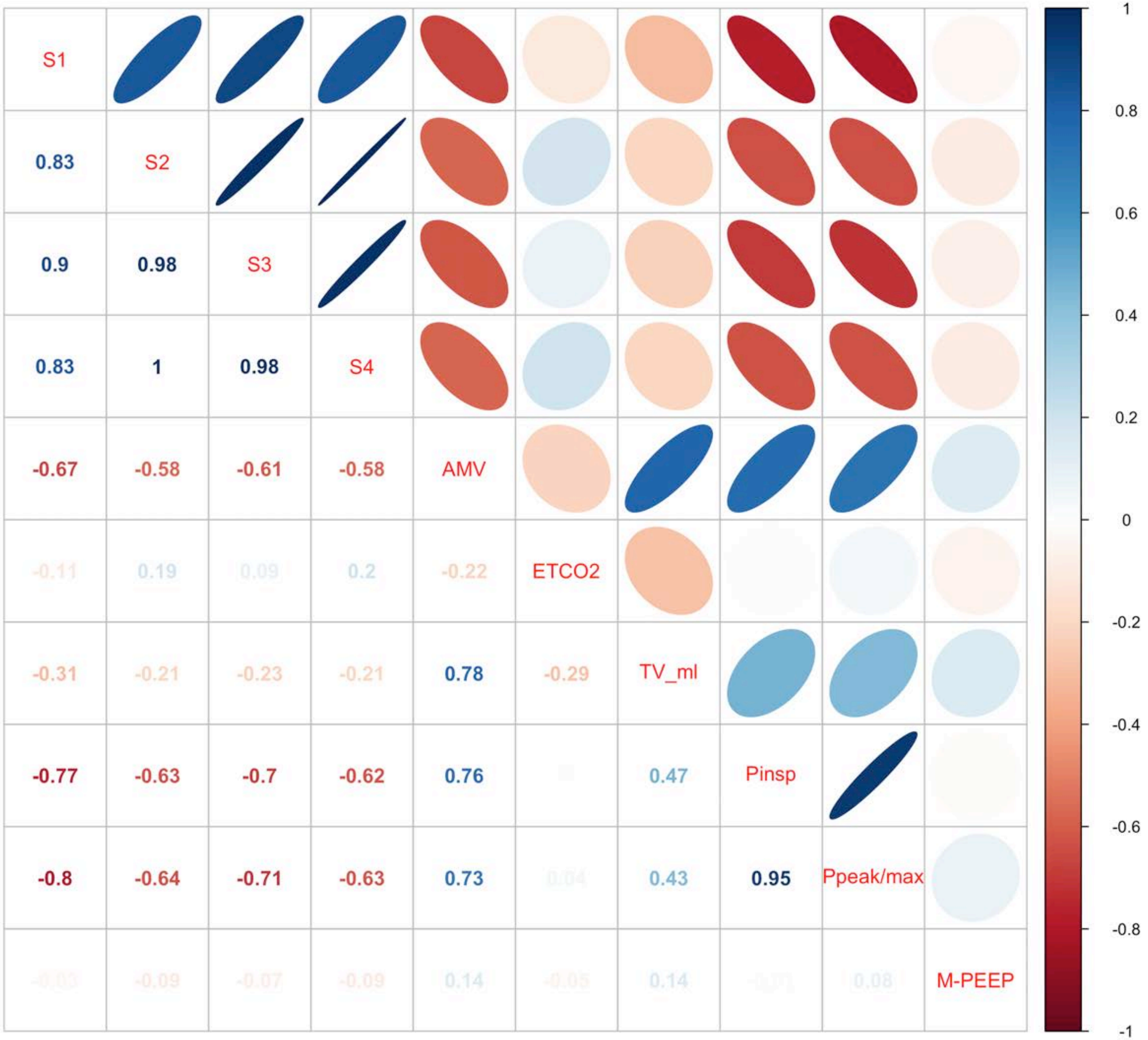

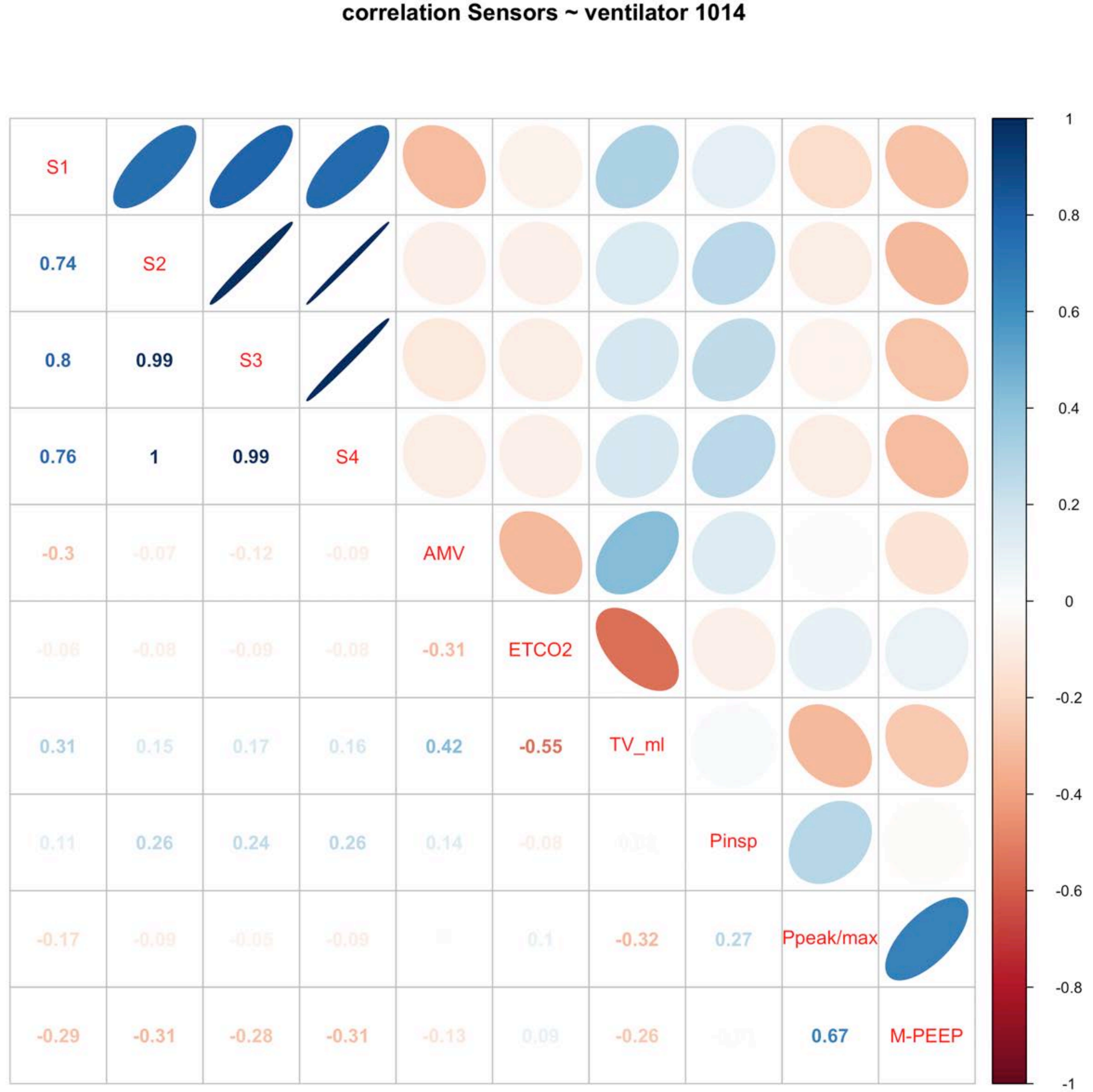
correlation Sensors ~ ventilator 1014
S1
0.74
S2
0.8
0.99
S3
0.76
1
0.99
S4
-0.3
AMV
-0.31
ETCO2
0.31
0.15
0.17
0.16
0.42
-0.55
TV_ml
0.26
0.24
0.26
0.14
Pinsp
-0.17
-0.32
0.27
Ppeak/max
-0.29
-0.31
-0.28
-0.31
-0.13
-0.26
0.67
M-PEEP
1
0.8
0.6
0.4
0.2
0
-0.2
-0.4
-0.6
-0.8
-1

correlation Sensors ~ ventilator 1015

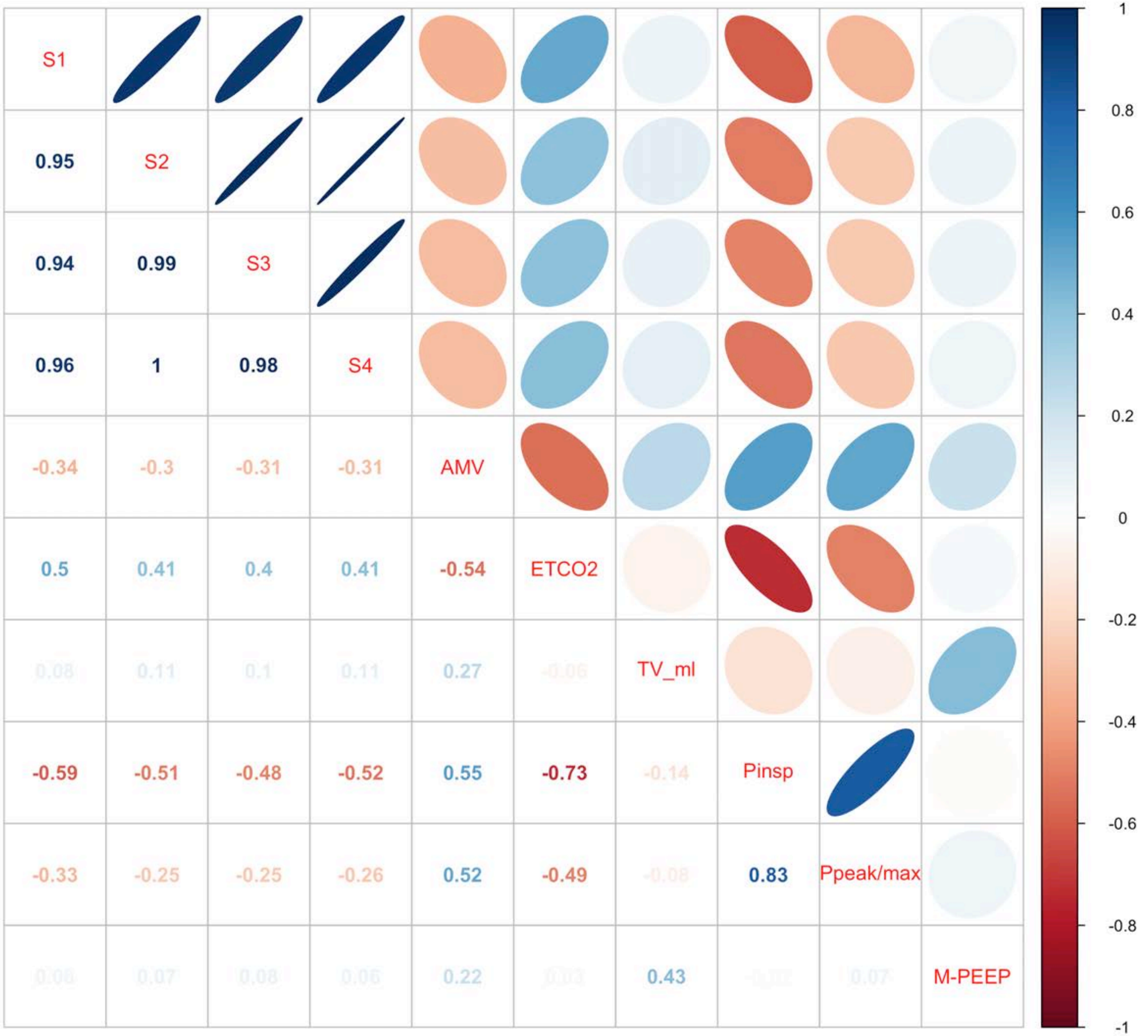

correlation Sensors ~ ventilator 1017

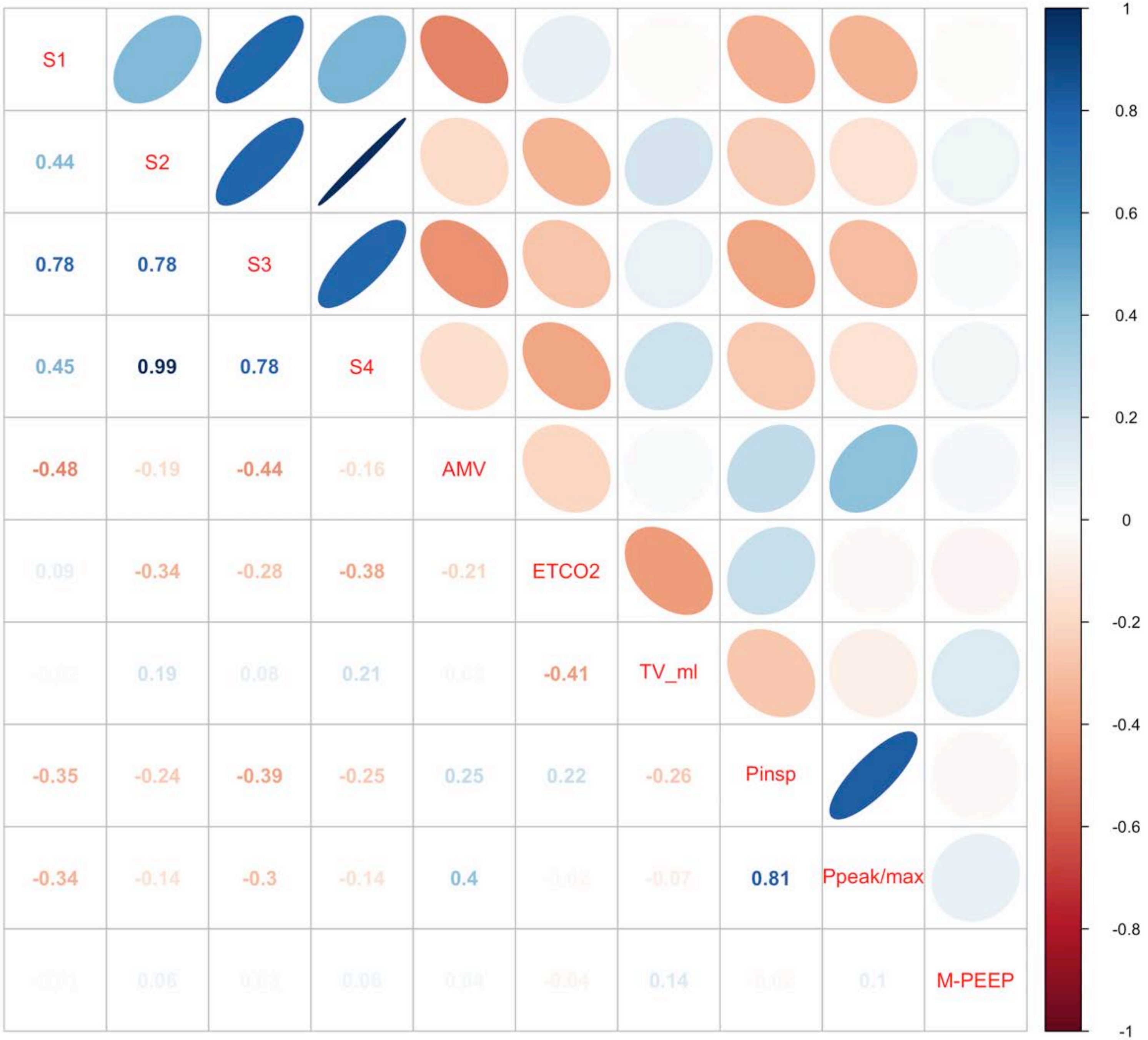

correlation Sensors ~ ventilator 1034

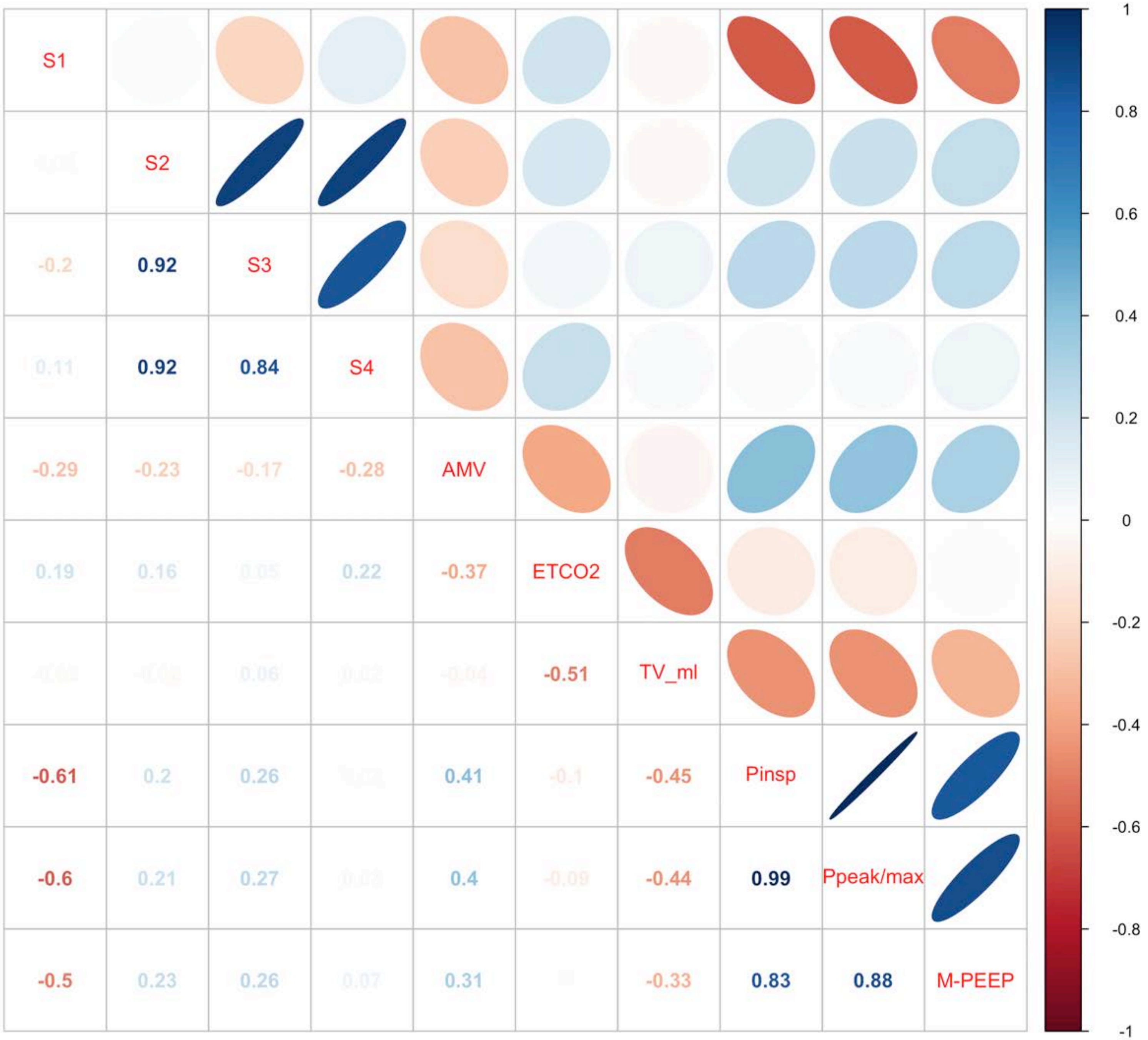

correlation Sensors ~ ventilator 1045

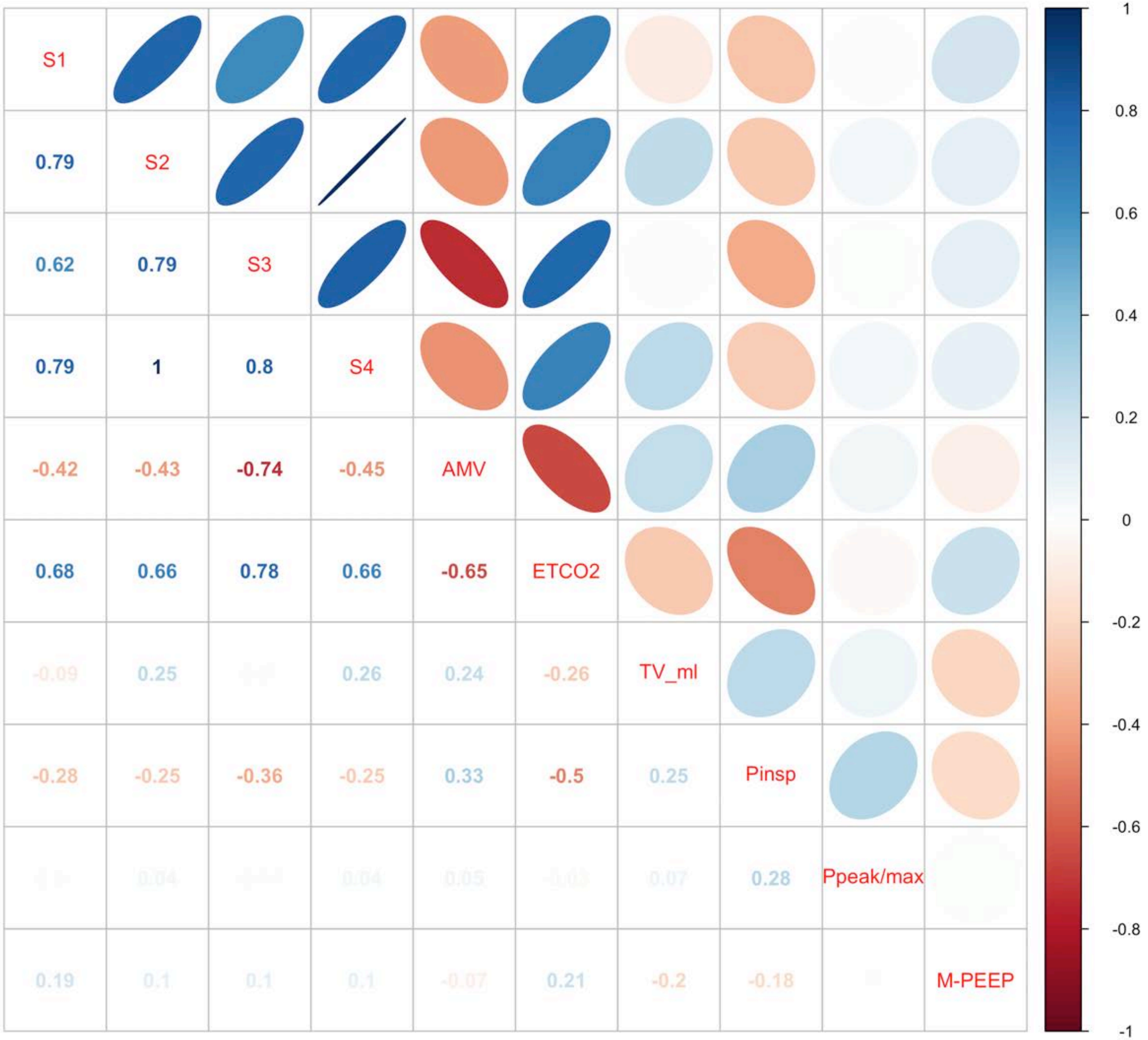

correlation Sensors ~ ventilator 1047

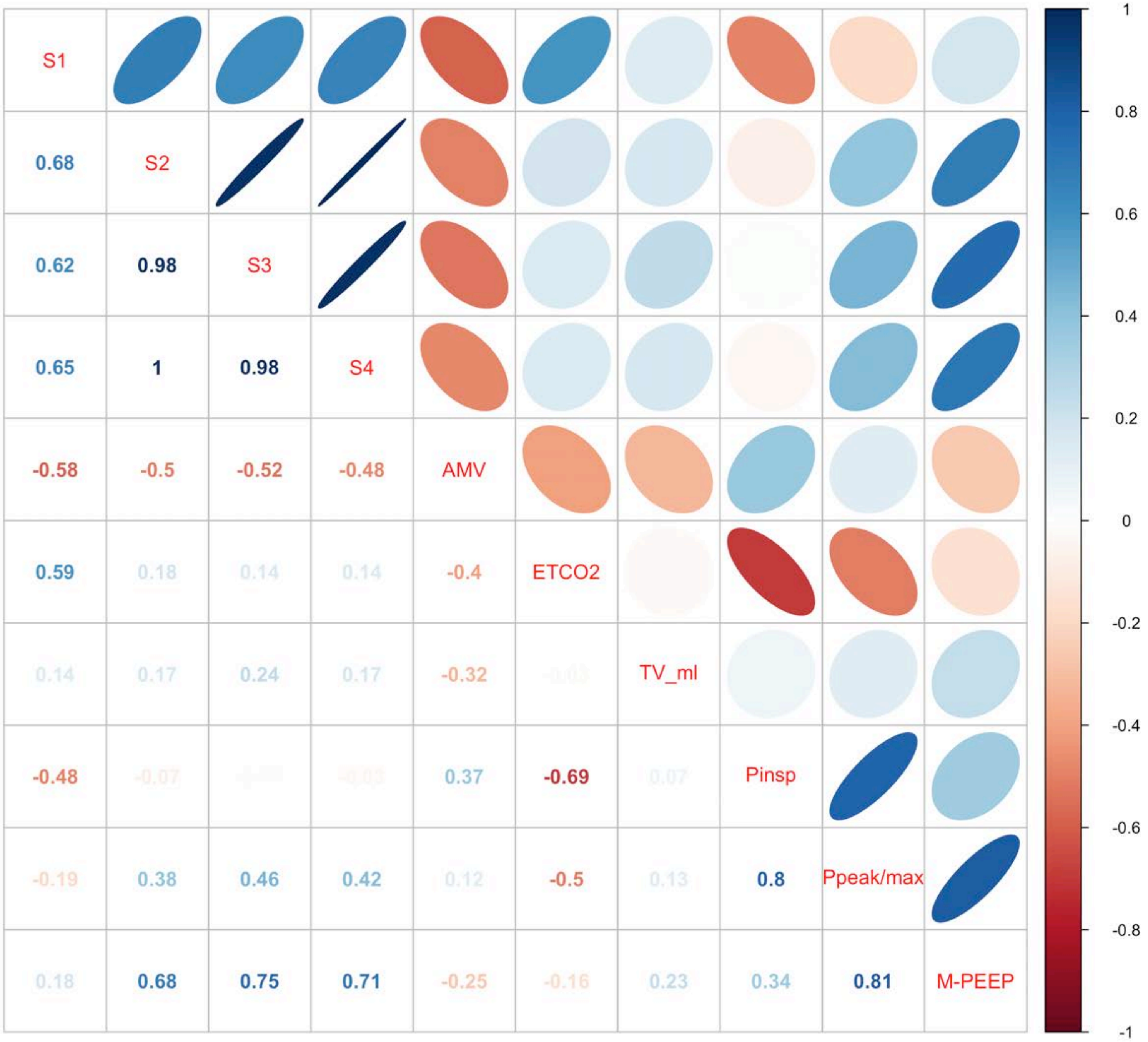

correlation Sensors ~ ventilator 1048

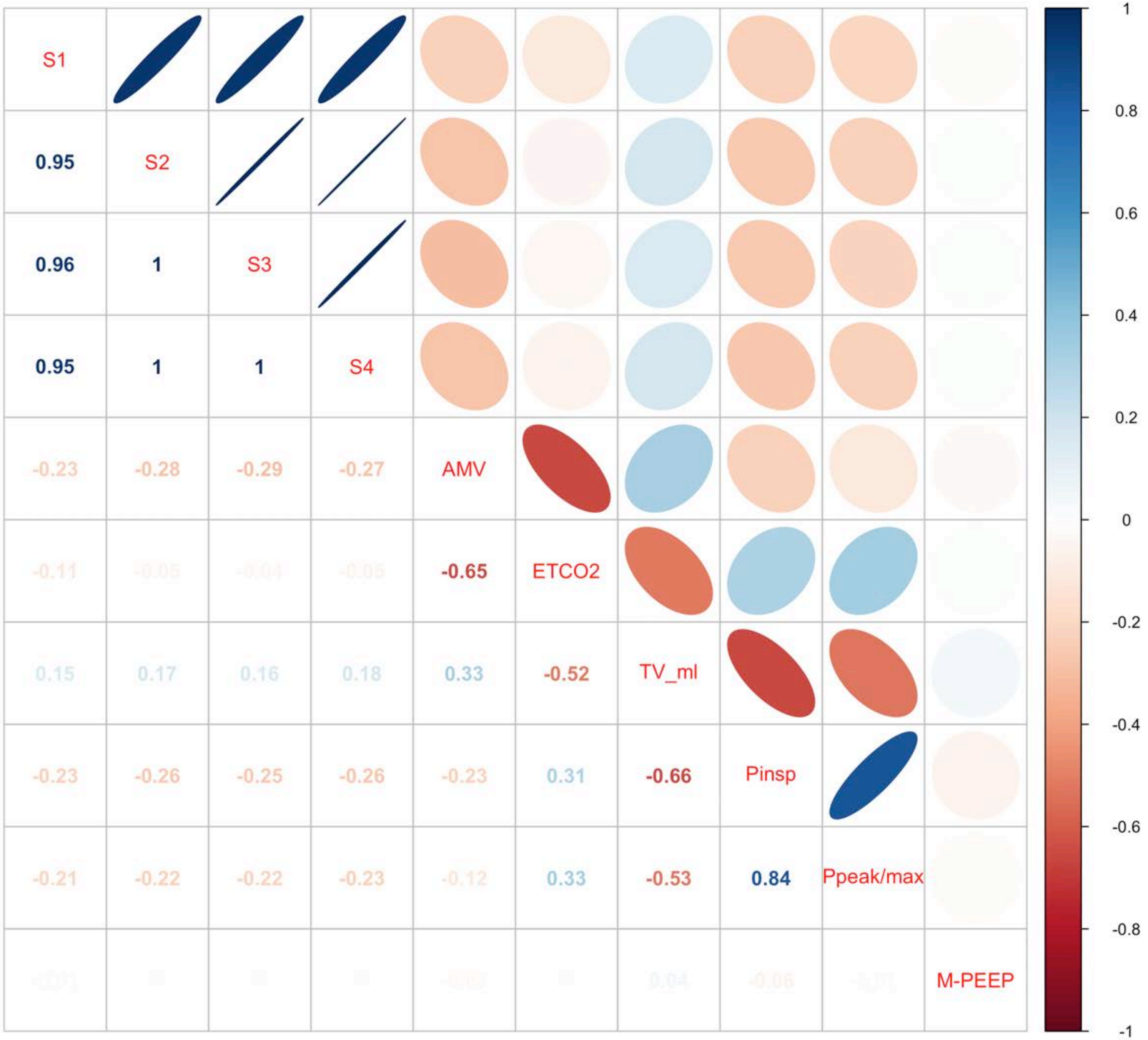

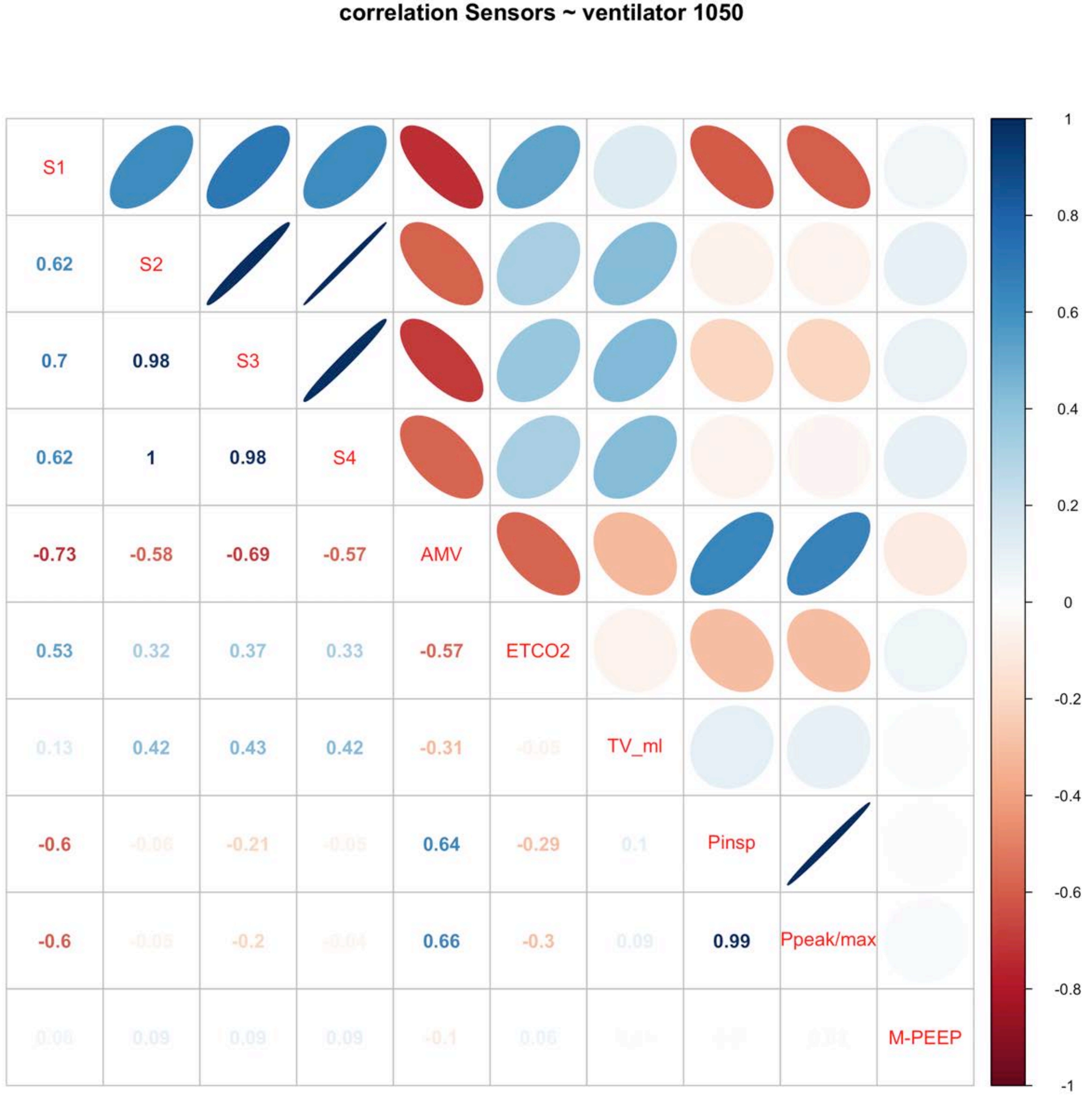
correlation Sensors ~ ventilator 1050
S1
S2
S3
S4
AMV
ETCO2
TV_ml
Pinsp
Ppeak/max
M-PEEP
0.62
0.7
0.98
0.62
1
0.98
-0.73
-0.58
-0.69
-0.57
0.53
0.32
0.37
0.33
-0.57
0.42
0.43
0.42
-0.31
-0.6
-0.21
0.64
-0.29
-0.6
-0.2
0.66
-0.3
0.99
1
0.8
0.6
0.4
0.2
0
-0.2
-0.4
-0.6
-0.8
-1

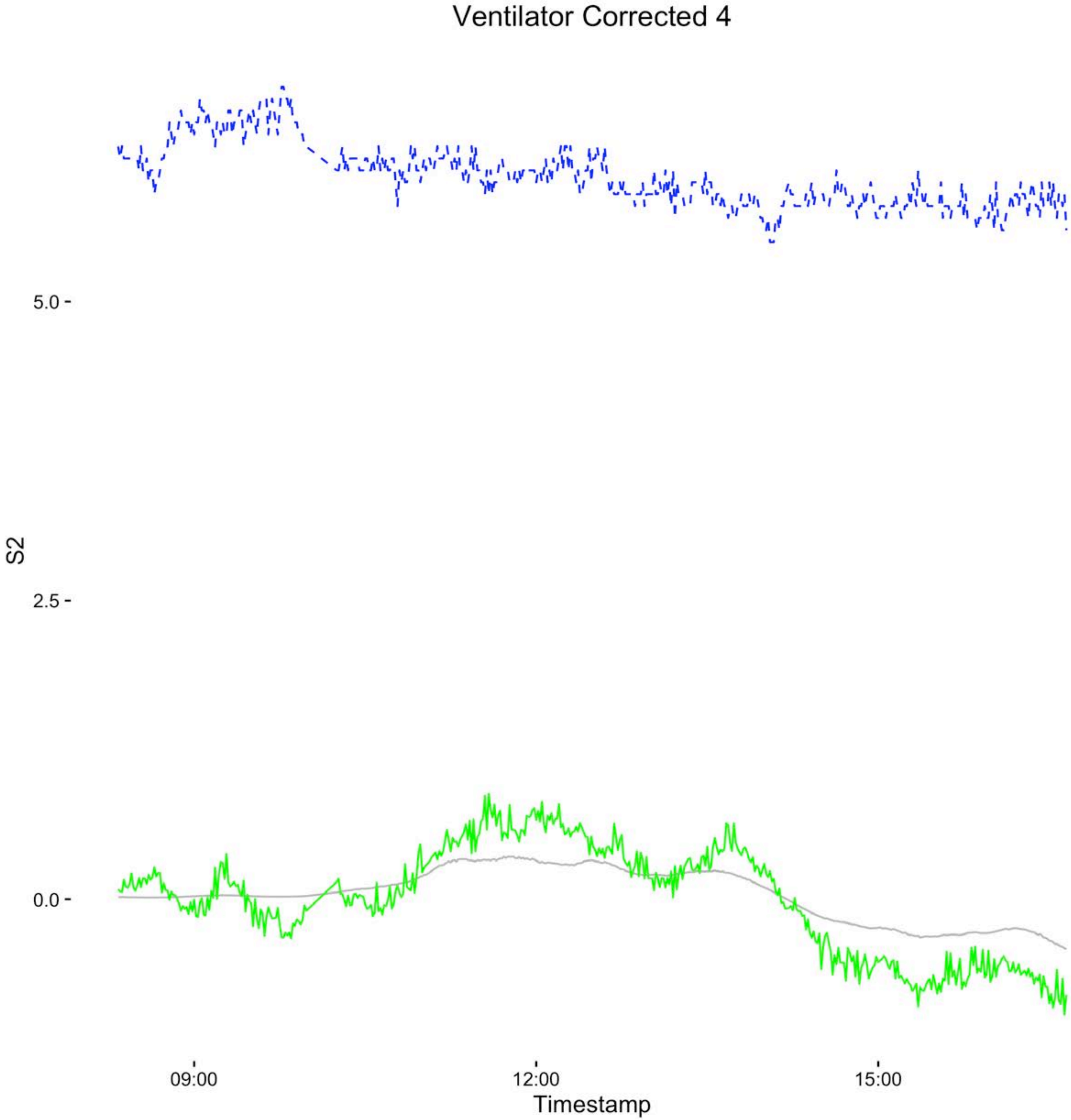
Ventilator Corrected 4
5.0
2.5
0.0
S2
09:00
12:00
15:00
Timestamp

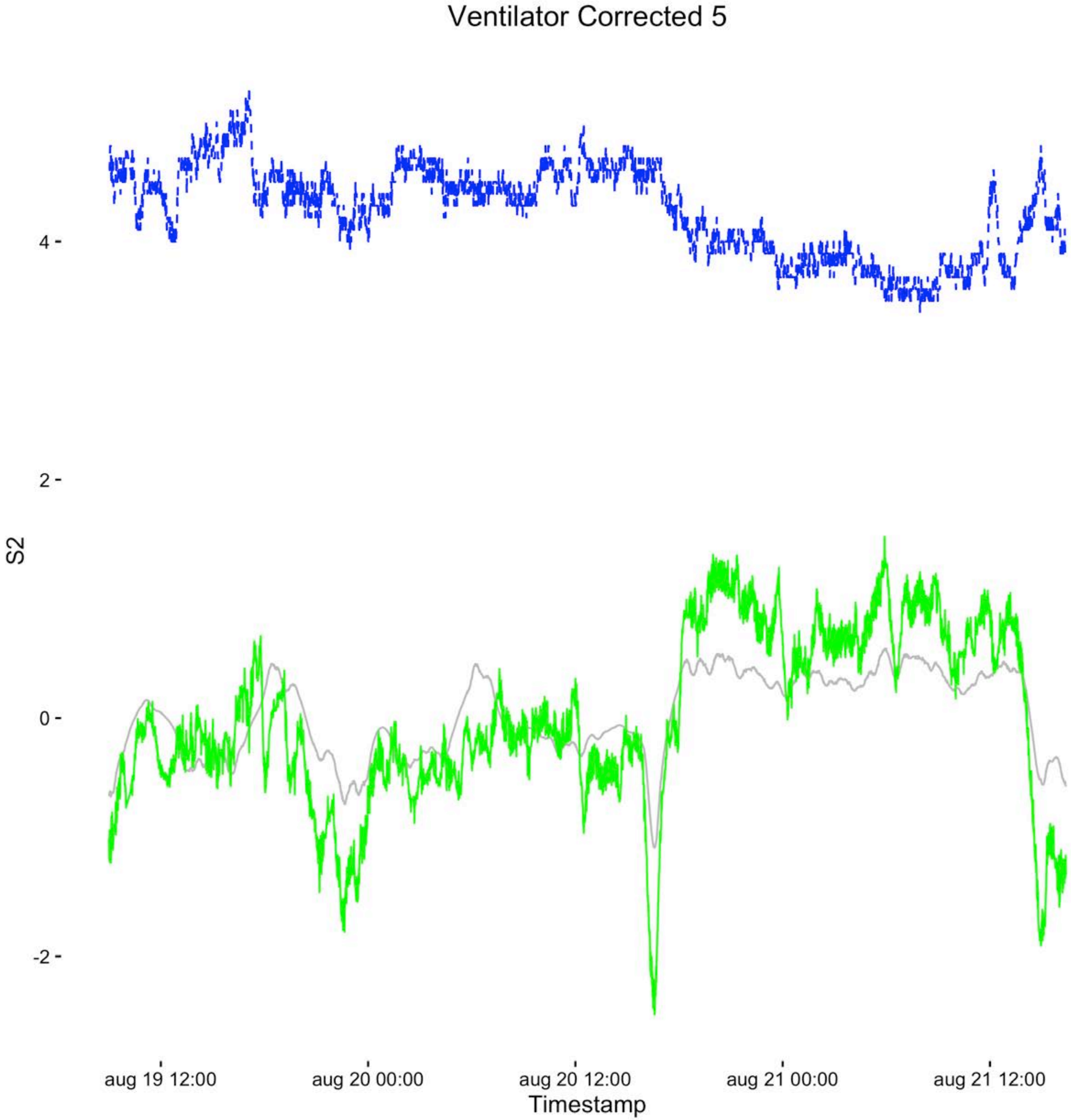
Ventilator Corrected 5
4
2
0
-2
S2
aug 19 12:00
aug 20 00:00
aug 20 12:00
aug 21 00:00
aug 21 12:00
Timestamp

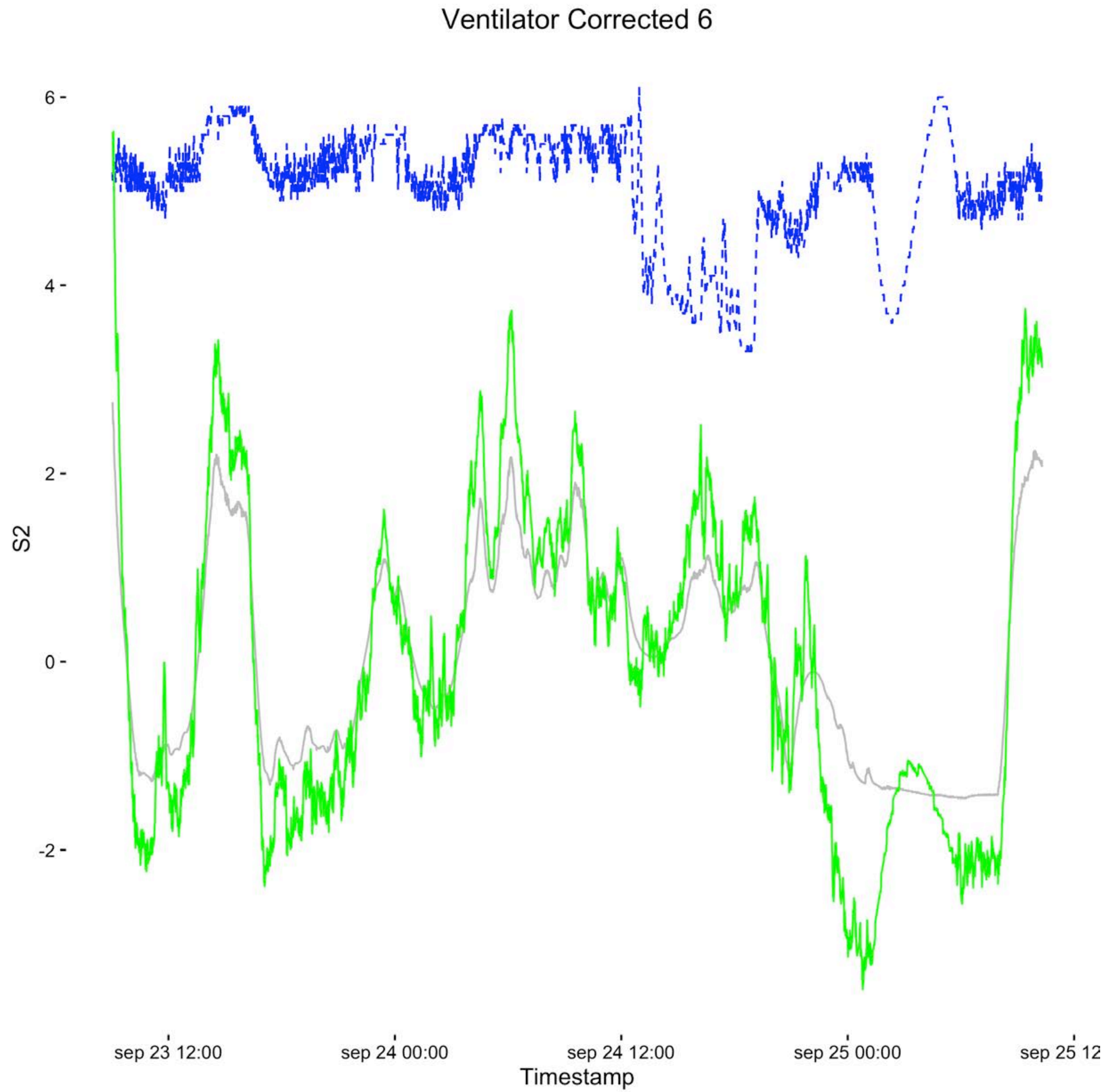

Ventilator Corrected 6
6
4
2
0
-2
S2
sep 23 12:00
sep 24 00:00
sep 24 12:00
sep 25 00:00
sep 25 12
Timestamp

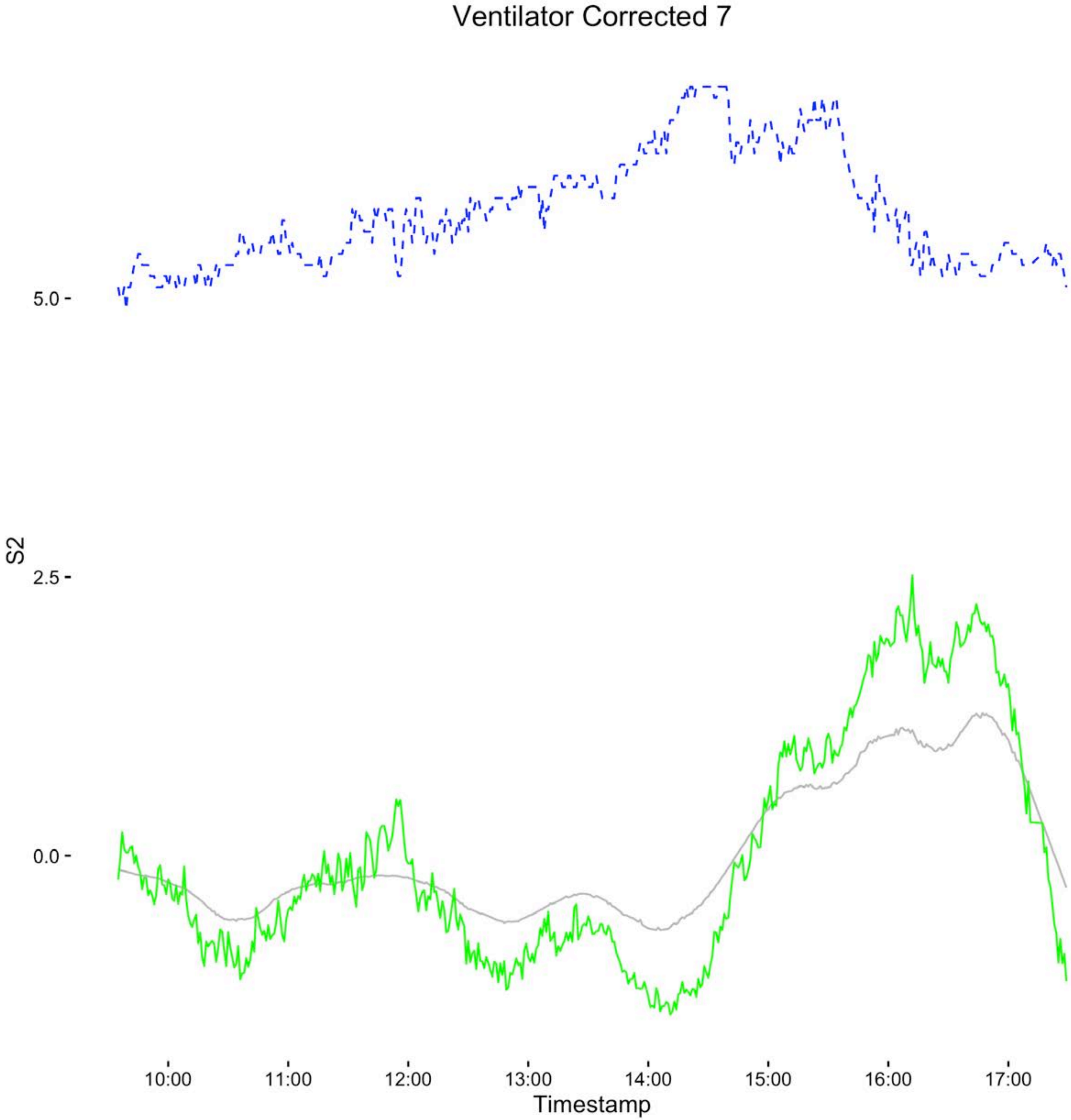

Ventilator Corrected 7
S2
5.0
2.5
0.0
10:00
11:00
12:00
13:00
14:00
15:00
16:00
17:00
Timestamp

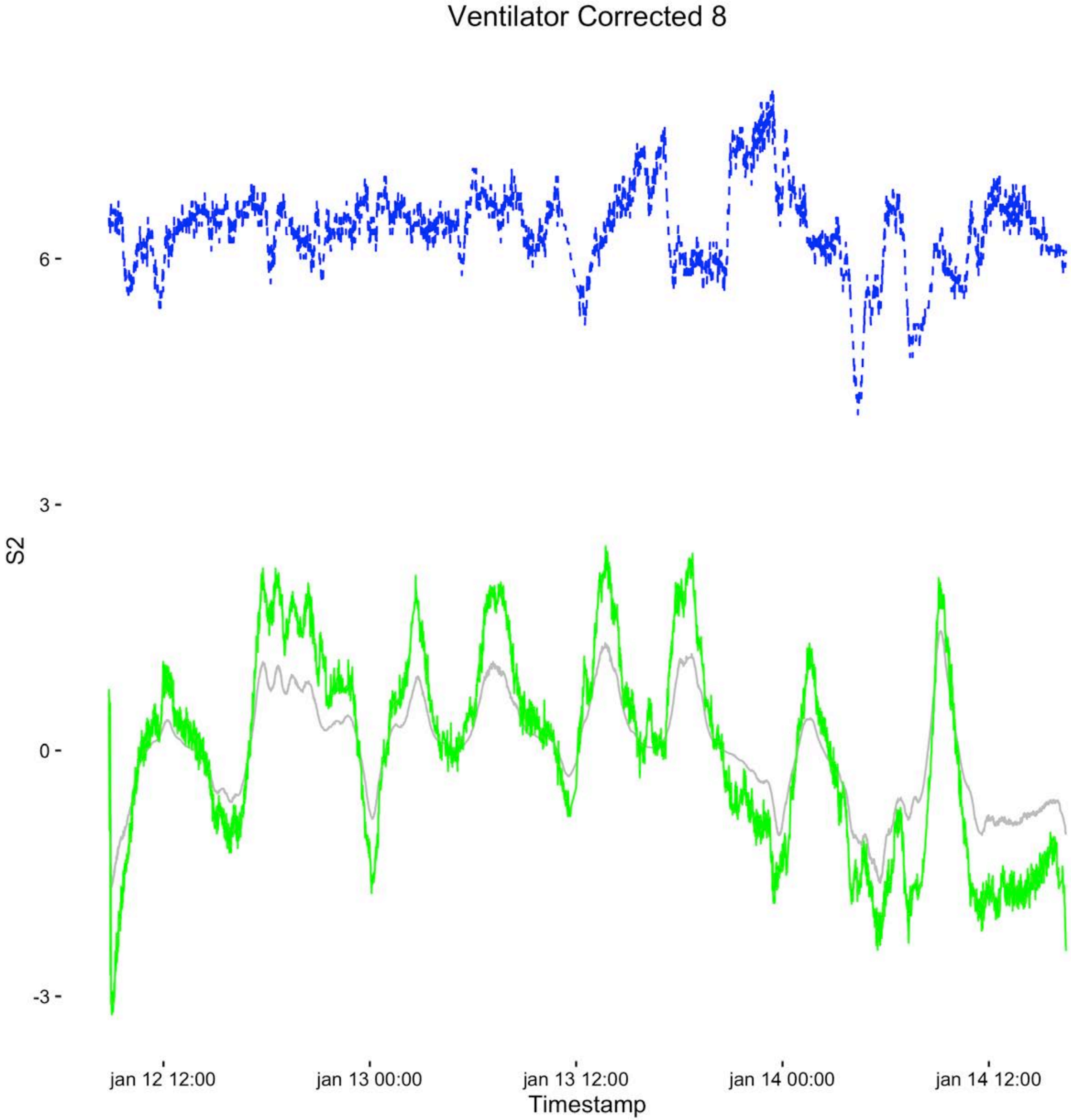

Ventilator Corrected 8
6
3
0
-3
S2
jan 12 12:00
jan 13 00:00
jan 13 12:00
jan 14 00:00
jan 14 12:00
Timestamp

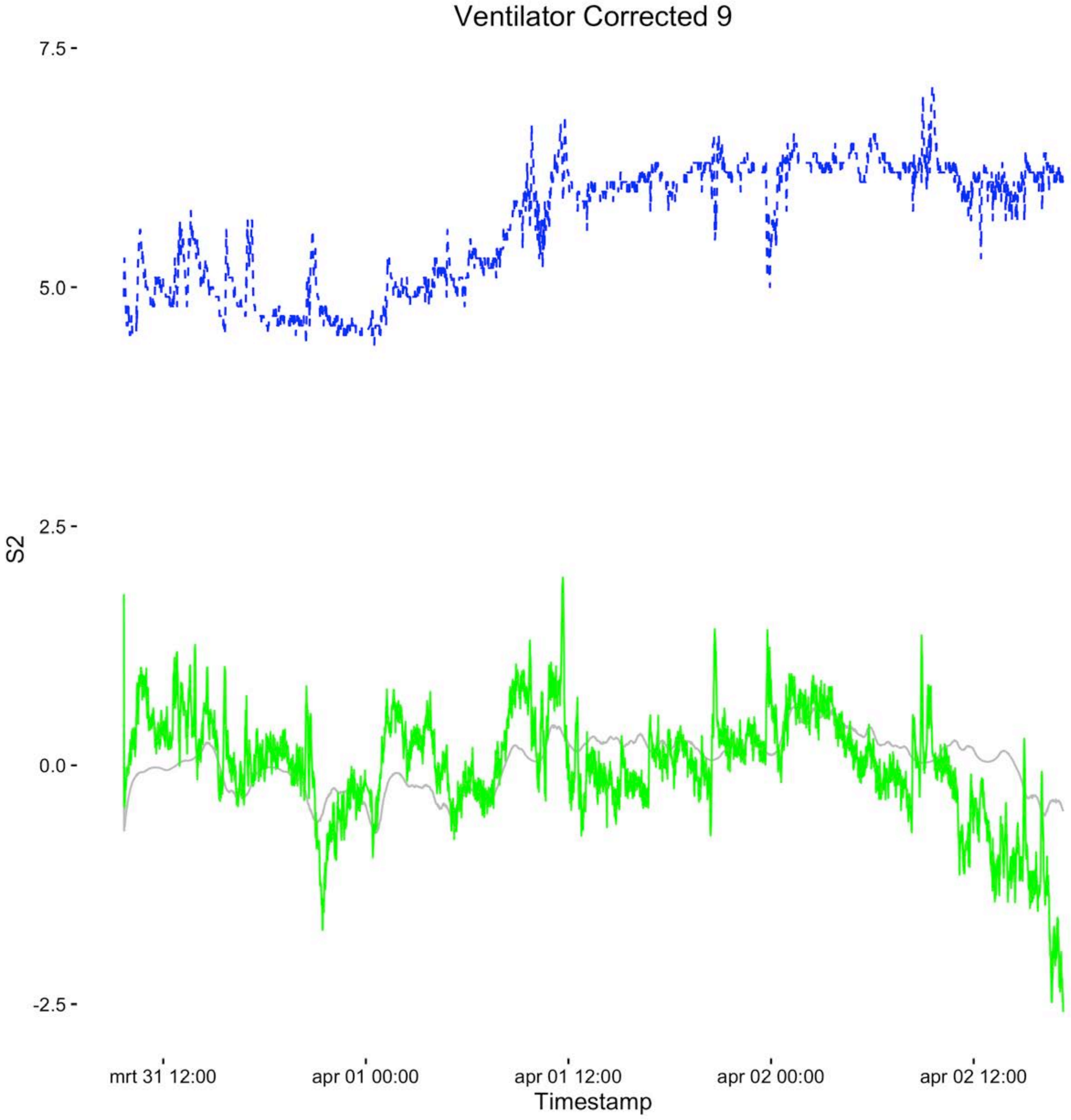

Ventilator Corrected 9
7.5
5.0
2.5
0.0
-2.5
S2
mrt 31 12:00
apr 01 00:00
apr 01 12:00
apr 02 00:00
apr 02 12:00
Timestamp

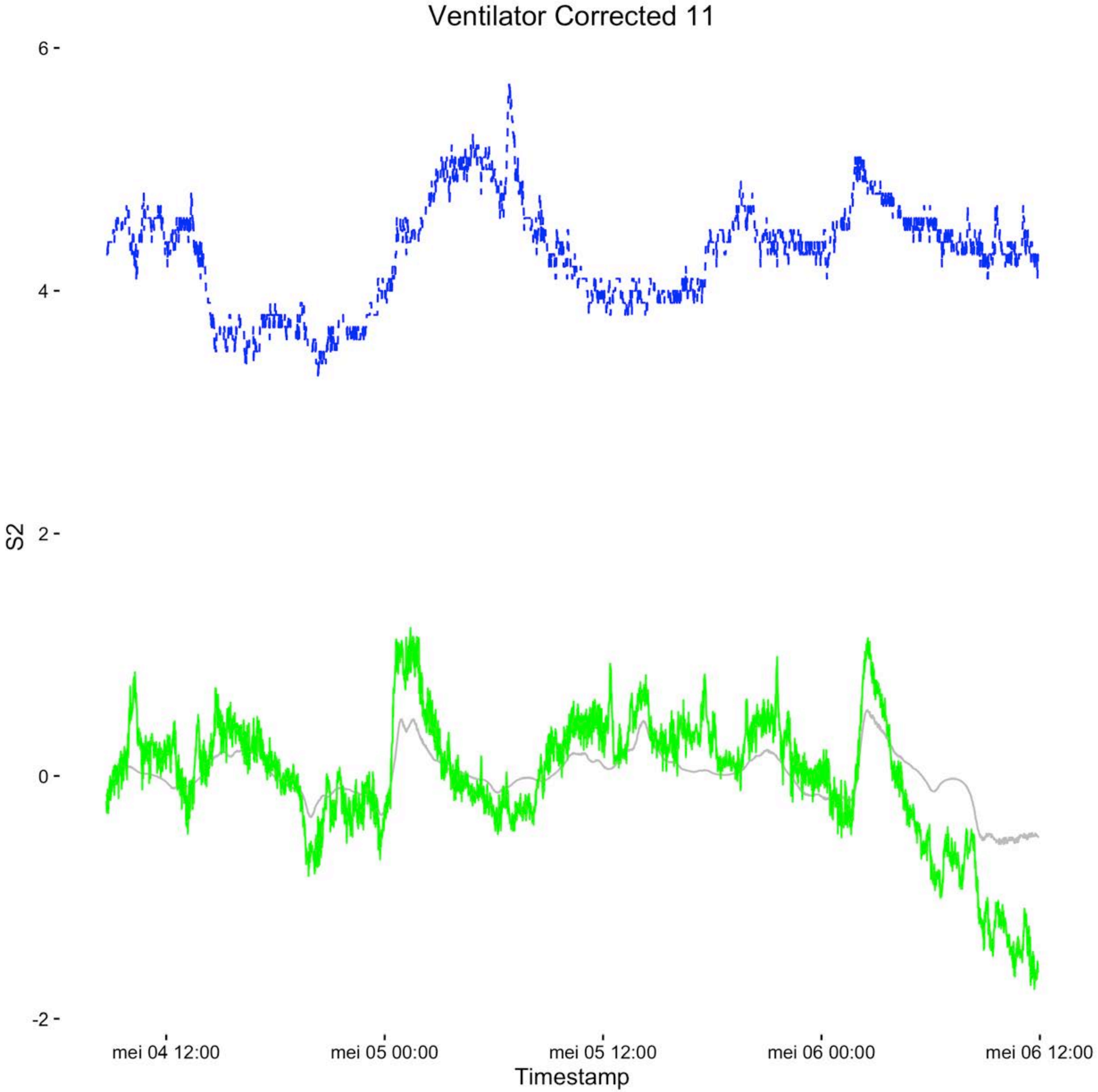

Ventilator Corrected 11
6
4
2
0
-2
S2
mei 04 12:00
mei 05 00:00
mei 05 12:00
mei 06 00:00
mei 06 12:00
Timestamp

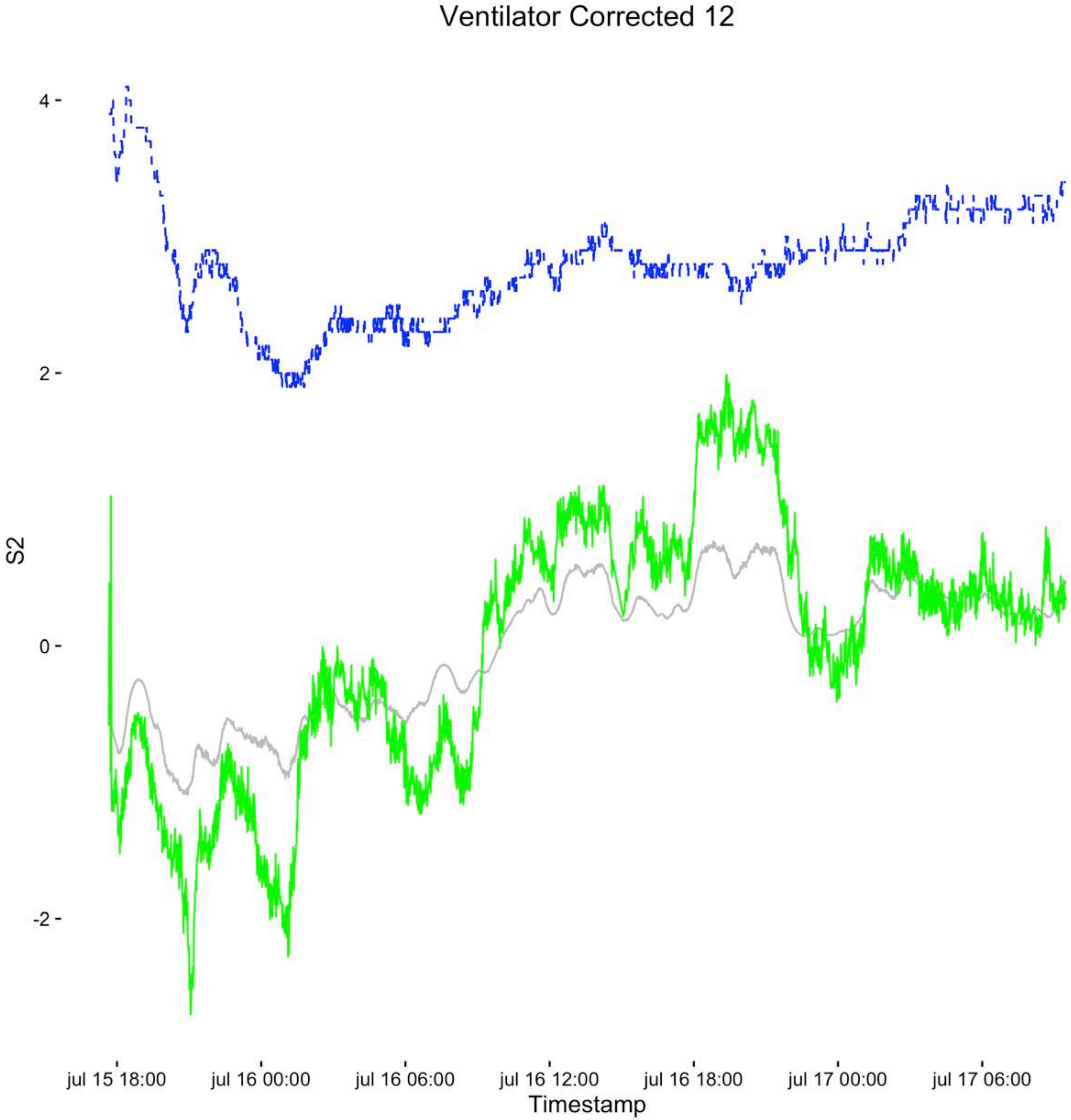

Ventilator Corrected 12
4
2
0
-2
S2
jul 15 18:00
jul 16 00:00
jul 16 06:00
jul 16 12:00
jul 16 18:00
jul 17 00:00
jul 17 06:00
Timestamp

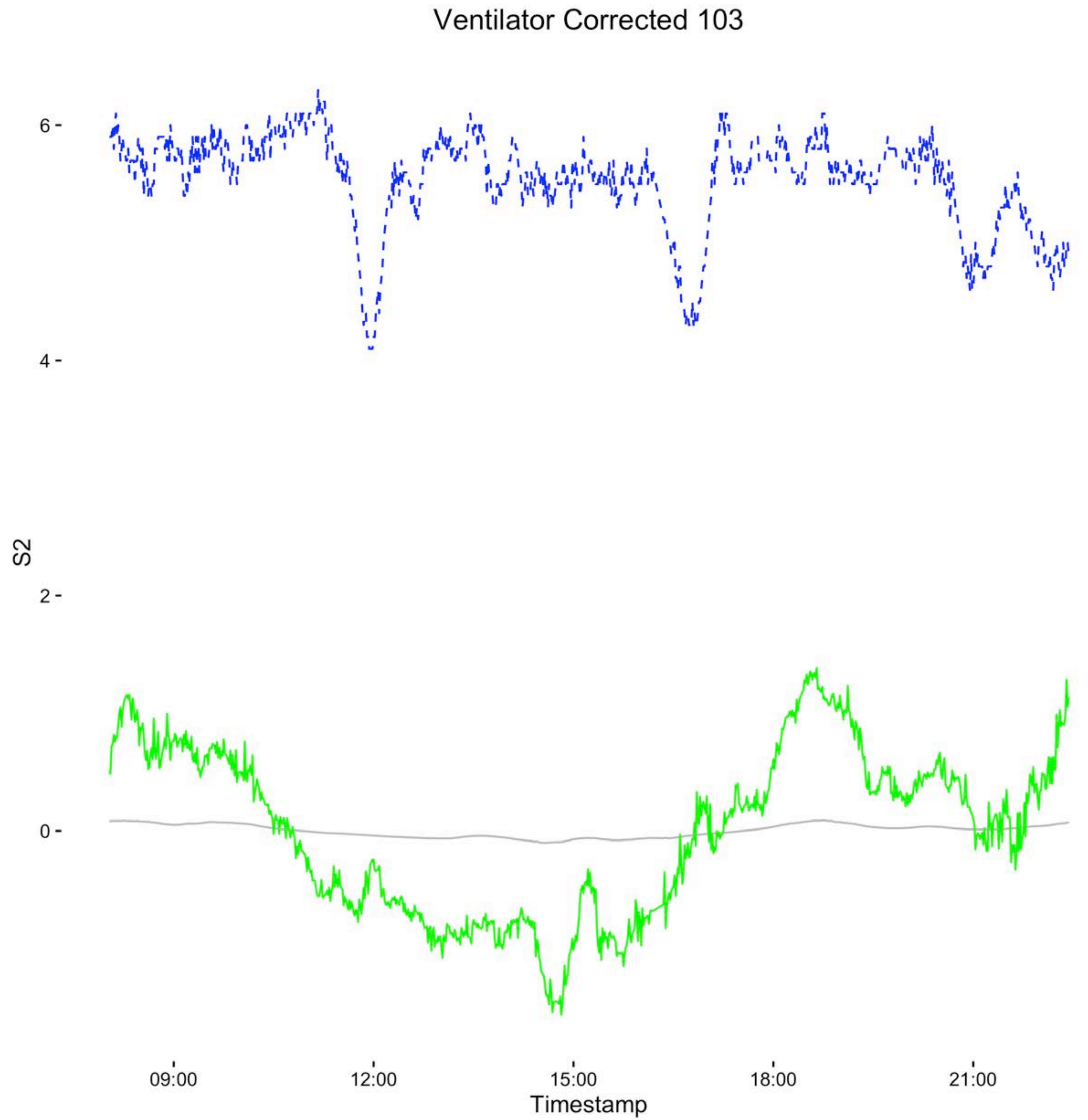

Ventilator Corrected 103
6
4
2
0
S2
09:00
12:00
15:00
18:00
21:00
Timestamp

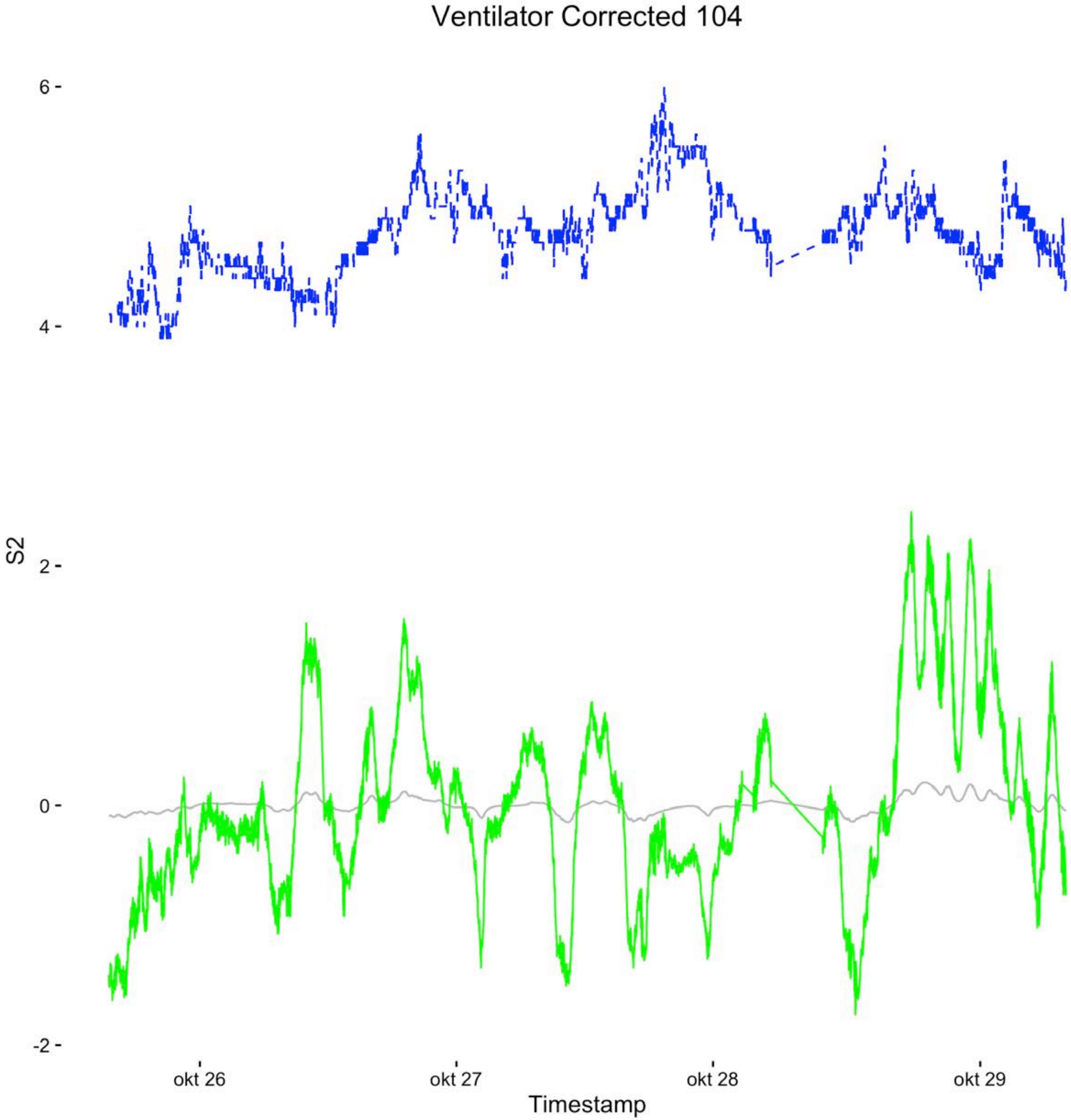

Ventilator Corrected 104
6
4
2
0
-2
S2
okt 26
okt 27
okt 28
okt 29
Timestamp

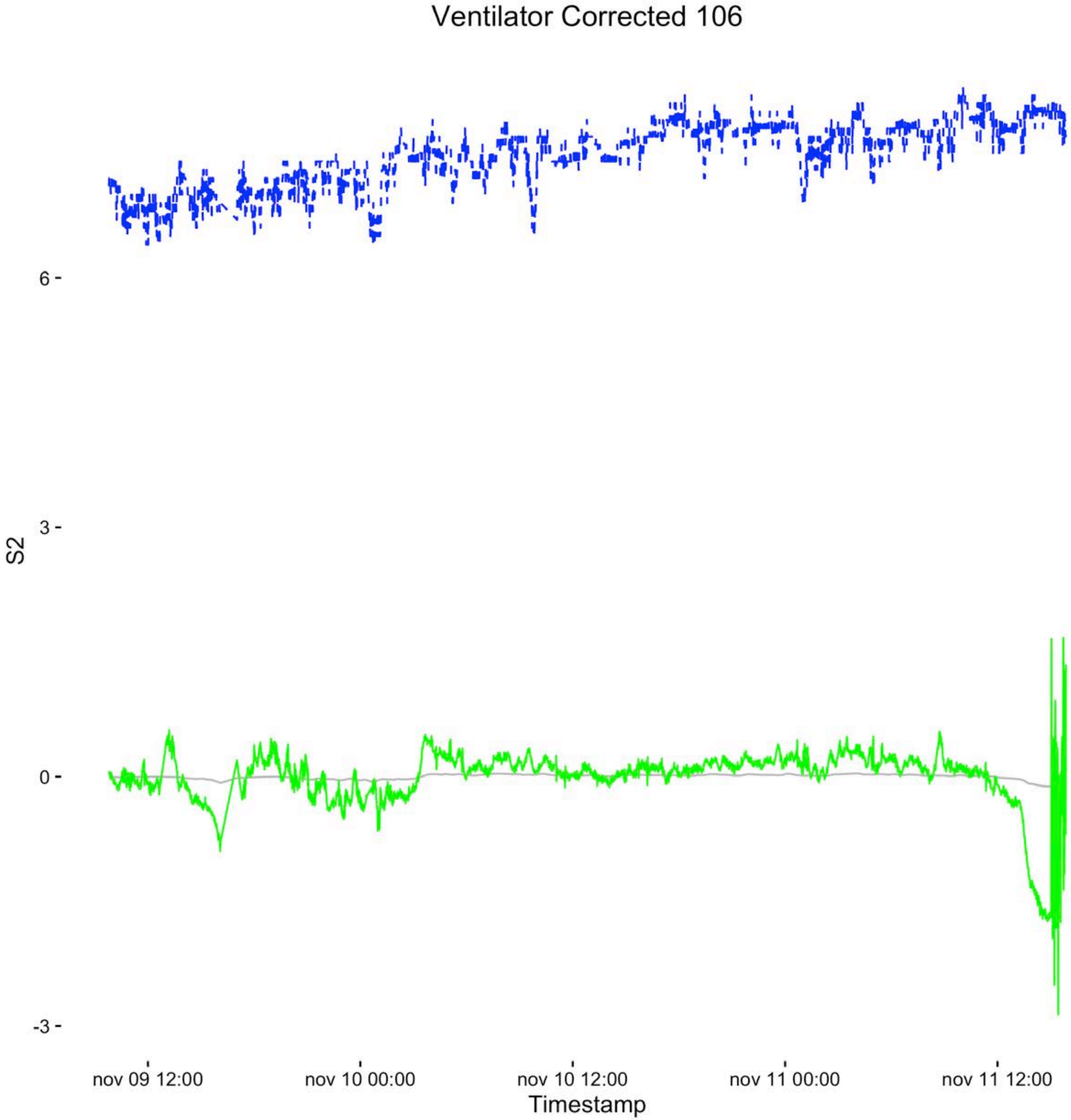
Ventilator Corrected 106
S2
6
3
0
-3
nov 09 12:00
nov 10 00:00
nov 10 12:00
nov 11 00:00
nov 11 12:00
Timestamp

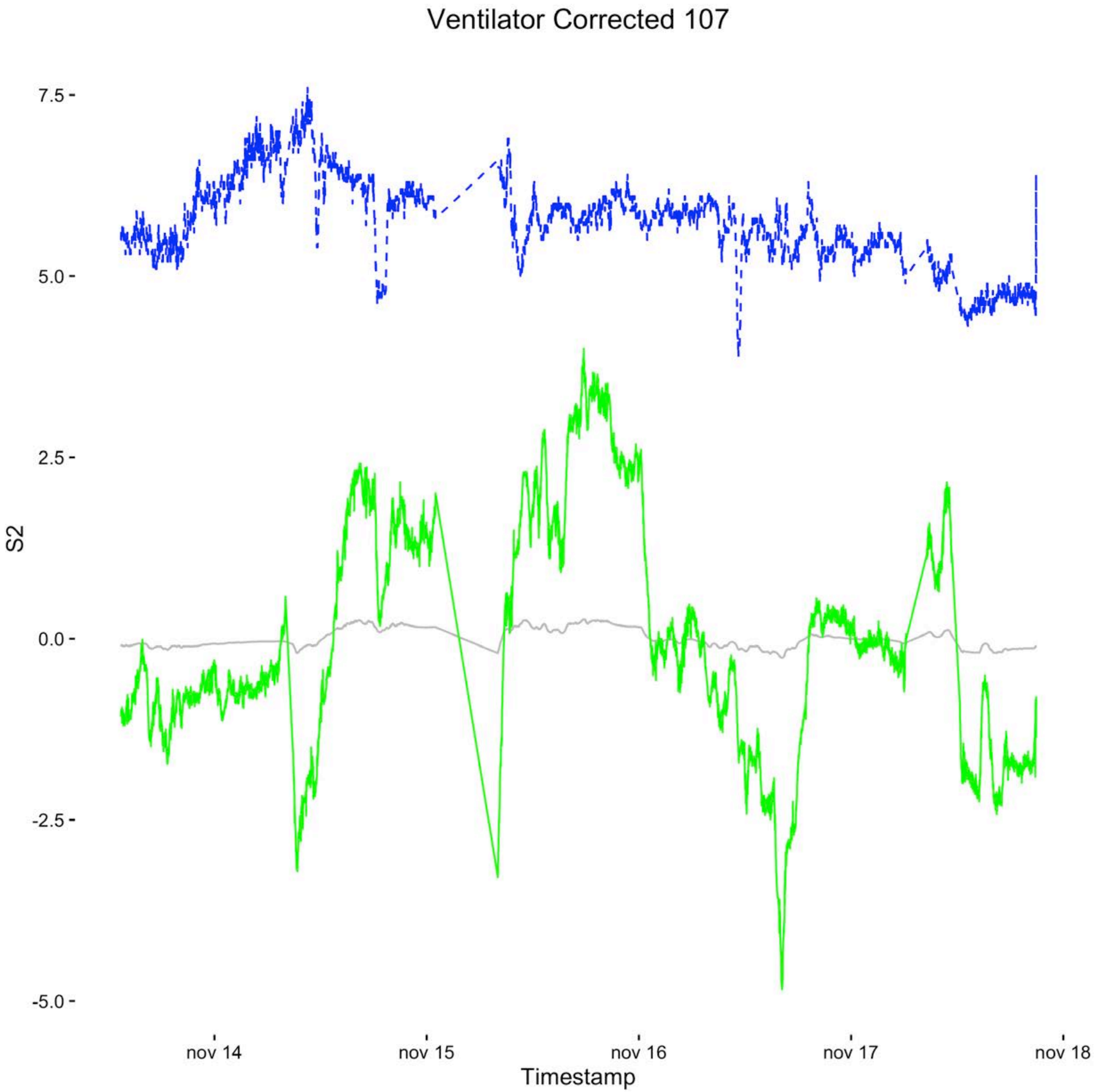

Ventilator Corrected 107
7.5
5.0
2.5
0.0
-2.5
-5.0
S2
nov 14
nov 15
nov 16
nov 17
nov 18
Timestamp

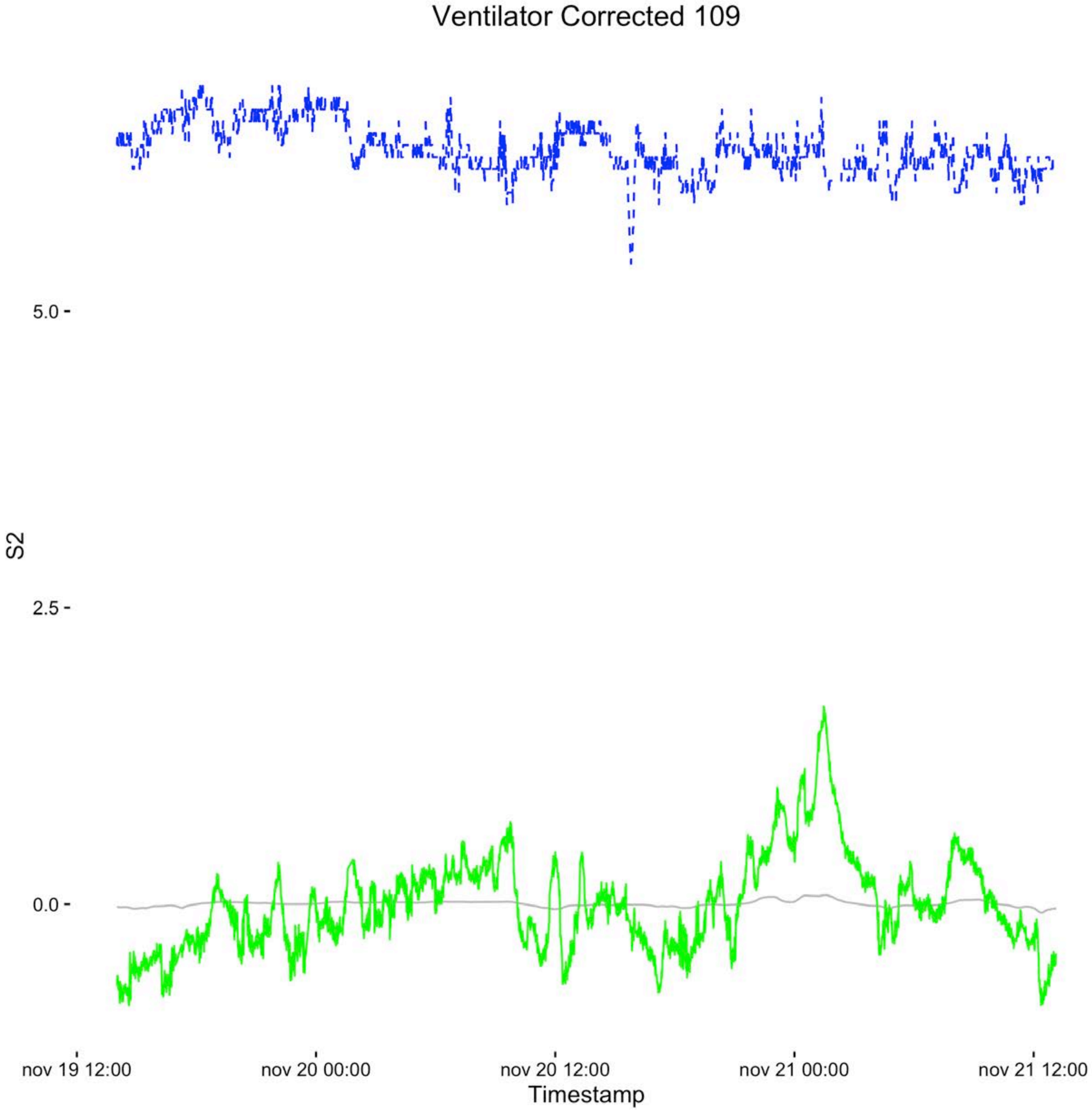
Ventilator Corrected 109
5.0
2.5
0.0
S2
nov 19 12:00
nov 20 00:00
nov 20 12:00
nov 21 00:00
nov 21 12:00
Timestamp

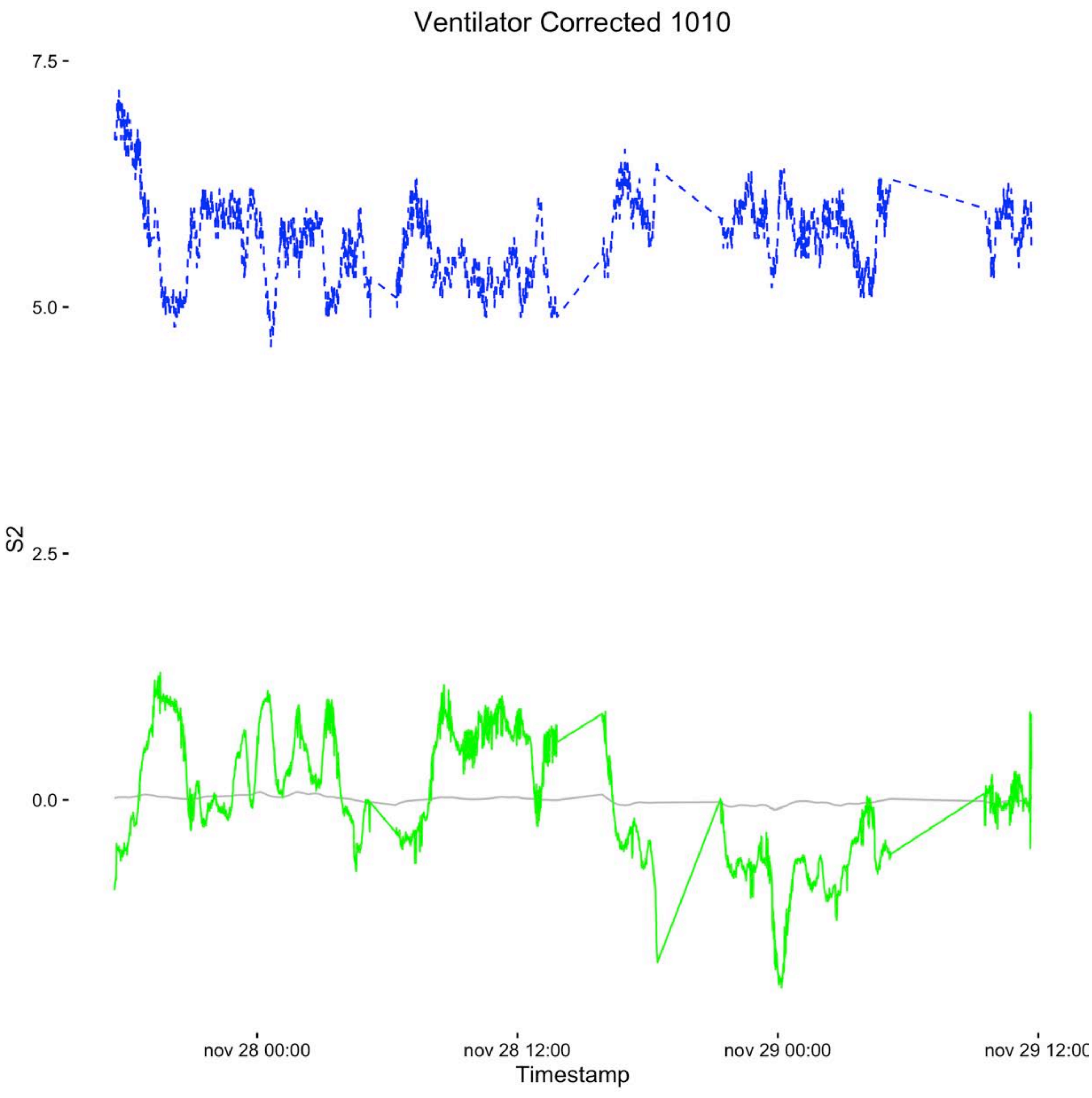
Ventilator Corrected 1010
7.5
5.0
2.5
0.0
S2
nov 28 00:00
nov 28 12:00
nov 29 00:00
nov 29 12:00
Timestamp

Ventilator Corrected 1012

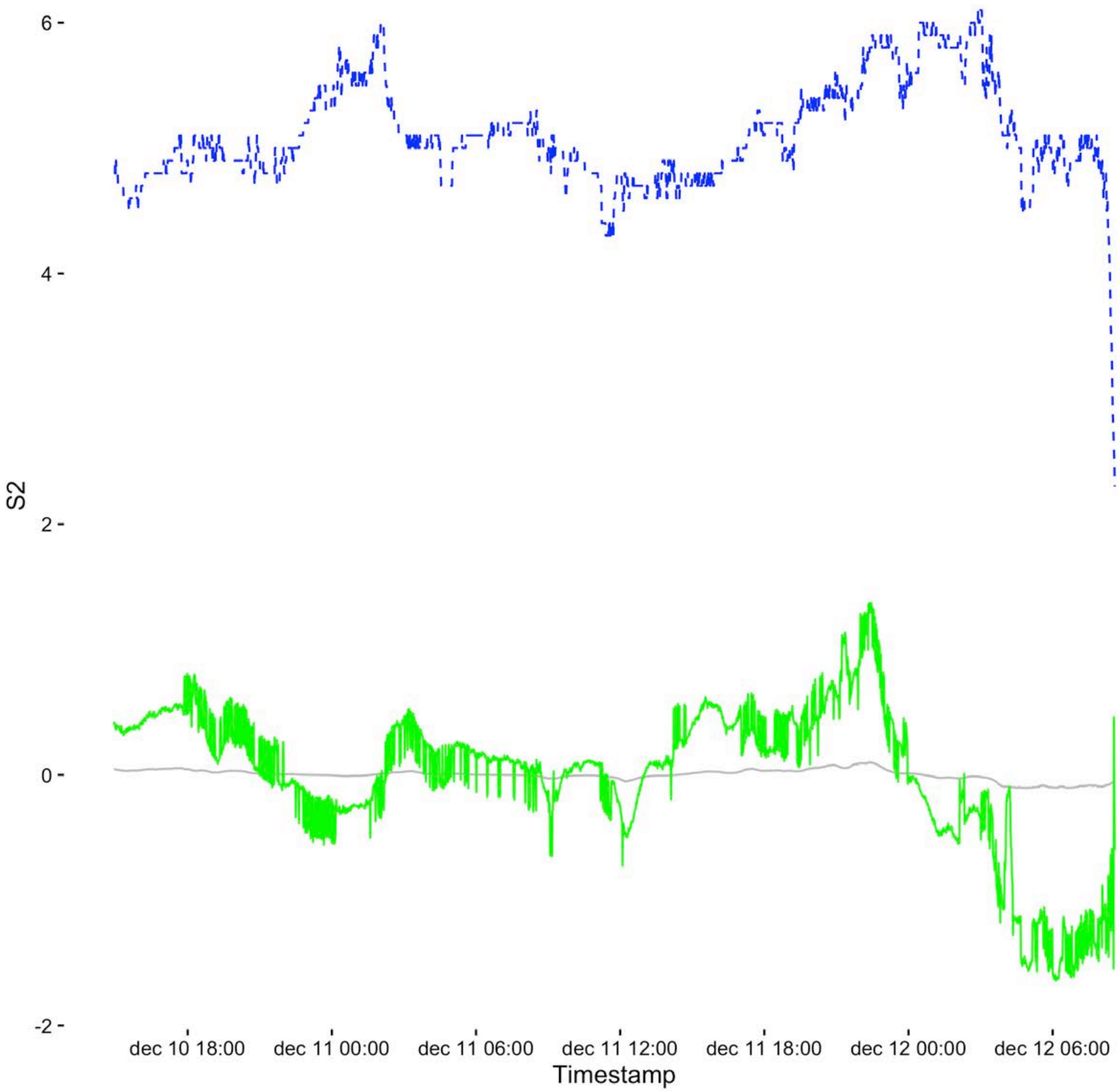

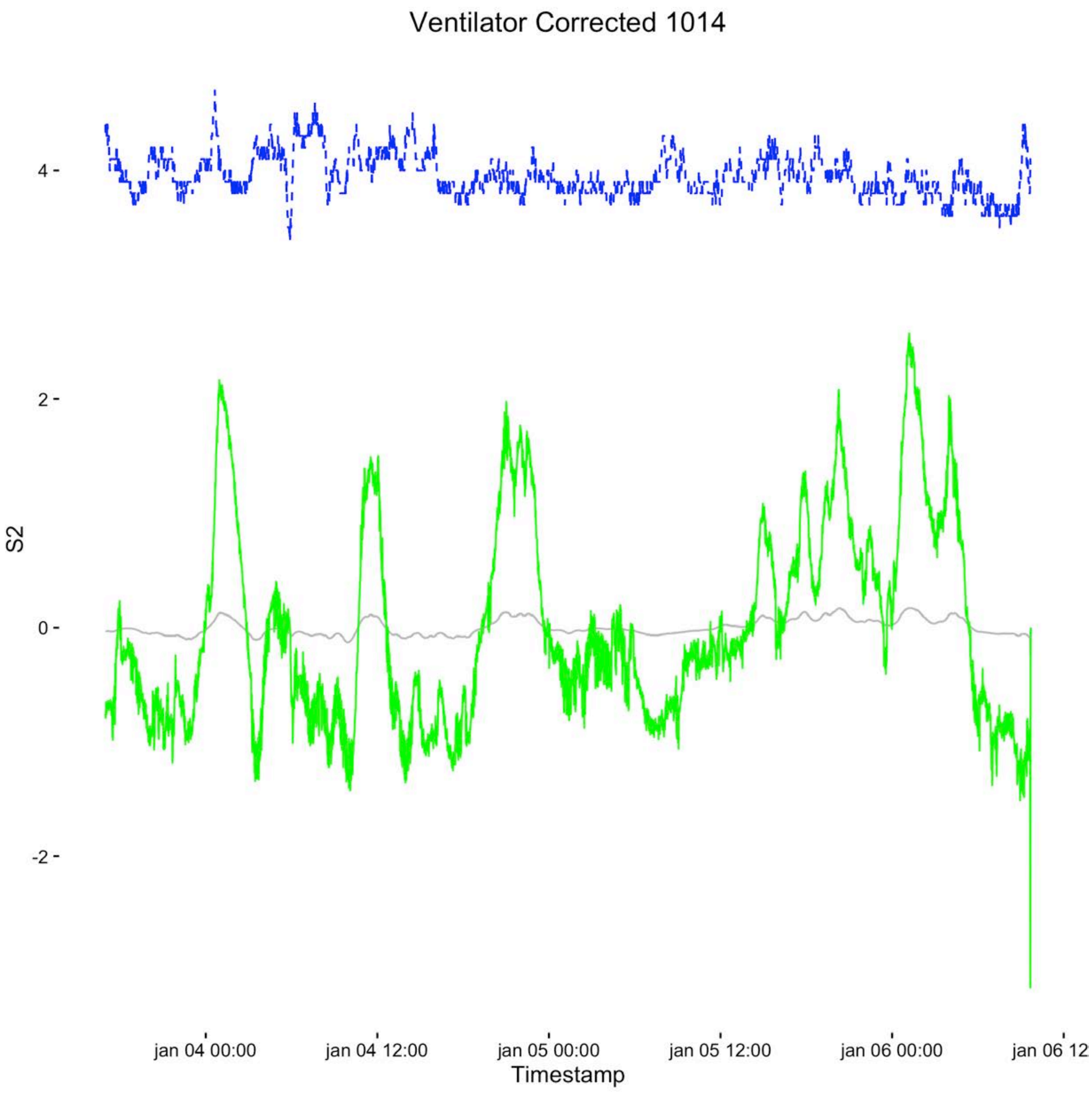
Ventilator Corrected 1014
S2
4
2
0
-2
jan 04 00:00
jan 04 12:00
jan 05 00:00
jan 05 12:00
jan 06 00:00
jan 06 12
Timestamp

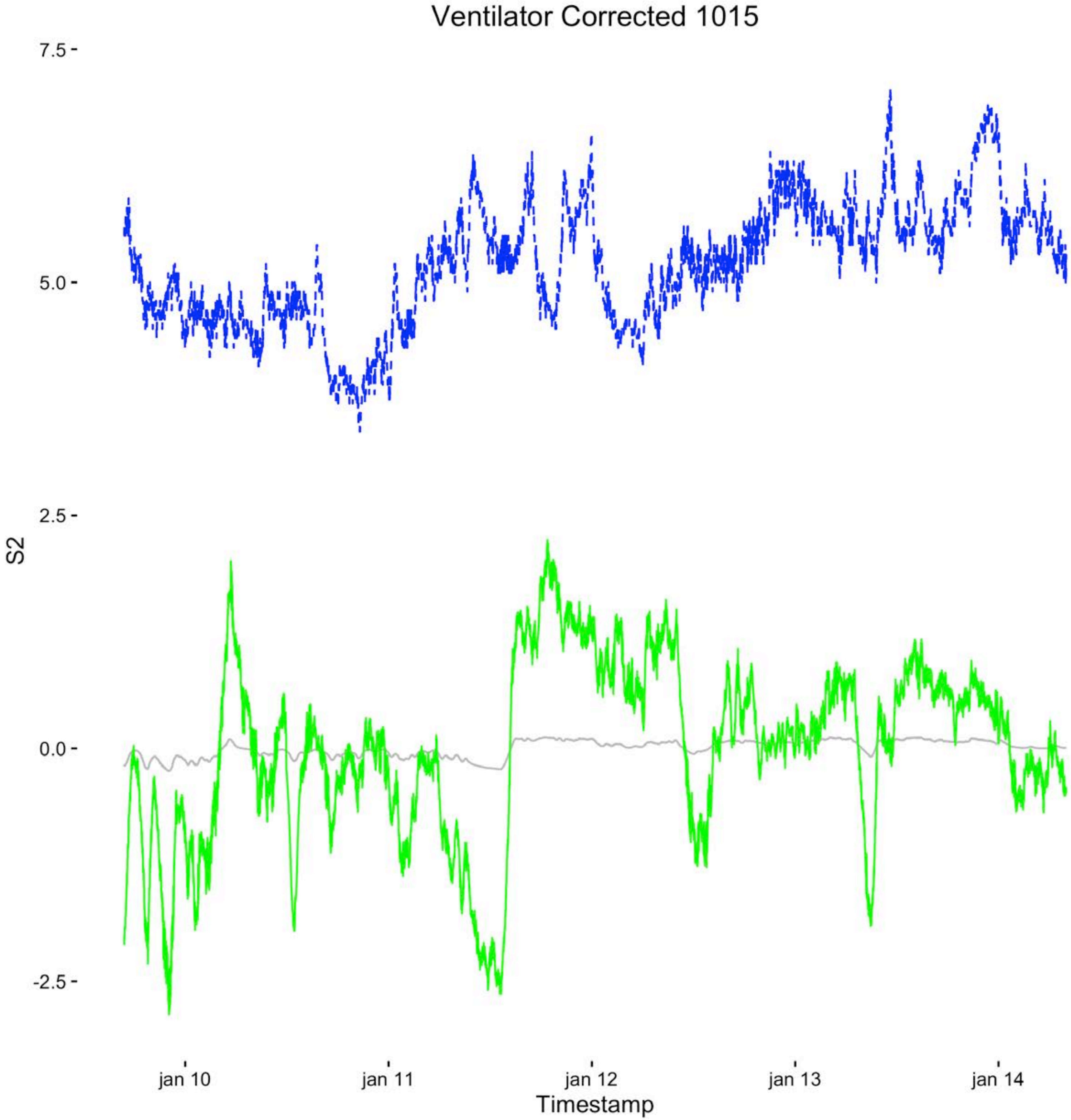
Ventilator Corrected 1015
7.5
5.0
2.5
0.0
-2.5
S2
jan 10
jan 11
jan 12
jan 13
jan 14
Timestamp

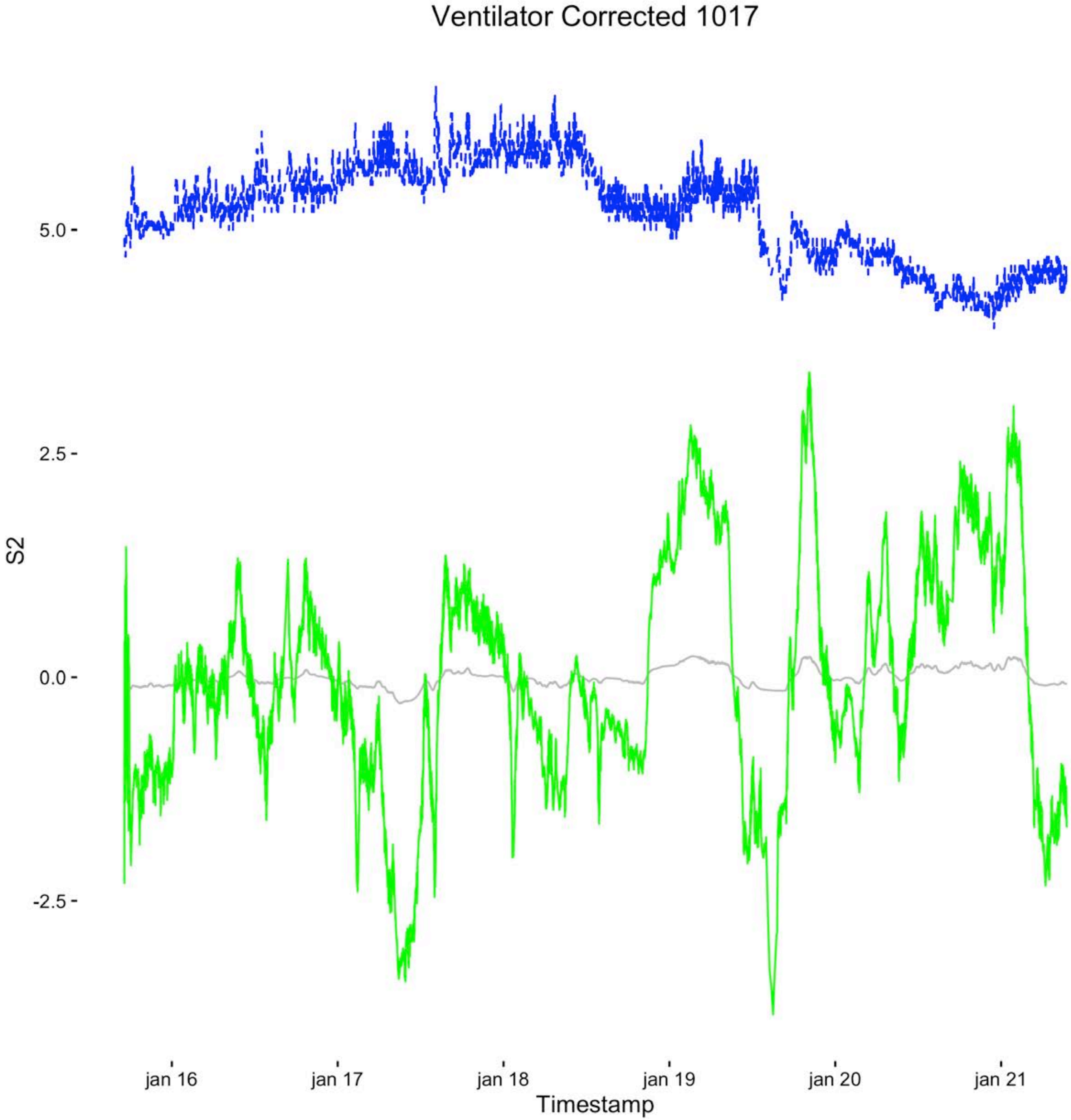

Ventilator Corrected 1017
5.0
2.5
0.0
-2.5
S2
jan 16
jan 17
jan 18
jan 19
jan 20
jan 21
Timestamp

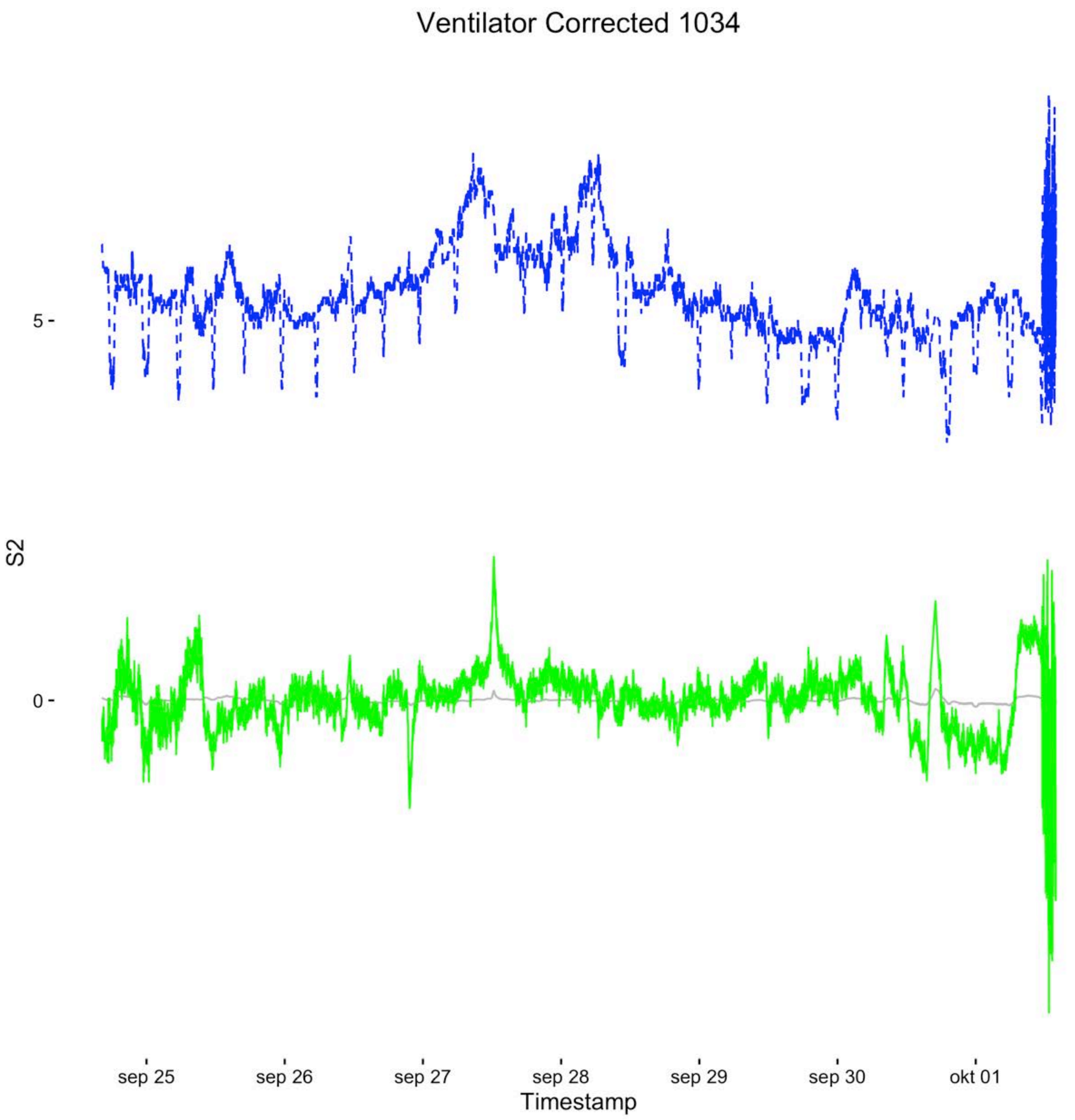

Ventilator Corrected 1034
5
0
S2
sep 25
sep 26
sep 27
sep 28
sep 29
sep 30
okt 01
Timestamp

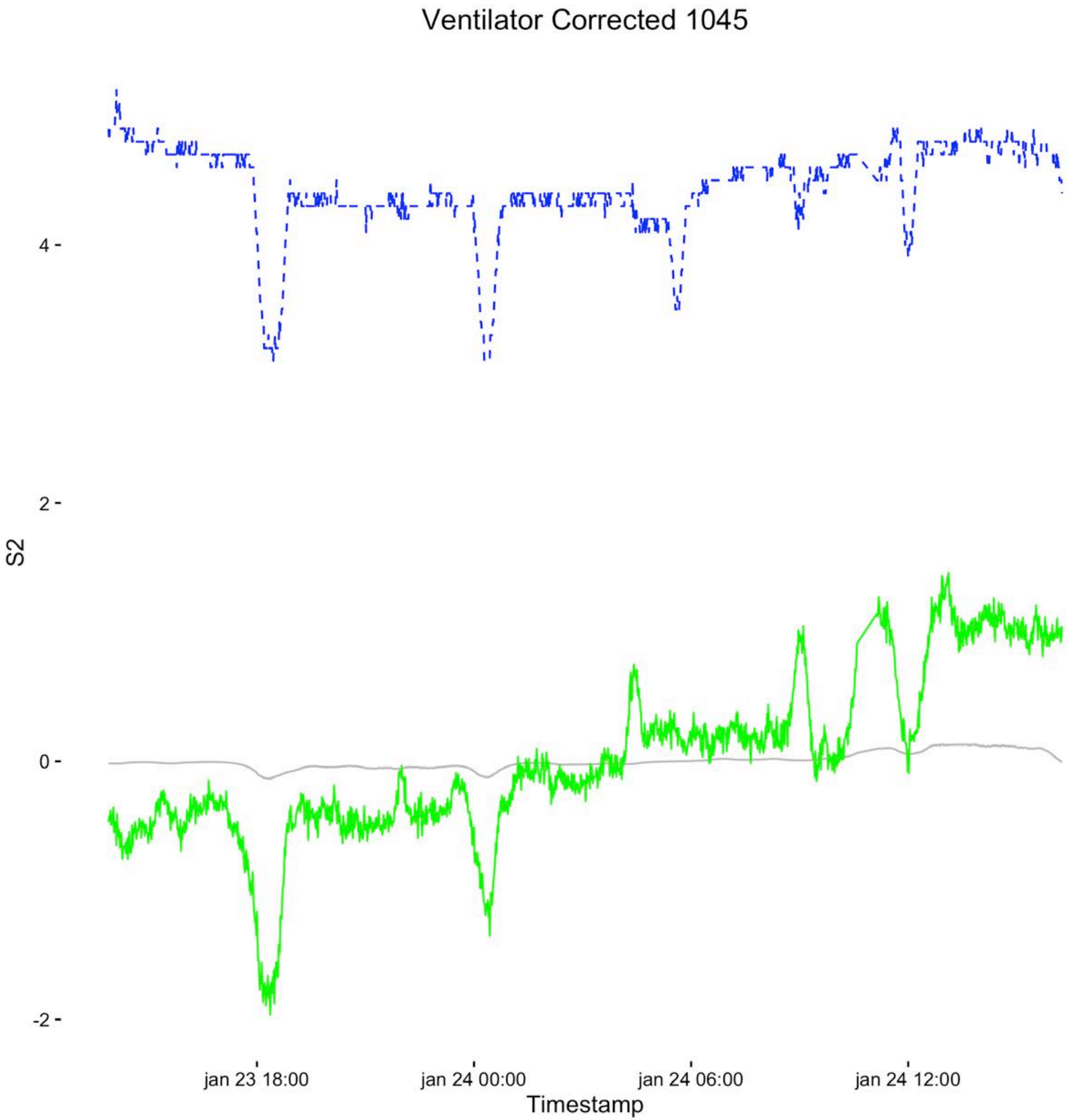

Ventilator Corrected 1045
4
2
0
-2
S2
jan 23 18:00
jan 24 00:00
jan 24 06:00
jan 24 12:00
Timestamp

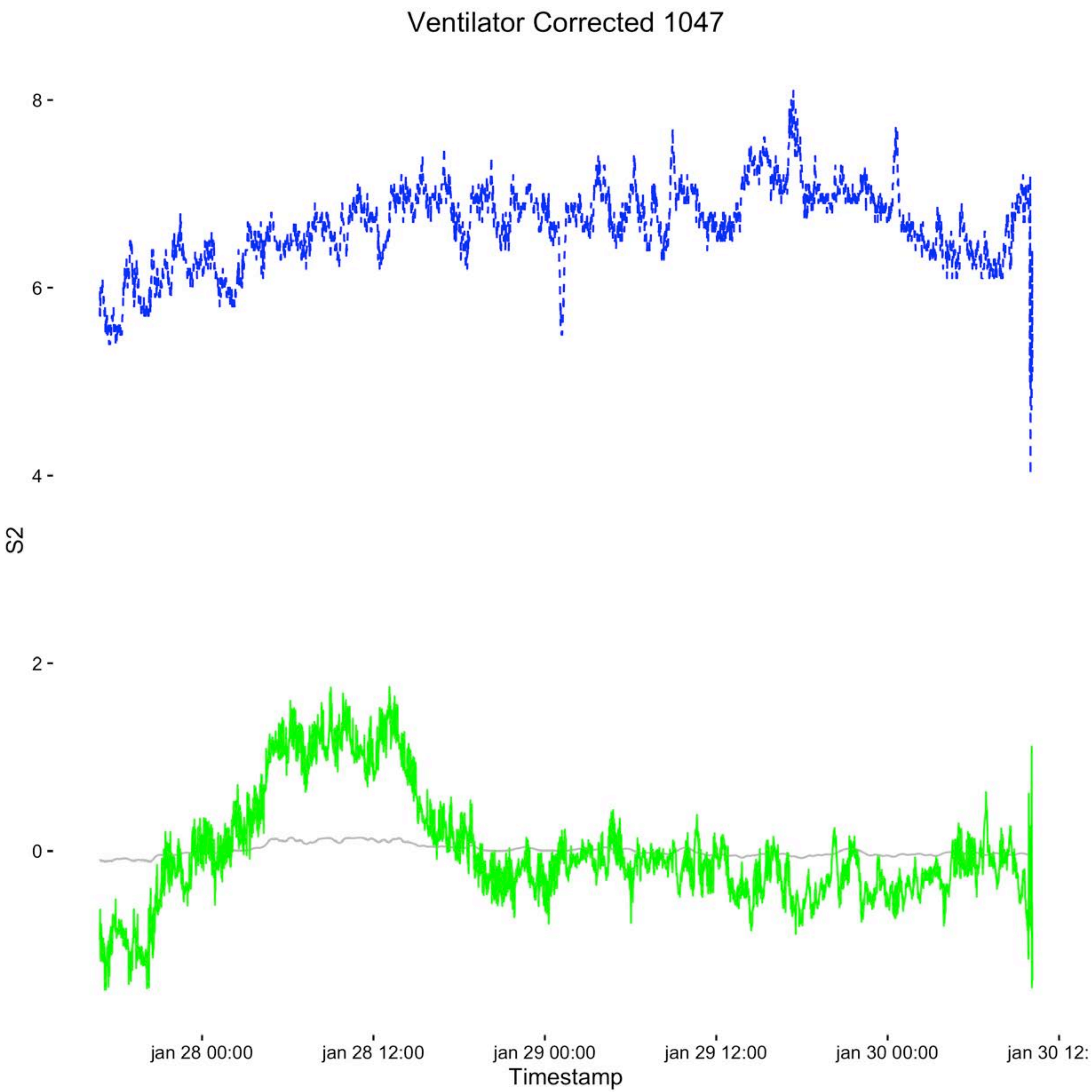
Ventilator Corrected 1047
8
6
4
2
0
S2
jan 28 00:00
jan 28 12:00
jan 29 00:00
jan 29 12:00
jan 30 00:00
jan 30 12:
Timestamp

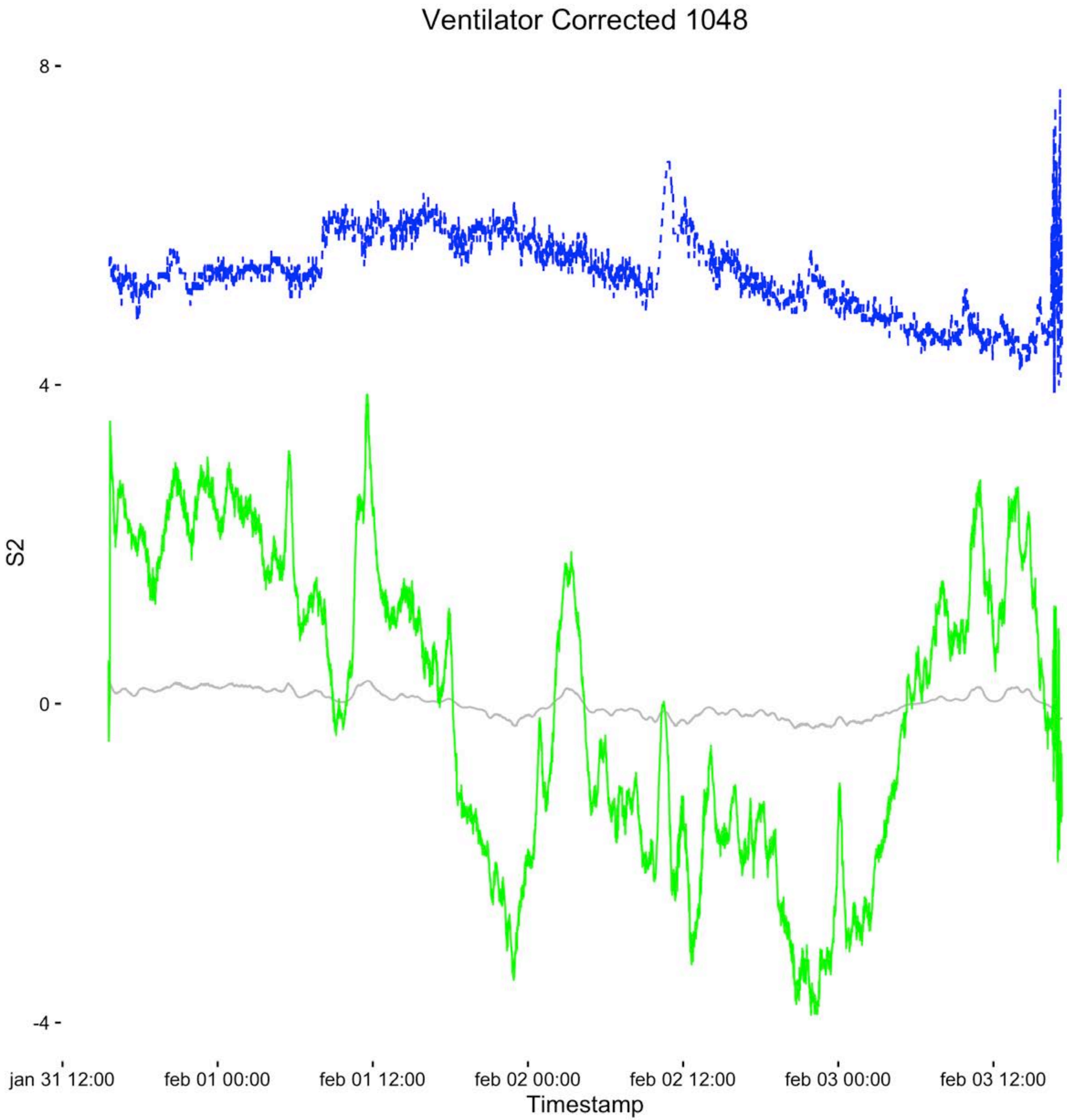
Ventilator Corrected 1048
8
4
0
-4
S2
jan 31 12:00
feb 01 00:00
feb 01 12:00
feb 02 00:00
feb 02 12:00
feb 03 00:00
feb 03 12:00
Timestamp

Ventilator Corrected 1050

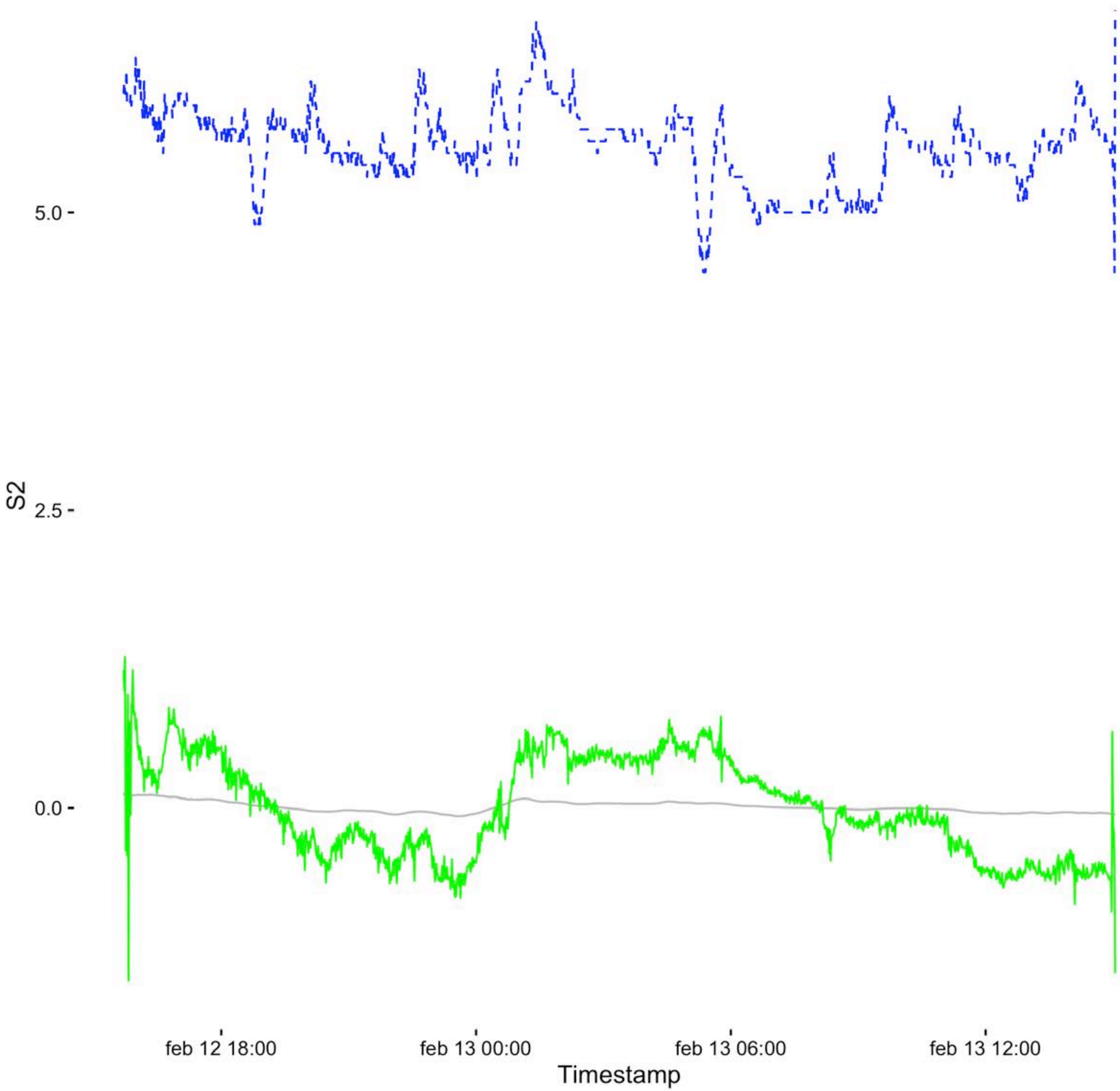

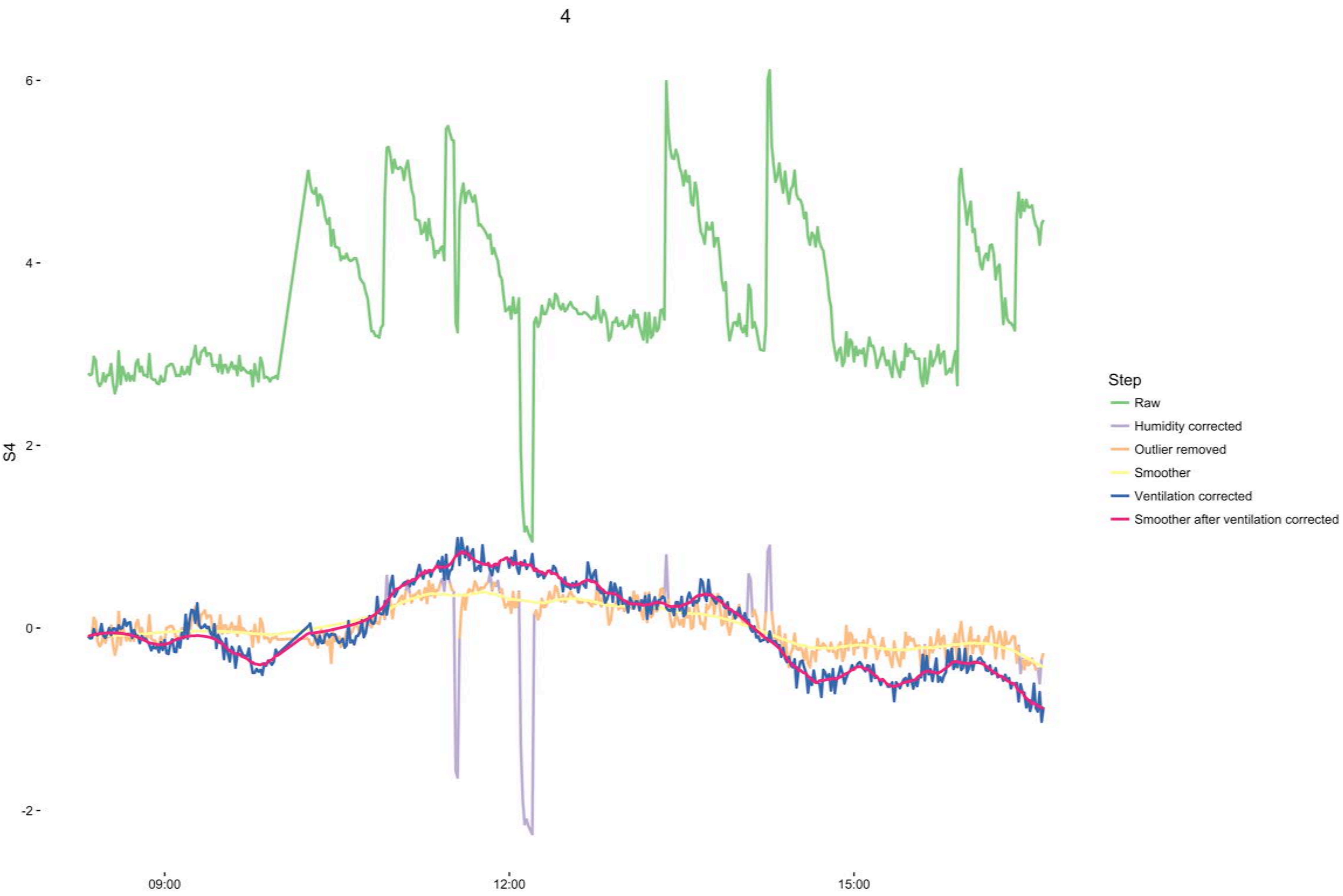
4
S4
6
4
2
0
-2
09:00
12:00
15:00
Step
Raw
Humidity corrected
Outlier removed
Smoother
Ventilation corrected
Smoother after ventilation corrected

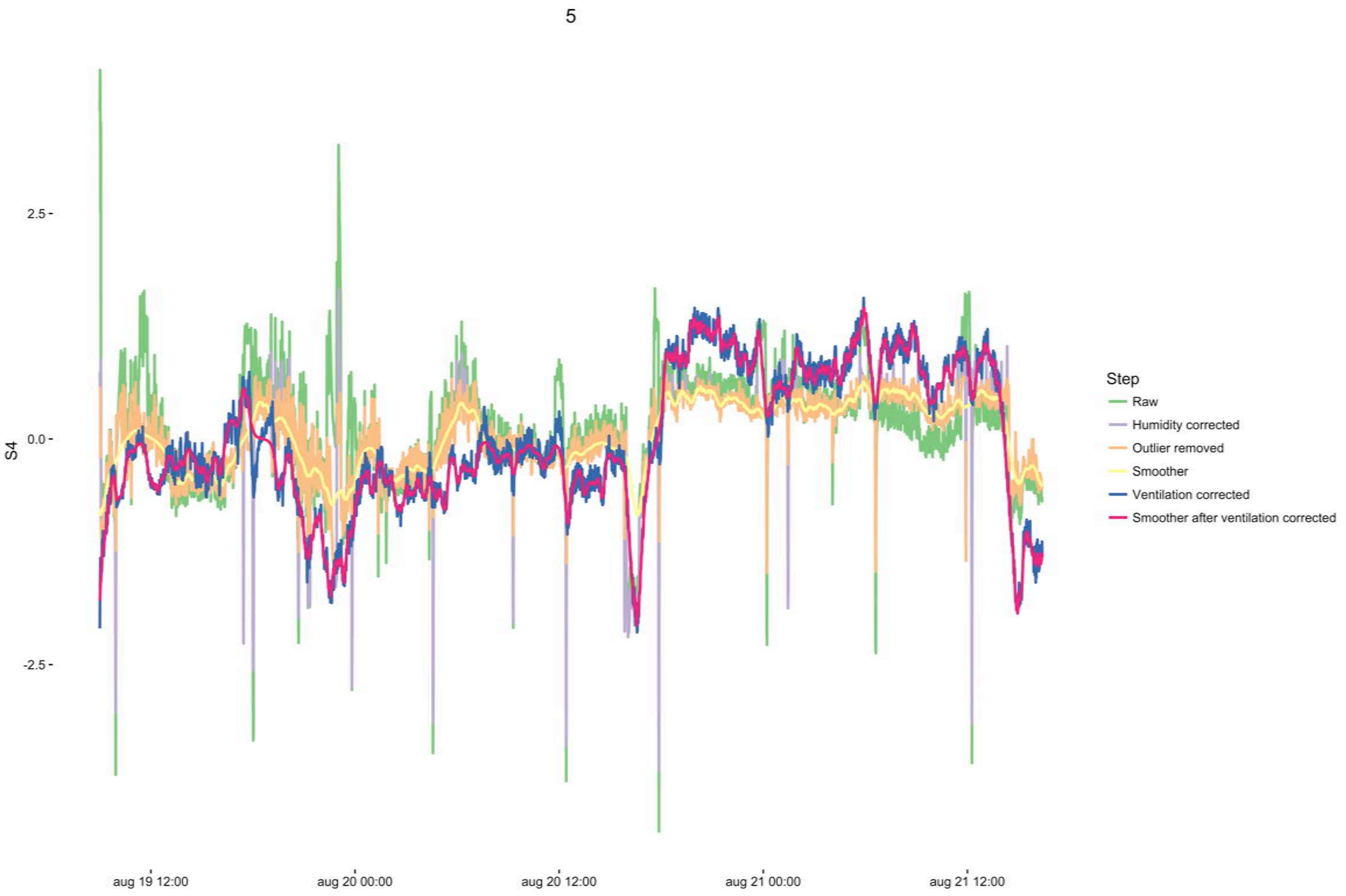
5
S4
2.5
0.0
-2.5
aug 19 12:00
aug 20 00:00
aug 20 12:00
aug 21 00:00
aug 21 12:00
Step
Raw
Humidity corrected
Outlier removed
Smoother
Ventilation corrected
Smoother after ventilation corrected

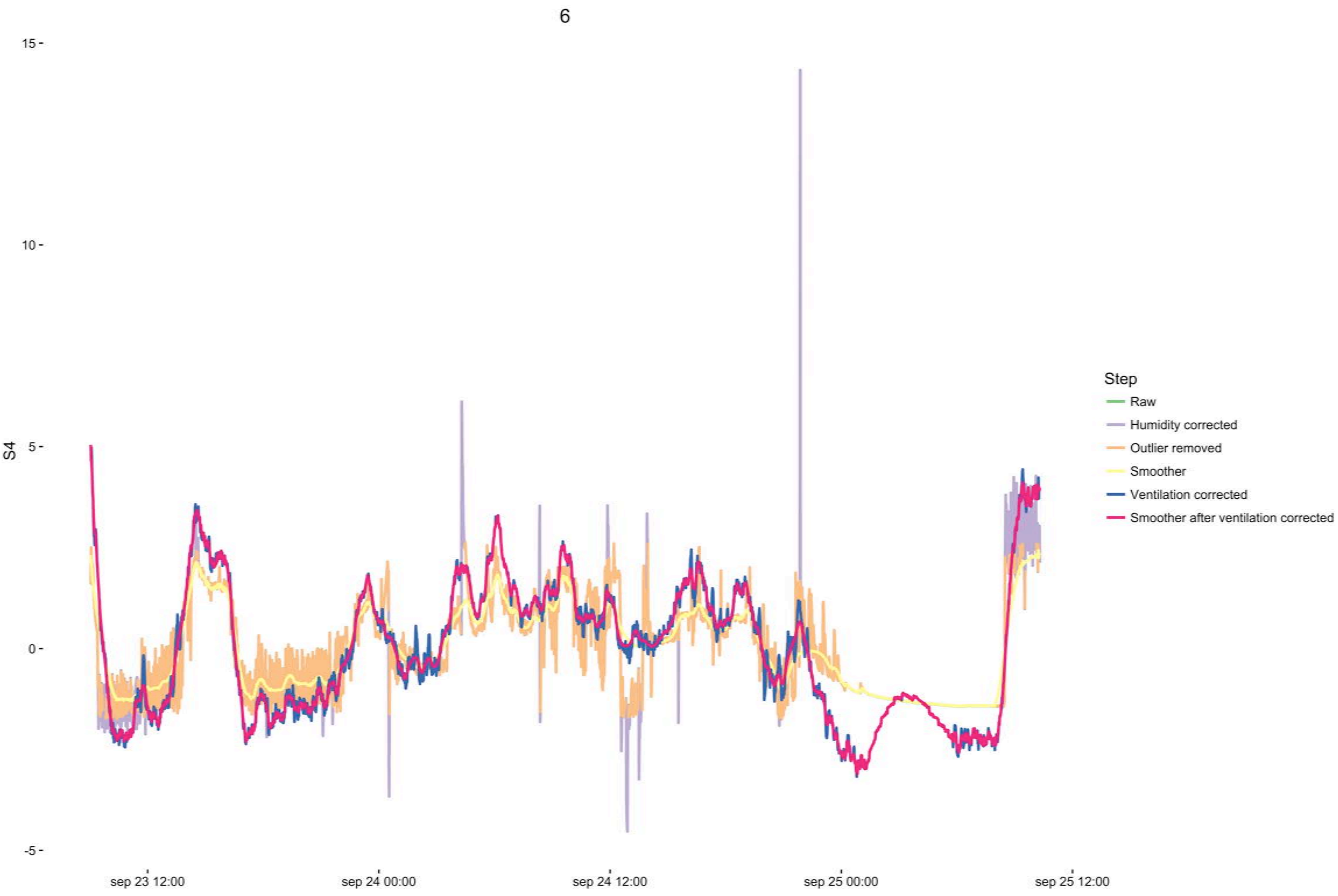
6
15
10
5
0
-5
S4
sep 23 12:00
sep 24 00:00
sep 24 12:00
sep 25 00:00
sep 25 12:00
Step
Raw
Humidity corrected
Outlier removed
Smoother
Ventilation corrected
Smoother after ventilation corrected

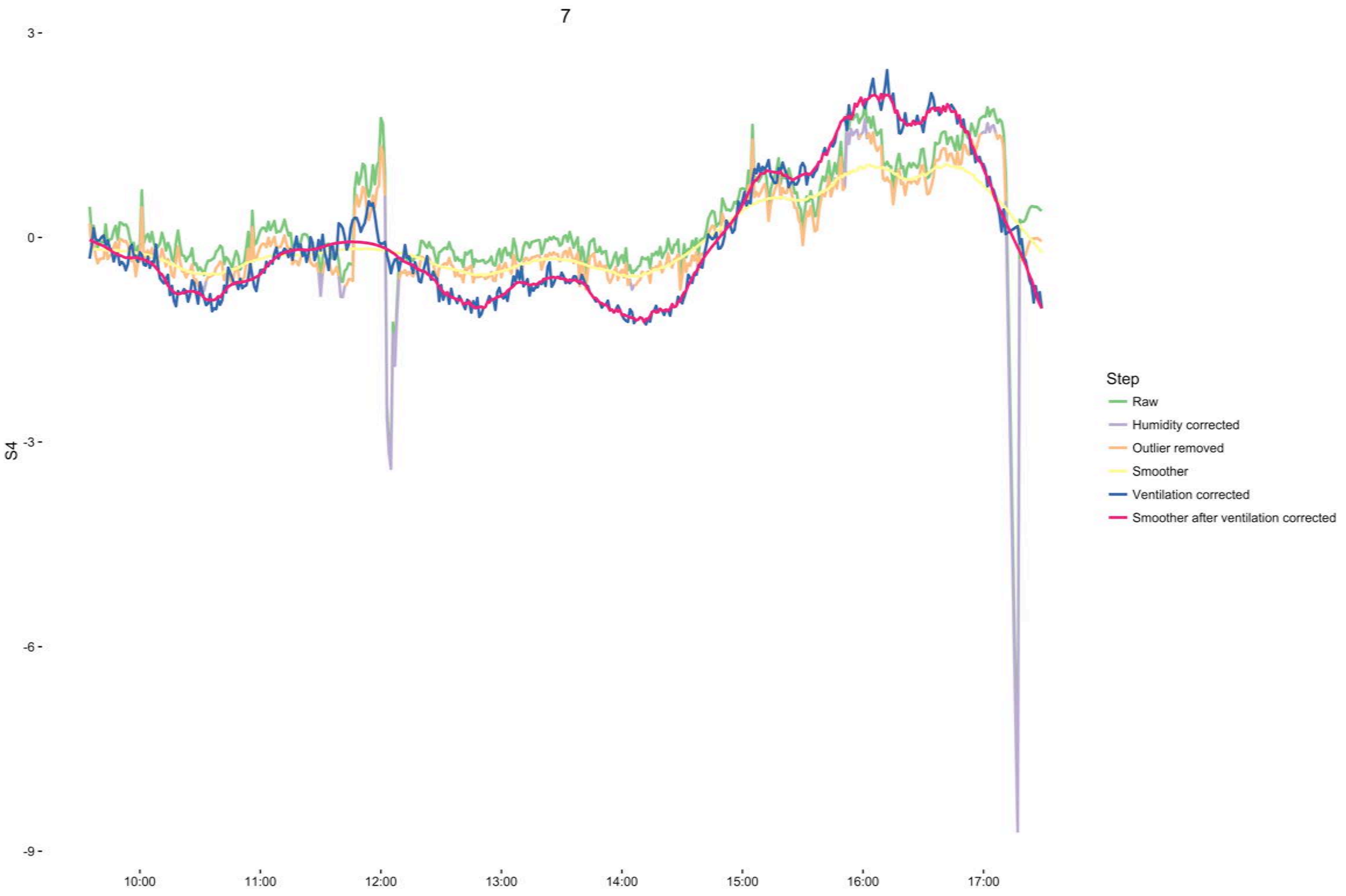
7
3
0
-3
-6
-9
S4
10:00
11:00
12:00
13:00
14:00
15:00
16:00
17:00
Step
Raw
Humidity corrected
Outlier removed
Smoother
Ventilation corrected
Smoother after ventilation corrected

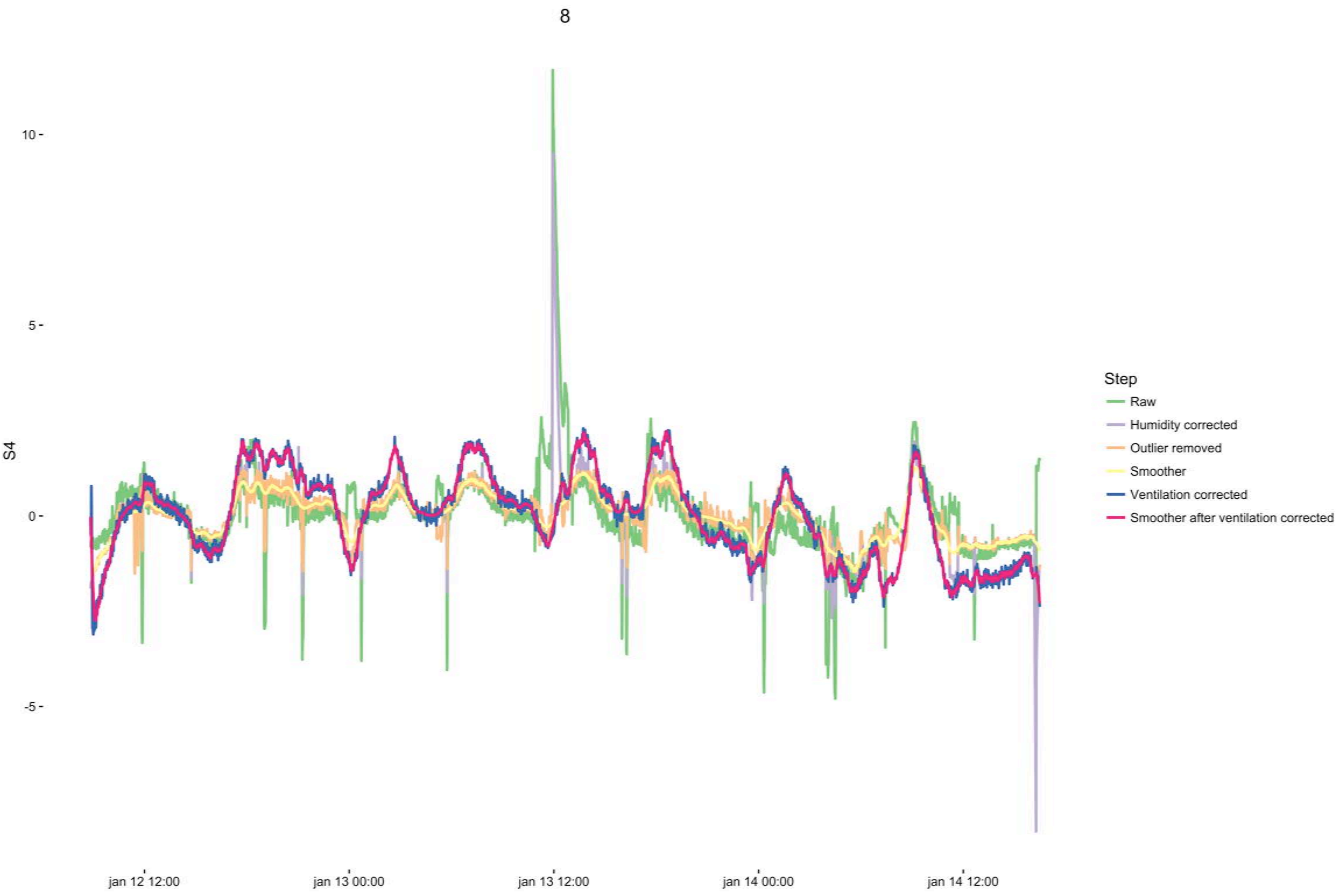
8
10
5
0
-5
S4
jan 12 12:00
jan 13 00:00
jan 13 12:00
jan 14 00:00
jan 14 12:00
Step
Raw
Humidity corrected
Outlier removed
Smoother
Ventilation corrected
Smoother after ventilation corrected

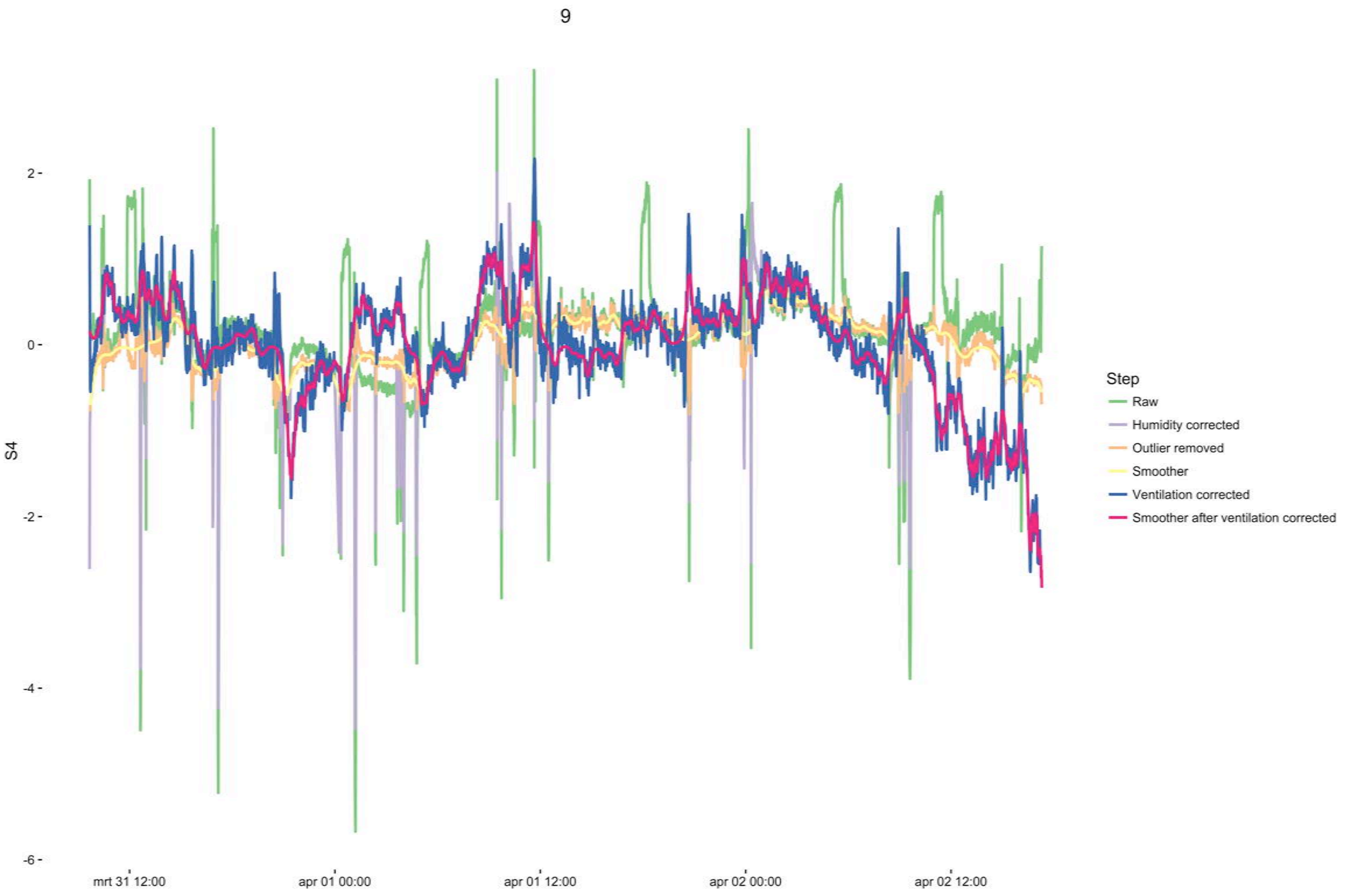

9
S4
2
0
-2
-4
-6
mrt 31 12:00
apr 01 00:00
apr 01 12:00
apr 02 00:00
apr 02 12:00
Step
Raw
Humidity corrected
Outlier removed
Smoother
Ventilation corrected
Smoother after ventilation corrected

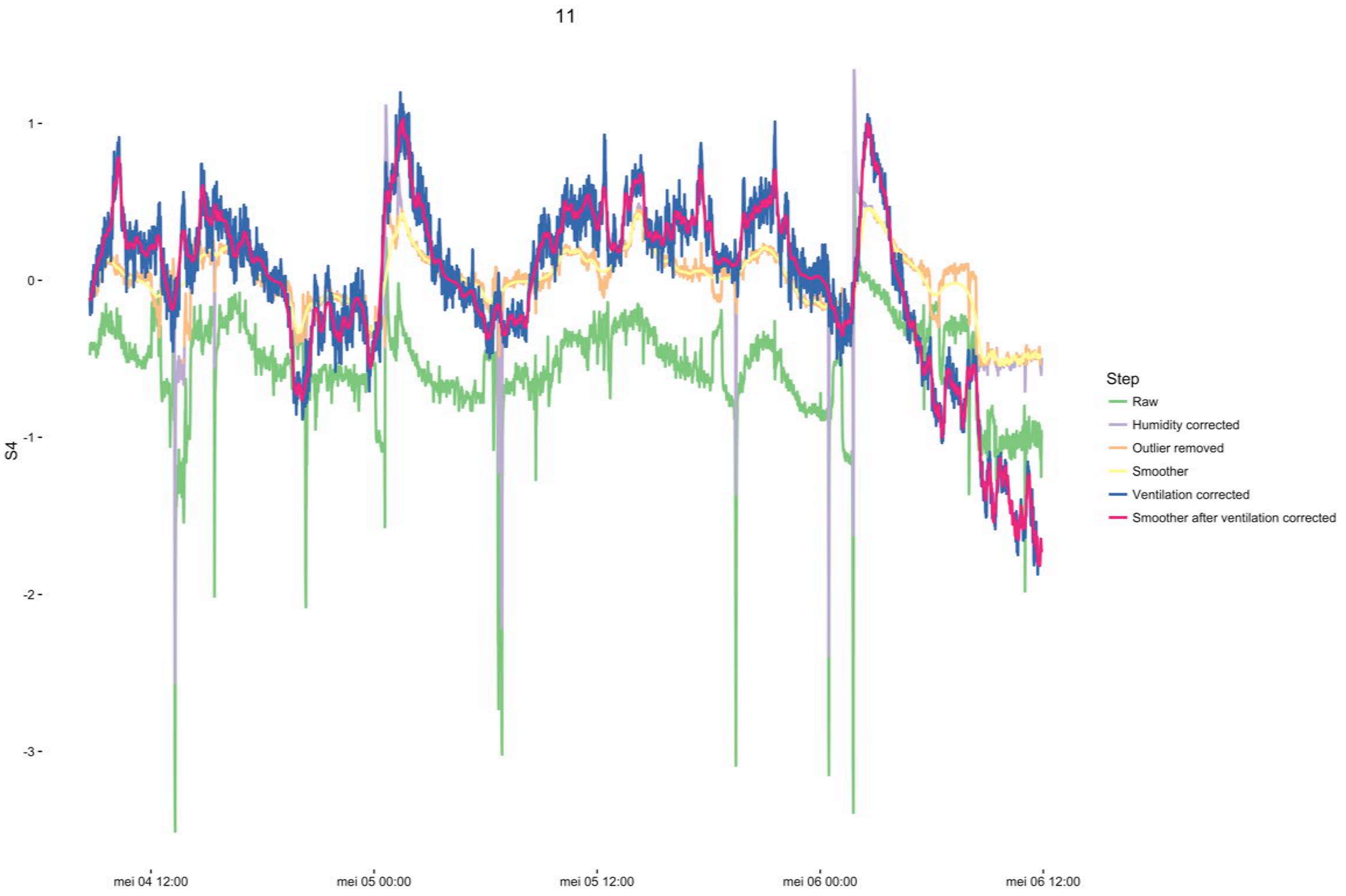
11
S4
1
0
-1
-2
-3
mei 04 12:00
mei 05 00:00
mei 05 12:00
mei 06 00:00
mei 06 12:00
Step
Raw
Humidity corrected
Outlier removed
Smoother
Ventilation corrected
Smoother after ventilation corrected

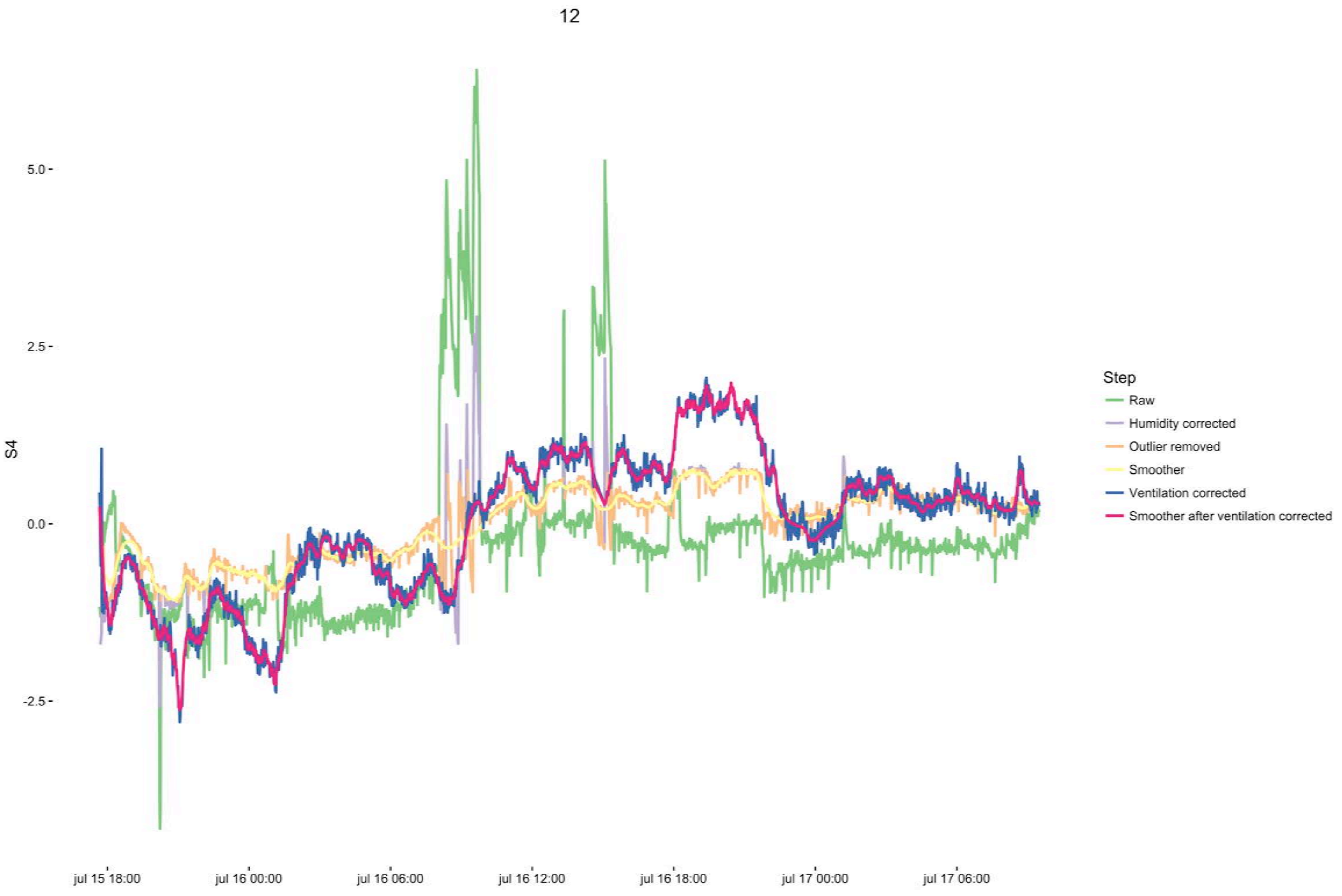
12
S4
5.0
2.5
0.0
-2.5
jul 15 18:00
jul 16 00:00
jul 16 06:00
jul 16 12:00
jul 16 18:00
jul 17 00:00
jul 17 06:00
Step
Raw
Humidity corrected
Outlier removed
Smoother
Ventilation corrected
Smoother after ventilation corrected

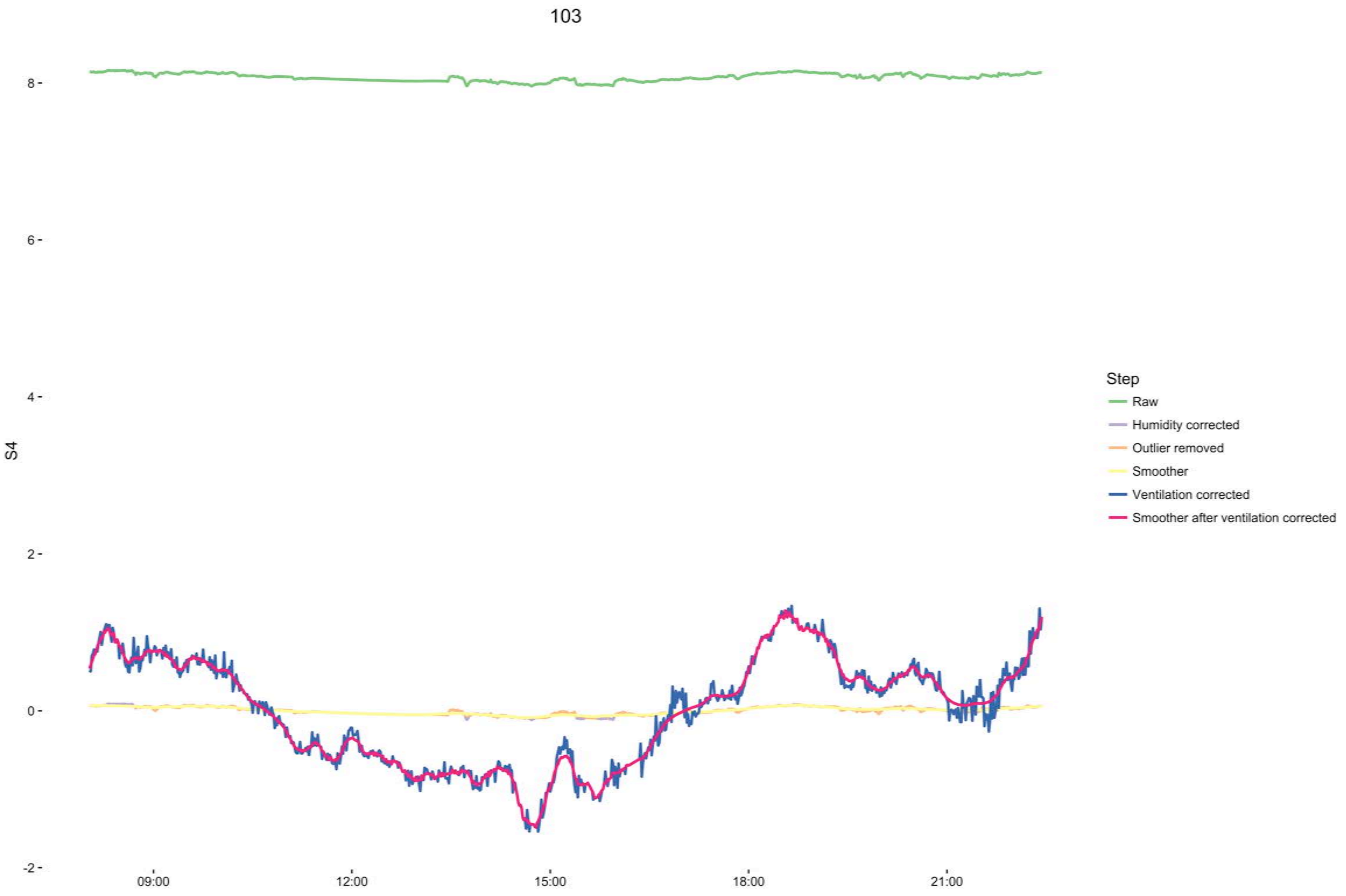

103
S4
8
6
4
2
0
-2
09:00
12:00
15:00
18:00
21:00
Step
Raw
Humidity corrected
Outlier removed
Smoother
Ventilation corrected
Smoother after ventilation corrected

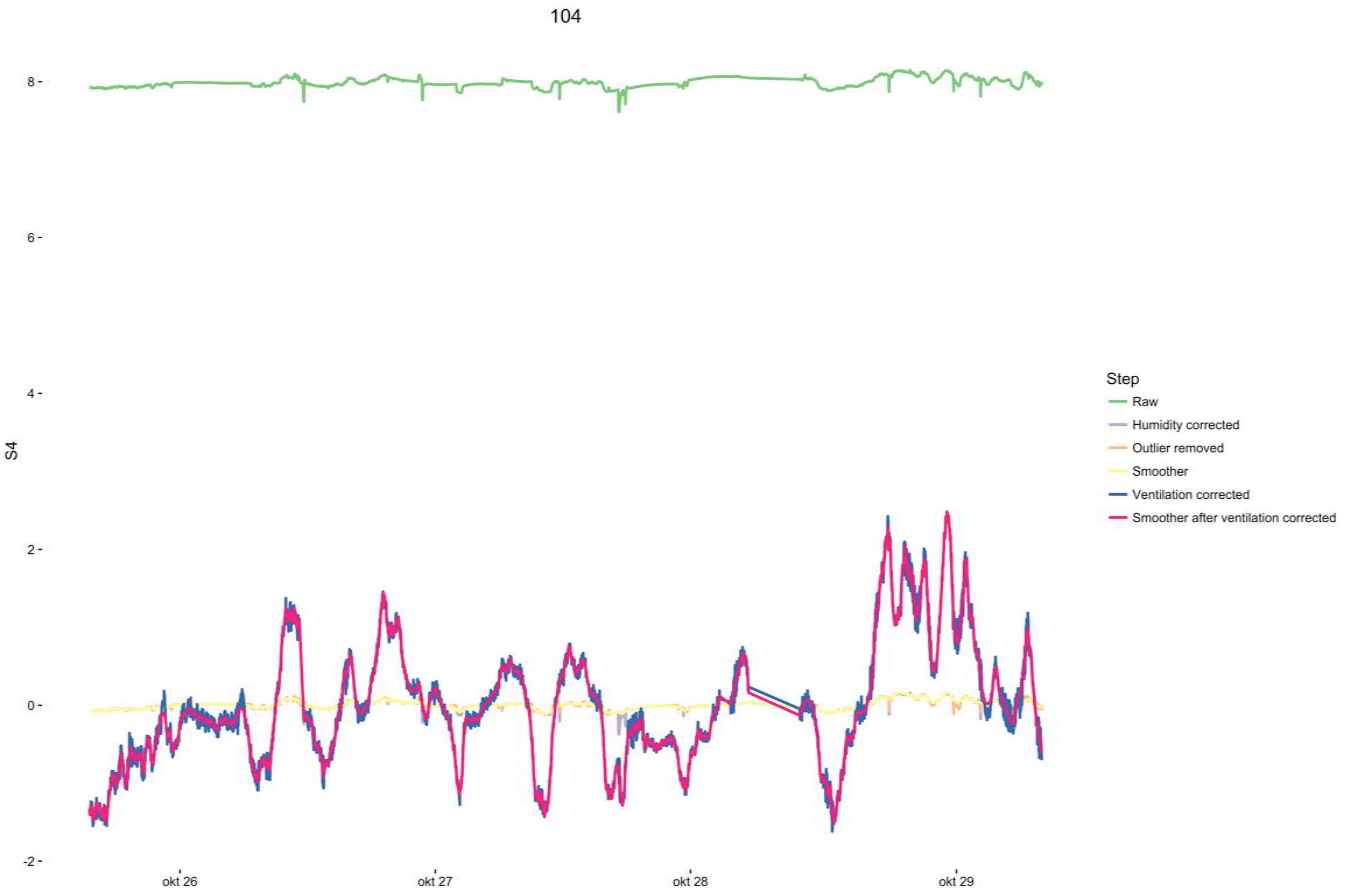

104
S4
8
6
4
2
0
-2
okt 26
okt 27
okt 28
okt 29
Step
Raw
Humidity corrected
Outlier removed
Smoother
Ventilation corrected
Smoother after ventilation corrected

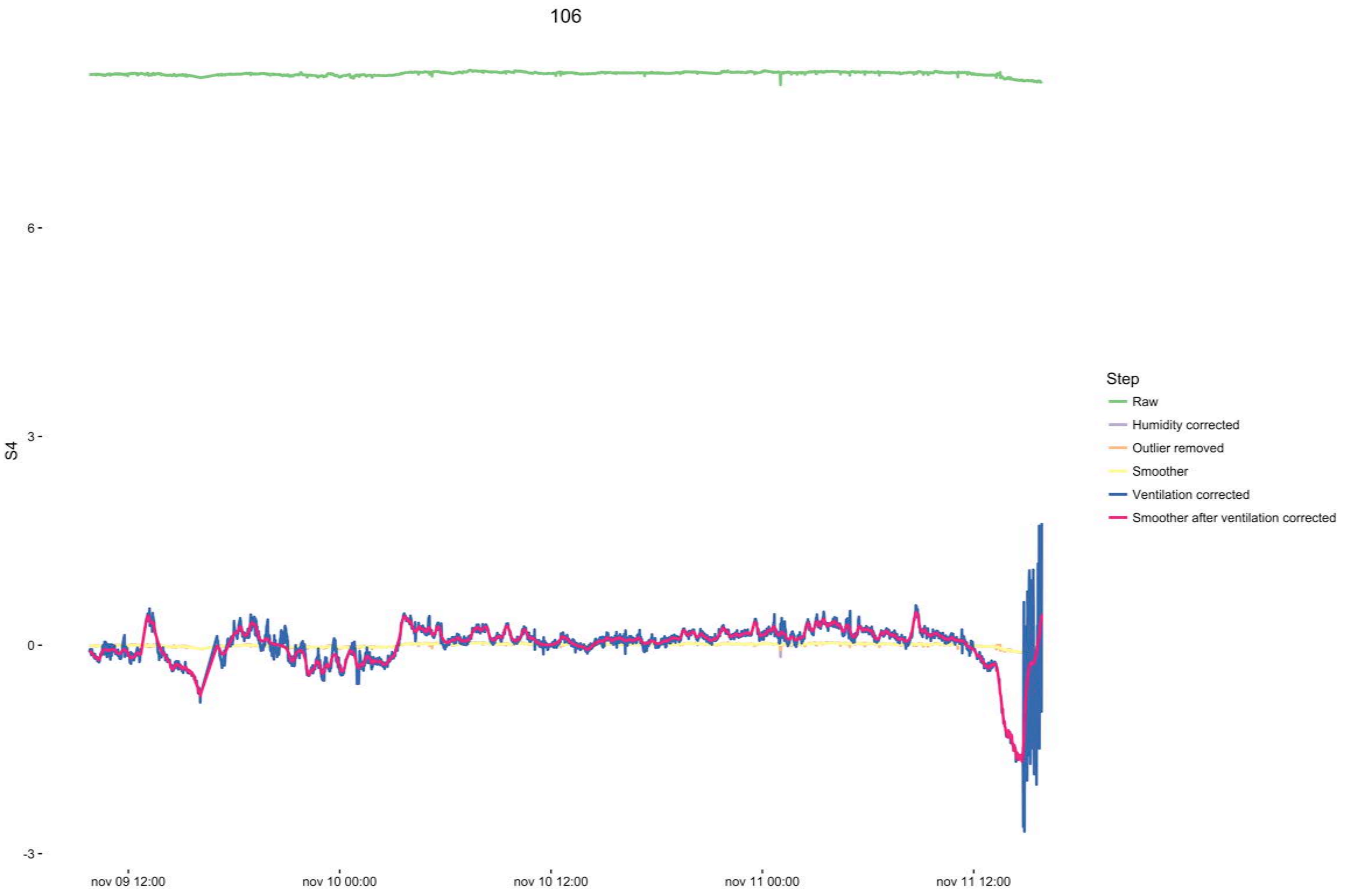

106
6
3
0
-3
S4
nov 09 12:00
nov 10 00:00
nov 10 12:00
nov 11 00:00
nov 11 12:00
Step
Raw
Humidity corrected
Outlier removed
Smoother
Ventilation corrected
Smoother after ventilation corrected

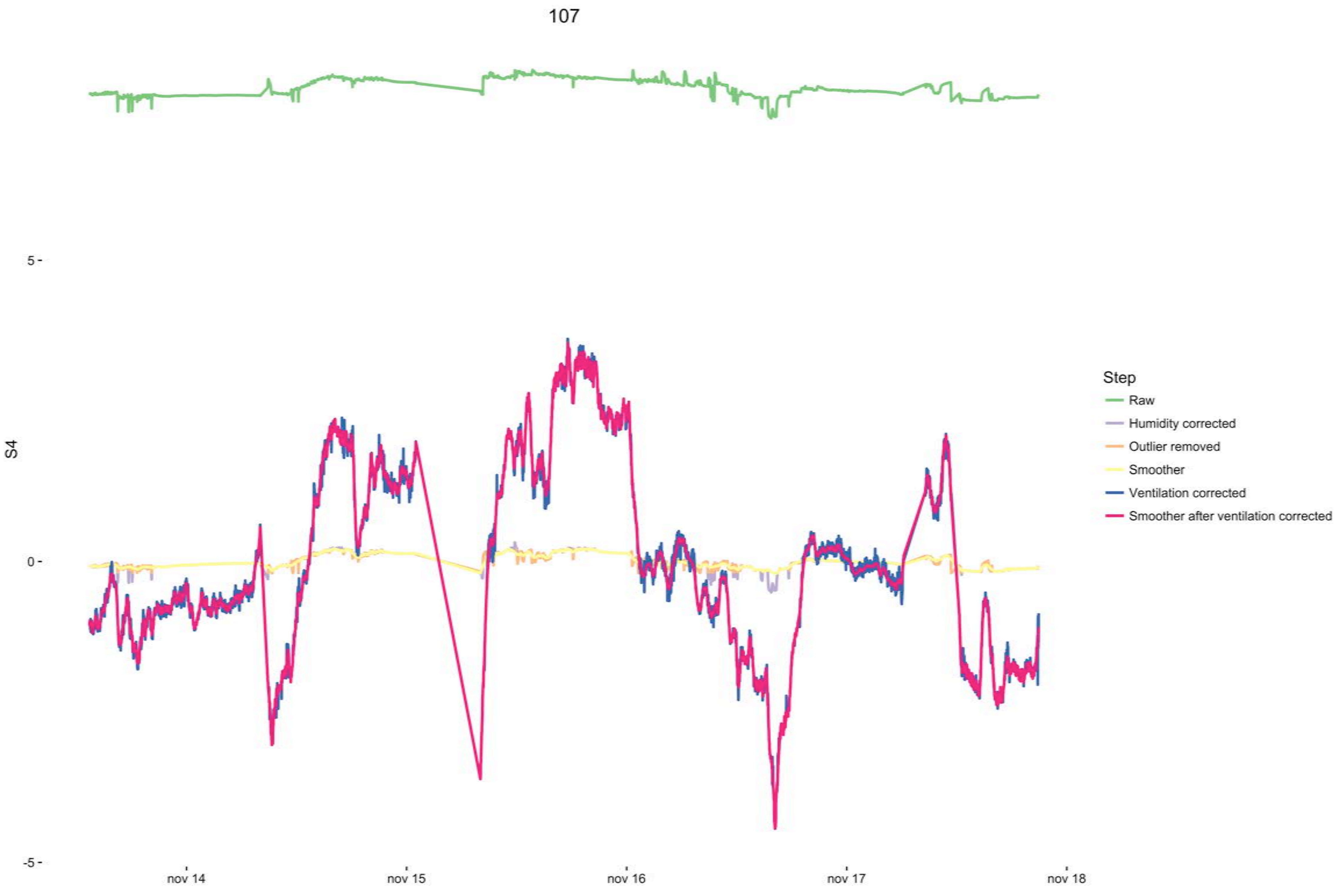
107
5
0
-5
S4
nov 14
nov 15
nov 16
nov 17
nov 18
Step
Raw
Humidity corrected
Outlier removed
Smoother
Ventilation corrected
Smoother after ventilation corrected

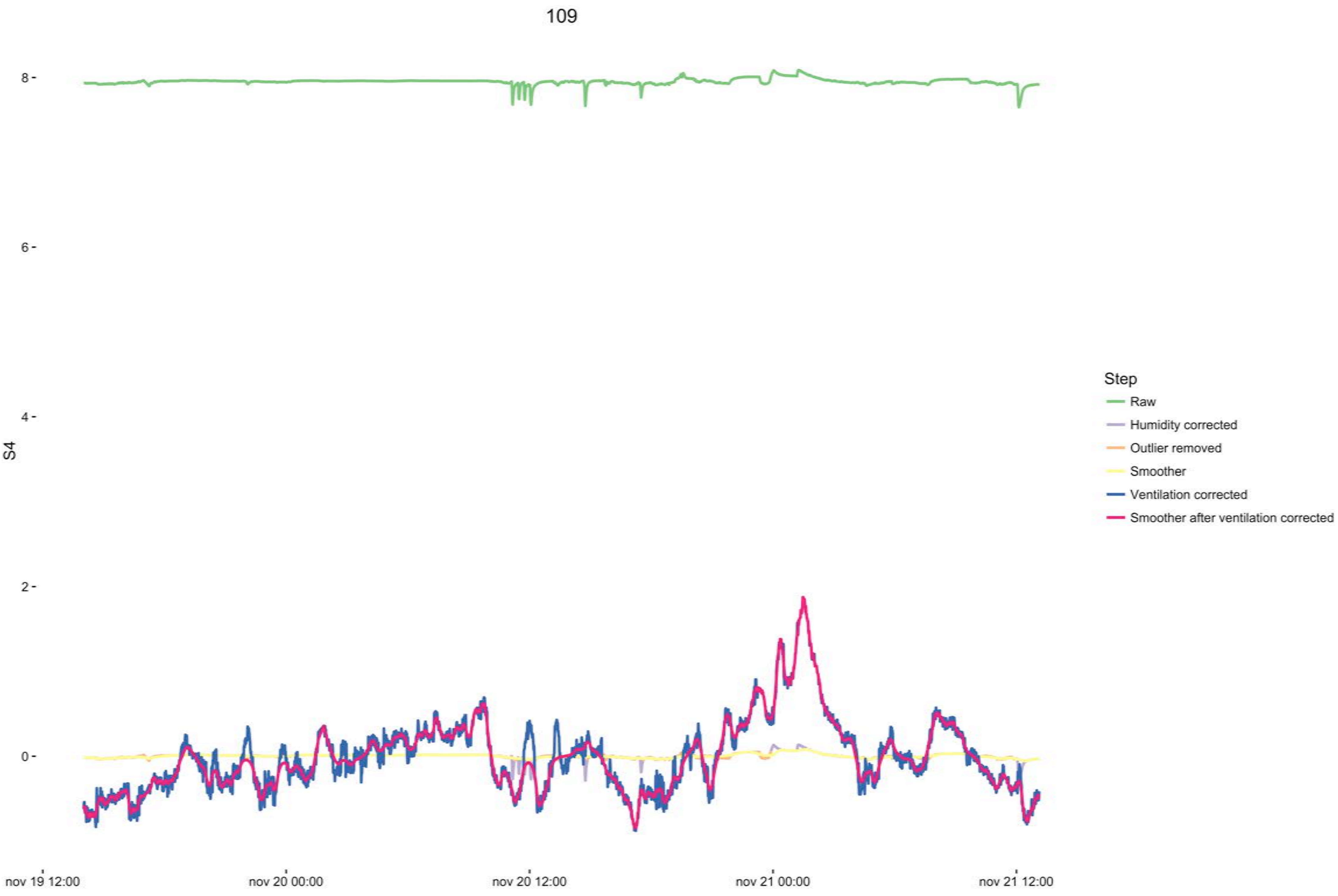

109
8
6
4
2
0
S4
nov 19 12:00
nov 20 00:00
nov 20 12:00
nov 21 00:00
nov 21 12:00
Step
Raw
Humidity corrected
Outlier removed
Smoother
Ventilation corrected
Smoother after ventilation corrected

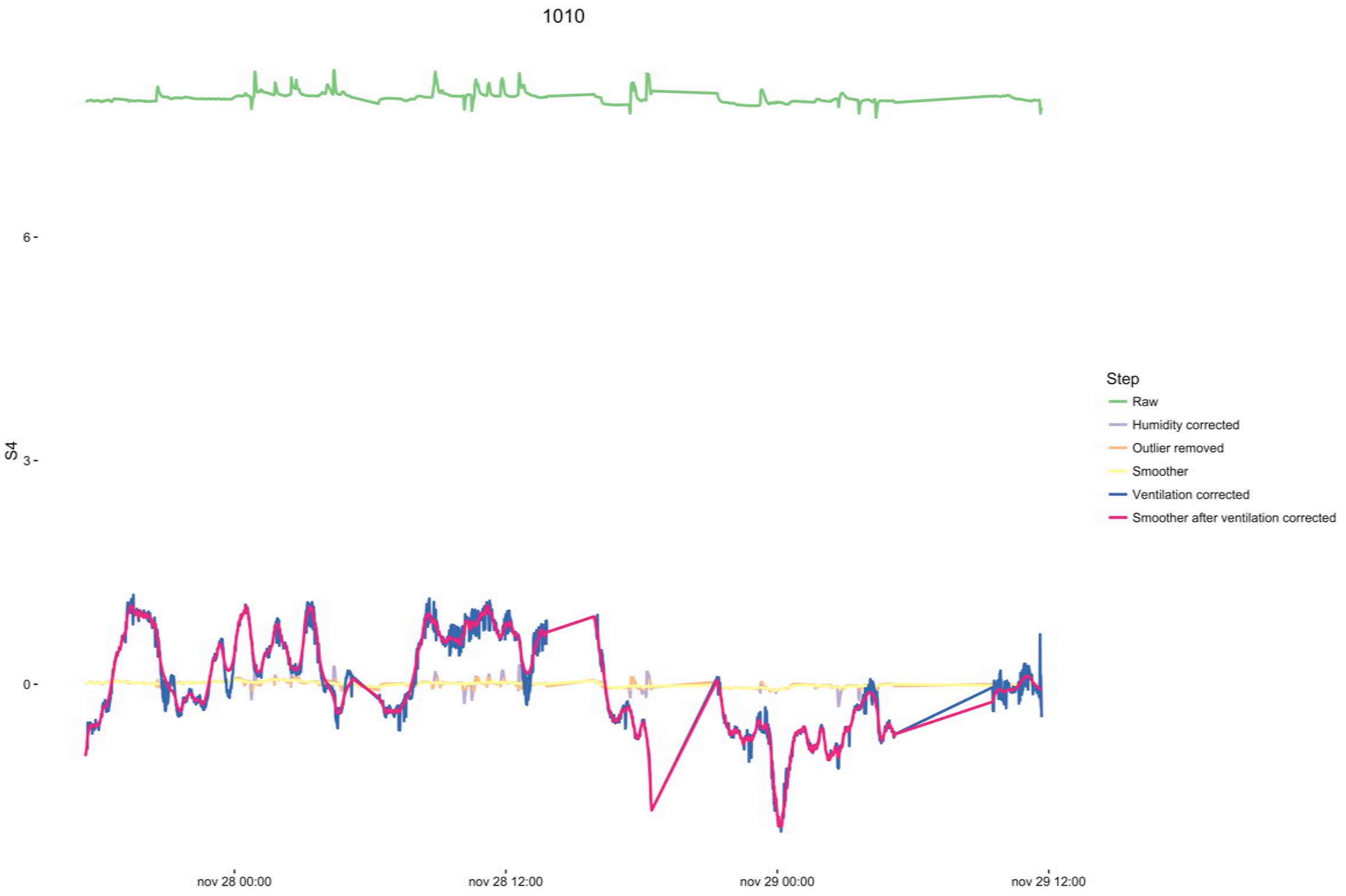
1010
6 -
3 -
0 -
S4
nov 28 00:00
nov 28 12:00
nov 29 00:00
nov 29 12:00
Step
Raw
Humidity corrected
Outlier removed
Smoother
Ventilation corrected
Smoother after ventilation corrected

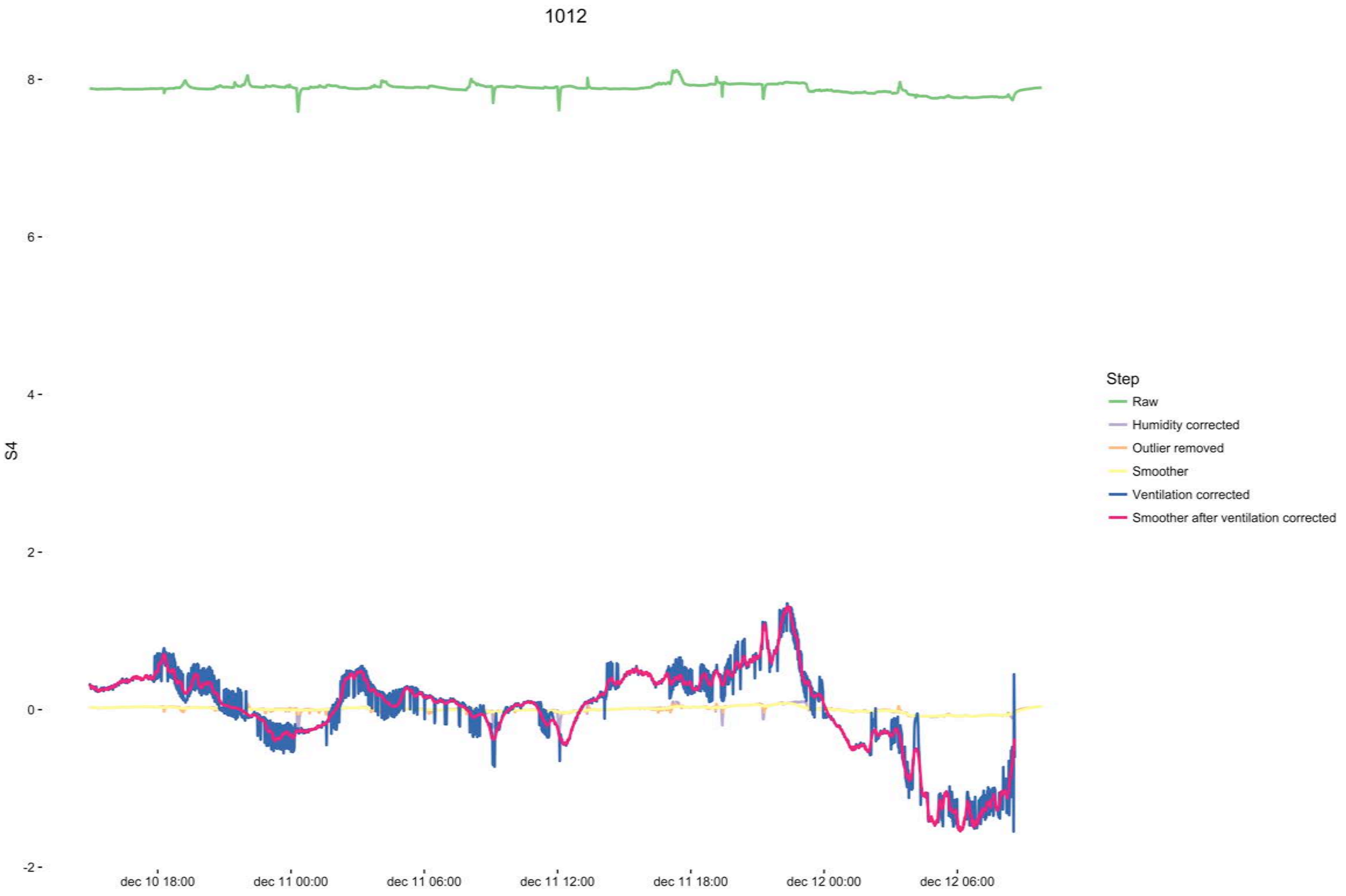

1012
8
6
4
2
0
-2
S4
dec 10 18:00
dec 11 00:00
dec 11 06:00
dec 11 12:00
dec 11 18:00
dec 12 00:00
dec 12 06:00
Step
Raw
Humidity corrected
Outlier removed
Smoother
Ventilation corrected
Smoother after ventilation corrected

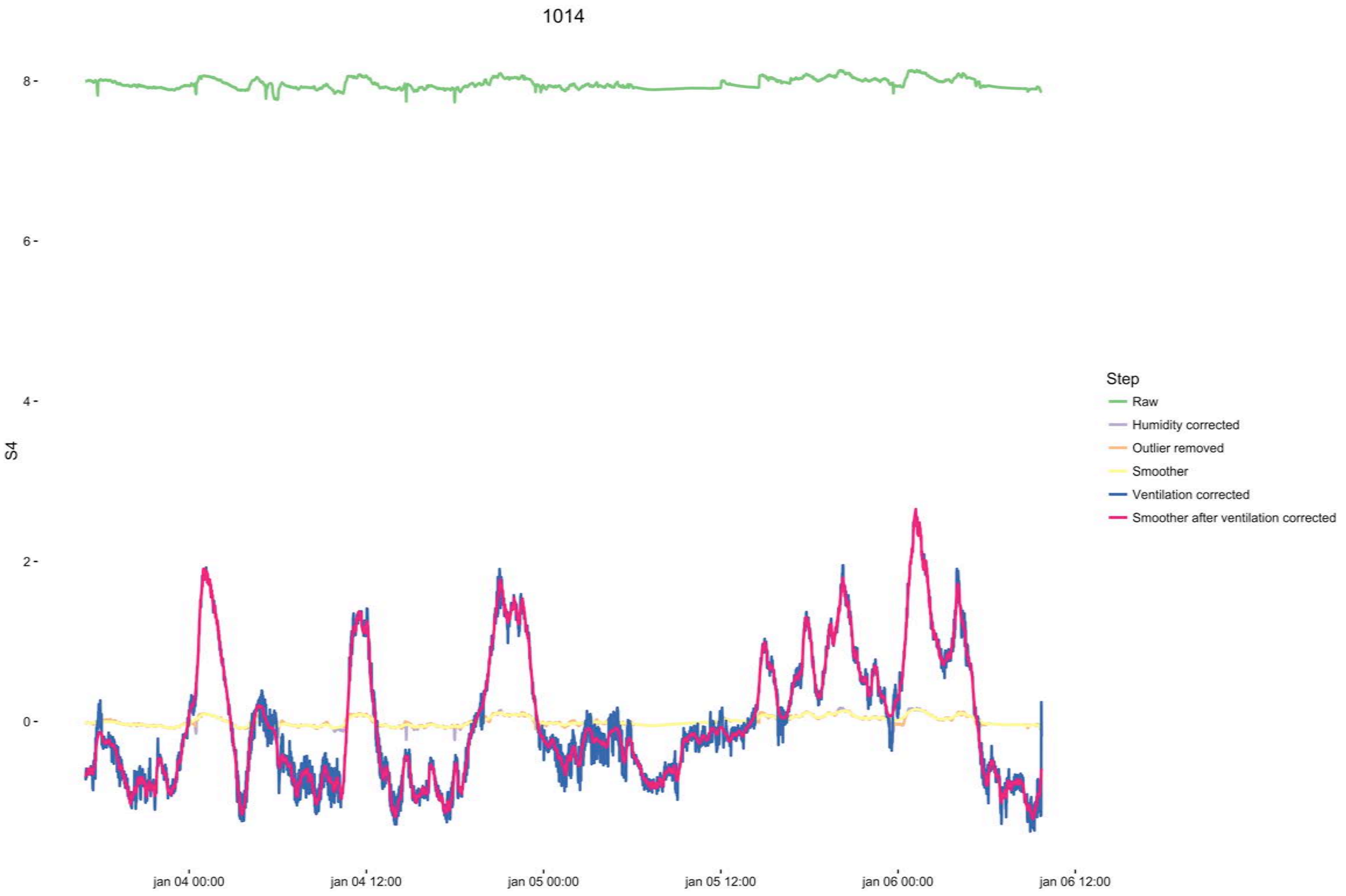

1014
S4
8
6
4
2
0
jan 04 00:00
jan 04 12:00
jan 05 00:00
jan 05 12:00
jan 06 00:00
jan 06 12:00
Step
Raw
Humidity corrected
Outlier removed
Smoother
Ventilation corrected
Smoother after ventilation corrected

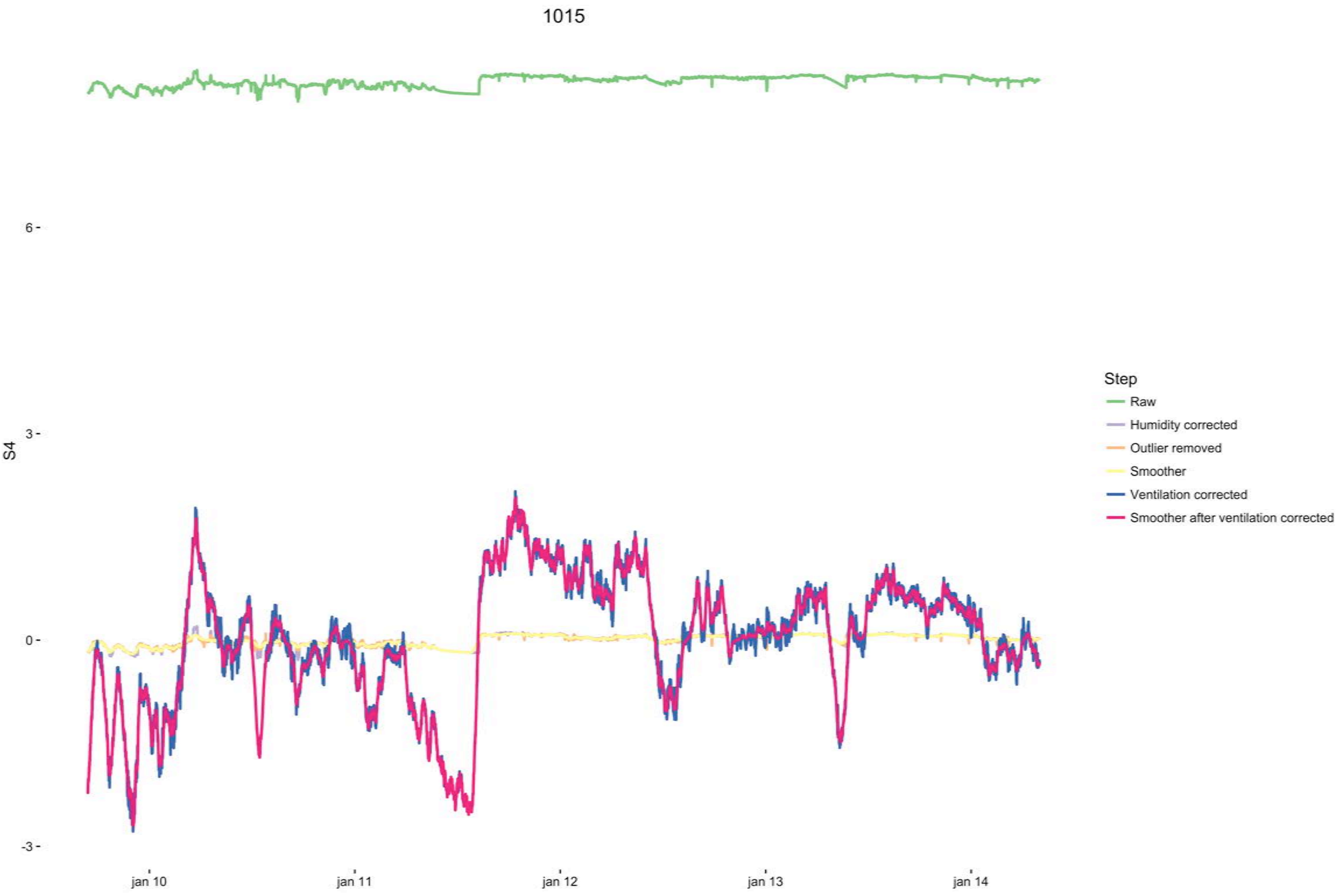
1015
6
3
0
-3
S4
jan 10
jan 11
jan 12
jan 13
jan 14
Step
Raw
Humidity corrected
Outlier removed
Smoother
Ventilation corrected
Smoother after ventilation corrected

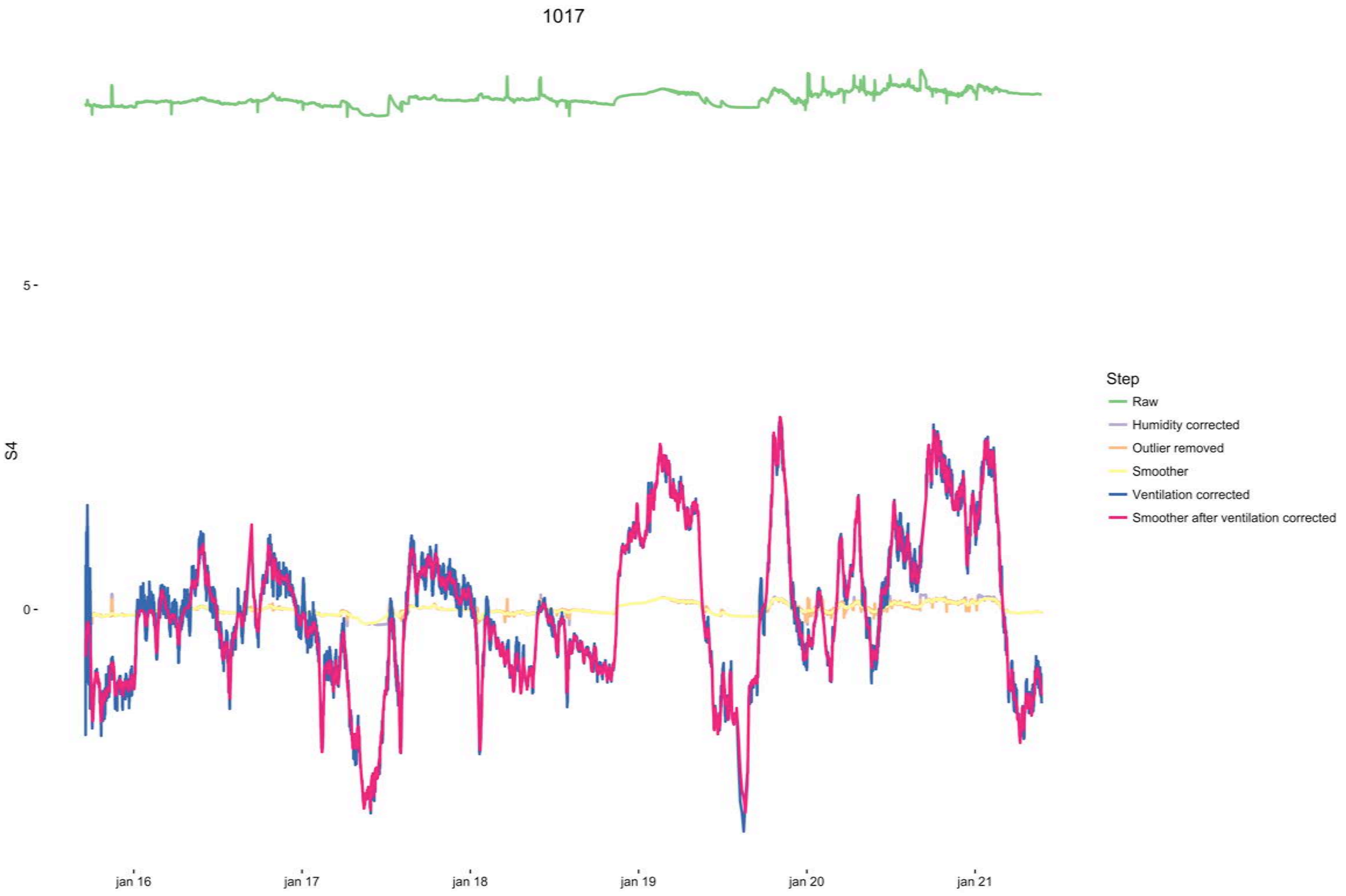
1017
5 -
0 -
S4
jan 16
jan 17
jan 18
jan 19
jan 20
jan 21
Step
Raw
Humidity corrected
Outlier removed
Smoother
Ventilation corrected
Smoother after ventilation corrected

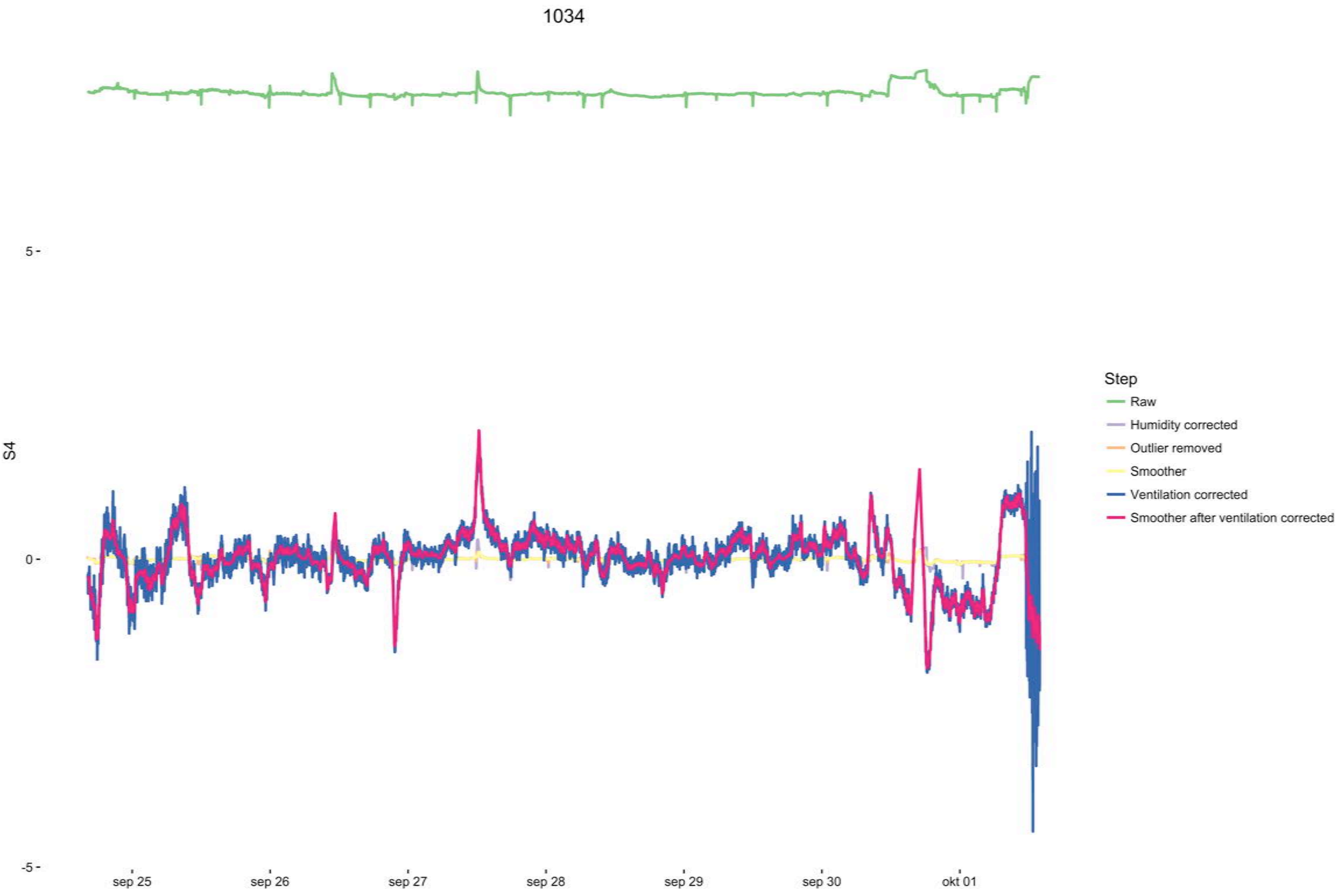

1034
S4
5
0
-5
sep 25
sep 26
sep 27
sep 28
sep 29
sep 30
okt 01
Step
Raw
Humidity corrected
Outlier removed
Smoother
Ventilation corrected
Smoother after ventilation corrected

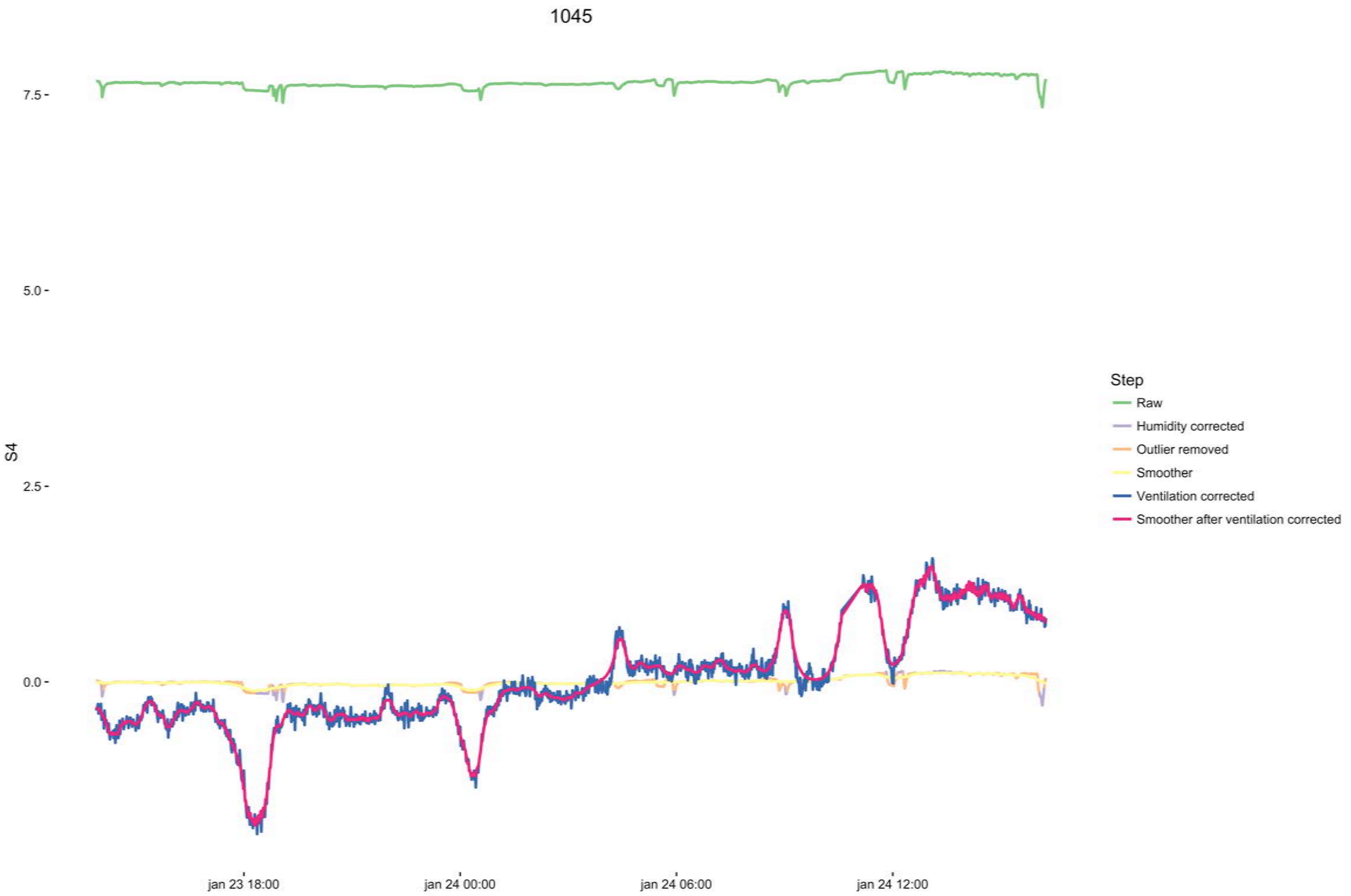
1045
7.5
5.0
2.5
0.0
S4
jan 23 18:00
jan 24 00:00
jan 24 06:00
jan 24 12:00
Step
Raw
Humidity corrected
Outlier removed
Smoother
Ventilation corrected
Smoother after ventilation corrected

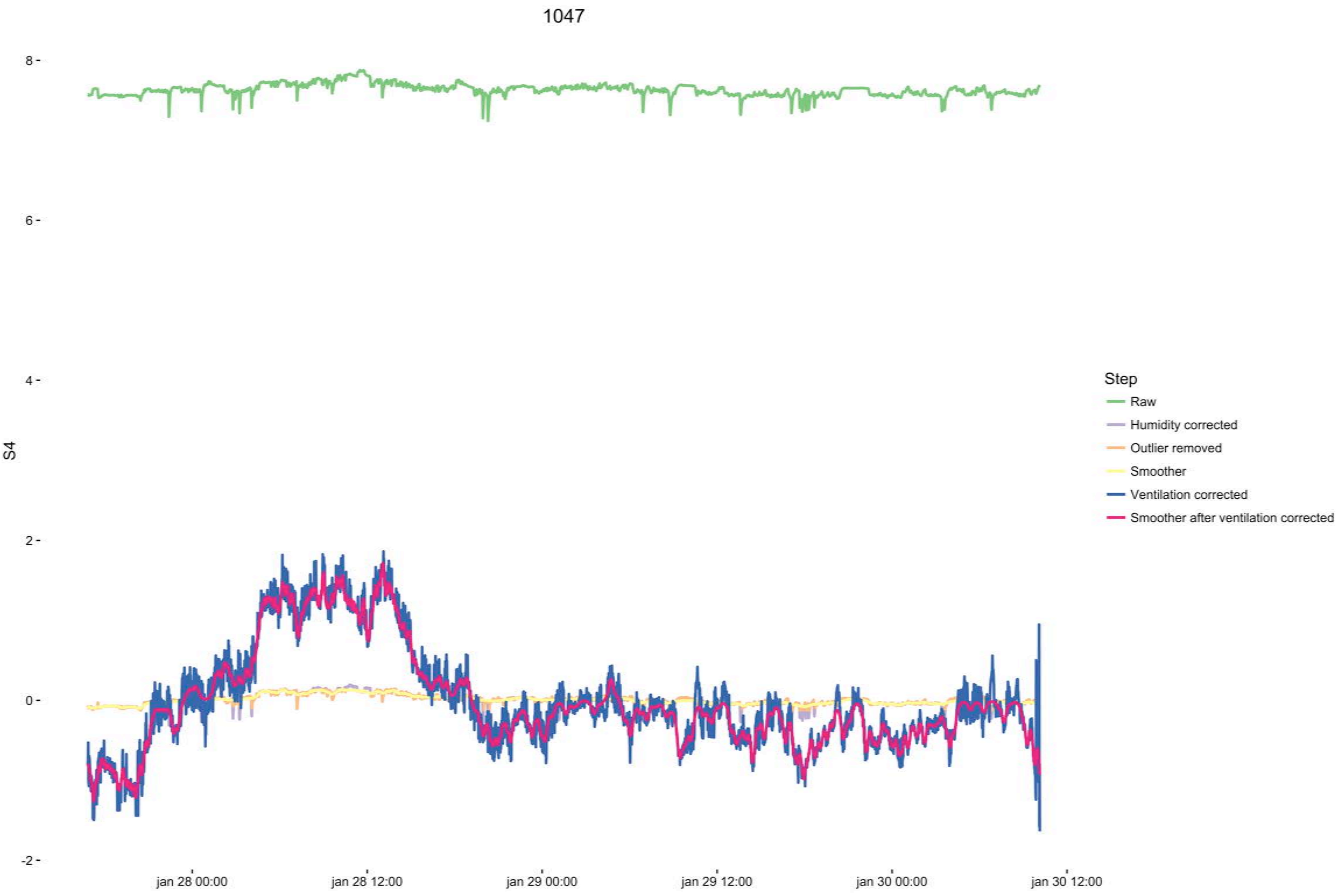
1047
8
6
4
2
0
-2
S4
jan 28 00:00
jan 28 12:00
jan 29 00:00
jan 29 12:00
jan 30 00:00
jan 30 12:00
Step
Raw
Humidity corrected
Outlier removed
Smoother
Ventilation corrected
Smoother after ventilation corrected

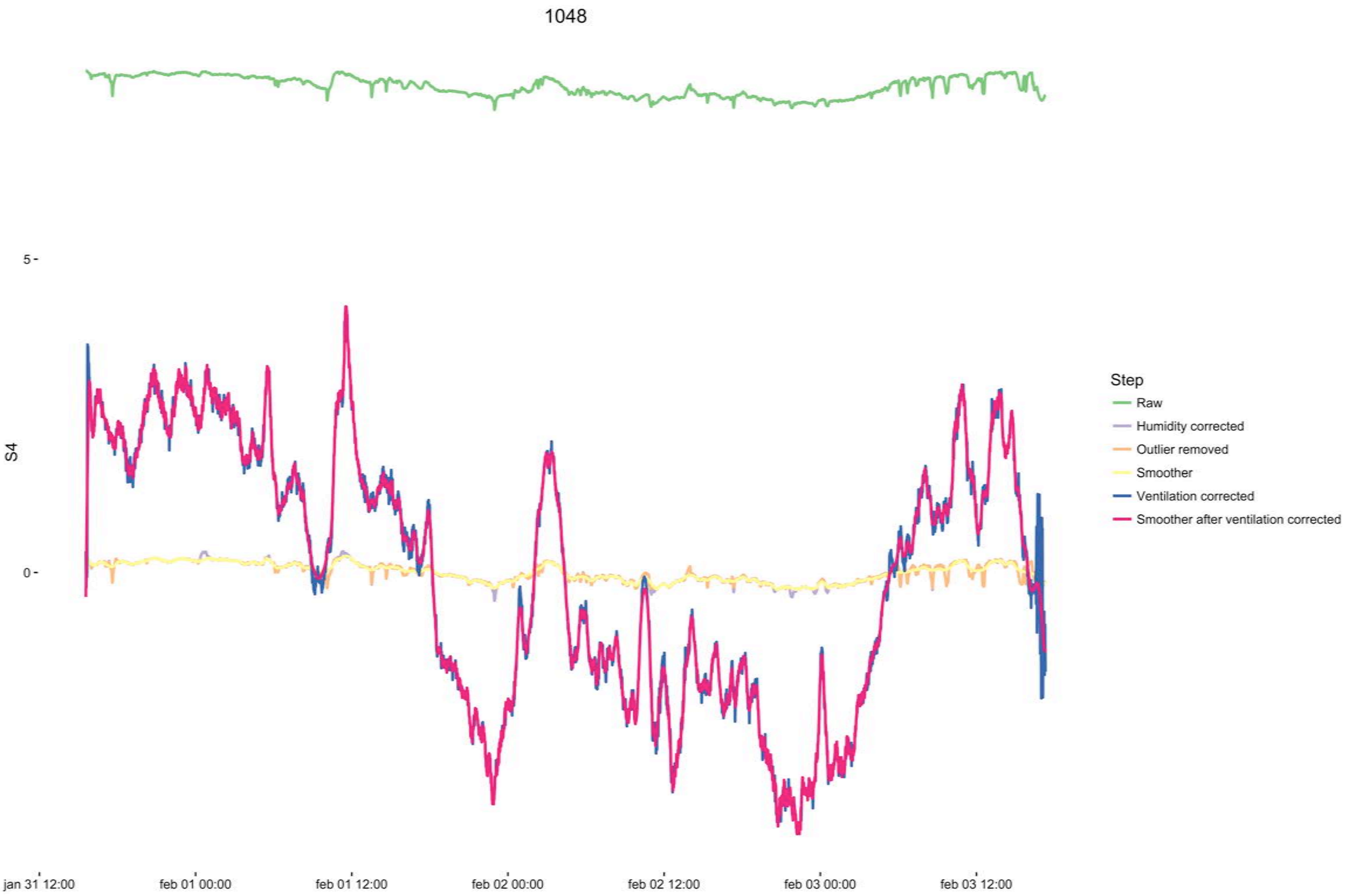
1048
5
0
S4
jan 31 12:00
feb 01 00:00
feb 01 12:00
feb 02 00:00
feb 02 12:00
feb 03 00:00
feb 03 12:00
Step
Raw
Humidity corrected
Outlier removed
Smoother
Ventilation corrected
Smoother after ventilation corrected

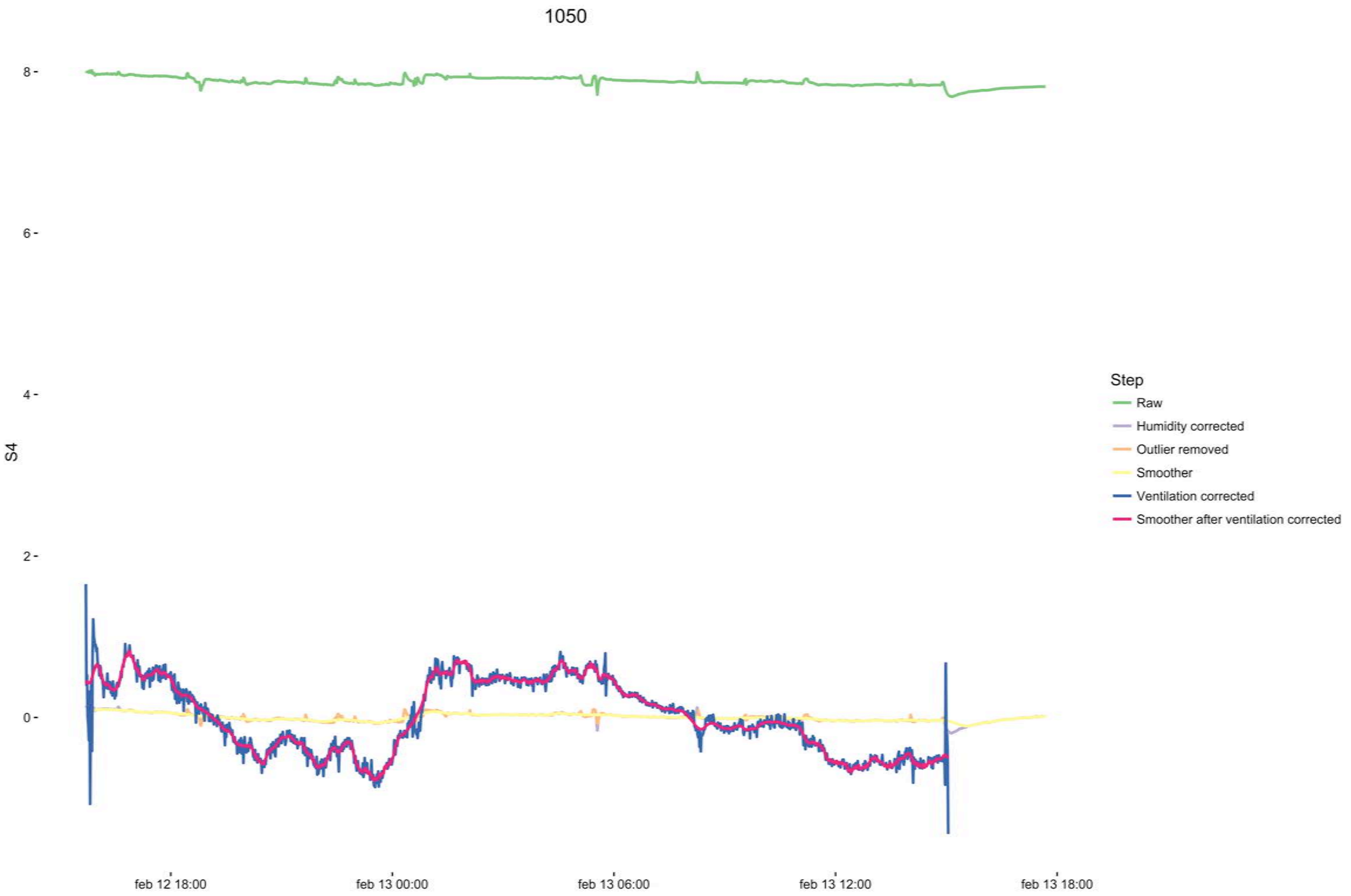
1050
8
6
4
2
0
S4
feb 12 18:00
feb 13 00:00
feb 13 06:00
feb 13 12:00
feb 13 18:00
Step
Raw
Humidity corrected
Outlier removed
Smoother
Ventilation corrected
Smoother after ventilation corrected
